# Untargeted serum metabolic profiling of diabetes mellitus among Parkinson’s disease patients

**DOI:** 10.1038/s41531-024-00711-4

**Published:** 2024-05-10

**Authors:** Shiwen Li, Yuyuan Lin, Dean Jones, Douglas I. Walker, Aline Duarte Folle, Irish Del Rosario, Yu Yu, Keren Zhang, Adrienne M. Keener, Jeff Bronstein, Beate Ritz, Kimberly C. Paul

**Affiliations:** 1 Department of Epidemiology, UCLA Fielding School of Public Health, Los Angeles, CA USA; 2 Division of Pulmonary, Allergy, Critical Care and Sleep Medicine, Department of Medicine, Emory University School of Medicine, Atlanta, USA; 3 Department of Biochemistry, Emory University School of Medicine, Atlanta, USA; 4 Gangarosa Department of Environmental Health, Rollins School of Public Health, Emory University, Atlanta, GA USA; 5 Center for Health Policy Research, UCLA Fielding School of Public Health, Los Angeles, CA USA; 6 Department of Neurology, David Geffen School of Medicine, Los Angeles, CA USA

**Keywords:** Prognostic markers, Parkinson's disease

## Abstract

Type 2 diabetes mellitus (T2DM) is a common comorbidity among Parkinson’s disease (PD) patients. Yet, little is known about dysregulated pathways that are unique in PD patients with T2DM. We applied high-resolution metabolomic profiling in serum samples of 636 PD and 253 non-PD participants recruited from Central California. We conducted an initial discovery metabolome-wide association and pathway enrichment analysis. After adjusting for multiple testing, in positive (or negative) ion mode, 30 (25) metabolic features were associated with T2DM in both PD and non-PD participants, 162 (108) only in PD participants, and 32 (7) only in non-PD participants. Pathway enrichment analysis identified 17 enriched pathways associated with T2DM in both the PD and non-PD participants, 26 pathways only in PD participants, and 5 pathways only in non-PD participants. Several amino acid, nucleic acids, and fatty acid metabolisms were associated with T2DM only in the PD patient group suggesting a possible link between PD and T2DM.

## Introduction

Parkinson’s disease (PD) is a chronic neurodegenerative disease affecting roughly 8 million people globally^1^. Comorbidities frequently occur in PD patients and at a rate higher than similar age controls, which complicates the management of the disease^2–4^. This includes a variety of health conditions, including type 2 diabetes mellitus (T2DM)^2,5^. PD patients with comorbidities are an especially vulnerable subpopulation among all PD patients, as these patients often have worse prognosis, reduced quality of life, and increased medical costs^5–7^.

Previous research has indicated that T2DM, characterized as insulin resistance that results in impairment of glucose regulation and metabolism, may contribute to the onset of neurodegenerative diseases, such as PD, and also influence the progression of these conditions^8,9^. Research points toward dysregulation of shared pathophysiologic pathways between T2DM and PD, including insulin resistance, mitochondrial dysfunction, inflammation, or metabolic dysregulation^9,10^. Few epidemiologic studies, however, have analyzed metabolic profiles associated with T2DM among PD patients to help identify physiologic responses that are similar or different in those suffering from both medical conditions. To date, no published study has investigated metabolic features associated with T2DM in PD patients using untargeted metabolomics.

High-resolution metabolomics (HRM)^11^ has emerged as a useful tool that can profile thousands of small molecules produced from metabolism (metabolites) in different biospecimens. Previous research has described metabolomic profiles in PD^12–14^, including our own^15^, and T2DM^16^ separately. Two previous studies including both targeted and untargeted metabolomics found that lower levels of low-density lipoprotein cholesterol, higher level of fibrinogen, and lipid metabolic dysregulation were associated with a higher risk of dementia in PD patients with T2DM than without T2DM^17,18^. The current study builds on this previous work, specifically assessing the metabolic profile of T2DM among PD patients. We applied untargeted HRM to serum samples of 636 PD patients of whom 96 also suffered from T2DM and 253 older adults without PD, among whom 36 were diagnosed with T2DM. The goal of the study is to describe shared and unique metabolic features in PD patients with and without T2DM and shed light on unique dysregulated metabolic pathways in PD with T2DM. Given the aging of populations and the projected increase of prevalence in both PD^19^ and T2DM^20^, understanding the links between PD and T2DM is a public health priority^21,22^.

## Results

### Study population

As shown in Table 1, 15% of the 636 PD patients had T2DM and the mean age of PD diagnosis was similar in PD patients with T2DM (68 y, SD = ±10 y) and without T2DM (67 y, SD = ±10 y). PD patients with and without T2DM were also similar in terms of the percentages of men, people with ≥12 years of education, and have never smoked. However, more of the PD patients with T2DM reported non-European ancestry than those without T2DM. Race and ethnicity of the study population is shown in Supplementary Table 1. In total, 253 non-PD participants were included in the analysis, with 36 (14%) having T2DM. The patterns of age, lifestyle, and ethnicity were also similar to T2DM (see Table 1).

**Table 1:** Parkinson’s disease (PD) patients’ and non-PD participants’ characteristics by diabetes among those with metabolomics data

Characteristic	Statistics	PD Patients (*N* = 636)		Non-PD participants (*N* = 253)	
		Diabetes (*N* = 96)	No diabetes (*N* = 540)	Diabetes (*N* = 36)	No diabetes (*N* = 217)
Age^a^	Mean (SD)	68.03 (9.83)	66.62 (10.44)	68.92 (11.47)	65.39 (12.93)
Male gender	*n* (%)	65 (68%)	341 (63%)	22 (61%)	97 (45%)
Years of education ≥12 years^b^	*n* (%)	75 (78%)	455 (84%)	30 (83%)	195 (90%)
Past or current smoker^b^	*n* (%)	45 (47%)	252 (47%)	17 (47%)	117 (54%)
Non-European ancestry^b^	*n* (%)	40 (42%)	117 (22%)	11 (31%)	38 (18%)
Study wave					
PEG1	*n* (%)	33 (34%)	248 (46%)	27 (75%)	163 (75%)
PEG2	*n* (%)	63 (66%)	292 (54%)	9 (25%)	54 (25%)

^a^Age refers to age of PD diagnosis for PD patient group and age of enrollment in the study for non-PD participants.

^b^7 PD patients with imputed education, 5 non-PD participants with imputed education, and 4 non-PD participants with imputed smoking and ethnicity data.

### Metabolome-wide analysis and annotation

**Fig. 1:**
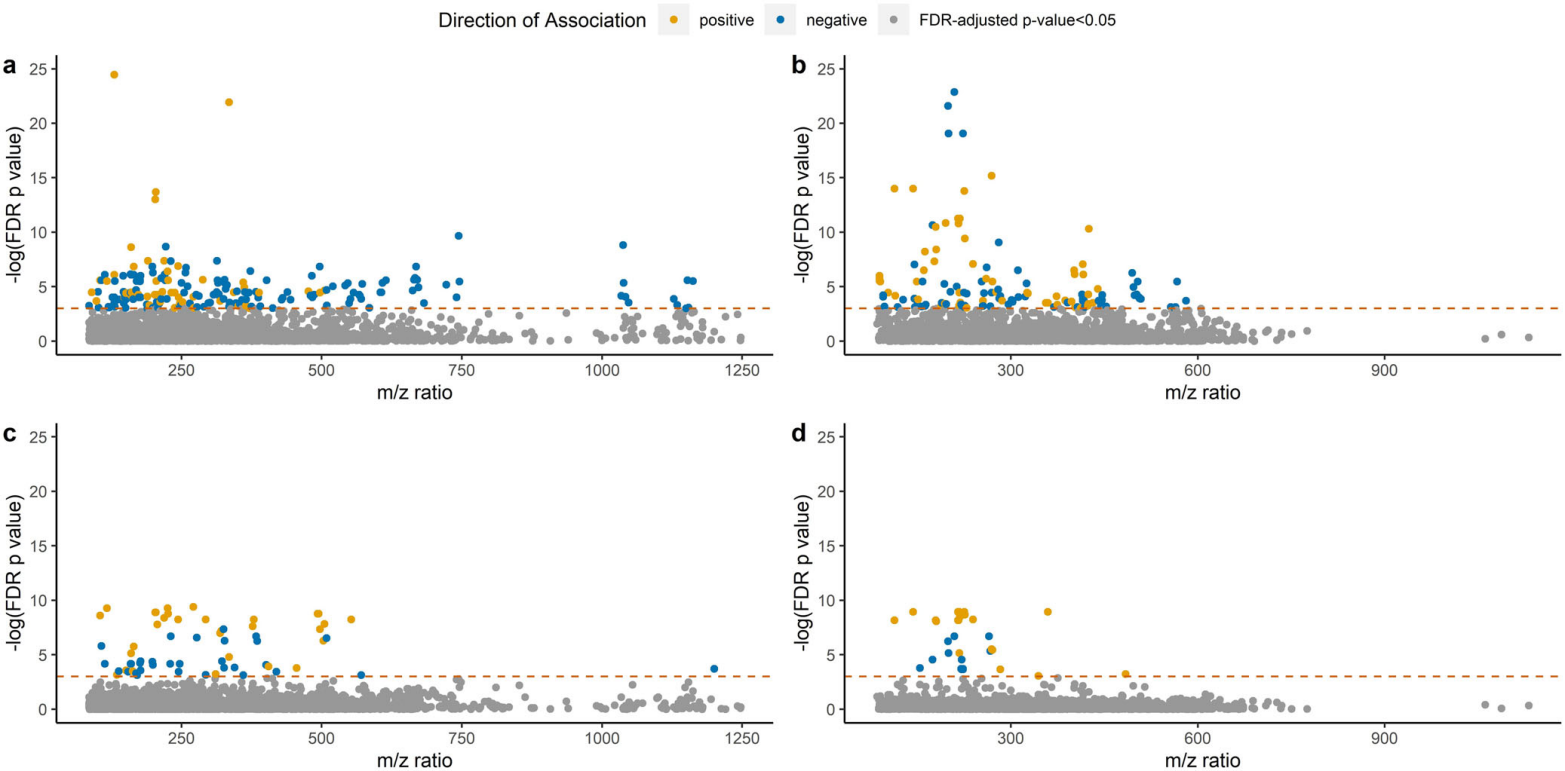
Manhattan plots of results of metabolome-wide association study for diabetes mellitus (DM). **a** Among Parkinson’s disease (PD) patients in HILIC column, **b** among PD patients in C18 column, **c** among non-PD participants in HILIC column, and **d** among non-PD participants in C18 column.

**Fig. 2:**
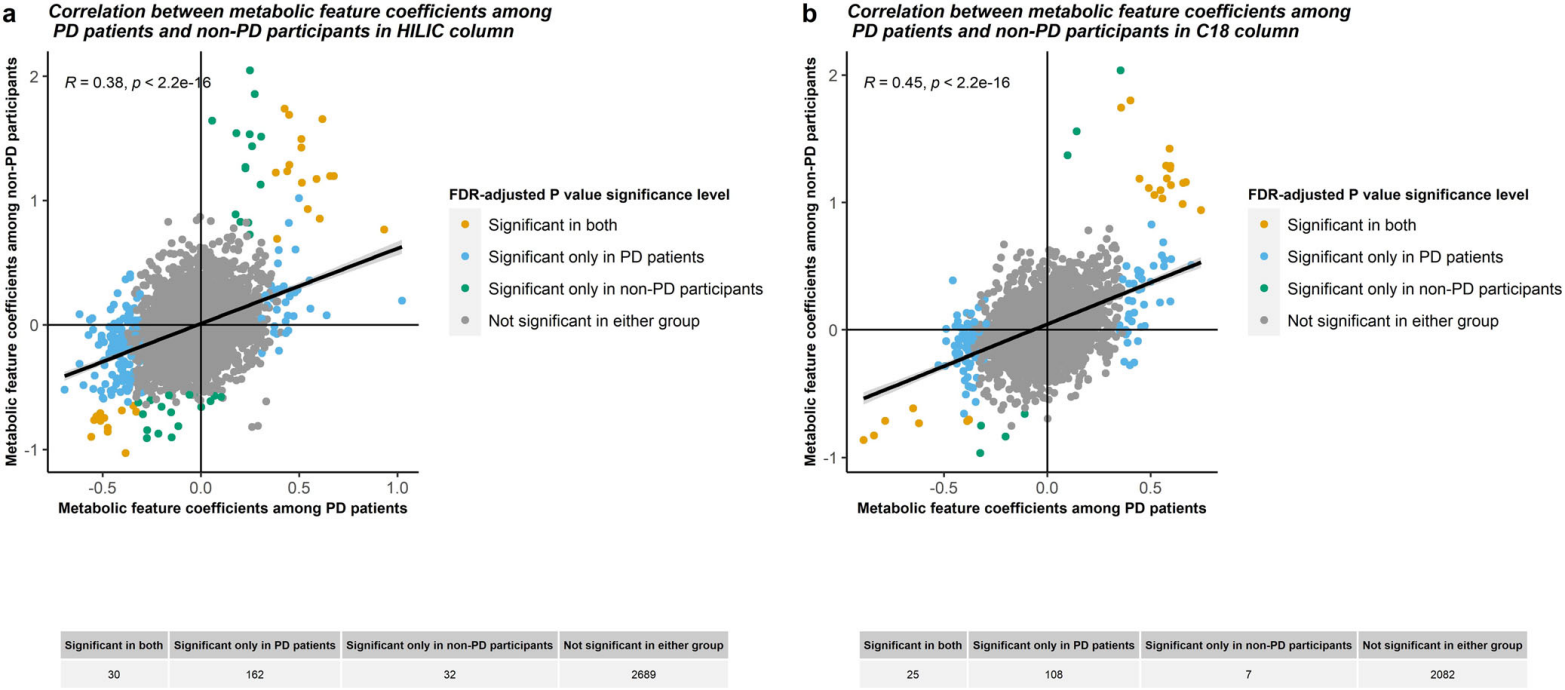
Correlations between coefficients of metabolic features from metabolome-wide association study for diabetes mellitus (DM) among Parkinson’s disease (PD) patients and non-PD participants. **a** HILIC column and **b** C18 column colored by significance level adjusting for multiple testing.

For untargeted, high-resolution metabolomics, we analyzed serum samples using both hydrophilic interactions (HILIC) chromatography with positive electrospray ionization (ESI) and C18 chromatography with negative ESI. After feature alignment and quality control, we included 2913 metabolic features detected in the positive ion mode (HILIC) and 2222 metabolic features from the negative ion mode (C18) in our MWAS analysis. MWAS results are shown in Figs. 1 and 2 and complete summary statistics for the metabolic features associated with T2DM are available through figshare (10.6084/m9.figshare.22589464). After adjusting for multiple testing and considering a significance level of FDR < 0.05, 192 metabolic features from the HILIC column and 133 in the C18 column were associated with T2DM among the PD patients. When considering only replication of the T2DM MWAS metabolites discovered in this PD population, after multiple testing correction, 49 of the 192 metabolic features associated in HILIC and 38 out of 133 metabolic features in C18 were also associated with T2DM at a replication FDR < 0.05 in the non-PD participants (Supplementary Table 2). The coefficients for T2DM association across metabolic features from the MWAS among the PD patient group and non-PD participant group were moderately correlated (HILIC: r = 0.4, *p* < 2.2e−16; C18: r = 0.45, *p* < 2.2e−16). This is displayed in Fig. 2, which plots metabolite feature coefficients from the T2DM MWAS in PD and non-PD participants, colored features by association status (i.e., associated with T2DM in PD patient group, non-PD participant group or both). Most features showed concordance in the direction of association with T2DM among both the PD and non-PD participants.

Annotations for all metabolic features using the in-house library and xMSannotator are included in Supplementary Tables 3 and 4. Among the T2DM-associated metabolic features, 9 metabolites were uniquely annotated at high confidence, while 9 features were annotated at high confidence to multiple metabolites based on both the in-house library and xMSannotator (see Table 2). For example, the top feature (*mz* = 215.0328, retention time = 30.913) associated with T2DM among PD patients (OR = 1.80 per SD, 95% CI = 1.46, 2.25, FDR = 1.30e−05) and non-PD patients (OR = 3.62, 95% CI = 2.22, 5.91, FDR = 1.33e−04) was annotated at high confidence to glucose. Similarly, another top feature (*mz* = 179.0562, retention time = 36.803) in both groups (PD patients, OR = 1.75, 95% CI = 1.41, 2.15, FDR = 2.81e−05; non-PD participants, OR = 2.80, 95% CI = 1.84, 4.26, FDR = 2.83e−04) was annotated to simple sugars (i.e., glucose, fructose and mannose). The amino acid metabolite citrulline (*mz* = 174.088, rt = 30.043) was inversely associated with T2DM in both PD patients (OR = 0.54, 95% CI = 0.43, 0.68, FDR = 2.35e−05) and non-PD participants (OR = 0.48, 95% CI = 0.33, 0.70, FDR = 1.07e−02).

**Table 2:** Annotation of metabolic features associated with diabetes mellitus among Parkinson’s disease (PD) patients and non-PD participants

						PD patients			Non-PD participants		
Chemical name	Library	Indicator of Match	Observed mass to charge ratio	Observed retention time	Column	OR [95%CI]	*p* value	FDR *p* value	OR [95%CI]	*p* value	FDR *p* value
D-Galactose/D-Glucose	xMSannotator	Multiple	215.033	30.913	C18	1.81 [1.46, 2.25]	0.000	0.000	3.62 [2.22, 5.91]	0.000	0.000
Citrulline	in-house	Unique	174.088	30.043	C18	0.54 [0.43, 0.68]	0.000	0.000	0.48 [0.33, 0.7]	0.000	0.011
	In-house	Unique	176.103	102.372	HILIC	0.61 [0.48, 0.78]	0.000	0.004	0.47 [0.32, 0.7]	0.000	0.014
myo-Inositol/Allose/Sorbitol/D-Fructose/L-Sorbose/Alpha-D-Glucose/D-Galactose/D-Tagatose/D-Mannose	In-house	Multiple	179.056	36.803	C18	1.75 [1.41, 2.15]	0.000	0.000	2.8 [1.84, 4.26]	0.000	0.000
L-Gulonolactone	In-house	Unique	177.040	33.332	C18	1.72 [1.36, 2.19]	0.000	0.001	1.24 [0.84, 1.83]	0.283	0.786
Argininic acid/Citrulline	xMSannotator	Multiple	198.085	100.113	HILIC	0.58 [0.46, 0.73]	0.000	0.001	0.47 [0.31, 0.69]	0.000	0.013
1-(Hydroxymethyl)-55-dimethyl-24-imidazolidinedione	xMSannotator	Unique	159.076	102.224	HILIC	0.59 [0.46, 0.75]	0.000	0.002	0.48 [0.33, 0.71]	0.000	0.015
Glyceraldehyde/L-Lactic acid	In-house	Multiple	89.024	31.295	C18	1.57 [1.26, 1.95]	0.000	0.003	1.57 [1.09, 2.27]	0.016	0.326
Indoleacetaldehyde	In-house	Unique	158.061	87.220	C18	0.61 [0.48, 0.78]	0.000	0.004	0.76 [0.52, 1.11]	0.150	0.650
Sarcosine/L-Alanine/D-Alanine/beta-Alanine	In-house	Multiple	90.055	84.601	HILIC	1.53 [1.21, 1.92]	0.000	0.011	1.36 [0.93, 2]	0.114	0.533
2-(Methylthio)methyl-2-butenal/2-Ethyldihydro-3(2H)-thiophenone/S-2-Propenyl propanethioate (R)-3-Hydroxybutyric acid/Alpha-Hydroxyisobutyric acid/2-Hydroxybutyric acid	In-house	Multiple	88.040	36.442	C18	1.3 [1.05, 1.6]	0.017	0.142	1.18 [0.81, 1.71]	0.397	0.869
	In-house	Multiple	103.040	32.211	C18	1.49 [1.2, 1.84]	0.000	0.012	1.44 [1, 2.06]	0.049	0.452
2-(Methylthio)methyl-2-butenal/2-Ethyldihydro-3(2H)-thiophenone/S-2-Propenyl propanethioate	xMSannotator	Multiple	148.080	94.797	HILIC	0.66 [0.53, 0.83]	0.000	0.012	0.83 [0.6, 1.16]	0.280	0.744
1-Pyrroline-4-hydroxy-2-carboxylate/5-Oxoprolinate/dimethadione/N-Acryloylglycine/Pyroglutamic acid/Pyrrolidonecarboxylic acid/Pyrroline hydroxycarboxylic acid	xMSannotator	Multiple	131.053	97.331	HILIC	0.7 [0.56, 0.88]	0.003	0.042	0.84 [0.6, 1.16]	0.289	0.751
L-Proline	In-house	Unique	114.056	36.877	C18	1.48 [1.19, 1.84]	0.000	0.016	1.09 [0.75, 1.59]	0.646	0.957
1-Pyrroline-4-hydroxy-2-carboxylate/5-Oxoprolinate/N-Acryloylglycine/Pyroglutamic acid/Pyrrolidonecarboxylic acid/Pyrroline hydroxycarboxylic acid/dimethadione	xMSannotator	Multiple	130.050	94.746	HILIC	0.67 [0.53, 0.85]	0.001	0.018	0.93 [0.66, 1.3]	0.666	0.918
L-Glutamic acid	In-house	Unique	148.060	89.479	HILIC	1.47 [1.18, 1.84]	0.001	0.018	0.93 [0.64, 1.35]	0.697	0.932
Biliverdin (S)-N-(45-Dihydro-1-methyl-4-oxo-1H-imidazol-2-yl)alanine	In-house	Unique	581.240	42.898	C18	0.71 [0.58, 0.87]	0.001	0.024	0.86 [0.58, 1.28]	0.471	0.909
	xMSannotator	Unique	220.049	30.642	C18	0.68 [0.53, 0.86]	0.002	0.034	0.49 [0.33, 0.72]	0.000	0.024
6-Chloro-N-(1-methylethyl)-135-triazine-24-diamine/Indoleacrylic acid	xMSannotator	Multiple	188.071	59.806	HILIC	0.68 [0.54, 0.87]	0.002	0.034	0.86 [0.58, 1.26]	0.442	0.828
3-Methyldioxyindole	In-house	Unique	162.056	40.608	C18	0.67 [0.53, 0.87]	0.002	0.040	0.87 [0.6, 1.27]	0.477	0.911
Mannitol/Galactitol	In-house	Multiple	181.071	37.306	C18	1.38 [1.12, 1.72]	0.003	0.050	1.3 [0.91, 1.86]	0.147	0.646
()-Tryptophan/3-Hydroxymethylantipyrine/4-Hydroxyantipyrine/D-Tryptophan/Ethotoin/L-Tryptophan/Nirvanol/S-nirvanol	xMSannotator	Multiple	205.097	59.833	HILIC	0.7 [0.55, 0.89]	0.003	0.051	0.84 [0.57, 1.24]	0.377	0.803
()-Tryptophan/3-Hydroxymethylantipyrine/4-Hydroxyantipyrine/D-Tryptophan/Ethotoin/L-Tryptophan/Nirvanol/S-nirvanol 3-(Carboxymethylamino)propanoic acid/D-Glutamic acid/DL-Glutamate/L-4-Hydroxyglutamate semialdehyde/L-Glutamic acid/N-Acetylserine/N-Methyl-D-aspartic acid/O-Acetylserine	xMSannotator	Multiple	249.061	60.032	HILIC	0.72 [0.57, 0.91]	0.005	0.067	0.76 [0.52, 1.11]	0.151	0.583
	xMSannotator	Multiple	192.024	88.823	HILIC	1.38 [1.1, 1.73]	0.006	0.070	1.47 [0.99, 2.18]	0.055	0.386
Uridine Dihydrothymine/L-Cyclo(alanylglycyl)/Squamolone	In-house	Unique	243.062	33.439	C18	1.38 [1.09, 1.73]	0.006	0.075	1.04 [0.71, 1.51]	0.858	0.989
	xMSannotator	Multiple	129.066	86.264	HILIC	0.69 [0.53, 0.91]	0.008	0.088	0.6 [0.38, 0.96]	0.033	0.309
L-Fucose/Rhamnose	xMSannotator	Multiple	173.030	86.515	HILIC	0.75 [0.57, 0.98]	0.032	0.206	0.59 [0.37, 0.92]	0.021	0.259
L-Fucose/Rhamnose	In-house	Multiple	163.061	33.325	C18	0.75 [0.61, 0.93]	0.008	0.088	0.59 [0.42, 0.84]	0.003	0.126
Hypoxanthine	In-house	Unique	137.046	56.412	HILIC	0.76 [0.61, 0.94]	0.013	0.118	1.17 [0.78, 1.75]	0.437	0.827
L-Aspartic acid/D-Aspartic acid	In-house	Multiple	134.045	102.083	HILIC	1.36 [1.06, 1.74]	0.015	0.129	0.98 [0.68, 1.41]	0.904	0.985
L-Serine	In-house	Unique	104.035	30.101	C18	0.75 [0.59, 0.94]	0.015	0.132	0.62 [0.42, 0.93]	0.020	0.374
L-Cystine	xMSannotator	Unique	241.031	231.655	HILIC	1.3 [1.05, 1.63]	0.019	0.146	1.1 [0.72, 1.67]	0.660	0.918
Oxoglutaric acid	In-house	Unique	145.014	28.164	C18	1.3 [1.04, 1.63]	0.022	0.166	1.31 [0.9, 1.9]	0.160	0.665
trans-Aconitic acid	In-house	Unique	173.009	29.367	C18	1.33 [1.04, 1.7]	0.022	0.166	1.06 [0.71, 1.57]	0.775	0.989
Pantothenic acid	In-house	Unique	220.118	41.476	HILIC	1.31 [1.04, 1.64]	0.023	0.167	1.12 [0.77, 1.63]	0.558	0.876
L-Threonine/L-Homoserine/L-Allothreonine	In-house	Multiple	120.066	87.232	HILIC	0.77 [0.61, 0.97]	0.027	0.183	0.89 [0.61, 1.3]	0.550	0.874
gamma-Aminobutyric acid/2-Aminoisobutyric acid/3-Aminoisobutanoic acid	In-house	Multiple	102.056	29.529	C18	1.28 [1.02, 1.61]	0.031	0.202	1.1 [0.76, 1.59]	0.623	0.952
Mevalonic acid	In-house	Unique	147.066	27.018	C18	1.27 [1.02, 1.59]	0.033	0.211	1.07 [0.75, 1.52]	0.714	0.978
3-Mercapto-2-methyl-1-butanol/3-Mercapto-3-methyl-1-butanol/4-(Methylthio)-1-butanol/4-(Methylthio)-2-butanol/xi-2-Mercapto-3-methyl-1-butanol	xMSannotator	Multiple	121.069	87.491	HILIC	0.79 [0.63, 0.99]	0.043	0.243	0.62 [0.42, 0.92]	0.018	0.246
L-Cystathionine	In-house	Unique	223.075	221.449	HILIC	1.28 [1.01, 1.63]	0.045	0.247	1.18 [0.79, 1.75]	0.413	0.821
1-(2-Thienyl)-1-heptanone	xMSannotator	Unique	219.083	108.587	HILIC	0.79 [0.62, 1]	0.046	0.252	1.12 [0.76, 1.65]	0.566	0.878
N-Acetylneuraminic acid	In-house	Unique	308.098	30.995	C18	1.23 [1, 1.51]	0.050	0.273	1.46 [1.02, 2.09]	0.038	0.436
(S)-N-(45-Dihydro-1-methyl-4-oxo-1H-imidazol-2-yl)alanine	xMSannotator	Unique	184.073	35.977	C18	0.8 [0.63, 1.01]	0.065	0.313	0.54 [0.37, 0.78]	0.001	0.079
L-Histidine	In-house	Unique	154.062	38.677	C18	0.84 [0.67, 1.06]	0.144	0.475	0.47 [0.29, 0.76]	0.002	0.105
246-Octatriyn-1-ol/357-Octatriyn-1-ol/Benzofuran/xi-23-Octadiene-57-diyn-1-ol	xMSannotator	Multiple	119.049	71.599	HILIC	0.86 [0.68, 1.08]	0.194	0.542	0.67 [0.46, 0.99]	0.042	0.343
Phenylpyruvic acid	In-house	Unique	165.055	72.205	HILIC	0.87 [0.69, 1.09]	0.228	0.581	0.67 [0.45, 0.98]	0.037	0.327
Stearic acid	In-house	Unique	283.264	264.643	C18	1.14 [0.91, 1.43]	0.241	0.608	1.55 [1.07, 2.25]	0.020	0.374
D-Arabitol/D-Xylitol/L-2-(Hydroxymethyl)-1234-butanetetrol/L-Arabitol/Ribitol	xMSannotator	Multiple	197.041	89.659	HILIC	0.89 [0.71, 1.12]	0.324	0.682	0.68 [0.48, 0.97]	0.033	0.309
	xMSannotator	Multiple	153.077	90.009	HILIC	1.03 [0.82, 1.29]	0.796	0.930	0.62 [0.42, 0.9]	0.011	0.202
O-Phosphoethanolamine	In-house	Unique	140.011	27.424	C18	0.91 [0.74, 1.11]	0.360	0.702	0.66 [0.47, 0.91]	0.012	0.299
Petroselinic acid/Oleic acid/Elaidic acid	In-house	Multiple	281.248	242.644	C18	1.11 [0.87, 1.4]	0.397	0.713	1.5 [1.01, 2.23]	0.044	0.440
Benzylamine	In-house	Unique	108.081	30.716	HILIC	0.94 [0.75, 1.18]	0.581	0.849	0.61 [0.39, 0.94]	0.024	0.274

Most were associated with T2DM only among PD patients, though many showed similar trends among the non-PD participants. Metabolites with unique annotation matches included several amino acids (cystathionine, cystine, glutamic acid, gulonolactone, proline, aspartic acid, and indoleacetaldehyde), nucleic acids (uridine, hypoxanthine), and tricarboxylic acid (TCA) cycle/coenzyme A (CoA) related metabolites (oxoglutaric acid, pantothenic acid, trans-aconitic acid, and mevalonic acid).

### Pathway enrichment analysis

**Fig. 3:**
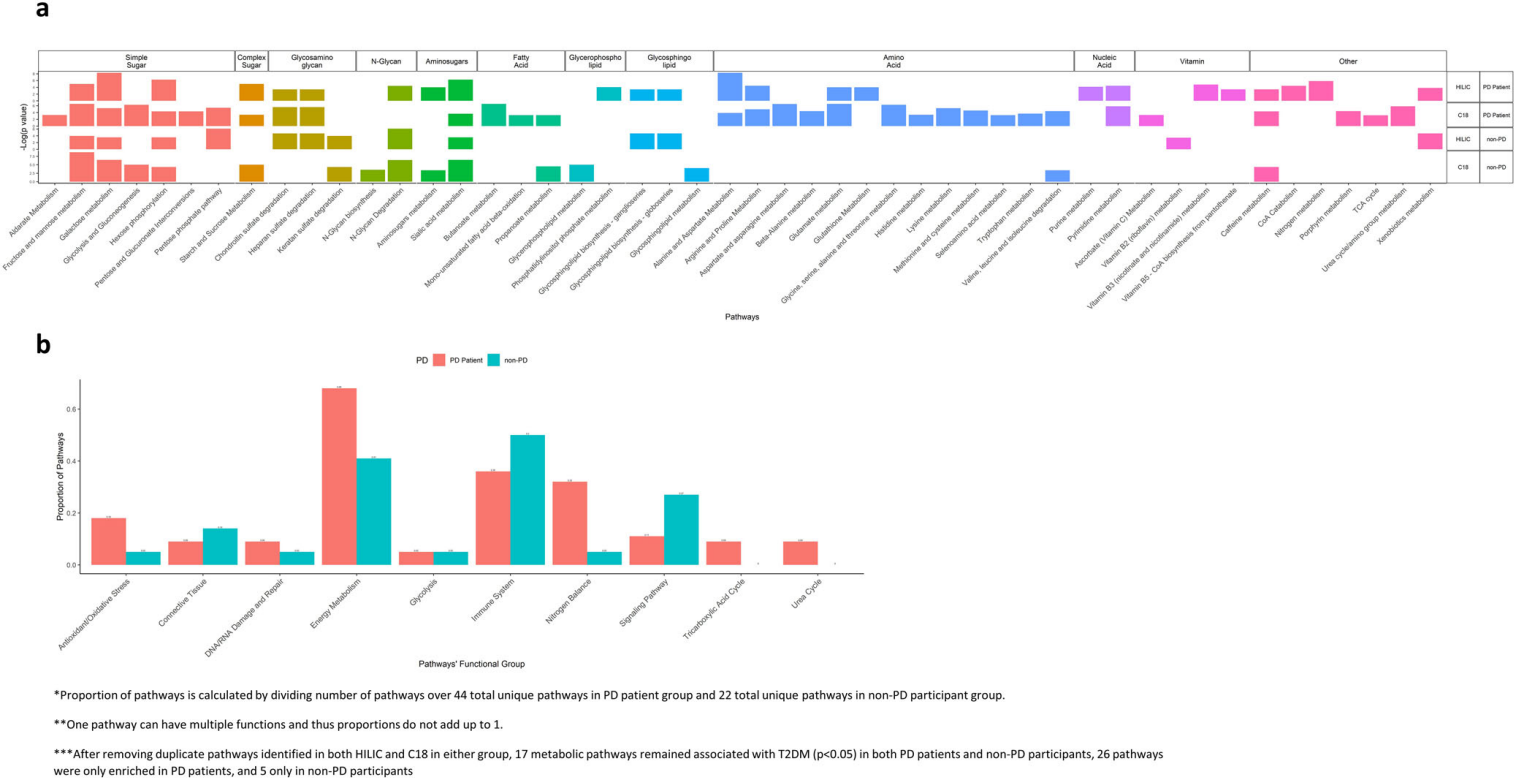
Pathway enriched among Parkinson’s disease (PD) patients and non-PD participants. **a** Annotation of pathways by chemical groups, **b** by functional proporties.

We identified 55 metabolic pathways enriched (*p* < 0.05) among the features associated with T2DM in PD patients and 28 metabolic pathways from the features associated with T2DM among non-PD participants (see Table 3). After removing duplicate pathways identified in both HILIC and C18 in either group, 17 metabolic pathways remained associated with T2DM (*p* < 0.05) in both PD patients and non-PD participants, 26 pathways were only enriched in PD patients, and 5 only in non-PD participants. Figure 3 shows these pathways, along with each pathway’s biochemical classification and functional group.

**Table 3:** Metabolic pathways associated with diabetes mellitus (DM) using metabolites captured by positive ion mode (HILIC) and negative ion mode (C18) among Parkinson’s disease (PD) patients and non-PD participants

pathway	Column	PD Patients			Non-PD participants		
		Overlap size	Pathway size	*p*-value	Overlap size	Pathway size	*p*-value
*Metabolic pathways associated with DM only among PD patients*							
Alanine and aspartate metabolism	C18	6	10	0.0211	2	10	0.2405
	HILIC	6	7	0.0003	1	7	0.2601
Arginine and proline metabolism	C18	8	13	0.0073	3	13	0.1682
	HILIC	6	14	0.0118	1	14	0.3388
Ascorbate (Vitamin C)	C18	7	14	0.0385	0	14	0.3702
Aldarate metabolism	C18	7	14	0.0385	0	14	0.3702
Aspartate and asparagine metabolism	C18	11	17	0.0015	4	17	0.1385
Beta-Alanine metabolism	C18	5	7	0.0124	2	7	0.1519
Butanoate metabolism	C18	5	5	0.0014	2	5	0.0888
CoA Catabolism	HILIC	2	2	0.0118	0	2	0.3738
Glutamate metabolism	C18	5	5	0.0014	1	5	0.2759
	HILIC	3	5	0.0186	0	5	0.3738
Glutathione metabolism	HILIC	3	5	0.0186	0	5	0.3738
Glycine, serine, alanine and threonine metabolism	C18	10	15	0.0018	3	15	0.214
Histidine metabolism	C18	3	4	0.0367	1	4	0.2443
Lysine metabolism	C18	5	6	0.005	1	6	0.3025
Methionine and cysteine metabolism	C18	6	9	0.0102	1	9	0.3451
Mono-unsaturated fatty acid beta-oxidation	C18	2	2	0.0405	0	2	0.3702
Nitrogen metabolism	HILIC	3	3	0.0029	0	3	0.3738
Pentose and glucuronate interconversions	C18	4	5	0.0135	0	5	0.3702
Phosphatidylinositol phosphate metabolism	HILIC	4	8	0.0177	2	8	0.0858
Porphyrin metabolism	C18	5	7	0.0124	0	7	0.3702
Purine metabolism	HILIC	5	11	0.0157	2	11	0.1524
Pyrimidine metabolism	HILIC	6	14	0.0118	3	14	0.0778
	C18	9	14	0.003	4	14	0.0843
Selenoamino acid metabolism	C18	2	2	0.0405	1	2	0.1441
TCA cycle	C18	5	9	0.0426	0	9	0.3702
Tryptophan metabolism	C18	9	18	0.0255	3	18	0.2708
Urea cycle/amino group metabolism	C18	12	21	0.0029	3	21	0.3142
Vitamin B3 (nicotinate and nicotinamide) metabolism	HILIC	4	7	0.0082	0	7	0.3738
Vitamin B5 - CoA biosynthesis from pantothenate	HILIC	2	3	0.0339	0	3	0.3738
*Metabolic pathways associated with DM only among non-PD participants*							
Glycerophospholipid metabolism	C18	5	18	0.368	7	18	0.0066
Glycosphingolipid metabolism	C18	3	9	0.2925	4	9	0.0177
Keratan sulfate degradation	C18	3	5	0.0722	3	5	0.0129
	HILIC	2	4	0.0623	2	4	0.019
N-Glycan biosynthesis	C18	2	3	0.112	2	3	0.0291
Vitamin B2 (riboflavin) metabolism	HILIC	0	1	0.4588	1	1	0.0329
*Metabolic pathways associated with DM among both PD patients and non-PD participants*							
Aminosugars metabolism	HILIC	3	5	0.0186	1	5	0.2012
	C18	4	7	0.055	3	7	0.034
Caffeine metabolism	C18	4	5	0.0135	3	5	0.0129
	HILIC	2	3	0.0339	1	3	0.1257
Chondroitin sulfate degradation	HILIC	2	3	0.0339	2	3	0.0093
	C18	4	4	0.0038	1	4	0.2443
Fructose and mannose metabolism	C18	5	5	0.0014	5	5	0.0002
	HILIC	3	4	0.0066	2	4	0.019
Galactose metabolism	C18	6	8	0.005	5	8	0.0016
	HILIC	5	5	0.0003	2	5	0.0297
Glycolysis and gluconeogenesis	C18	6	7	0.0018	4	7	0.0066
Glycosphingolipid biosynthesis—ganglioseries	HILIC	2	3	0.0339	2	3	0.0093
Glycosphingolipid biosynthesis—globoseries	HILIC	2	3	0.0339	2	3	0.0093
Heparan sulfate degradation	HILIC	2	3	0.0339	2	3	0.0093
	C18	4	4	0.0038	1	4	0.2443
Hexose phosphorylation	C18	4	5	0.0135	3	5	0.0129
	HILIC	4	5	0.0019	2	5	0.0297
N-Glycan degradation	HILIC	2	2	0.0118	2	2	0.0023
	C18	2	3	0.112	3	3	0.0017
Pentose phosphate pathway	HILIC	1	2	0.1668	2	2	0.0023
	C18	7	10	0.0046	3	10	0.0993
Propanoate metabolism	C18	2	2	0.0405	2	2	0.011
Sialic acid metabolism	C18	5	8	0.0268	5	8	0.0016
	HILIC	4	5	0.0019	2	5	0.0297
Starch and sucrose metabolism	C18	3	4	0.0367	3	4	0.0066
	HILIC	3	4	0.0066	1	4	0.1665
Valine, leucine and isoleucine degradation	C18	5	7	0.0124	3	7	0.034
Xenobiotics metabolism	HILIC	5	12	0.0219	4	12	0.0093

The pathways enriched for T2DM-associated metabolites in both PD and non-PD participants included carbohydrate-associated metabolic pathways (e.g., simple sugar metabolism, such as fructose, mannose, galactose metabolism and glycolysis, and complex sugar metabolism, such as starch and sucrose metabolism, n-glycan pathway, amino sugars metabolism) and glycosphingolipid metabolism.

The T2DM-associated pathways enriched only among PD patients included multiple amino acid metabolism pathways (e.g., alanine, aspartate, arginine, proline, glutamate, and glutathione metabolism), nucleic acid metabolism pathways (e.g., purine metabolism and pyrimidine metabolism), and fatty acid metabolisms (e.g., butanoate metabolism and mono-unsaturated fatty acid beta-oxidation). In addition, the T2DM-associated metabolites from PD patients were also enriched for urea cycle and TCA cycle-related pathways, including CoA catabolism and vitamin B5 - CoA biosynthesis from pantothenate.

Full pathway analysis results are included in Supplementary Table 5. The pathway classifications and supporting references are provided in Supplementary Table 6. In addition, when we used a significance level of 0.15 for pathway enrichment analysis among non-PD participants, the T2DM-associated metabolic pathways were similar to the pathways identified using a significance level of 0.05 among non-PD participants (See Supplementary Table 7).

### Power analysis

Based on power analysis for replication of the T2DM metabolite associations discovered among the PD patients in the non-PD participants, we were powered to detect betas >0.81 and <−0.82 [power = 0.8, alpha = 0.05/192 of tests (HILIC), sample size of 253, event rate of 0.14] and betas >0.80 and <−0.80 [power = 0.8, alpha = 0.05/133 of tests (C18)]. For a full, untargeted MWAS among the non-PD participants with logistic regression, we were powered to detect betas >0.93 and <−0.94 [power = 0.8, alpha = 0.05/2931 of tests (HILIC)] and betas >0.92 and <−0.92 [power = 0.8, alpha = 0.05/2222 of tests (C18)]. Additionally, for the pathway analysis among the T2DM-associated metabolites from non-PD controls, we assessed enrichment with more relaxed significance thresholds from the MWAS for metabolite inclusion. This included an unadjusted p = 0.15, which we were powered to detect betas >0.41 and <−0.42 (power = 0.8, alpha = 0.15) and unadjusted *p* = 0.05, which we were powered to detect betas >0.51 and <−0.51 (power = 0.8, alpha=0.05).

### Comparison between T2DM and PD MWAS

We have previously published the findings from a PD MWAS using the same population^15^. Overall, there were 13 metabolites identified as associated with T2DM among the PD patients and PD (Supplementary Table 8). These metabolites include indoleacetaldehyde, glutamic acid, indoleacrylic acid, 3-methyldioxyindole, tryptophan, glutamate, uridine, serine, oxoglutaric acid, threonine, aminobutyric acid, squamolone, and 3-mercapto-3-methylbutan-1-ol.

### Sensitivity analyses

The results from the T2MD MWAS among the PD patients stratified by gender (Supplementary Table 9) and European Ancestry (Supplementary Table 10) can be found in the supplement. Beta coefficients were highly correlated between those found among the men and the primary MWAS (r = 0.91 and 0.93, *p* < 2.2e−16 for HILIC and C18) and somewhat less, though still correlated, comparing the women and the primary MWAS (r = 0.63 and 0.67, *p* < 2.2e−16 for HILIC and C18), which shown in Supplementary Fig. 1. Beta coefficients were also highly correlated between the European ancestry only group and the primary MWAS (r = 0.91 and 0.93, *p* < 2.2e−16 for HILIC and C18) and between the non-European ancestry group and our main analysis (r = 0.74 and 0.75, *p* < 2.2e−16 for HILIC and C18), which shown in Supplementary Fig. 2.

The results of 3-fold cross-validation can be found in Supplementary Table 11. The significance rates for the validation are the number of significantly replicated metabolic features in the replicate sample divided by the total number of significant metabolic features in the discovery sample for each fold. For the T2DM MWAS among PD patients, the significance rates were 49%, 50%, and 70% in HILIC with the correlation of the betas between discovery and the replication fold at 0.88, 0.85, and 0.92 (Supplementary Fig. 3), and 25%, 77%, and 58% in C18 with beta correlations at 0.69, 0.98, and 0.94 (Supplementary Fig. 3). For the T2DM MWAS among non-PD participants, the significance rates and beta correlations were 95%, 75%, and 96% and 0.93, 0.88, and 0.88 (Supplementary Fig. 4) for HILIC metabolites, and 85%, 48%, and 100% and 0.98, 0.70, and 0.87 (Supplementary Fig. 4) for C18 metabolites.

Further sensitivity analyses, additionally controlling for levodopa equivalent daily dose (Supplementary Table 12 and Supplementary Fig. 5) and sample year (Supplementary Tables 13, 14 and Supplementary Figs. 6 and 7) showed very similar results (MWAS beta correlations >0.99). Similarly, considering T2DM with T2DM medication use as the exposure resulted in very similar results (MWAS beta correlations >0.96, Supplementary Table 15 and Supplementary Figs. 8 and 9).

## Discussion

Comorbidities including type 2 diabetes mellitus (T2DM) can significantly impact the prognosis of PD patients and increase their financial burden^5^. Recent studies have suggested that T2DM contributes to faster motor progression and more severe cognitive impairment among PD patients^23–26^. However, the underlying mechanisms are unknown^27^. To better understand the influence of T2DM on PD physiology, we used untargeted HRM to profile thousands of metabolites in the serum of PD patients and non-PD elderly participants with and without T2DM.

Our results suggest that—as one would expect—some of the most dysregulated T2DM-related metabolic pathways are shared by PD and non-PD study participants, confirming the impact of T2DM on the human metabolome independent of PD. Even though the number of statistically significant metabolic features was small, the correlation of the coefficients across metabolic features was somewhat high (R > 0.4) (see Fig. 2). These metabolites were enriched in sixteen pathways of high biologic plausibility for T2DM, that is, one would expect to find them enriched in T2DM, such as carbohydrate metabolism, glycosaminoglycans, glycan metabolism, and amino sugars metabolism, as well as glycosphingolipid metabolism, the latter has been linked to insulin resistance^28^.

In terms of individual metabolites, we observed many expected associations, such as glucose and simple sugars, along with several interesting associations, including biliverdin, which was negatively associated with T2DM among PD patients. Biliverdin is derived from the breakdown of heme, which is then generally quickly broken down to bilirubin by biliverdin reductase^29^. Interestingly, both a buildup of and reduction in biliverdin have been associated with disease^29^. For instance, biliverdin has been shown to have anti-inflammatory effects and be protective against insulin resistance^30^. However, liver failure and impaired metabolism also result in elevated biliverdin^29^.

We also observed several pathways that were altered with T2DM among PD patients. These include essential amino acid pathways such as histidine metabolism, lysine metabolism, and tryptophan metabolism. These amino acids are important for many physiologic functions, including energy production and nitrogen balance, which are essential for growth, development, and tissue repair^31,32^, as well as regulating neurological function, gene expression, cell signaling, antioxidative responses, and immunity^33^. Essential amino acids have also been linked to mitochondrial dysfunction via disrupted energy production and oxidative stress^34^. Mitochondrial dysfunction is strongly linked to the pathogenesis of both T2DM^35^ and PD^36^. Tryptophan is also metabolized into indole derivatives, including 3-methyldioxyindole, indoleacetic acid, and indoleacetaldehyde via gut microbiota^37–39^. In the present study, these indole derivatives were also negatively associated with T2DM among the PD patients. Interestingly, tryptophan metabolism, indole metabolism, and metabolic disease have been linked recently through the gut microbiome^38,40,41^. The importance of the gut microbiome and gut-brain axis is also increasingly recognized in PD^42^.

There is also evidence that several non-essential amino acids, including glutamate^43^ and glycine^44^, are involved in oxidative stress pathways. Our study showed elevated levels of glutamate metabolites associated with T2DM in PD patients. Glutamate can be converted to α-ketoglutarate to participate TCA cycle^45^. However, excessive amounts of glutamate, called glutamate excitotoxicity, can lead to Ca^2+^ influx that can promote mitochondria dysfunction and cell death^46^. Furthermore, these amino acid pathways have other links to PD. For instance, through overexpression of PGC-1α, which can result in mitochondrial function and a decrease in phenylalanine, tyrosine, and glutamine^47,48^, or involvement of PINK1, which has been linked to an increased glutamate and dysregulation of the TCA cycle^49^. Oxidative stress has been suggested as a shared pathway between PD and T2DM^50^ and is closely linked to mitochondria dysfunction among other mechanisms^51^. In T2DM, oxidative stress is induced by hyperglycemia^52^. In PD, oxidative stress is thought to play an important role in the death of dopaminergic neurons^53^. It is especially interesting that nearly 20% of the pathways enriched in PD patients with T2DM are linked to oxidative stress versus <1% of the pathways enriched among non-PD participants with T2DM.

Dysregulation of the urea cycle and nitrogen metabolism was also linked to T2DM among PD patients. Increased levels of urea in the kidneys are associated with insulin resistance and suppression of insulin, with evidence from both experimental and epidemiological studies^54,55^. Elevated urea levels were also found in the brains of PD patients in a brain tissue study^56^. Urea accumulated in the blood can slowly cross the blood-brain barrier as it becomes compromised with PD, resulting in elevated levels of urea in the brain^57,58^. In addition, previous metabolomics studies also identified urea metabolism associated with PD and T2DM independently^59,60^.

Several other pathways with compelling links to both PD and T2DM were also implicated by our results. Both pyrimidine and purine metabolism were enriched in the T2DM-associated metabolites of PD patients. Purines have roles in energy metabolism and signaling, and, along with pyrimidines, DNA and RNA production. A previous metabolomics study identified altered purine metabolism with T2DM^61^ and mouse models have linked purine metabolites to faster T2DM progression^62^. Similarly, purine metabolism has also been linked to faster PD motor progression in epidemiological studies and DNA damage has been implicated as contributing to PD pathogenesis by epidemiological and experimental studies^63,64^. Two fatty acid metabolism pathways, butanoate metabolism, and mono-unsaturated fatty acid β oxidation were also associated with T2DM among PD patients. Higher levels of fatty acid metabolites can impact the severity of insulin resistance^65,66^. Fatty acid oxidation has been linked to PD onset and mild cognitive impairment in a previous metabolic study of PD patients^67^. In addition, β oxidation has been suggested as a potential biomarker for the diagnosis of PD at an early stage^68^. Butanoate or butyrate metabolism refers to short-chain fatty acid metabolism produced by the bacterial formation in the human colon and butyrate is important for maintaining energy balance^69^. A previous metabolic profiling of T2DM patients also showed an elevated level of butanoate^70^ and a meta-analysis of gut microbiome-focused studies found bacterial butanoate metabolism to be upregulated among PD patients^71^. Our results suggest that PD patients with T2DM have levels of these important PD-linked metabolites that are more strongly altered than in PD patients without T2DM.

Our study had several limitations to note. First, HRM analyses were performed after the onset of both T2DM and PD and therefore, detected metabolite alterations likely capture metabolic responses related to existing PD and T2DM. This information can be very informative for understanding disease progression and biological pathways affected by both diseases. However, further longitudinal studies assessing metabolomic patterns prior to disease onset will be needed for causal inferences. The analyses among non-PD participants were also underpowered. So, while we report associations related to T2DM among PD patients, many of which were not related to T2DM among the non-PD participants, the smaller sample size limits conclusions related to the specificity. There are also limitations related to metabolite annotation and pathway analysis. Many of the metabolic features could not be annotated or were annotated one to many. However, we did make use on an in-house library, providing confidence in features we were able to match^72^. Pathway analysis also depends on linking metabolites to pathways, which requires some level of annotation. *mummichog* pathway analysis is designed for untargeted, unannotated MWAS data^73^. But many top features could not be linked to annotation even using *mummichog*. However, given that the identification of metabolites and metabolic pathways is based on existing knowledge, when new information is made available, future analyses can use our summary statistics for annotation and pathway analysis. In addition, our summary statistics provide an opportunity for future meta-analytical explorations. Given our sample size, we were also unable to consider the effects due to better or worse diabetes disease management. In addition, T2DM medication use was almost perfectly correlated with T2DM status and therefore, we were not able to adjust T2DM medication use. This would be of interest for future studies as perhaps better T2DM disease management may mitigate the contributions of T2DM to PD progression. Finally, it is important to note that this is an initial discovery study to uncover the molecular link between PD and T2DM. Our untargeted metabolomics analysis needs to be replicated, but the knowledge gained by this approach may help to eventually improve the prognosis of PD patients with T2DM in the future.

Our study provides insights into metabolic profiles related uniquely to diabetes among PD patients. The differences in metabolites and dysregulated metabolic pathways shed some light on mechanisms that possibly contributed to PD progression or pathogenesis and may eventually provide targets for disease management for comorbid patients. We found that the most important metabolic pathways associated with T2DM do not depend on PD status as expected. However, more informative are pathways we found to be uniquely associated with diabetes among PD patients, including amino acid, nucleic acid, and fatty acid metabolisms pathways. These pathways implicate the enhanced dysregulation of energy metabolism, nitrogen balance, mitochondrial dysfunction, and oxidative stress as potential contributing factors to progression in PD patients with T2DM as these pathways were found to be enriched even compared to PD patients without T2DM.

## Methods

### Study population

PD patients were recruited as part of a community-based case-control study, the Parkinson’s Gene and Environment (PEG) study. Idiopathic PD patients were recruited between 2000 and 2017 from central California (Kern, Fresno, and Tulare) in two separate study waves, referred to as PEG1 and PEG2. PEG1 participants were recruited between 2001 and 2007 and PEG2 participants between 2011 and 2017. Eligibility criteria for cases included: living in California for five years at minimum, having been diagnosed with PD for ≤3 years for PEG1 and ≤5 years for PEG2, and agreeing to participate in the study. PD patients were recruited from local clinics, neurologists, medical groups, radio advertisements, and the California PD registry. Most of the PD patients (>70%) were taking PD medications (see Supplementary Table 1). Population controls for PD were randomly sampled from the study area using either Medicare enrollee lists (prior to Health Insurance Portability and Accountability Act) or residential parcels listed in property tax assessor records. PEG1 and PEG2 also enrolled a set of household controls for PD patients. These household controls for PD were limited to one per household. Eligibility criteria for controls included: being more than 35 years old, living in California for five years at minimum, and not being diagnosed with PD. Household controls had an additional requirement of living in the same household with the PD participants for at least 1 year. In total, 831 PD patients and 873 non-PD older adults were enrolled in the PEG study. Detailed study population and recruitment methods were published elsewhere^74,75^. For our analysis, we included 636 PD patients and 253 non-PD older adults with metabolomics data. These were all study participants with serum available for metabolic profiling. The participants contributing to the metabolomics data included in the analysis were similar in covariate distribution to those without metabolomics data (see Table 1 and Supplementary Table 1).

We collected demographic and lifestyle information including age (age at interview for controls and diagnosis for PD patients), gender, race/ethnicity, education, smoking status, as well as their self-reported medical history, including T2DM status and age of diagnosis for T2DM. We excluded PD patients and non-PD participants without blood samples or who did not report their medical history. We imputed education, smoking status, and race/ethnicity for 7 PD patients (education only) and 5 non-PD patient controls.

We obtained blood samples at enrollment and all lab staff followed the same sample processing and storing procedure. A detailed procedure was published previously^76^. Briefly, after blood was drawn, we let the blood sit for 30 min to clot. We then centrifuged the blood for 20 min to remove the clot and aliquoted 1 mL of the serum per 1.5 mL microcentrifuge tubes. Biospecimens were then transferred from the field on dry ice and stored in a −80 °C freezer at UCLA within the same day. Serum samples were mailed out on dry ice and analyzed for metabolomics at Emory University. We used serum instead of plasma primarily due to sample availability.

### High-resolution metabolomics (HRM)

Briefly, serum samples along with quality control samples were centrifuged and analyzed using both hydrophilic interactions (HILIC) chromatography with positive electrospray ionization (ESI) and C18 chromatography with negative ESI (Ultimate 3000, Q-Exactive HF, Thermo Fisher, *m*/*z* range 85–1275)^72,76^. The samples were analyzed in batches of 40. Two quality control samples were used: NIST 1950, which was run as the first and last sample, and commercially purchased plasma pooled from an unknown number of males and females, which were run at the beginning, middle, and end of each batch. HRM detected ions by producing metabolic features including mass-to-charge ratio (*m*/*z*), retention time (rt), and abundance. The raw data were extracted and aligned using apLCMS^77^ with modifications by xMSanalyzer^78^. Batch effects were corrected by using ComBat from the sva package^79^. For data analyses, we only included metabolomic features detected in >50% of all samples, with median coefficients of variation (CV) among technical replicates <75% and Pearson correlation >0.7. The metabolic abundance was imputed for missing values with minimum value of each metabolic feature, median-normalized, and auto-scaled.

### Annotation

Confirmed metabolites were identified by comparison to an in-house library of reference standards that includes over 300 metabolites that were previously analyzed using identical analytical parameters^72,80^ using a mass error of <10 ppm and a retention time difference of <30 s. Metabolites without standard available were annotated using xMSannotator^81^. We retained annotations with a confidence score >2, mass error <10 ppm for *m*/*z*, and retention time difference <30 s for grouping adducts and isotopes.

### Statistical analysis

We conducted a metabolome-wide association analysis (MWAS) to assess each metabolic feature’s association with T2DM among PD patients (i.e., comparing PD patients with and without T2DM to each other) using logistic regression and controlling for age of PD diagnosis, gender, education, smoking, non-European ancestry, and study wave. We applied a false discovery rate (FDR) to correct for multiple testing. We then conducted pathway enrichment analysis using mummichog version 2 with metabolic features including m/z, retention time, and test statistics from the logistic model (z-value and crude *p*-value)^82^. Mummichog primarily uses the Kyoto Encyclopedia of Genes and Genomes (KEGG), UCSD Recon1, and the Edinburgh human metabolic network. Unlike the traditional method that identified metabolites and then maps them to the metabolic network, Mummichog takes untargeted data and maps all possible metabolites, and then searches for local enrichment. The algorithm has been previously validated^73^. For the mummichog analysis parameters, we selected the M[1+], M + H[1+], or M+Na[1+] adduct types for positive ion mode feature annotation and M-H[-], M-2H[2-], or M-H2O-H[-] for the negative ions. We repeated the same MWAS and pathway enrichment analysis to assess associations with T2DM among the non-PD participant group. We controlled for the same set of potential confounders except for the age of enrollment in place of the age of PD diagnosis. We adjusted for multiple testing, both based on just replication of the T2DM-associated metabolites among PD patients and for the full MWAS. The sample size for the non-PD participant group was limited, therefore, we also present results from a power analysis. Furthermore, to take into account a potential lack of statistical power due to the smaller number of non-PD participants, we compared enrichment analyses using a significance level of 0.15 and 0.05 for metabolic features.

We additionally performed 3-fold cross-validation to assess the generalizability of the findings using an FDR-adjusted *p*-value of 0.05 in the discovery population to identify associated metabolic features and a crude *p*-value of 0.10 in the replication population to identify whether metabolites in the discovery population were replicated. We chose these cut-off *p* values due to the smaller sample size when splitting the population into 3 folds. We then calculated a significance rate as the number of significantly replicated metabolic features in the replicate sample divided by the total number of significant metabolic features in the discovery sample.

To identify metabolites associated with both T2DM and PD in the study population, we compared our present T2DM MWAS results with previously performed PD MWAS results^15^. We also performed several sensitivity analyses for the metabolic features identified in the main analysis. We present gender-stratified and race/ethnicity-stratified results to assess the potential effect measure modification. To assess potential confounding, we additionally adjusted the T2DM MWAS among PD patients for the amount of levodopa equivalent daily dose taken in mg, and for the sample collection among both PD and non-PD participants. We also compared the results using T2DM diagnosis with T2DM medication treatment, comparing the metabolic differences between participants who reported both having T2DM and using T2DM medication with those participants who reported not having T2DM and not using T2DM medication.

All analyses were performed in R version 4.1.3.

### Reporting summary

Further information on research design is available in the Nature Research Reporting Summary linked to this article.

### Supplementary information

Supplementary Figures and Tables

Reporting Summary

## Data Availability

The data is publicly available at Metabolomics Workbench [project (PR001964): 10.21228/M8VD96]. The complete summary statistics for the metabolic features associated with T2DM are available through figshare (10.6084/m9.figshare.22589464).

## Code availability

The code is available on GitHub at https://github.com/sherlockli1/metabolomics-PD-and-DM.git.
