# Supplementary material for: Untargeted serum metabolic profiling of diabetes mellitus among Parkinson’s disease patients
Source: NPJ Parkinsons Dis. 2024 May 10;10:100. doi: 10.1038/s41531-024-00711-4 (PMC11087477; doi:10.1038/s41531-024-00711-4)
Supplement: Supplementary file 1 — Supplementary Figures and Tables [file 41531_2024_711_MOESM1_ESM.pdf]

Supplementary Figure 1. Comparing metabolomics-wide association analysis (MWAS) results for gender stratified analysis in HILIC (A and B) and C18 (C and D) colored by significance level adjusted for multiple testing from full MWAS.

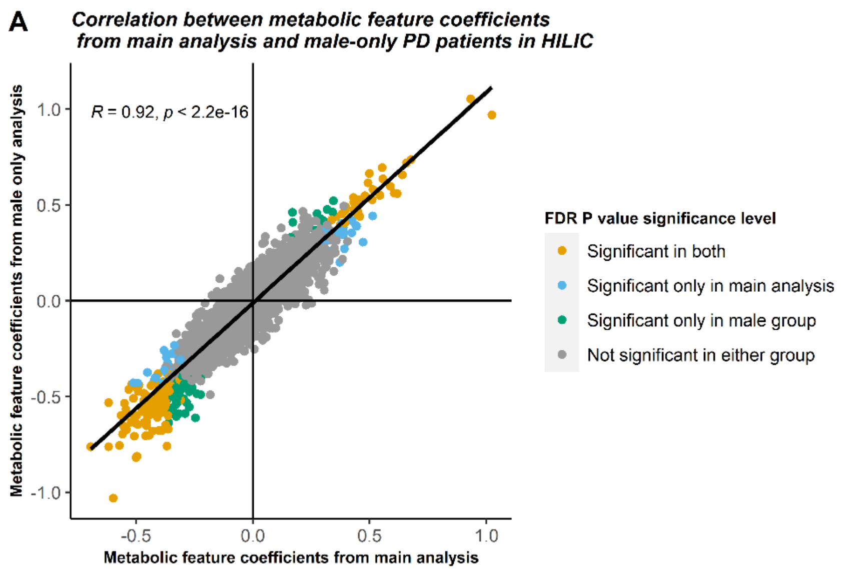

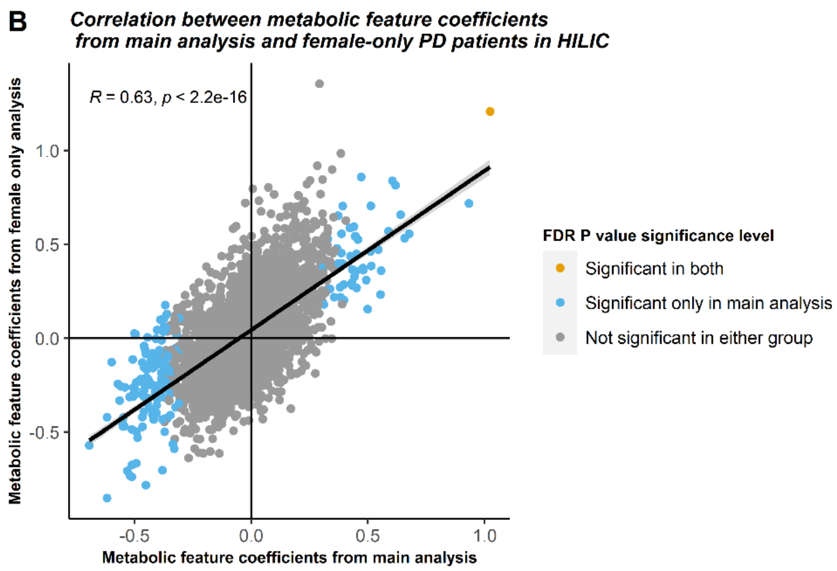

Significant in both	Significant only in main analysis	Significant only in male group	Not significant in either group
155	37	57	2664

Significant in both	Significant only in main analysis	Significant only in female group	Not significant in either group
1	191	0	2721

Supplementary Figure 1 Continued. Comparing metabolomics-wide association analysis (MWAS) results for gender stratified analysis in HILIC (A and B) and C18 (C and D) colored by significance level adjusted for multiple testing from full MWAS.

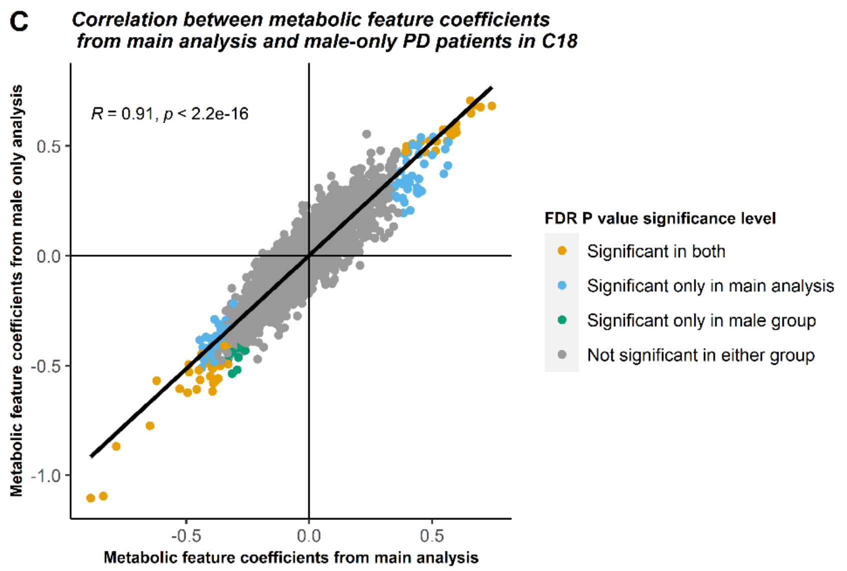

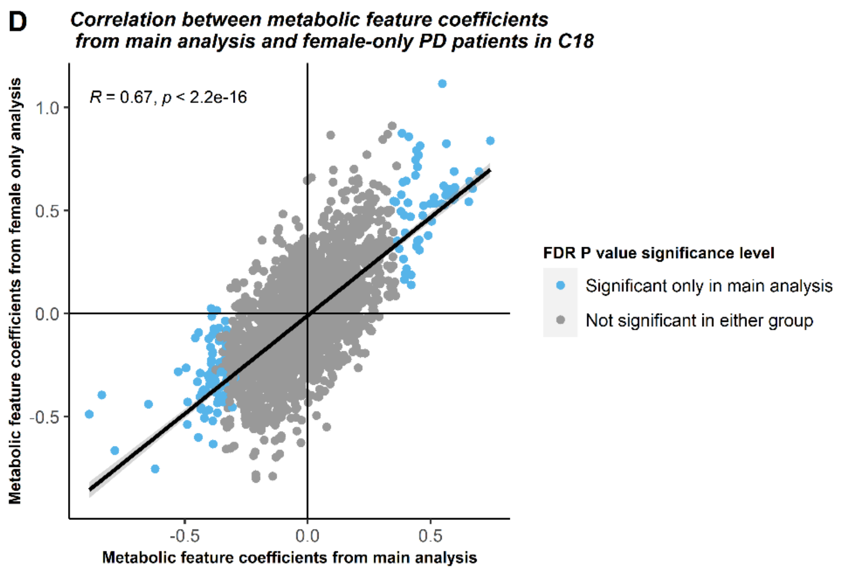

Significant in both	Significant only in main analysis	Significant only in male group	Not significant in either group
61	72	8	2081

Significant in both	Significant only in main analysis	Significant only in female group	Not significant in either group
0	133	0	2089

Supplementary Figure 2. Comparing metabolomics-wide association analysis (MWAS) results for race stratified analysis in HILIC (A and B) and C18 (C and D) colored by significance level adjusted for multiple testing from full MWAS.

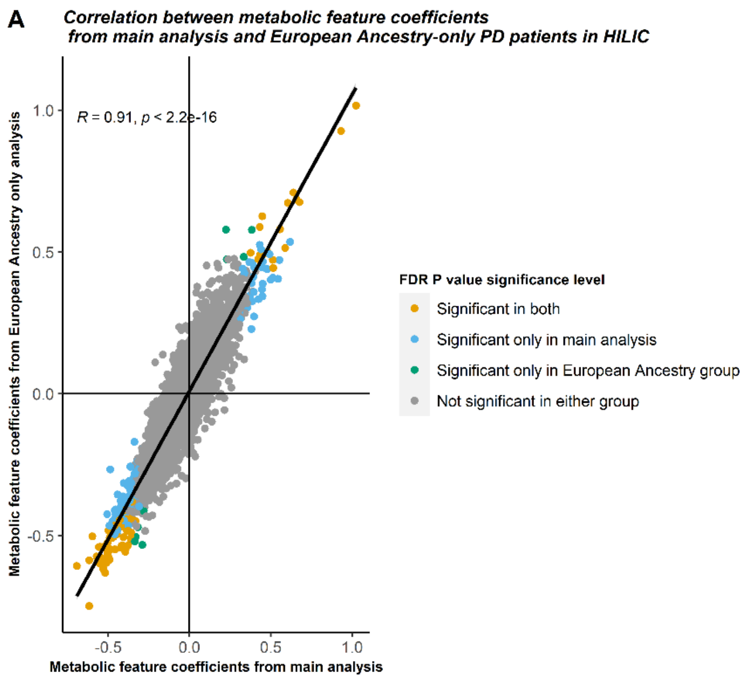

Significant in both	Significant only in main analysis	Significant only in European Ancestry group	Not significant in either group
80	112	10	2711

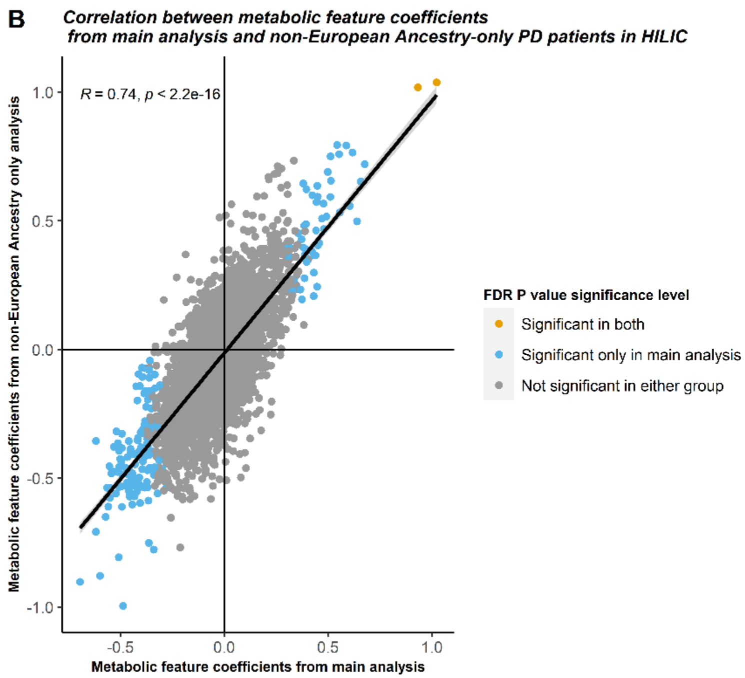

Significant in both	Significant only in main analysis	Significant only in non-European Ancestry group	Not significant in either group
2	190	0	2721

Supplementary Figure 2 Continued. Comparing metabolomics-wide association analysis (MWAS) results for race stratified analysis in HILIC (A and B) and C18 (C and D) colored by significance level adjusted for multiple testing from full MWAS.

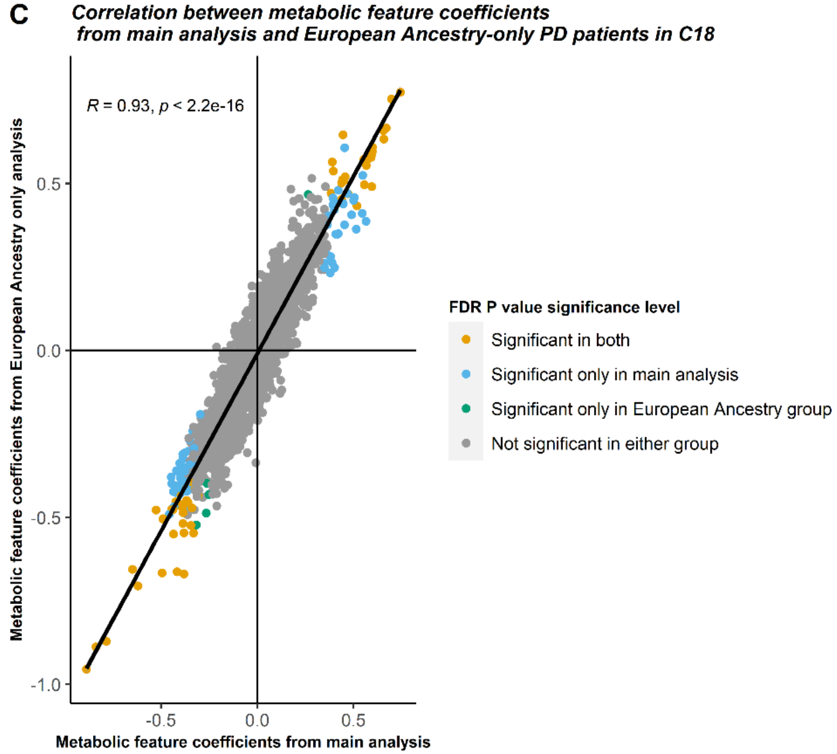

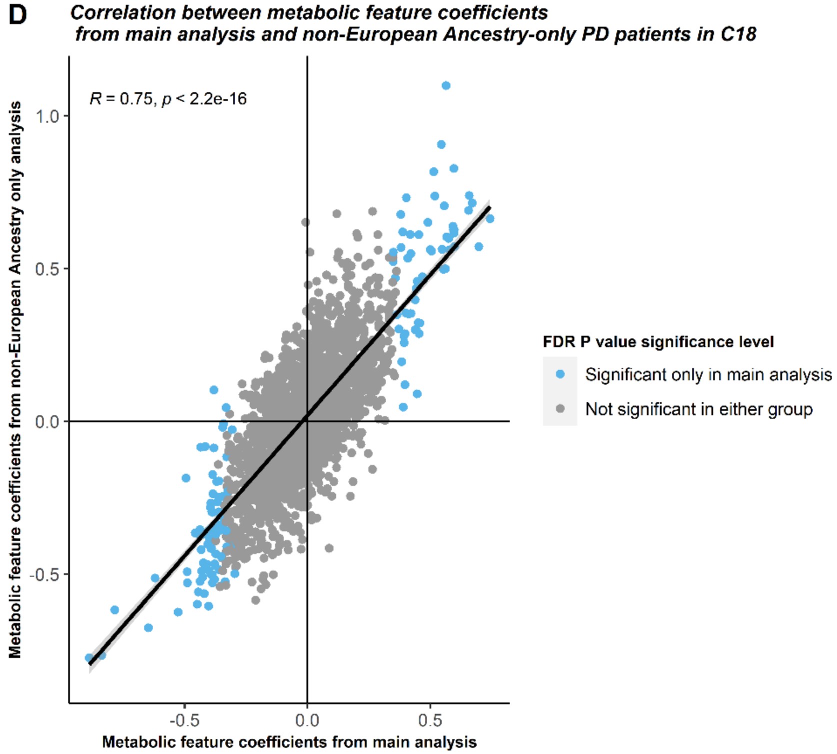

Significant in both	Significant only in main analysis	Significant only in European Ancestry group	Not significant in either group
61	72	6	2083

Significant in both	Significant only in main analysis	Significant only in non-European Ancestry group	Not significant in either group
0	133	0	2089

Supplementary Figure 3. Comparing discovery and replication samples' beta coefficients from cross validation among PD patients in HILIC (A, B, and C) and C18 (D, E and F) colored by significantly replicated metabolic features.

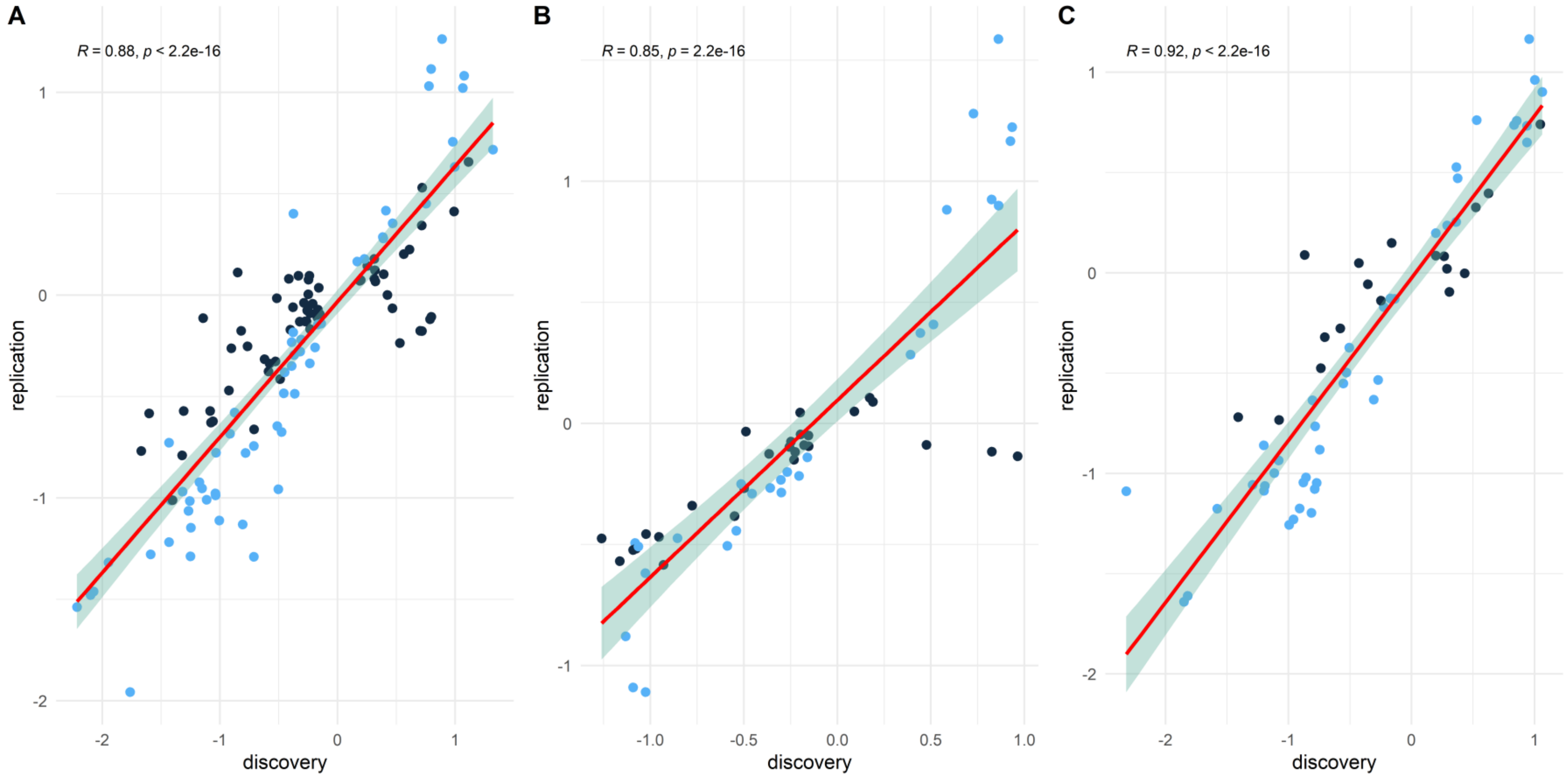

Supplementary Figure 3 Continued. Comparing discovery and replication samples' beta coefficients from cross validation among PD patients in HILIC (A, B, and C) and C18 (D, E and F) colored by significantly replicated metabolic features.

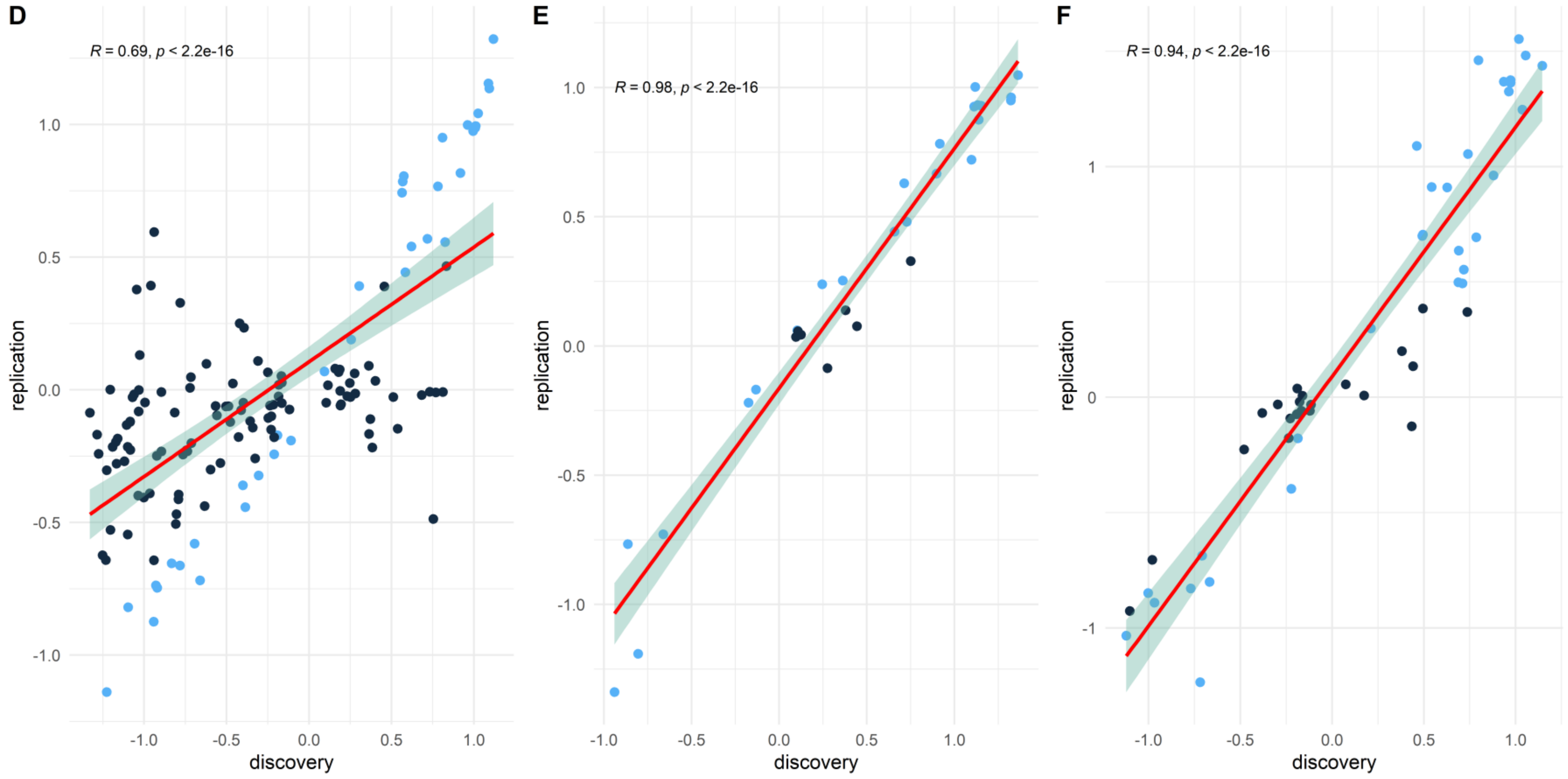

Supplementary Figure 4. Comparing discovery and replication samples' beta coefficients from cross validation among non-PD participants in HILIC (A, B, and C) and C18 (D, E and F) colored by significantly replicated metabolic features.

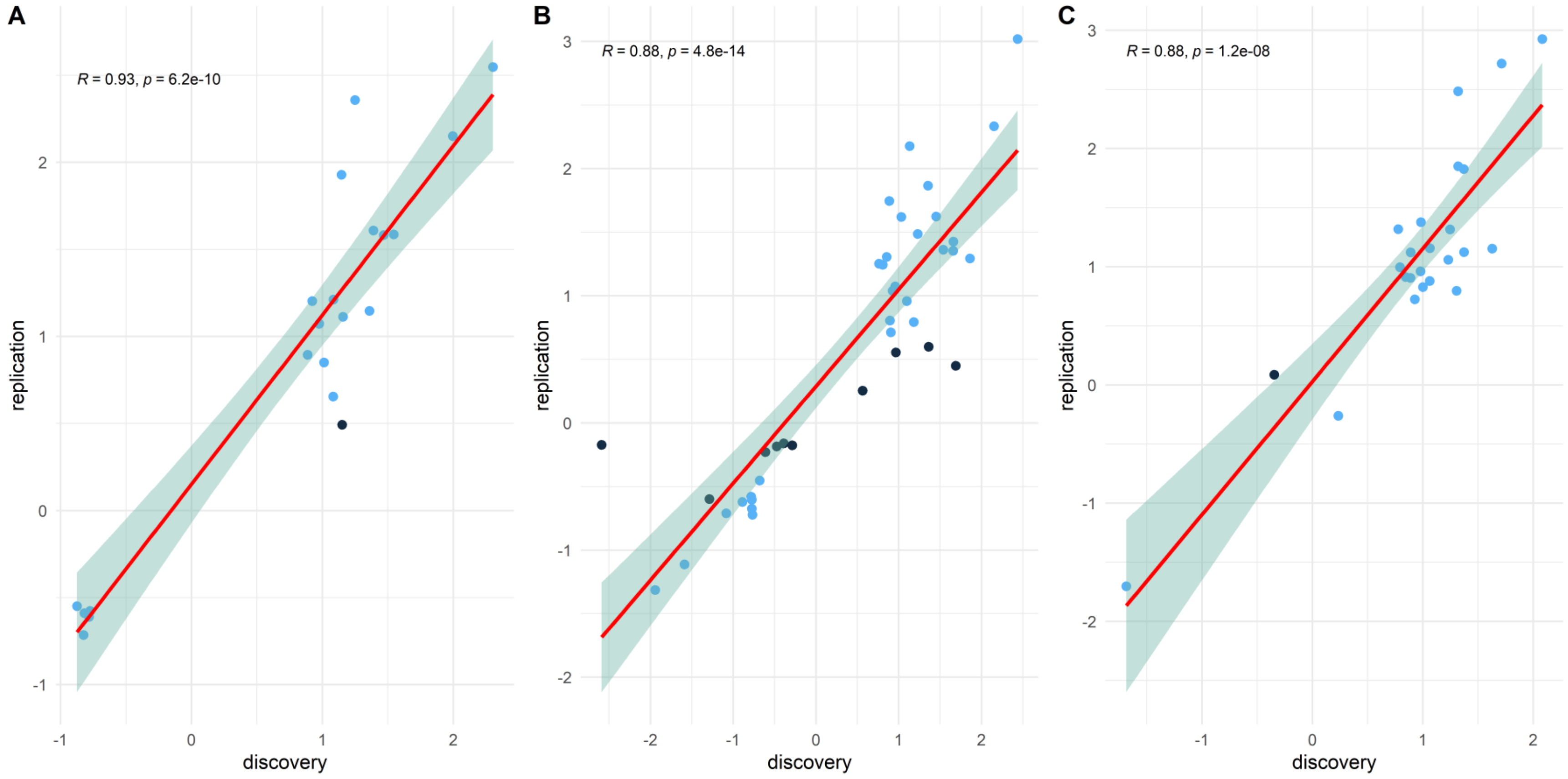

Supplementary Figure 4 Continued. Comparing discovery and replication samples' beta coefficients from cross among non-PD participants in HILIC (A, B, and C) and C18 (D, E and F) colored by significantly replicated metabolic features.

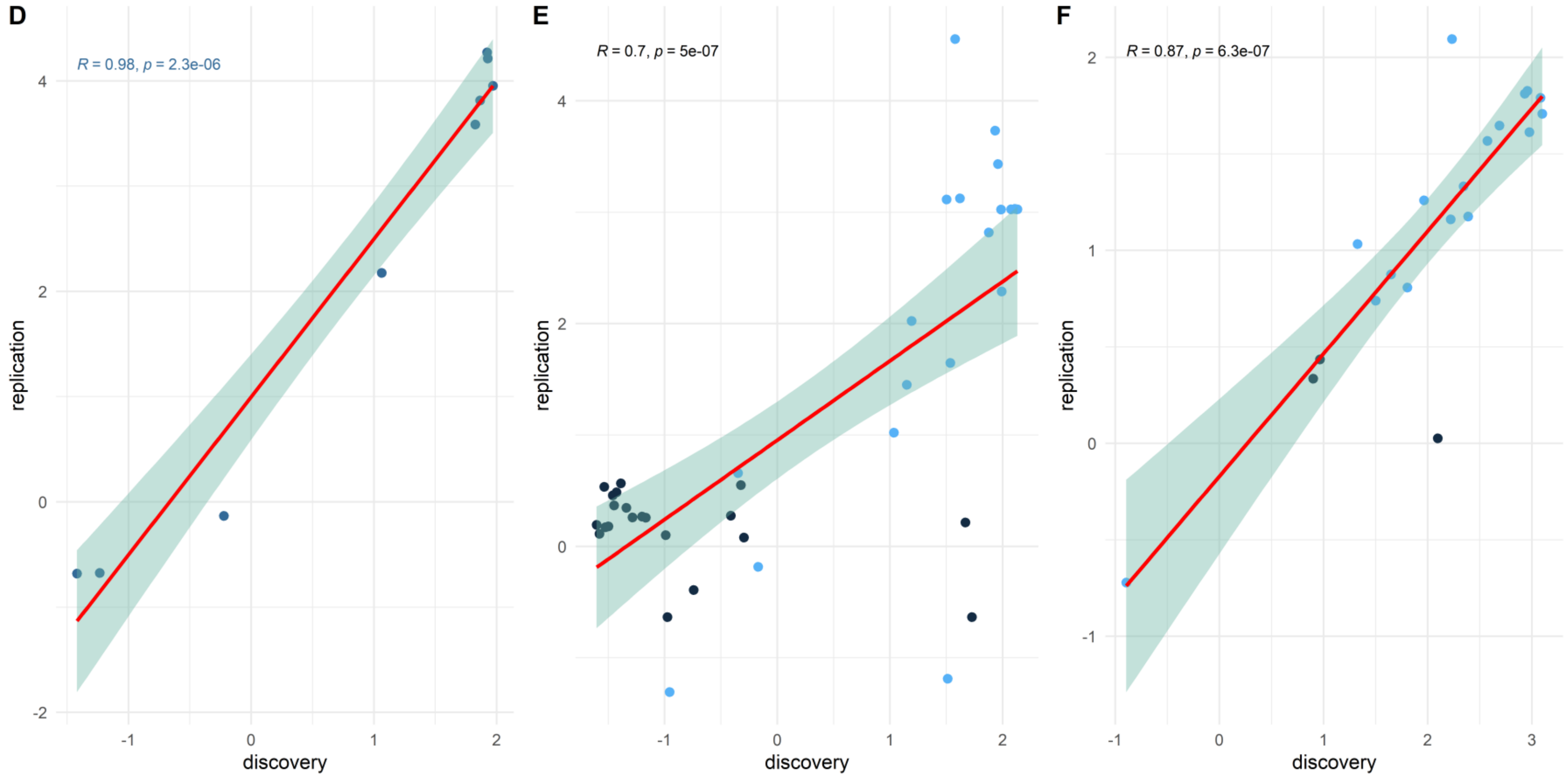

Supplementary Figure 5. Comparing metabolomics-wide association analysis (MWAS) results adjusting and not adjusting Parkinson's disease medication among PD participants colored by significance level adjusted for multiple testing from full MWAS.

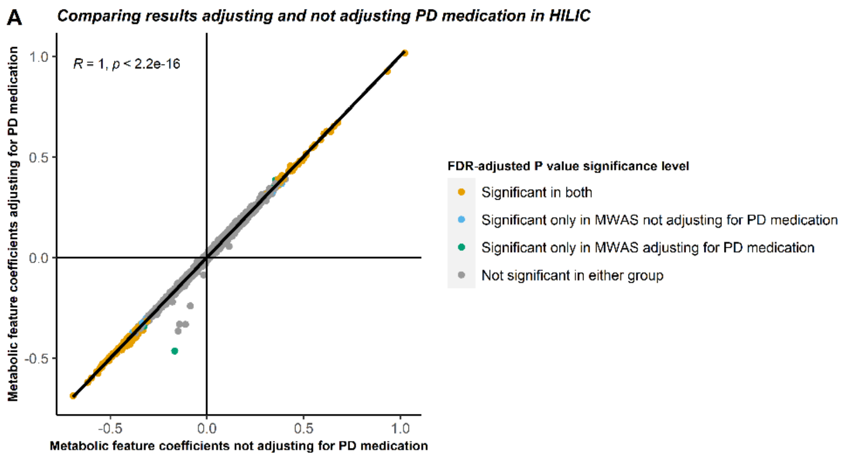

Significant in both	Significant only in MWAS not adjusting for PD medication	Significant only in MWAS adjusting for PD medication	Not significant in either group
179	13	4	2717

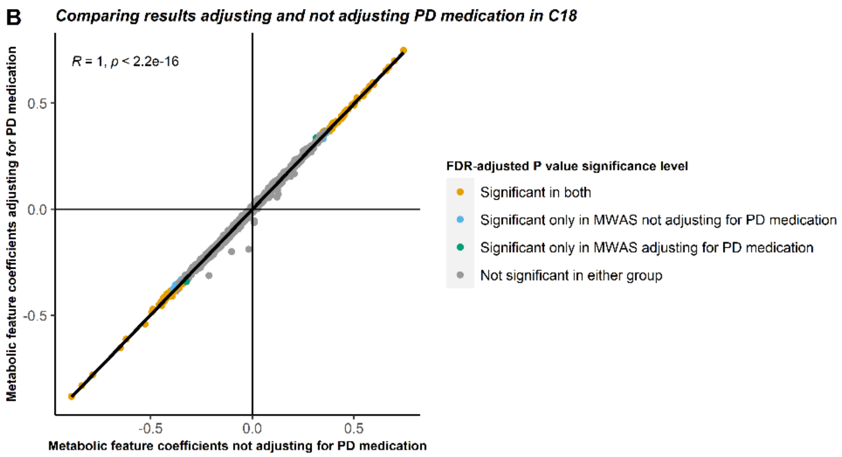

Significant in both	Significant only in MWAS not adjusting for PD medication	Significant only in MWAS adjusting for PD medication	Not significant in either group
119	14	2	2087

Supplementary Figure 6. Comparing metabolomics-wide association analysis (MWAS) results adjusting and not adjusting sample collection year among PD participants colored by significance level adjusted for multiple testing from full MWAS.

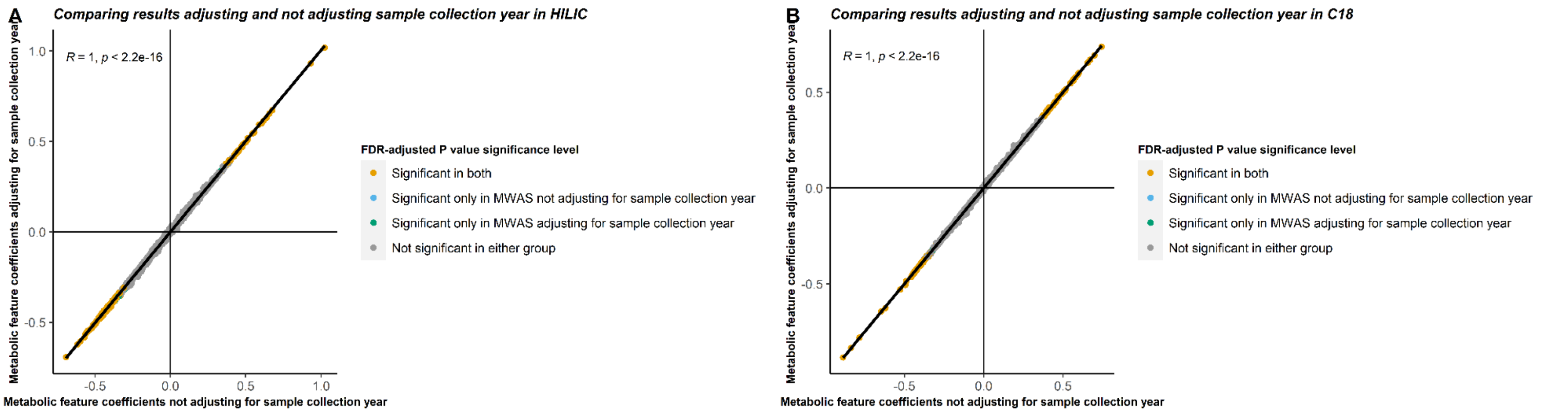

Significant in both	Significant only in MWAS not adjusting for sample collection year	Significant only in MWAS adjusting for sample collection year	Not significant in either group
191	1	8	2713

Significant in both	Significant only in MWAS not adjusting for sample collection year	Significant only in MWAS adjusting for sample collection year	Not significant in either group
132	1	1	2089

Supplementary Figure 7. Comparing metabolomics-wide association analysis (MWAS) results adjusting and not adjusting sample collection year among non-PD participants colored by significance level adjusted for multiple testing from full MWAS.

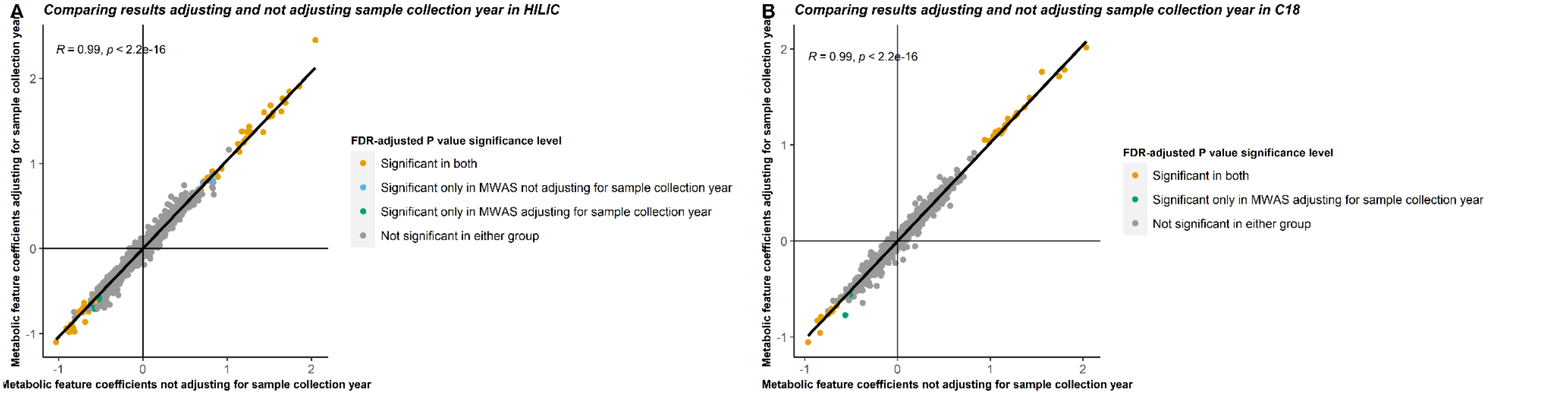

Significant in both	Significant only in MWAS not adjusting for sample collection year	Significant only in MWAS adjusting for sample collection year	Not significant in either group
61	1	5	2846

Significant in both	Significant only in MWAS not adjusting for sample collection year	Significant only in MWAS adjusting for sample collection year	Not significant in either group
32	0	3	2187

Supplementary Figure 8. Comparing metabolomics-wide association analysis (MWAS) results for type 2 diabetes mellitus status and for type 2 diabetes mellitus medication use among Parkinson's disease participants in HILIC and C18 colored by significance level adjusted for multiple testing from full MWAS.

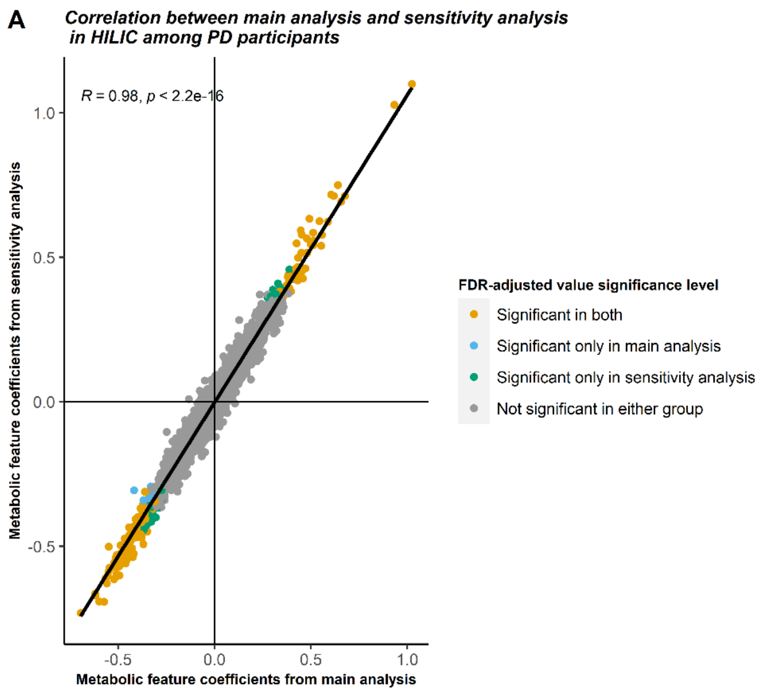

Significant in both	Significant only in main analysis	Significant only in sensitivity analysis	Not significant in either group
187	5	48	2673

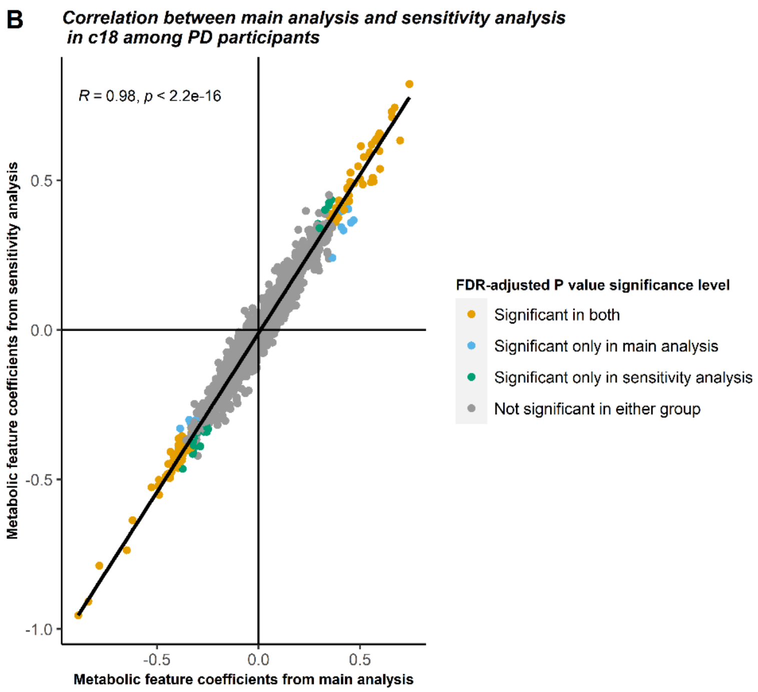

Significant in both	Significant only in main analysis	Significant only in sensitivity analysis	Not significant in either group
121	12	40	2049

Supplementary Figure 9. Comparing metabolomics-wide association analysis (MWAS) results for type 2 diabetes mellitus status and for type 2 diabetes mellitus medication use among non-Parkinson’s disease participants in HILIC and C18 colored by significance level adjusted for multiple testing from full MWAS.

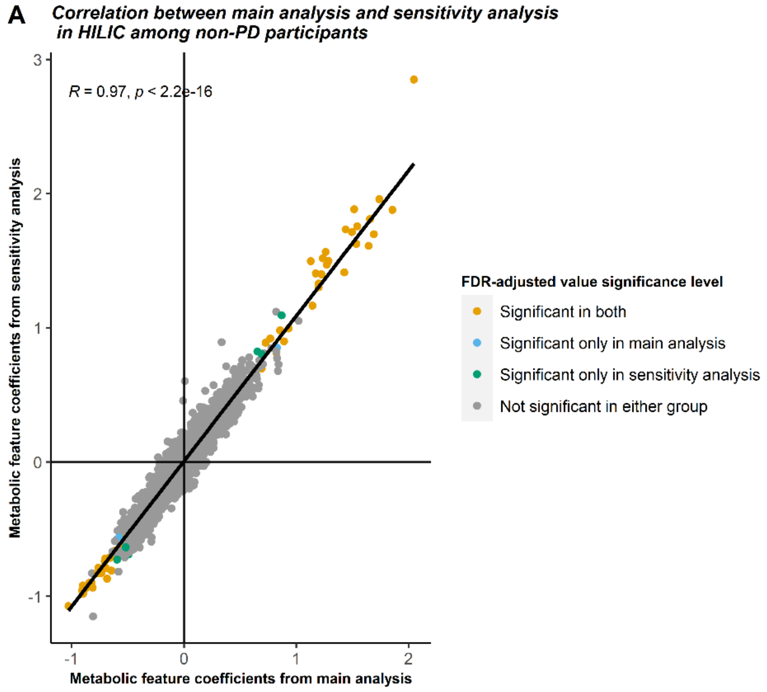

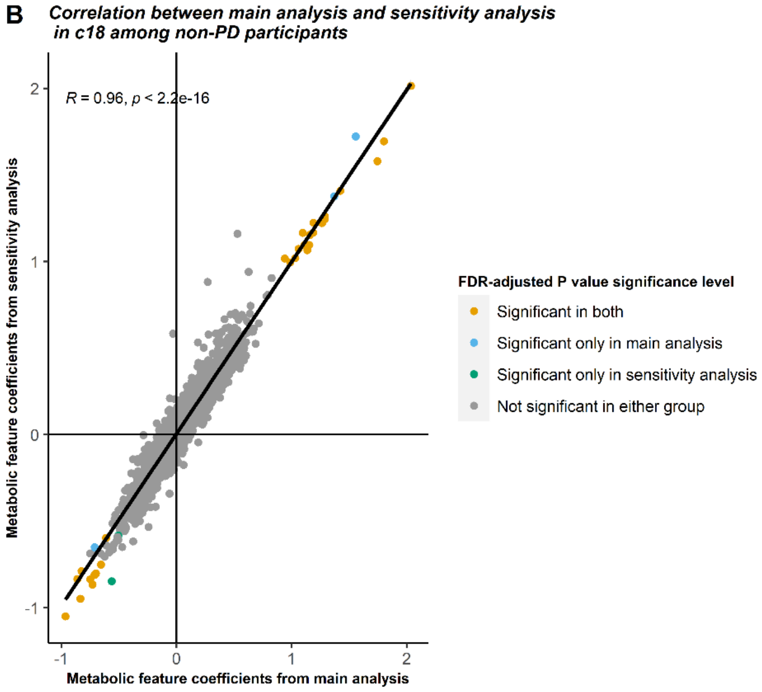

Significant in both	Significant only in main analysis	Significant only in sensitivity analysis	Not significant in either group
60	2	7	2844

Significant in both	Significant only in main analysis	Significant only in sensitivity analysis	Not significant in either group
29	3	2	2188

Supplementary Table 1. Characteristics of Parkinson's disease (PD) patients and non-PD participants with and without metabolomics by diabetes.

		Without Metabolomics Data			
		**PD Patients (N = 194)[1]**		**non-PD Participants (N = 607)[1]**	
**Characteristic**	**Statistics**	**Diabetes (N = 33)**	**No Diabetes (N = 161)**	**Diabetes (N = 118)**	**No Diabetes (N = 489)**
Age[2]	Mean (SD)	70.42 (8.87)	70.54 (11.39)	68.03 (8.50)	65.59 (11.67)
Male Gender	n (%)	23 (70%)	95 (59%)	61 (52%)	211 (43%)
Years of Education ≥12 Years	n (%)	26 (79%)	137 (85%)	95 (81%)	418 (85%)
Past or Current Smoker	n (%)	19 (58%)	64 (40%)	66 (56%)	237 (48%)
Non-European Descent	n (%)	10 (30%)	27 (17%)	53 (45%)	157 (32%)
Race and Ethnicity	n (%)				
White		23 (70%)	134 (83%)	65 (55%)	332 (68%)
Black		0 (0%)	0 (0%)	7 (5.9%)	17 (3.5%)
Latino		9 (27%)	17 (11%)	34 (29%)	95 (19%)
Asian		1 (3.0%)	7 (4.3%)	4 (3.4%)	18 (3.7%)
Other		0 (0%)	3 (1.9%)	8 (6.8%)	25 (5.1%)
Unspecified		0 (0%)	0 (0%)	0 (0%)	2 (0.4%)
Study Wave					
PEG1	n (%)	8 (24%)	70 (43%)	30 (25%)	173 (35%)
PEG2	n (%)	25 (76%)	91 (57%)	88 (75%)	316 (65%)
Levodopa Use	n (%)	23 (70%)	115 (71%)	NA	NA
Amount of levodopa equivalent daily dose taken (mg)	Mean (SD)	359.09 (295.92)	330.59 (320.73)	NA	NA
Diabetes Medication Use	n (%)	27 (82%)	NA	105 (89%)	NA
Duration of diabetes medication use	Mean (SD)	9.20 (7.15)	NA	7.66 (6.83)	NA
Sample Collection Year	Mean (SD)	NA	NA	NA	NA

1. One PD patient and 13 non-PD participants missing diabetes status.
2. Age refers to age of PD diagnosis for PD patient group and age of enrollment in the study for non-PD participants.

Supplementary Table 1 Continued. Characteristics of Parkinson's disease (PD) patients and non-PD participants with and without metabolomics by diabetes.

With Metabolomics Data			
**PD Patients (N = 636)**		**Non-PD Participants (N = 253)**	
**Diabetes (N = 96)**	**No Diabetes (N = 540)**	**Diabetes (N = 36)**	**No Diabetes (N = 217)**
68.03 (9.83)	66.62 (10.44)	68.92 (11.47)	65.39 (12.93)
65 (68%)	341 (63%)	22 (61%)	97 (45%)
75 (78%)	455 (84%)	30 (83%)	195 (90%)
45 (47%)	252 (47%)	17 (47%)	117 (54%)
40 (42%)	117 (22%)	11 (31%)	38 (18%)
56 (58%)	423 (78%)	24 (69%)	176 (82%)
4 (4.2%)	1 (0.2%)	0 (0%)	3 (1.4%)
29 (30%)	82 (15%)	9 (26%)	24 (11%)
2 (2.1%)	12 (2.2%)	1 (2.9%)	2 (0.9%)
5 (5.2%)	21 (3.9%)	1 (2.9%)	9 (4.2%)
0 (0%)	1 (0.2%)	1 (2.9%)	3 (1.4%)
33 (34%)	248 (46%)	27 (75%)	163 (75%)
63 (66%)	292 (54%)	9 (25%)	54 (25%)
71 (74%)	395 (73%)	NA	NA
362.50 (338.92)	314.90 (281.47)	NA	NA
86 (90%)	NA	32 (89%)	NA
10.60 (9.88)	NA	6.87 (4.80)	NA
2010 (4.59)	2009 (5.02)	2,007 (6.35)	2,008 (6.56)

Supplementary Table 1 Continued. Characteristics of Parkinson's disease (PD) patients and non-PD participants with and without metabolomics by diabetes.

		PD Participants with Metabolomics Data (N = 636)		Non-PD Participants with Metabolomics Data (N = 253)	
**Characteristic**	**Statistics**	**PEG1 (N = 281)**	**PEG2 (N = 355)**	**PEG1 (N = 190)**	**PEG2 (N = 63)**
Type 2 Diabetes	n (%)	33 (12%)	63 (18%)	27 (14%)	9 (14%)
Age	Mean (SD)	67.42 (10.13)	66.37 (10.53)	66.27 (13.52)	64.75 (10.18)
Male Gender	n (%)	165 (59%)	241 (68%)	97 (51%)	22 (35%)
Years of Education ≥12 Years	n (%)	232 (83%)	298 (84%)	170 (89%)	55 (87%)
Past or Current Smoker	n (%)	139 (49%)	158 (45%)	108 (57%)	26 (41%)
Non-European Descent	n (%)	61 (22%)	96 (27%)	29 (15%)	20 (32%)
T2DM Medication Use	n (%)	27 (9.6%)	59 (17%)	24 (13%)	8 (13%)
Duration of diabetes medication use	Mean (SD)	10.08 (10.45)	9.38 (9.22)	6.61 (4.91)	8.61 (6.79)
Sample Collection Year	Mean (SD)	2004 (2.64)	2013 (1.44)	2004 (2.54)	2018 (0.49)
Amount of levodopa equivalent daily dose taken (mg)	Mean (SD)	294.12 (261.94)	344.22 (310.80)	NA	NA

Supplementary Table 2. Replication of MWAS results from PD participants in non-PD participants.						
HILIC						
mz	rt	beta	se	zvalue	pvalue	FDR_pvalue
271.04	74.974	1.738012644	0.313117508	5.550672185	2.84573E-08	5.46381E-06
225.034	75.303	1.494525527	0.280285182	5.332160335	9.70512E-08	9.31691E-06
203.053	75.296	1.197892487	0.23159807	5.172290446	2.31242E-07	1.13795E-05
204.056	74.501	1.197470323	0.231724924	5.167637148	2.37072E-07	1.13795E-05
226.038	75.461	1.654308382	0.324551404	5.097215291	3.44686E-07	1.3236E-05
104.992	76.36	1.286185876	0.257155682	5.001584503	5.6861E-07	1.81955E-05
219.026	74.325	1.173070117	0.237519703	4.938832864	7.85915E-07	2.15565E-05
244.079	71.325	1.143798528	0.234304888	4.881667384	1.05193E-06	2.52462E-05
504.871	71.618	1.690156466	0.355786397	4.750480847	2.02934E-06	4.32925E-05
207.024	79.355	1.426746852	0.301998192	4.724355608	2.30846E-06	4.43225E-05
324.99	75.983	-1.029589401	0.22385186	-4.59942303	4.23663E-06	6.7786E-05
496.84	72.752	1.235443178	0.268213497	4.606193166	4.10107E-06	6.7786E-05
318.97	76.169	1.224393884	0.271827053	4.504312102	6.65883E-06	9.83458E-05
231.055	75.316	-0.898699785	0.203051695	-4.425965437	9.60119E-06	0.000131673
326.987	76.127	-0.824238316	0.191321785	-4.308125785	1.64644E-05	0.000210744
106.999	75.195	-0.857602603	0.205411012	-4.175056609	2.97912E-05	0.000357494
165.013	74.843	0.931350934	0.224272717	4.152760746	3.28488E-05	0.000370998
160.133	55.358	0.8548371	0.213660986	4.000904027	6.31009E-05	0.000673077
335.094	86.335	0.766940991	0.195975633	3.913450756	9.09865E-05	0.000919442
177.106	100.743	-0.768846468	0.201679425	-3.812220649	0.000137724	0.001322149
198.085	100.113	-0.763212002	0.201767996	-3.78262171	0.000155185	0.001418836
176.103	102.372	-0.746861958	0.19932973	-3.746866847	0.000179057	0.00156268
159.076	102.224	-0.734094855	0.197394272	-3.718926839	0.000200071	0.001609476
160.08	100.482	-0.737781682	0.19846048	-3.71752443	0.000201185	0.001609476
113.071	101.512	-0.735940478	0.198702486	-3.703730598	0.000212452	0.00163163
199.088	100.117	-0.706873972	0.193253991	-3.65774579	0.000254443	0.001878965
177.1	100.826	-0.697160759	0.199769791	-3.489820737	0.000483345	0.003437118
138.013	74.729	-0.646475758	0.186955835	-3.457906289	0.000544391	0.003604242

163.06	105.755	0.690596095	0.199523582	3.461225426	0.000537722	0.003604242
570.665	67.778	-0.686188002	0.207357872	-3.309196784	0.000935641	0.005988099
744.59	42.524	-0.519017373	0.162871869	-3.186660633	0.001439255	0.008914098
740.546	67.144	-0.61731451	0.194649051	-3.171423171	0.00151694	0.009101639
1154.245	67.712	-0.438703349	0.142445112	-3.079806265	0.002071353	0.012051507
252.108	109.021	0.601744457	0.200012584	3.008532987	0.002625123	0.014824224
326.805	68.896	-0.59194387	0.207820435	-2.848342948	0.004394754	0.024108364
745.593	42.325	-0.426188737	0.150406075	-2.833587247	0.004602874	0.024548662
322.81	69.374	-0.582247481	0.208182148	-2.796817534	0.005160867	0.026780715
672.558	68.111	-0.560774346	0.201938474	-2.776956448	0.005487053	0.027724058
358.057	88.539	-0.441371459	0.162294707	-2.719567795	0.006536729	0.031376301
566.67	67.811	-0.538600523	0.197838283	-2.722428208	0.006480411	0.031376301
161.137	55	0.820326057	0.308496583	2.659109052	0.00783476	0.034983114
205.043	58.162	-0.542472547	0.203231186	-2.669238699	0.00760234	0.034983114
390.798	67.798	-0.529603054	0.198664272	-2.665819318	0.007680096	0.034983114
508.711	67.545	-0.517663678	0.196627354	-2.632714455	0.008470553	0.036962412
1162.277	67.73	-0.473739272	0.183666129	-2.579350226	0.009898637	0.040557349
382.766	68.554	-0.527033855	0.204320025	-2.57945278	0.009895699	0.040557349
614.6	67.891	-0.527996935	0.204783046	-2.57832348	0.009928101	0.040557349
146.6	96.634	-0.450458958	0.176169624	-2.556961568	0.010559088	0.042236353
336.834	67.349	-0.485140249	0.193761711	-2.503798334	0.012286805	0.048144214
496.686	67.924	-0.481957784	0.19443143	-2.478805943	0.0131823	0.050620034
682.586	67.347	-0.444279054	0.180530134	-2.460968954	0.013856236	0.052164652
130.123	27.204	0.605700097	0.249790335	2.424833998	0.015315388	0.055464013
245.049	81.304	0.494184292	0.204363843	2.418159124	0.015599254	0.055464013
334.836	67.586	-0.466435387	0.191818298	-2.431652207	0.015030132	0.055464013
386.76	68.644	-0.508440649	0.211124964	-2.408245051	0.016029418	0.05595724
202.119	68.492	1.018406649	0.437577073	2.327376621	0.019945229	0.067183929
482.36	49.589	-0.511978412	0.219831455	-2.328958843	0.019861246	0.067183929
438.727	68.044	-0.439567473	0.191110825	-2.300065807	0.021444492	0.070988664
444.719	68.358	-0.438164268	0.193425072	-2.265291995	0.023494767	0.076457547

480.345	47	-0.474044375	0.212388224	-2.231971087	0.025616874	0.081973996
210.888	69.976	-0.437722969	0.19939001	-2.195310438	0.028141337	0.08857601
353.9	79.616	0.406052023	0.187846297	2.161618459	0.030647597	0.094908689
402.083	87.844	-0.398010076	0.186539772	-2.133647272	0.032871665	0.100180313
257.147	74.625	-0.405126467	0.191309358	-2.117651067	0.03420463	0.10261389
1152.248	67.801	-0.363042587	0.174447149	-2.081103576	0.037424427	0.110545999
1034.331	67.999	-0.357185503	0.172550118	-2.070039165	0.038448677	0.111850697
307.019	63.703	-0.401978871	0.19643096	-2.046413003	0.040715754	0.116677981
214.023	130.222	-0.374159336	0.189707537	-1.972295578	0.048575874	0.137155409
207.897	68.486	-0.395914505	0.201498098	-1.964854805	0.049431045	0.137547255
214.18	26.723	-0.316343319	0.164674204	-1.921025341	0.054728513	0.150112492
483.364	49.807	-0.316421191	0.167139744	-1.893153502	0.058337459	0.157757635
220.067	99.688	-0.321564003	0.178623386	-1.800234615	0.0718236	0.191529601
247.14	110.194	0.315551948	0.179417275	1.758760123	0.078618261	0.206776795
147.555	96.563	-0.294066552	0.170061433	-1.729178378	0.083777175	0.217367805
221.988	85.683	-0.311392469	0.18433943	-1.689234192	0.091174559	0.233406871
1150.25	67.735	-0.247179366	0.14822917	-1.667548742	0.095405341	0.241024019
349.064	96.841	-0.289221339	0.174169611	-1.660572912	0.096799253	0.241369566
281.008	53.407	0.36612831	0.222049846	1.648856396	0.099177058	0.244128143
412.853	73.358	-0.294873284	0.182757303	-1.613469219	0.106642605	0.259182028
206.094	58.321	-0.305023649	0.192947814	-1.580860865	0.113909854	0.27132155
90.055	84.601	0.309625952	0.196159045	1.57844341	0.114463779	0.27132155
238.107	39.303	0.279368572	0.179388647	1.557336966	0.119390503	0.279548495
258.15	74.904	-0.289779995	0.187811892	-1.542926769	0.122848545	0.281642819
369.894	67.014	0.38098806	0.247169662	1.541403003	0.123218734	0.281642819
130.041	96.347	-0.231146312	0.151321417	-1.527518817	0.126632052	0.286039458
360.15	108.476	0.281445916	0.186296417	1.510742506	0.130854068	0.292139315
509.379	48.096	-0.317811425	0.211925695	-1.499636113	0.133708688	0.295081242
205.068	73.03	0.356192171	0.24022133	1.482766629	0.138136425	0.297672457
363.017	77.333	-0.248978174	0.168509871	-1.477528725	0.139533964	0.297672457
722.486	66.165	-0.284366749	0.191681369	-1.483538805	0.137931314	0.297672457

388.254	41.378	0.286891428	0.197247177	1.454476728	0.145814181	0.307651899
373.057	87.578	-0.237600876	0.168635629	-1.408960122	0.15884696	0.331506699
261.043	87.544	-0.221294983	0.160061672	-1.382560734	0.166799596	0.340697047
272.185	35.432	0.236556861	0.170888063	1.384279614	0.166272854	0.340697047
101.071	96.528	-0.250093319	0.182277347	-1.37204827	0.170048411	0.34367679
117.077	78.853	0.277473145	0.216485496	1.281717023	0.199941931	0.399883862
159.092	58.364	-0.243967259	0.195258935	-1.249455029	0.211498692	0.418636586
121.063	90.503	-0.240842743	0.195479812	-1.232059416	0.21792689	0.426958805
312.782	71.919	-0.228540107	0.19070147	-1.198418174	0.230754261	0.447523415
151.144	58.911	0.261308265	0.222492257	1.174460039	0.240210809	0.461204754
372.1	37.886	-0.225440596	0.195430316	-1.153560001	0.248680605	0.472739368
1041.685	49.43	0.254121896	0.227272648	1.118136735	0.263508599	0.486477414
193.028	89.085	0.211983996	0.187764495	1.128988714	0.258902594	0.486477414
85.052	96.569	0.248816415	0.221838745	1.121609367	0.262028567	0.486477414
670.518	68.336	-0.214486793	0.192959483	-1.111563887	0.266325707	0.486995579
148.08	94.797	-0.181965552	0.168362357	-1.080797129	0.279787366	0.503197891
176.06	99.313	-0.217214494	0.201244087	-1.079358393	0.280427991	0.503197891
131.053	97.331	-0.177528575	0.167377399	-1.060648424	0.288849707	0.504174034
173.092	90.691	-0.207860033	0.194633626	-1.067955407	0.285540627	0.504174034
551.425	36.279	-0.202365658	0.19006989	-1.06469077	0.287015888	0.504174034
151.048	94.49	-0.190275066	0.185020379	-1.028400584	0.303761434	0.516125622
199.119	92.991	0.215844337	0.209242903	1.031549145	0.302283374	0.516125622
374.734	70.26	-0.196673633	0.190611534	-1.031803425	0.302164214	0.516125622
301.118	42.143	-0.197331035	0.195146868	-1.011192424	0.311924341	0.525346259
130.077	96.602	-0.164429178	0.169624307	-0.969372731	0.332359253	0.548642831
221.07	102.078	-0.180347571	0.187265796	-0.963056652	0.335519094	0.548642831
316.776	71.543	-0.181514352	0.189128929	-0.959738702	0.33718674	0.548642831
98.058	59.679	0.227966575	0.235600836	0.96759663	0.333245861	0.548642831
164.029	86.645	-0.176672145	0.195411747	-0.904101967	0.365941335	0.590426356
212.101	91.485	0.181748487	0.203566288	0.89282213	0.371952433	0.595123893
315.161	42.821	0.162278959	0.189196203	0.857728414	0.391042447	0.6201216

365.105	100.434	0.157933413	0.185361441	0.852029482	0.394197729	0.6201216
666.523	68.093	-0.161176598	0.190400124	-0.846515196	0.3972654	0.6201216
120.003	86.774	-0.158889919	0.18949642	-0.838485071	0.401758329	0.620373714
130.136	49.642	0.193610113	0.231952966	0.83469557	0.403889136	0.620373714
127.03	48.917	-0.142276022	0.177202368	-0.80290136	0.42203175	0.630885537
149.081	97.299	-0.132195154	0.165302931	-0.799714517	0.42387622	0.630885537
428.698	68.62	-0.152518278	0.188175122	-0.810512443	0.417645716	0.630885537
556.859	81.955	-0.156573131	0.191941485	-0.815733665	0.41465248	0.630885537
1128.196	68.006	-0.139579579	0.178851403	-0.780422053	0.435142492	0.634819043
147.076	98.78	-0.128382437	0.163149527	-0.78690045	0.431340144	0.634819043
188.071	59.806	-0.151556352	0.197114478	-0.768874782	0.441967649	0.634819043
189.074	58.358	-0.151389399	0.197365319	-0.767051678	0.443050791	0.634819043
314.779	72.2	-0.145818959	0.187806249	-0.776432944	0.437493399	0.634819043
289.975	84.825	0.133997822	0.1854441	0.722577974	0.469939204	0.666545782
346.862	70.229	-0.126616702	0.176099396	-0.719007018	0.472136596	0.666545782
486.656	68.489	-0.13305905	0.186887441	-0.711974276	0.476480706	0.66776858
190.119	93.248	0.131057079	0.185562459	0.706269357	0.480020646	0.667854812
1038.657	51.615	0.153241468	0.220543474	0.69483565	0.487158304	0.668102817
250.064	59.738	-0.138910577	0.19983746	-0.695117807	0.486981476	0.668102817
249.108	93.074	0.13971294	0.21076912	0.662871963	0.50741256	0.690944762
246.955	138.586	-0.114301438	0.179389625	-0.637168608	0.524015023	0.703572618
568.426	36.345	-0.134846768	0.209840114	-0.642616728	0.520472826	0.703572618
131.111	109.554	0.122862967	0.194577645	0.631434136	0.527756702	0.703675603
101.977	134.09	0.121451308	0.202967331	0.598378601	0.549587341	0.717018502
174.05	76.594	0.129424879	0.219538941	0.589530399	0.555505526	0.717018502
238.092	127.057	0.121459808	0.20230093	0.600391743	0.548245189	0.717018502
520.12	55.829	-0.111345952	0.189317785	-0.588143118	0.556436234	0.717018502
608.565	68.642	-0.114187007	0.188965542	-0.60427423	0.545661336	0.717018502
430.696	70.699	-0.108330531	0.190675535	-0.568140692	0.569939439	0.729522482
165.033	87.089	-0.097742155	0.176027024	-0.555267895	0.578711435	0.73305988
606.568	68.596	-0.103313687	0.186861364	-0.552889508	0.580339072	0.73305988

662.53	68.617	-0.091583135	0.18883919	-0.484979496	0.62769089	0.787690529
130.05	94.746	-0.074197643	0.171869499	-0.431709197	0.665952782	0.801448685
149.077	96.666	-0.075628082	0.175186676	-0.431699966	0.665959492	0.801448685
223.985	85.258	0.08387587	0.194292383	0.43169922	0.665960034	0.801448685
265.035	59.429	0.082298804	0.191808402	0.429067773	0.667873904	0.801448685
298.101	114.873	-0.084091377	0.186852683	-0.450041046	0.652680845	0.801448685
664.526	68.434	-0.086161005	0.185871938	-0.463550367	0.642969921	0.801448685
668.521	68.55	-0.082606395	0.188532984	-0.438153546	0.661274981	0.801448685
1037.653	51.282	0.084243045	0.202480359	0.416055393	0.67736947	0.807794647
148.06	89.479	-0.073577841	0.189207238	-0.388874346	0.697369101	0.815711592
216.063	122.209	0.070696794	0.184121568	0.383968022	0.701002149	0.815711592
288.119	128.322	0.077127467	0.193647905	0.398287124	0.690418551	0.815711592
548.827	78.227	0.073014555	0.186867973	0.390728031	0.695998277	0.815711592
343.123	109.031	0.074711642	0.205062146	0.364336583	0.715606685	0.82768966
476.306	44.819	-0.066787267	0.206243065	-0.323827939	0.746068303	0.857755174
232.027	52.237	-0.0603013	0.190051095	-0.31728994	0.75102361	0.858312697
254.824	72.613	-0.054075568	0.190575432	-0.283748892	0.776602823	0.877104365
351.088	56.365	0.049804418	0.174370079	0.285624792	0.775165513	0.877104365
1047.732	48.709	0.063710252	0.242879131	0.262312583	0.793080462	0.880181784
276.119	116.59	0.051743765	0.189939822	0.272421888	0.785297645	0.880181784
313.154	42.894	0.052422942	0.19980209	0.262374343	0.793032851	0.880181784
159.084	121.116	-0.045633398	0.189998599	-0.240177549	0.810192617	0.894005647
1134.187	68.175	-0.035065946	0.189821641	-0.184731022	0.853440013	0.900332321
146.118	59.646	-0.039462782	0.210721944	-0.187274191	0.851445657	0.900332321
170.055	95.669	0.040381673	0.181780511	0.222145229	0.82420083	0.900332321
329.03	87.116	-0.038859094	0.180012213	-0.215869211	0.829089697	0.900332321
331.092	96.557	-0.034632536	0.170176005	-0.203510098	0.838736353	0.900332321
365.152	102.353	0.036775534	0.190511183	0.19303609	0.846930704	0.900332321
572.662	68.132	-0.038716823	0.188278696	-0.205635709	0.837075482	0.900332321
585.195	102.316	0.035567808	0.190792581	0.186421339	0.852114359	0.900332321
362.062	90.079	0.027676044	0.189782321	0.145830465	0.884055225	0.923827233

542.153	102.319	0.027765588	0.192535443	0.14421027	0.885334432	0.923827233
546.83	77.148	-0.022565103	0.187327418	-0.120458093	0.904120275	0.938330231
335.84	67.832	0.020722261	0.187391136	0.11058293	0.911947084	0.941364732
604.571	68.42	-0.018395464	0.187963297	-0.09786732	0.92203765	0.946691063
314.158	42.873	-0.01462212	0.190822889	-0.076626658	0.938920552	0.958897585
189.123	85.138	-0.011881724	0.190318918	-0.062430601	0.950219927	0.965302783
169.059	96.539	0.009417546	0.176313731	0.053413572	0.957402392	0.966554791
277.953	141.319	-0.01025317	0.212521099	-0.048245421	0.961520651	0.966554791
150.084	96.708	0.003741933	0.178486591	0.020964786	0.983273746	0.983273746

Supplementary Table 2 Continued. Replication of MWAS results from PD participants in non-PD participants.						
C18						
mz	rt	beta	se	zvalue	pvalue	FDR_pvalue
143.035	36.297	1.158642709	0.221102907	5.240287083	1.60327E-07	7.94178E-06
215.033	30.913	1.286754283	0.249718229	5.152824808	2.56592E-07	7.94178E-06
217.03	30.51	1.264464647	0.246075724	5.138518454	2.76913E-07	7.94178E-06
225.062	37.895	1.153088961	0.225021431	5.124351746	2.98563E-07	7.94178E-06
359.121	49.326	1.801304688	0.344189099	5.233473957	1.66353E-07	7.94178E-06
226.065	36.217	1.18881357	0.235866333	5.040200331	4.65045E-07	1.03085E-05
218.033	30.739	1.423278088	0.284990827	4.99411894	5.9105E-07	1.12299E-05
219.034	30.853	1.744425193	0.351757683	4.959167284	7.0796E-07	1.17698E-05
239.077	35.736	1.113519287	0.228295026	4.877545106	1.07414E-06	1.58734E-05
113.024	36.458	0.986794113	0.205951374	4.791393677	1.65627E-06	1.69449E-05
179.056	36.803	1.030946039	0.21400433	4.817407381	1.45436E-06	1.69449E-05
215.091	31.459	1.135502352	0.236720746	4.796801171	1.61219E-06	1.69449E-05
216.035	31.278	1.288011993	0.268471926	4.79756678	1.60605E-06	1.69449E-05
180.06	36.11	1.059370812	0.222639022	4.758244101	1.95284E-06	1.8552E-05
209.067	36.808	-0.862730171	0.194144735	-4.443747454	8.84054E-06	7.83861E-05
199.038	31.744	-0.826212973	0.190738792	-4.33164625	1.47999E-05	0.000123024
269.088	35.076	0.939267232	0.226040322	4.155308315	3.24849E-05	0.000254147
270.091	34.705	1.095487198	0.265540509	4.125499352	3.69931E-05	0.000273338
200.041	32.191	-0.61280285	0.152201791	-4.026252565	5.66728E-05	0.000376874
216.971	30.784	1.186932477	0.29405152	4.036477952	5.42596E-05	0.000376874
174.088	30.043	-0.731119962	0.189598038	-3.856157846	0.000115183	0.000729493
220.049	30.642	-0.714035026	0.19733691	-3.618355165	0.000296481	0.001714436
223.082	38.976	-0.712507646	0.196557201	-3.624937901	0.000289031	0.001714436
221.053	30.668	-0.700870295	0.196120595	-3.573670044	0.000352012	0.001872706
223.05	30.675	-0.702326304	0.196426608	-3.575515105	0.000349539	0.001872706
253.093	34.817	-0.504826823	0.152370014	-3.313163856	0.000922469	0.004718785
127.051	32.393	-0.656116629	0.2019048	-3.249633632	0.001155538	0.005692093
402.293	232.497	0.687163038	0.220255872	3.119839808	0.001809494	0.008595097

225.054	30.806	-0.564003679	0.185304954	-3.043651383	0.002337258	0.010719149
286.06	27.608	0.825992702	0.273187038	3.0235428	0.002498337	0.01107596
401.291	235.525	0.58497407	0.202239294	2.892484732	0.003822078	0.016397946
425.291	227.677	0.508199702	0.185573923	2.738529715	0.006171458	0.025650124
415.306	235.89	0.553976301	0.203126125	2.727252834	0.006386408	0.025739161
161.987	32.772	0.503419545	0.187968498	2.678212306	0.007401629	0.02895343
189.067	63.388	-0.45235488	0.171069132	-2.644281148	0.008186463	0.031108558
416.309	235.341	0.549280679	0.210439693	2.610157201	0.009050062	0.033434953
148.98	33.213	0.500024148	0.201348462	2.48337705	0.013014325	0.046323704
90.028	31.729	0.467259708	0.188610951	2.477373167	0.013235344	0.046323704
89.024	31.295	0.451731228	0.187752148	2.405997663	0.016128368	0.05500187
415.322	215.543	-0.439903869	0.190080599	-2.314301781	0.020651174	0.068665154
89.009	34.171	0.424466131	0.187991227	2.257903936	0.023951649	0.077696813
118.051	31.331	-0.447310706	0.202694184	-2.206825558	0.027326249	0.086488843
439.306	227.232	0.498408845	0.226775088	2.197811268	0.027962558	0.086488843
89.04	33.444	0.399082187	0.188291257	2.119493987	0.034048741	0.102920059
268.96	35.981	-0.393939731	0.193737112	-2.033372576	0.042014896	0.124177359
412.278	210.722	0.396005612	0.196016321	2.020268569	0.043355537	0.125354053
494.81	33.188	-0.320394438	0.161617192	-1.982427944	0.047431365	0.13422067
103.04	32.211	0.363372437	0.184190022	1.972812816	0.048516892	0.134432223
445.988	31.336	-0.368537342	0.188025419	-1.960040001	0.049991115	0.135690169
369.154	47.826	-0.372986775	0.193089686	-1.931676326	0.053399469	0.142042586
387.275	216.137	0.454166897	0.238440736	1.904737021	0.056814264	0.148162689
414.204	35.694	0.388123782	0.207376317	1.871591646	0.06126312	0.156692211
397.259	210.47	0.362136038	0.198454974	1.824776824	0.068034715	0.170728623
229.144	107.471	-0.289380608	0.16251851	-1.780600918	0.07497767	0.184667224
503.631	32.36	-0.262177408	0.151842908	-1.72663585	0.084233083	0.20369091
501.634	32.333	-0.261415045	0.153087242	-1.707621368	0.087706621	0.208303225
145.062	30.621	-0.27409575	0.173849456	-1.576627016	0.114881373	0.268056536
216.909	24.258	-0.275850056	0.182956066	-1.507739326	0.131621256	0.301821157
146.065	30.227	-0.205807107	0.144801802	-1.421302119	0.155228948	0.324606901

158.061	87.22	-0.280227836	0.194800765	-1.438535603	0.150282143	0.324606901
217.048	30.99	0.31443275	0.220869685	1.423612074	0.154558799	0.324606901
373.26	213.573	0.376854257	0.259712351	1.451044801	0.146767386	0.324606901
423.275	218.074	0.322330802	0.227319751	1.417962144	0.156201817	0.324606901
424.247	181.74	-0.253708065	0.178056014	-1.424877825	0.154192521	0.324606901
418.95	37.272	-0.227999902	0.166099073	-1.372674139	0.169853672	0.347546745
322.932	37.944	-0.243771014	0.18203441	-1.33914799	0.1805225	0.36378019
431.244	82.667	0.230549501	0.17964059	1.283393144	0.199354375	0.395733311
261.041	34.747	-0.211594861	0.16987956	-1.245558096	0.212926674	0.416459525
195.051	31.406	0.223204251	0.181858171	1.227353439	0.219689779	0.417410579
279.614	29.271	-0.246326336	0.199805399	-1.232831233	0.217638736	0.417410579
194.039	27.536	-0.248306582	0.204736141	-1.212812653	0.225201457	0.42185625
295.984	32.846	-0.210219608	0.176945611	-1.188046473	0.234815101	0.433755673
266.963	36.467	-0.214296006	0.186730609	-1.147621204	0.251124972	0.457529059
213.049	35.964	-0.183029729	0.164867373	-1.110163434	0.266928606	0.467125061
324.921	34.963	-0.188112658	0.167500034	-1.123060421	0.261411834	0.467125061
403.296	242.279	0.224142983	0.201423579	1.112794161	0.265796844	0.467125061
177.04	33.332	0.213404254	0.198752502	1.07371858	0.282948831	0.488729799
255.63	33.175	0.239561293	0.228668758	1.047634561	0.294807008	0.502683744
424.278	219.234	0.197071136	0.19041497	1.034956108	0.30068941	0.506223943
195.976	28.201	-0.169048848	0.181601644	-0.9308773	0.351917038	0.585062075
203.021	32.471	-0.141787601	0.15613931	-0.908083944	0.363833873	0.597406235
300.877	34.139	-0.142343621	0.169849722	-0.838056249	0.401999113	0.644167253
564.083	212.912	0.176481648	0.208944074	0.844635815	0.398314206	0.644167253
96.922	31.526	-0.149759238	0.187672783	-0.797980588	0.424881755	0.672729445
162.056	40.608	-0.136493527	0.191863063	-0.711411174	0.476829477	0.734943265
224.952	56.909	0.132344647	0.194998205	0.678696745	0.497330029	0.734943265
416.953	38.484	-0.113884249	0.167648446	-0.679303935	0.496945304	0.734943265
581.24	42.898	-0.145732022	0.202081632	-0.721154221	0.470814634	0.734943265
94.908	31.854	-0.124550569	0.181908271	-0.684688874	0.493540271	0.734943265
94.942	31.882	-0.126648274	0.180974451	-0.699813	0.484044095	0.734943265

508.623	32.501	0.128481748	0.195526567	0.657106349	0.511112537	0.747010631
151.007	30.177	0.13470969	0.209462795	0.643119893	0.520146307	0.75195064
254.982	28.027	0.122781274	0.1992759	0.616137096	0.537804041	0.769117606
254.623	31.407	-0.102473312	0.17992153	-0.569544471	0.5689867	0.80505565
326.087	28.546	-0.101209106	0.191247182	-0.529205737	0.596662737	0.829728669
498.638	32.269	0.114476571	0.217644219	0.525980298	0.598901896	0.829728669
566.582	32.607	-0.089409984	0.173998464	-0.513855021	0.607353357	0.83276285
496.807	33.723	-0.093169966	0.192956701	-0.482854264	0.629199218	0.853913224
114.056	36.877	0.088026081	0.191460204	0.459761765	0.645687231	0.867438401
260.023	28.879	-0.089052882	0.20219291	-0.440435239	0.659621907	0.877297137
440.679	32.243	-0.074957743	0.177991259	-0.421131594	0.673658992	0.887095504
311.14	35.236	-0.070770281	0.176624417	-0.400682318	0.68865403	0.889323274
446.67	32.191	-0.07120577	0.177753464	-0.400587244	0.688724039	0.889323274
312.143	35.633	-0.073069088	0.187610932	-0.389471379	0.696927479	0.891263026
415.216	36.104	0.066516417	0.190771342	0.348670906	0.727336389	0.921292759
374.263	214.626	0.065808169	0.201408574	0.326739659	0.743864801	0.933339798
256.934	34.638	-0.056041966	0.184913645	-0.303071017	0.761835741	0.934565086
302.874	34.257	-0.053280933	0.184669679	-0.288520203	0.772948568	0.934565086
444.673	32.115	-0.056822836	0.180394595	-0.314991899	0.752767805	0.934565086
562.588	32.428	0.062237029	0.215215415	0.28918481	0.772439955	0.934565086
149.061	32.211	0.049133794	0.191767452	0.2562155	0.797784441	0.949099741
163.077	35.967	-0.03525534	0.184174585	-0.191423482	0.848193824	0.949099741
188.057	31.438	0.035848615	0.186691121	0.192020996	0.847725759	0.949099741
276	31.551	-0.0355619	0.187137499	-0.19003086	0.849284948	0.949099741
282.975	35.721	-0.036261065	0.18014746	-0.201285461	0.84047537	0.949099741
390.709	32.22	-0.033499975	0.185034571	-0.181047116	0.856330594	0.949099741
406.925	35.575	0.048022563	0.18883843	0.254305031	0.799259913	0.949099741
505.628	32.397	0.041008251	0.195677534	0.209570565	0.834002855	0.949099741
506.626	32.343	-0.03992106	0.185189885	-0.215568252	0.829324306	0.949099741
556.597	32.309	0.04368589	0.203986766	0.214160414	0.830421965	0.949099741
160.062	29.915	0.029853528	0.199160343	0.149896952	0.880845916	0.952459405

193.123	84.259	0.028640683	0.183533682	0.156051375	0.875992526	0.952459405
281.124	29.849	0.032136546	0.19153484	0.167784336	0.866752946	0.952459405
144.97	29.085	0.014894509	0.189465791	0.078613183	0.937340301	0.958971231
149.046	31.935	0.02160626	0.207684602	0.104034002	0.917142365	0.958971231
156.878	31.938	0.021814298	0.197960425	0.110195246	0.912254532	0.958971231
226.018	34.225	-0.022569412	0.193312587	-0.116750867	0.90705748	0.958971231
229.012	27.859	-0.016145245	0.188319177	-0.085733412	0.931678341	0.958971231
442.676	32.194	-0.021485441	0.188985873	-0.113688083	0.90948506	0.958971231
452.705	33.294	-0.018802889	0.198753394	-0.094604117	0.924629279	0.958971231
280.622	30.778	0.007060544	0.201326362	0.035070139	0.972023812	0.98686387
356.098	28.295	-0.004205236	0.186162096	-0.022589108	0.981978032	0.989417259
327.091	28.264	-0.000906807	0.181015339	-0.005009557	0.996002968	0.996002968

Supplementary Table 3. Annotation of metabolites based on in-house library.

							PD Patients					non-PD Participants				
**Chemical name**	**Observed mass to charge ratio**	**Observed retention time**	**Retention time difference**	**Mass error**	**indicator of match**	**column**	**Beta**	**se**	**zvalue**	**p value**	**FDR p value**	**Beta**	**se**	**zvalue**	**p value**	**FDR p value**
Citrulline	174.0883984	30.04349642	8.44349642	2.920358834	unique	C18	-0.62	0.12	-5.24	**0.00**	**0.00**	-0.73	0.19	-3.86	**0.00**	**0.01**
myo-Inositol	179.0562404	36.80263892	15.20263892	3.632492607	multiple	C18	0.56	0.11	5.20	**0.00**	**0.00**	1.03	0.21	4.82	**0.00**	**0.00**
Allose	179.0562404	36.80263892	15.20263892	3.632492607	multiple	C18	0.56	0.11	5.20	**0.00**	**0.00**	1.03	0.21	4.82	**0.00**	**0.00**
Sorbitol	179.0562404	36.80263892	15.20263892	3.632492607	multiple	C18	0.56	0.11	5.20	**0.00**	**0.00**	1.03	0.21	4.82	**0.00**	**0.00**
D-Fructose	179.0562404	36.80263892	15.20263892	3.632492607	multiple	C18	0.56	0.11	5.20	**0.00**	**0.00**	1.03	0.21	4.82	**0.00**	**0.00**
L-Sorbose	179.0562404	36.80263892	15.20263892	3.632492607	multiple	C18	0.56	0.11	5.20	**0.00**	**0.00**	1.03	0.21	4.82	**0.00**	**0.00**
Alpha-D-Glucose	179.0562404	36.80263892	15.20263892	3.632492607	multiple	C18	0.56	0.11	5.20	**0.00**	**0.00**	1.03	0.21	4.82	**0.00**	**0.00**
D-Galactose	179.0562404	36.80263892	15.20263892	3.632492607	multiple	C18	0.56	0.11	5.20	**0.00**	**0.00**	1.03	0.21	4.82	**0.00**	**0.00**
D-Tagatose	179.0562404	36.80263892	15.20263892	3.632492607	multiple	C18	0.56	0.11	5.20	**0.00**	**0.00**	1.03	0.21	4.82	**0.00**	**0.00**
D-Mannose	179.0562404	36.80263892	15.20263892	3.632492607	multiple	C18	0.56	0.11	5.20	**0.00**	**0.00**	1.03	0.21	4.82	**0.00**	**0.00**
L-Gulonolactone	177.0404706	33.33171737	11.73171737	2.997275039	unique	C18	0.55	0.12	4.51	**0.00**	**0.00**	0.21	0.20	1.07	0.28	0.79
Glyceraldehyde	89.02442221	31.29479075	8.394790746	5.865998845	multiple	C18	0.45	0.11	4.07	**0.00**	**0.00**	0.45	0.19	2.41	**0.02**	0.33
L-Lactic acid	89.02442221	31.29479075	9.094790746	5.865998845	multiple	C18	0.45	0.11	4.07	**0.00**	**0.00**	0.45	0.19	2.41	**0.02**	0.33
Citrulline	176.1029855	102.3720203	11.17202035	-2.864696551	unique	HILIC	-0.49	0.12	-3.96	**0.00**	**0.00**	-0.75	0.20	-3.75	**0.00**	**0.01**
Indoleacetaldehyde	158.0611687	87.21980259	27.21980259	3.534862247	unique	C18	-0.50	0.13	-3.95	**0.00**	**0.00**	-0.28	0.19	-1.44	0.15	0.65
beta-Alanine	90.05497123	84.60116104	13.60116104	-5.649501024	multiple	HILIC	0.42	0.12	3.59	**0.00**	**0.01**	0.31	0.20	1.58	0.11	0.53
D-Alanine	90.05497123	84.60116104	12.30116104	-5.649501024	multiple	HILIC	0.42	0.12	3.59	**0.00**	**0.01**	0.31	0.20	1.58	0.11	0.53
Sarcosine	90.05497123	84.60116104	13.60116104	-5.649501024	multiple	HILIC	0.42	0.12	3.59	**0.00**	**0.01**	0.31	0.20	1.58	0.11	0.53
L-Alanine	90.05497123	84.60116104	25.60116104	-5.649501024	multiple	HILIC	0.42	0.12	3.59	**0.00**	**0.01**	0.31	0.20	1.58	0.11	0.53
(R)-3-Hydroxybutyric acid	103.0400643	32.21065915	9.410659154	4.991041197	multiple	C18	0.40	0.11	3.63	**0.00**	**0.01**	0.36	0.18	1.97	**0.05**	0.45
Alpha-Hydroxyisobutyric acid	103.0400643	32.21065915	8.110659154	4.991041197	multiple	C18	0.40	0.11	3.63	**0.00**	**0.01**	0.36	0.18	1.97	**0.05**	0.45
2-Hydroxybutyric acid	103.0400643	32.21065915	9.410659154	5.088091804	multiple	C18	0.40	0.11	3.63	**0.00**	**0.01**	0.36	0.18	1.97	**0.05**	0.45
L-Proline	114.0560525	36.87677207	10.97677207	4.581191706	unique	C18	0.39	0.11	3.50	**0.00**	**0.02**	0.09	0.19	0.46	0.65	0.96
L-Glutamic acid	148.0604207	89.47885317	9.478853173	0.139657903	unique	HILIC	0.39	0.11	3.37	**0.00**	**0.02**	-0.07	0.19	-0.39	0.70	0.93
Biliverdin	581.2403432	42.89769953	-23.70230047	0.521706737	unique	C18	-0.35	0.10	-3.30	**0.00**	**0.02**	-0.15	0.20	-0.72	0.47	0.91
3-Methyldioxyindole	162.0560548	40.60765598	8.207655978	0.189945802	unique	C18	-0.39	0.13	-3.08	**0.00**	**0.04**	-0.14	0.19	-0.71	0.48	0.91
Mannitol	181.0713628	37.30573595	15.10573595	0.678094594	multiple	C18	0.32	0.11	2.96	**0.00**	0.05	0.26	0.18	1.45	0.15	0.65

Galactitol	181.0713628	37.30573595	15.10573595	0.678094594	multiple	C18	0.32	0.11	2.96	**0.00**	0.05	0.26	0.18	1.45	0.15	0.65
Uridine	243.0618807	33.43868816	9.038688157	0.578830066	unique	C18	0.32	0.12	2.74	**0.01**	0.08	0.03	0.19	0.18	0.86	0.99
L-Fucose	163.0612494	33.32490539	-5.175094605	3.491772486	multiple	C18	-0.29	0.11	-2.65	**0.01**	0.09	-0.52	0.17	-2.99	**0.00**	0.13
Rhamnose	163.0612494	33.32490539	11.72490539	0.155590614	multiple	C18	-0.29	0.11	-2.65	**0.01**	0.09	-0.52	0.17	-2.99	**0.00**	0.13
Hypoxanthine	137.0458318	56.41162292	12.21162292	-3.489278803	unique	HILIC	-0.28	0.11	-2.49	**0.01**	0.12	0.16	0.21	0.78	0.44	0.83
L-Aspartic acid	134.0447799	102.0826772	16.88267722	-3.954543773	multiple	HILIC	0.31	0.13	2.43	**0.01**	0.13	-0.02	0.19	-0.12	0.90	0.99
D-Aspartic acid	134.0447799	102.0826772	14.78267722	-3.954543773	multiple	HILIC	0.31	0.13	2.43	**0.01**	0.13	-0.02	0.19	-0.12	0.90	0.99
L-Serine	104.03532	30.10058958	7.400589581	5.094881871	unique	C18	-0.29	0.12	-2.43	**0.01**	0.13	-0.47	0.20	-2.33	**0.02**	0.37
Sarcosine	88.04039361	36.44153073	11.74153073	5.833797609	multiple	C18	0.26	0.11	2.39	**0.02**	0.14	0.16	0.19	0.85	0.40	0.87
L-Alanine	88.04039361	36.44153073	10.84153073	5.833797609	multiple	C18	0.26	0.11	2.39	**0.02**	0.14	0.16	0.19	0.85	0.40	0.87
D-Alanine	88.04039361	36.44153073	12.44153073	5.833797609	multiple	C18	0.26	0.11	2.39	**0.02**	0.14	0.16	0.19	0.85	0.40	0.87
beta-Alanine	88.04039361	36.44153073	11.74153073	5.833797609	multiple	C18	0.26	0.11	2.39	**0.02**	0.14	0.16	0.19	0.85	0.40	0.87
Oxoglutaric acid	145.0143277	28.16391522	7.163915217	4.121888758	unique	C18	0.26	0.12	2.30	**0.02**	0.17	0.27	0.19	1.40	0.16	0.67
trans-Aconitic acid	173.0089454	29.36718149	10.06718149	1.765296493	unique	C18	0.29	0.13	2.29	**0.02**	0.17	0.06	0.20	0.29	0.78	0.99
Pantothenic acid	220.1179138	41.47633633	5.876336325	-2.526702836	unique	HILIC	0.27	0.12	2.28	**0.02**	0.17	0.11	0.19	0.59	0.56	0.88
L-Threonine	120.0655333	87.2319273	10.6319273	-4.220013611	multiple	HILIC	-0.26	0.12	-2.21	**0.03**	0.18	-0.11	0.19	-0.60	0.55	0.87
L-Homoserine	120.0655333	87.2319273	10.5319273	-4.220013611	multiple	HILIC	-0.26	0.12	-2.21	**0.03**	0.18	-0.11	0.19	-0.60	0.55	0.87
L-Allothreonine	120.0655333	87.2319273	11.5319273	-4.220013611	multiple	HILIC	-0.26	0.12	-2.21	**0.03**	0.18	-0.11	0.19	-0.60	0.55	0.87
gamma-Aminobutyric acid	102.0560374	29.52858456	9.128584556	4.971404891	multiple	C18	0.25	0.12	2.16	**0.03**	0.20	0.09	0.19	0.49	0.62	0.95
gamma-Aminobutyric acid	102.0560374	29.52858456	9.128584556	4.971404891	multiple	C18	0.25	0.12	2.16	**0.03**	0.20	0.09	0.19	0.49	0.62	0.95
2-Aminoisobutyric acid	102.0560374	29.52858456	4.928584556	4.971404891	multiple	C18	0.25	0.12	2.16	**0.03**	0.20	0.09	0.19	0.49	0.62	0.95
3-Aminoisobutanoic acid	102.0560374	29.52858456	9.628584556	4.971404891	multiple	C18	0.25	0.12	2.16	**0.03**	0.20	0.09	0.19	0.49	0.62	0.95
Mevalonic acid	147.0662787	27.0178076	4.817807597	-0.308009126	unique	C18	0.24	0.11	2.13	**0.03**	0.21	0.07	0.18	0.37	0.71	0.98
L-Cystathionine	223.07462	221.4494019	24.0494019	-2.734585261	unique	HILIC	0.25	0.12	2.01	**0.04**	0.25	0.16	0.20	0.82	0.41	0.82
N-Acetylneuraminic acid	308.0984287	30.99515515	11.19515515	0.807068456	unique	C18	0.21	0.11	1.96	0.05	0.27	0.38	0.18	2.08	**0.04**	0.44
L-Isoleucine	130.0873514	37.54855684	12.54855684	4.00781455	multiple	C18	0.21	0.11	1.86	0.06	0.31	0.11	0.20	0.58	0.56	0.94
L-Norleucine	130.0873514	37.54855684	12.84855684	4.00781455	multiple	C18	0.21	0.11	1.86	0.06	0.31	0.11	0.20	0.58	0.56	0.94
L-Leucine	130.0873514	37.54855684	12.04855684	4.00781455	multiple	C18	0.21	0.11	1.86	0.06	0.31	0.11	0.20	0.58	0.56	0.94
D-Aspartic acid	132.0302219	28.86114517	8.96114517	3.877310554	unique	C18	0.21	0.11	1.83	0.07	0.32	-0.16	0.19	-0.86	0.39	0.87
Arachidic acid	311.2953022	284.8771108	-7.322889218	0.874420307	unique	C18	-0.20	0.11	-1.81	0.07	0.33	0.23	0.19	1.20	0.23	0.74
L-Norleucine	132.1019301	61.1847716	14.2847716	-3.783834457	multiple	HILIC	0.19	0.11	1.74	0.08	0.35	0.30	0.21	1.46	0.14	0.58
L-Leucine	132.1019301	61.1847716	14.3847716	-3.783834457	multiple	HILIC	0.19	0.11	1.74	0.08	0.35	0.30	0.21	1.46	0.14	0.58

L-Isoleucine	132.1019301	61.1847716	15.0847716	-3.783834457	multiple	HILIC	0.19	0.11	1.74	0.08	0.35	0.30	0.21	1.46	0.14	0.58
2-Pyrocatechuic acid	153.0193633	33.4620323	9.462032297	3.616067858	multiple	C18	0.18	0.12	1.55	0.12	0.44	0.22	0.20	1.07	0.29	0.79
Protocatechuic acid	153.0193633	33.4620323	9.462032297	3.616067858	multiple	C18	0.18	0.12	1.55	0.12	0.44	0.22	0.20	1.07	0.29	0.79
Gentisic acid	153.0193633	33.4620323	9.462032297	3.616067858	multiple	C18	0.18	0.12	1.55	0.12	0.44	0.22	0.20	1.07	0.29	0.79
Creatine	132.0767076	83.6448874	28.4448874	-4.334064436	unique	HILIC	0.18	0.12	1.48	0.14	0.46	0.03	0.21	0.14	0.89	0.98
L-Lysine	147.1128016	112.896452	7.896452026	-3.591551557	multiple	HILIC	0.19	0.13	1.47	0.14	0.47	0.09	0.19	0.49	0.63	0.90
L-Lysine	147.1128016	112.896452	9.296452026	-3.591551557	multiple	HILIC	0.19	0.13	1.47	0.14	0.47	0.09	0.19	0.49	0.63	0.90
L-Histidine	154.062263	38.67651646	15.67651646	3.829921713	multiple	C18	-0.17	0.12	-1.46	0.14	0.48	-0.75	0.24	-3.09	**0.00**	0.10
L-Histidine	154.062263	38.67651646	15.27651646	3.784485191	multiple	C18	-0.17	0.12	-1.46	0.14	0.48	-0.75	0.24	-3.09	**0.00**	0.10
Uric acid	169.0356148	79.72296921	19.22296921	-3.106991457	unique	HILIC	0.16	0.11	1.37	0.17	0.52	0.12	0.20	0.63	0.53	0.87
Indoleacetic acid	176.0706182	36.86039473	7.560394726	-2.906790182	multiple	HILIC	0.15	0.11	1.28	0.20	0.55	0.05	0.20	0.26	0.80	0.96
Indoleacetic acid	176.0706182	36.86039473	8.060394726	-2.906790182	multiple	HILIC	0.15	0.11	1.28	0.20	0.55	0.05	0.20	0.26	0.80	0.96
trans-Cinnamic acid	147.0450025	35.14218792	5.142187924	2.533451745	unique	C18	-0.14	0.11	-1.25	0.21	0.57	0.25	0.20	1.25	0.21	0.73
Phenylpyruvic acid	165.0546551	72.20503329	13.00503329	-2.998203291	unique	HILIC	-0.14	0.12	-1.21	0.23	0.58	-0.41	0.20	-2.09	**0.04**	0.33
Uric acid	167.0209701	30.00882789	10.00882789	2.574972893	unique	C18	0.15	0.12	1.22	0.22	0.59	0.00	0.21	0.00	1.00	1.00
gamma-Aminobutyric acid	104.0705693	76.94815301	6.748153009	-5.387589041	multiple	HILIC	0.13	0.11	1.18	0.24	0.59	0.22	0.18	1.19	0.24	0.70
2-Aminoisobutyric acid	104.0705693	76.94815301	6.748153009	-5.387589041	multiple	HILIC	0.13	0.11	1.18	0.24	0.59	0.22	0.18	1.19	0.24	0.70
gamma-Aminobutyric acid	104.0705693	76.94815301	6.748153009	-5.387589041	multiple	HILIC	0.13	0.11	1.18	0.24	0.59	0.22	0.18	1.19	0.24	0.70
3-Aminoisobutanoic acid	104.0705693	76.94815301	6.448153009	-5.387589041	multiple	HILIC	0.13	0.11	1.18	0.24	0.59	0.22	0.18	1.19	0.24	0.70
all-trans-Retinoic acid	299.2013998	200.4141564	-25.18584362	0.901583724	unique	C18	0.14	0.12	1.20	0.23	0.59	0.02	0.19	0.11	0.91	0.99
Stearic acid	283.2641592	264.6434092	-17.35659078	1.515019648	unique	C18	0.13	0.11	1.17	0.24	0.61	0.44	0.19	2.33	**0.02**	0.37
Xanthine	151.0261555	32.67231243	7.772312434	3.479776416	unique	C18	0.11	0.10	1.10	0.27	0.64	0.20	0.17	1.20	0.23	0.75
Pantothenic acid	218.1032084	30.19670271	7.09670271	1.551627436	unique	C18	0.11	0.11	1.01	0.31	0.67	0.17	0.18	0.95	0.34	0.84
L-Arginine	175.1189479	109.2300623	-8.769937708	-3.032818802	unique	HILIC	-0.11	0.12	-0.96	0.34	0.69	-0.04	0.18	-0.22	0.83	0.97
O-Phosphoethanolamine	140.0106943	27.42395439	-21.57604561	-4.326299984	unique	C18	-0.09	0.10	-0.91	0.36	0.70	-0.42	0.17	-2.52	**0.01**	0.30
Gamma-Linolenic acid	277.2171819	210.2480655	-12.95193451	1.44984892	unique	C18	0.11	0.12	0.89	0.37	0.70	0.36	0.19	1.89	0.06	0.48
Pyrrolidonecarboxylic acid	128.0353433	30.63031114	9.630311139	4.321120974	multiple	C18	-0.10	0.12	-0.88	0.38	0.70	-0.14	0.18	-0.79	0.43	0.89
Pyroglutamic acid	128.0353433	30.63031114	10.03031114	4.321120974	multiple	C18	-0.10	0.12	-0.88	0.38	0.70	-0.14	0.18	-0.79	0.43	0.89
Petroselinic acid	281.2484662	242.6443551	-14.75564486	1.373324582	multiple	C18	0.10	0.12	0.85	0.40	0.71	0.41	0.20	2.02	**0.04**	0.44
Oleic acid	281.2484662	242.6443551	-15.35564486	1.373324582	multiple	C18	0.10	0.12	0.85	0.40	0.71	0.41	0.20	2.02	**0.04**	0.44
Elaidic acid	281.2484662	242.6443551	-14.15564486	1.373324582	multiple	C18	0.10	0.12	0.85	0.40	0.71	0.41	0.20	2.02	**0.04**	0.44
Kynurenic acid	190.0498778	30.04931881	-18.05068119	-2.69498885	unique	HILIC	0.10	0.12	0.83	0.40	0.74	-0.23	0.22	-1.01	0.31	0.77

Homocysteine	136.042706	74.27796678	15.27796678	-3.631228338	unique	HILIC	0.09	0.12	0.80	0.42	0.75	0.15	0.19	0.76	0.45	0.83
Taurine	126.0219624	79.83584075	17.33584075	-4.027695196	unique	HILIC	-0.08	0.12	-0.68	0.50	0.80	-0.13	0.20	-0.64	0.52	0.86
L-Serine	106.0498786	98.4517137	11.4517137	-4.821850679	unique	HILIC	-0.07	0.12	-0.60	0.55	0.83	-0.18	0.19	-0.95	0.34	0.78
Benzylamine	108.0807628	30.71595471	0.015954711	-4.969912603	unique	HILIC	-0.06	0.12	-0.55	0.58	0.85	-0.50	0.22	-2.25	**0.02**	0.27
Indoleacetic acid	174.0559764	34.41570572	8.615705722	2.564652887	multiple	C18	-0.05	0.11	-0.46	0.65	0.86	0.03	0.19	0.17	0.87	0.99
Indoleacetic acid	174.0559764	34.41570572	7.415705722	2.564652887	multiple	C18	-0.05	0.11	-0.46	0.65	0.86	0.03	0.19	0.17	0.87	0.99
Itaconic acid	129.0192106	33.81768844	11.61768844	3.105261868	multiple	C18	-0.05	0.12	-0.42	0.68	0.87	-0.32	0.20	-1.58	0.11	0.60
Citraconic acid	129.0192106	33.81768844	12.21768844	3.105261868	multiple	C18	-0.05	0.12	-0.42	0.68	0.87	-0.32	0.20	-1.58	0.11	0.60
L-Histidine	156.0767718	113.890548	12.89054798	-3.211439061	multiple	HILIC	0.04	0.12	0.35	0.73	0.91	-0.02	0.19	-0.12	0.90	0.99
L-Histidine	156.0767718	113.890548	15.59054798	-3.256288494	multiple	HILIC	0.04	0.12	0.35	0.73	0.91	-0.02	0.19	-0.12	0.90	0.99
Palmitoleic acid	253.2172518	217.5338764	-10.46612363	1.863352251	unique	C18	0.03	0.12	0.27	0.79	0.93	0.08	0.19	0.41	0.68	0.97
Phenylpyruvic acid	163.0401038	23.50978849	-1.090211509	3.396986792	multiple	C18	0.02	0.12	0.22	0.83	0.95	0.15	0.19	0.80	0.42	0.89
4-Hydroxycinnamic acid	163.0401038	23.50978849	-1.690211509	3.396986792	multiple	C18	0.02	0.12	0.22	0.83	0.95	0.15	0.19	0.80	0.42	0.89
Gamma-Linolenic acid	279.2316106	35.51712489	9.517124895	-2.755349564	unique	HILIC	-0.02	0.12	-0.14	0.89	0.96	0.12	0.19	0.62	0.54	0.87
3-Methoxytyrosine	210.0771914	35.97784078	11.37784078	2.339355497	unique	C18	-0.02	0.11	-0.15	0.88	0.97	0.08	0.21	0.37	0.71	0.98
Trigonelline	138.0549764	77.0976013	9.497601303	-3.648016649	unique	HILIC	0.01	0.12	0.05	0.96	0.99	0.24	0.22	1.09	0.28	0.74
Carnosine	227.1137191	96.45635241	-10.84364759	-2.954103291	unique	HILIC	0.01	0.12	0.05	0.96	0.99	-0.01	0.21	-0.03	0.98	1.00
Cinnamaldehyde	133.0648295	22.80858635	-4.791413653	-3.686092879	unique	HILIC	0.00	0.12	0.03	0.97	0.99	0.03	0.19	0.13	0.89	0.98

Supplementary Table 4. Annotation of metabolites based on xMSannotator.

																PD Patients					non-PD Participants				
**Chemical name**	**Observed mass to charge ratio**	**Observed retention time**	**chemical_ID**	**Indicator of match**	**Retention time difference**	**Mass error**	**Confidence**	**delta ppm**	**Formula**	**Monoisotopic Mass**	**Adduct**	**IS group**	**mean_int_vec**	**MD**	**column**	**Beta**	**se**	**z value**	**p value**	**FDR p value**	**Beta**	**se**	**z value**	**p value**	**FDR p value**
Dihydro-4-mercapto-3(2H)-furanone	119.01614	74.06905366	HMDB39786	unique	-0.030946341	0.000170015	3	0.08	C4H6O2S	118.0088501	M+H	ISgroup_20_1_2	23.9	0.016	HILIC	0.04	0.11	0.39	**0.69**	0.89	0.09	0.19	0.46	0.65	0.91
246-Octatriyn-1-ol	119.0491796	71.59850711	HMDB30968	multiple	-0.001492886	-0.003491658	3	0.34	C8H6O	118.0418648	M+H	ISgroup_17_1_5	24.1	0.049	HILIC	-0.15	0.12	-1.30	**0.19**	0.54	-0.40	0.19	-2.03	**0.04**	0.34
357-Octatriyn-1-ol	119.0491796	71.59850711	HMDB38998	multiple	-0.001492886	-0.003491658	3	0.34	C8H6O	118.0418648	M+H	ISgroup_17_1_5	24.1	0.049	HILIC	-0.15	0.12	-1.30	**0.19**	0.54	-0.40	0.19	-2.03	**0.04**	0.34
Benzofuran	119.0491796	71.59850711	HMDB32929	multiple	-0.001492886	-0.003491658	3	0.34	C8H6O	118.0418648	M+H	ISgroup_17_1_5	24.1	0.049	HILIC	-0.15	0.12	-1.30	**0.19**	0.54	-0.40	0.19	-2.03	**0.04**	0.34
xi-23-Octadiene-57-diyn-1-ol	119.0491796	71.59850711	HMDB40549	multiple	-0.001492886	-0.003491658	3	0.34	C8H6O	118.0418648	M+H	ISgroup_17_1_5	24.1	0.049	HILIC	-0.15	0.12	-1.30	**0.19**	0.54	-0.40	0.19	-2.03	**0.04**	0.34
3-Mercapto-2-methyl-1-butanol	121.0688764	87.49138229	HMDB32373	multiple	-0.008617714	-0.029340806	3	5.95	C5H12OS	120.0608857	M+H	ISgroup_10_1_7	24	0.069	HILIC	-0.24	0.12	-2.02	**0.04**	0.24	-0.47	0.20	-2.37	**0.02**	0.25
3-Mercapto-3-methyl-1-butanol	121.0688764	87.49138229	HMDB36149	multiple	-0.008617714	-0.029340806	3	5.95	C5H12OS	120.0608857	M+H	ISgroup_10_1_7	24	0.069	HILIC	-0.24	0.12	-2.02	**0.04**	0.24	-0.47	0.20	-2.37	**0.02**	0.25
4-(Methylthio)-1-butanol	121.0688764	87.49138229	HMDB41562	multiple	-0.008617714	-0.029340806	3	5.95	C5H12OS	120.0608857	M+H	ISgroup_10_1_7	24	0.069	HILIC	-0.24	0.12	-2.02	**0.04**	0.24	-0.47	0.20	-2.37	**0.02**	0.25
4-(Methylthio)-2-butanol	121.0688764	87.49138229	HMDB41476	multiple	-0.008617714	-0.029340806	3	5.95	C5H12OS	120.0608857	M+H	ISgroup_10_1_7	24	0.069	HILIC	-0.24	0.12	-2.02	**0.04**	0.24	-0.47	0.20	-2.37	**0.02**	0.25
xi-2-Mercapto-3-methyl-1-butanol	121.0688764	87.49138229	HMDB39781	multiple	-0.008617714	-0.029340806	3	5.95	C5H12OS	120.0608857	M+H	ISgroup_10_1_7	24	0.069	HILIC	-0.24	0.12	-2.02	**0.04**	0.24	-0.47	0.20	-2.37	**0.02**	0.25
Taurine	126.0219624	79.83584075	HMDB00251	unique	0.03584075	0.019202233	3	0.16	C2H7NO3S	125.0146638	M+H	ISgroup_57_1_3	24.9	0.022	HILIC	-0.08	0.12	-0.68	**0.50**	0.80	-0.13	0.20	-0.64	0.52	0.86
Dihydrothymine	129.0658526	86.26353603	HMDB00079	multiple	-0.036463968	0.020458487	3	0	C5H8N2O2	128.0585775	M+H	ISgroup_55_1_7	27.3	0.066	HILIC	-0.37	0.14	-2.66	**0.01**	0.09	-0.51	0.24	-2.14	**0.03**	0.31
L-Cyclo(alanylglycyl)	129.0658526	86.26353603	HMDB31547	multiple	-0.036463968	0.020458487	3	0	C5H8N2O2	128.0585775	M+H	ISgroup_55_1_7	27.3	0.066	HILIC	-0.37	0.14	-2.66	**0.01**	0.09	-0.51	0.24	-2.14	**0.03**	0.31
Squamolone	129.0658526	86.26353603	HMDB29874	multiple	-0.036463968	0.020458487	3	0	C5H8N2O2	128.0585775	M+H	ISgroup_55_1_7	27.3	0.066	HILIC	-0.37	0.14	-2.66	**0.01**	0.09	-0.51	0.24	-2.14	**0.03**	0.31
1-Pyrroline-4-hydroxy-2-carboxylate	130.0498753	94.74563001	HMDB02234	multiple	0.045630013	-0.035861233	3	0.08	C5H7NO3	129.0425931	M+H	ISgroup_21_1_5	30.7	0.05	HILIC	-0.40	0.12	-3.36	**0.00**	**0.02**	-0.07	0.17	-0.43	0.67	0.92
5-Oxoprolinate	130.0498753	94.74563001	HMDB60262	multiple	0.045630013	-0.035861233	3	0.08	C5H7NO3	129.0425931	M+H	ISgroup_21_1_5	30.7	0.05	HILIC	-0.40	0.12	-3.36	**0.00**	**0.02**	-0.07	0.17	-0.43	0.67	0.92
N-Acryloylglycine	130.0498753	94.74563001	HMDB01843	multiple	0.045630013	-0.035861233	3	0.08	C5H7NO3	129.0425931	M+H	ISgroup_21_1_5	30.7	0.05	HILIC	-0.40	0.12	-3.36	**0.00**	**0.02**	-0.07	0.17	-0.43	0.67	0.92
Pyroglutamic acid	130.0498753	94.74563001	HMDB00267	multiple	0.045630013	-0.035861233	3	0.08	C5H7NO3	129.0425931	M+H	ISgroup_21_1_5	30.7	0.05	HILIC	-0.40	0.12	-3.36	**0.00**	**0.02**	-0.07	0.17	-0.43	0.67	0.92
Pyrrolidonecarboxylic acid	130.0498753	94.74563001	HMDB00805	multiple	0.045630013	-0.035861233	3	0.08	C5H7NO3	129.0425931	M+H	ISgroup_21_1_5	30.7	0.05	HILIC	-0.40	0.12	-3.36	**0.00**	**0.02**	-0.07	0.17	-0.43	0.67	0.92
Pyrroline hydroxycarboxylic acid	130.0498753	94.74563001	HMDB01369	multiple	0.045630013	-0.035861233	3	0.08	C5H7NO3	129.0425931	M+H	ISgroup_21_1_5	30.7	0.05	HILIC	-0.40	0.12	-3.36	**0.00**	**0.02**	-0.07	0.17	-0.43	0.67	0.92
dimethadione	130.0498753	94.74563001	HMDB61093	multiple	0.045630013	-0.035861233	3	0.08	C5H7NO3	129.0425931	M+H	ISgroup_21_1_5	30.7	0.05	HILIC	-0.40	0.12	-3.36	**0.00**	**0.02**	-0.07	0.17	-0.43	0.67	0.92
2-(Methylthio)methyl-2-butenal	131.0532507	97.33070679	HMDB31370	multiple	0.030706789	0.005636568	3	5.65	C6H10OS	130.0452356	M+H	ISgroup_21_1_6	26.4	0.053	HILIC	-0.36	0.12	-3.01	**0.00**	**0.04**	-0.18	0.17	-1.06	0.29	0.75
2-Ethyldihydro-3(2H)-thiophenone	131.0532507	97.33070679	HMDB40238	multiple	0.030706789	0.005636568	3	5.65	C6H10OS	130.0452356	M+H	ISgroup_21_1_6	26.4	0.053	HILIC	-0.36	0.12	-3.01	**0.00**	**0.04**	-0.18	0.17	-1.06	0.29	0.75
S-2-Propenyl propanethioate	131.0532507	97.33070679	HMDB37493	multiple	0.030706789	0.005636568	3	5.65	C6H10OS	130.0452356	M+H	ISgroup_21_1_6	26.4	0.053	HILIC	-0.36	0.12	-3.01	**0.00**	**0.04**	-0.18	0.17	-1.06	0.29	0.75
Beta-Guanidinopropionic acid	132.0767076	83.6448874	HMDB13222	multiple	0.044887398	-0.018409317	3	0.3	C4H9N3O2	131.0694765	M+H	ISgroup_55_1_8	29	0.077	HILIC	0.18	0.12	1.48	**0.14**	0.46	0.03	0.21	0.14	0.89	0.98
Creatine	132.0767076	83.6448874	HMDB00064	multiple	0.044887398	-0.018409317	3	0.3	C4H9N3O2	131.0694765	M+H	ISgroup_55_1_8	29	0.077	HILIC	0.18	0.12	1.48	**0.14**	0.46	0.03	0.21	0.14	0.89	0.98
Taurine	148.0038947	75.53748397	HMDB00251	unique	0.037483969	0.031893628	3	0.07	C2H7NO3S	125.0146638	M+Na	ISgroup_57_1_1	25.3	0.004	HILIC	-0.10	0.12	-0.80	**0.42**	0.75	-0.14	0.19	-0.71	0.48	0.85
3-(Carboxymethylamino)propanoic acid	148.0604207	89.47885317	HMDB33550	multiple	-0.021146827	0.004577895	3	0.07	C5H9NO4	147.0531578	M+H	ISgroup_27_1_7	27.8	0.06	HILIC	0.39	0.11	3.37	**0.00**	**0.02**	-0.07	0.19	-0.39	0.70	0.93
D-Glutamic acid	148.0604207	89.47885317	HMDB03339	multiple	-0.021146827	0.004577895	3	0.07	C5H9NO4	147.0531578	M+H	ISgroup_27_1_7	27.8	0.06	HILIC	0.39	0.11	3.37	**0.00**	**0.02**	-0.07	0.19	-0.39	0.70	0.93

DL-Glutamate	148.0604207	89.47885317	HMDB60475	multiple	-0.021146827	0.004577895	3	0.07	C5H9NO4	147.0531578	M+H	ISgroup_27_1_7	27.8	0.06	HILIC	0.39	0.11	3.37	**0.00**	**0.02**	-0.07	0.19	-0.39	0.70	0.93
L-4-Hydroxyglutamate semialdehyde	148.0604207	89.47885317	HMDB06556	multiple	-0.021146827	0.004577895	3	0.07	C5H9NO4	147.0531578	M+H	ISgroup_27_1_7	27.8	0.06	HILIC	0.39	0.11	3.37	**0.00**	**0.02**	-0.07	0.19	-0.39	0.70	0.93
L-Glutamic acid	148.0604207	89.47885317	HMDB00148	multiple	-0.021146827	0.004577895	3	0.07	C5H9NO4	147.0531578	M+H	ISgroup_27_1_7	27.8	0.06	HILIC	0.39	0.11	3.37	**0.00**	**0.02**	-0.07	0.19	-0.39	0.70	0.93
N-Acetylserine	148.0604207	89.47885317	HMDB02931	multiple	-0.021146827	0.004577895	3	0.07	C5H9NO4	147.0531578	M+H	ISgroup_27_1_7	27.8	0.06	HILIC	0.39	0.11	3.37	**0.00**	**0.02**	-0.07	0.19	-0.39	0.70	0.93
N-Methyl-D-aspartic acid	148.0604207	89.47885317	HMDB02393	multiple	-0.021146827	0.004577895	3	0.07	C5H9NO4	147.0531578	M+H	ISgroup_27_1_7	27.8	0.06	HILIC	0.39	0.11	3.37	**0.00**	**0.02**	-0.07	0.19	-0.39	0.70	0.93
O-Acetylserine	148.0604207	89.47885317	HMDB03011	multiple	-0.021146827	0.004577895	3	0.07	C5H9NO4	147.0531578	M+H	ISgroup_27_1_7	27.8	0.06	HILIC	0.39	0.11	3.37	**0.00**	**0.02**	-0.07	0.19	-0.39	0.70	0.93
2-(Methylthio)methyl-2-butenal	148.0796265	94.79743135	HMDB31370	multiple	-0.002568651	-0.023736037	3	3.85	C6H10OS	130.0452356	M+NH4	ISgroup_21_1_8	27.6	0.08	HILIC	-0.41	0.12	-3.52	**0.00**	**0.01**	-0.18	0.17	-1.08	0.28	0.74
2-Ethyldihydro-3(2H)-thiophenone	148.0796265	94.79743135	HMDB40238	multiple	-0.002568651	-0.023736037	3	3.85	C6H10OS	130.0452356	M+NH4	ISgroup_21_1_8	27.6	0.08	HILIC	-0.41	0.12	-3.52	**0.00**	**0.01**	-0.18	0.17	-1.08	0.28	0.74
S-2-Propenyl propanethioate	148.0796265	94.79743135	HMDB37493	multiple	-0.002568651	-0.023736037	3	3.85	C6H10OS	130.0452356	M+NH4	ISgroup_21_1_8	27.6	0.08	HILIC	-0.41	0.12	-3.52	**0.00**	**0.01**	-0.18	0.17	-1.08	0.28	0.74
L-Methionine	150.0583768	64.37351091	HMDB00696	unique	-0.026489087	-0.021626067	3	0.33	C5H11NO2S	149.0510493	M+H	ISgroup_20_1_6	27	0.058	HILIC	0.03	0.11	0.28	**0.78**	0.93	0.04	0.19	0.23	0.82	0.96
D-Arabitol	153.0772031	90.00869136	HMDB00568	multiple	0.008691357	0.019990897	3	9.47	C5H12O5	152.0684735	M+H	ISgroup_3_1_8	25.7	0.077	HILIC	0.03	0.11	0.26	**0.80**	0.93	-0.48	0.19	-2.53	**0.01**	0.20
D-Xylitol	153.0772031	90.00869136	HMDB02917	multiple	0.008691357	0.019990897	3	9.47	C5H12O5	152.0684735	M+H	ISgroup_3_1_8	25.7	0.077	HILIC	0.03	0.11	0.26	**0.80**	0.93	-0.48	0.19	-2.53	**0.01**	0.20
L-2-(Hydroxymethyl)-1234-butanetetrol	153.0772031	90.00869136	HMDB33730	multiple	0.008691357	0.019990897	3	9.47	C5H12O5	152.0684735	M+H	ISgroup_3_1_8	25.7	0.077	HILIC	0.03	0.11	0.26	**0.80**	0.93	-0.48	0.19	-2.53	**0.01**	0.20
L-Arabitol	153.0772031	90.00869136	HMDB01851	multiple	0.008691357	0.019990897	3	9.47	C5H12O5	152.0684735	M+H	ISgroup_3_1_8	25.7	0.077	HILIC	0.03	0.11	0.26	**0.80**	0.93	-0.48	0.19	-2.53	**0.01**	0.20
Ribitol	153.0772031	90.00869136	HMDB00508	multiple	0.008691357	0.019990897	3	9.47	C5H12O5	152.0684735	M+H	ISgroup_3_1_8	25.7	0.077	HILIC	0.03	0.11	0.26	**0.80**	0.93	-0.48	0.19	-2.53	**0.01**	0.20
1-(Hydroxymethyl)-55-dimethyl-24-imidazolidinedione	159.0764319	102.2237385	HMDB31670	unique	0.023738547	0.011788481	3	0.06	C6H10N2O3	158.0691422	M+H	ISgroup_9_1_8	27.1	0.076	HILIC	-0.53	0.12	-4.30	**0.00**	**0.00**	-0.73	0.20	-3.72	**0.00**	**0.02**
Taurine	169.9858663	75.52256879	HMDB00251	unique	0.022568785	-0.021619459	3	0.29	C2H7NO3S	125.0146638	M+2Na-H	ISgroup_57_1_99	26.5	0.986	HILIC	-0.22	0.12	-1.73	**0.08**	0.36	-0.23	0.19	-1.19	0.24	0.70
Dihydrothymine	173.0298062	86.51538398	HMDB00079	multiple	0.015383984	-0.02192113	3	0.4	C5H8N2O2	128.0585775	M+2Na-H	ISgroup_55_1_3	23.2	0.03	HILIC	-0.29	0.14	-2.14	**0.03**	0.21	-0.54	0.23	-2.30	**0.02**	0.26
L-Cyclo(alanylglycyl)	173.0298062	86.51538398	HMDB31547	multiple	0.015383984	-0.02192113	3	0.4	C5H8N2O2	128.0585775	M+2Na-H	ISgroup_55_1_3	23.2	0.03	HILIC	-0.29	0.14	-2.14	**0.03**	0.21	-0.54	0.23	-2.30	**0.02**	0.26
Squamolone	173.0298062	86.51538398	HMDB29874	multiple	0.015383984	-0.02192113	3	0.4	C5H8N2O2	128.0585775	M+2Na-H	ISgroup_55_1_3	23.2	0.03	HILIC	-0.29	0.14	-2.14	**0.03**	0.21	-0.54	0.23	-2.30	**0.02**	0.26
D-Arginine	175.1189479	109.2300623	HMDB03416	multiple	0.030062292	-0.012024104	3	0	C6H14N4O2	174.1116757	M+H	ISgroup_23_1_12	31.3	0.119	HILIC	-0.11	0.12	-0.96	**0.34**	0.69	-0.04	0.18	-0.22	0.83	0.97
L-Arginine	175.1189479	109.2300623	HMDB00517	multiple	0.030062292	-0.012024104	3	0	C6H14N4O2	174.1116757	M+H	ISgroup_23_1_12	31.3	0.119	HILIC	-0.11	0.12	-0.96	**0.34**	0.69	-0.04	0.18	-0.22	0.83	0.97
Beta-Guanidinopropionic acid	176.0406721	85.42099922	HMDB13222	multiple	0.020999223	0.011966269	3	0.17	C4H9N3O2	131.0694765	M+2Na-H	ISgroup_55_1_5	24.6	0.041	HILIC	0.19	0.12	1.57	**0.12**	0.43	0.18	0.22	0.84	0.40	0.82
Creatine	176.0406721	85.42099922	HMDB00064	multiple	0.020999223	0.011966269	3	0.17	C4H9N3O2	131.0694765	M+2Na-H	ISgroup_55_1_5	24.6	0.041	HILIC	0.19	0.12	1.57	**0.12**	0.43	0.18	0.22	0.84	0.40	0.82
Argininic acid	176.1029855	102.3720203	HMDB03148	multiple	-0.027979653	-0.025457038	3	0.11	C6H13N3O3	175.0956913	M+H	ISgroup_9_1_11	28.4	0.103	HILIC	-0.49	0.12	-3.96	**0.00**	**0.00**	-0.75	0.20	-3.75	**0.00**	**0.01**
Citrulline	176.1029855	102.3720203	HMDB00904	multiple	-0.027979653	-0.025457038	3	0.11	C6H13N3O3	175.0956913	M+H	ISgroup_9_1_11	28.4	0.103	HILIC	-0.49	0.12	-3.96	**0.00**	**0.00**	-0.75	0.20	-3.75	**0.00**	**0.01**
6-Chloro-N-(1-methylethyl)-135-triazine-24-diamine	188.0706097	59.80558851	HMDB33249	multiple	0.005588514	-0.001809488	3	4.57	C6H10ClN5	187.0624731	M+H	ISgroup_5_1_8	29.5	0.071	HILIC	-0.38	0.12	-3.10	**0.00**	**0.03**	-0.15	0.20	-0.77	0.44	0.83
Indoleacrylic acid	188.0706097	59.80558851	HMDB00734	multiple	0.005588514	-0.001809488	3	0.05	C11H9NO2	187.0633285	M+H	ISgroup_5_1_8	29.5	0.071	HILIC	-0.38	0.12	-3.10	**0.00**	**0.03**	-0.15	0.20	-0.77	0.44	0.83
3-(Carboxymethylamino)propanoic acid	192.0243214	88.82329231	HMDB33550	multiple	0.023292308	0.007276393	3	0	C5H9NO4	147.0531578	M+2Na-H	ISgroup_27_1_3	24.9	0.024	HILIC	0.32	0.12	2.77	**0.01**	0.07	0.39	0.20	1.92	0.05	0.39
D-Glutamic acid	192.0243214	88.82329231	HMDB03339	multiple	0.023292308	0.007276393	3	0	C5H9NO4	147.0531578	M+2Na-H	ISgroup_27_1_3	24.9	0.024	HILIC	0.32	0.12	2.77	**0.01**	0.07	0.39	0.20	1.92	0.05	0.39
DL-Glutamate	192.0243214	88.82329231	HMDB60475	multiple	0.023292308	0.007276393	3	0	C5H9NO4	147.0531578	M+2Na-H	ISgroup_27_1_3	24.9	0.024	HILIC	0.32	0.12	2.77	**0.01**	0.07	0.39	0.20	1.92	0.05	0.39
L-4-Hydroxyglutamate semialdehyde	192.0243214	88.82329231	HMDB06556	multiple	0.023292308	0.007276393	3	0	C5H9NO4	147.0531578	M+2Na-H	ISgroup_27_1_3	24.9	0.024	HILIC	0.32	0.12	2.77	**0.01**	0.07	0.39	0.20	1.92	0.05	0.39

L-Glutamic acid	192.0243214	88.82329231	HMDB00148	multiple	0.023292308	0.007276393	3	0	C5H9NO4	147.0531578	M+2Na-H	ISgroup_27_1_3	24.9	0.024	HILIC	0.32	0.12	2.77	**0.01**	0.07	0.39	0.20	1.92	0.05	0.39
N-Acetylserine	192.0243214	88.82329231	HMDB02931	multiple	0.023292308	0.007276393	3	0	C5H9NO4	147.0531578	M+2Na-H	ISgroup_27_1_3	24.9	0.024	HILIC	0.32	0.12	2.77	**0.01**	0.07	0.39	0.20	1.92	0.05	0.39
N-Methyl-D-aspartic acid	192.0243214	88.82329231	HMDB02393	multiple	0.023292308	0.007276393	3	0	C5H9NO4	147.0531578	M+2Na-H	ISgroup_27_1_3	24.9	0.024	HILIC	0.32	0.12	2.77	**0.01**	0.07	0.39	0.20	1.92	0.05	0.39
O-Acetylserine	192.0243214	88.82329231	HMDB03011	multiple	0.023292308	0.007276393	3	0	C5H9NO4	147.0531578	M+2Na-H	ISgroup_27_1_3	24.9	0.024	HILIC	0.32	0.12	2.77	**0.01**	0.07	0.39	0.20	1.92	0.05	0.39
L-Methionine	194.022288	65.05717883	HMDB00696	unique	-0.042821166	-0.010357539	3	0.41	C5H11NO2S	149.0510493	M+2Na-H	ISgroup_20_1_3	23.8	0.022	HILIC	-0.10	0.12	-0.89	**0.38**	0.72	-0.03	0.19	-0.17	0.87	0.97
D-Arabitol	197.0411158	89.65939444	HMDB00568	multiple	-0.040605556	-0.021245144	3	7.56	C5H12O5	152.0684735	M+2Na-H	ISgroup_3_1_5	22.9	0.041	HILIC	-0.11	0.12	-0.99	**0.32**	0.68	-0.38	0.18	-2.14	**0.03**	0.31
D-Xylitol	197.0411158	89.65939444	HMDB02917	multiple	-0.040605556	-0.021245144	3	7.56	C5H12O5	152.0684735	M+2Na-H	ISgroup_3_1_5	22.9	0.041	HILIC	-0.11	0.12	-0.99	**0.32**	0.68	-0.38	0.18	-2.14	**0.03**	0.31
L-2-(Hydroxymethyl)-1234-butanetetrol	197.0411158	89.65939444	HMDB33730	multiple	-0.040605556	-0.021245144	3	7.56	C5H12O5	152.0684735	M+2Na-H	ISgroup_3_1_5	22.9	0.041	HILIC	-0.11	0.12	-0.99	**0.32**	0.68	-0.38	0.18	-2.14	**0.03**	0.31
L-Arabitol	197.0411158	89.65939444	HMDB01851	multiple	-0.040605556	-0.021245144	3	7.56	C5H12O5	152.0684735	M+2Na-H	ISgroup_3_1_5	22.9	0.041	HILIC	-0.11	0.12	-0.99	**0.32**	0.68	-0.38	0.18	-2.14	**0.03**	0.31
Ribitol	197.0411158	89.65939444	HMDB00508	multiple	-0.040605556	-0.021245144	3	7.56	C5H12O5	152.0684735	M+2Na-H	ISgroup_3_1_5	22.9	0.041	HILIC	-0.11	0.12	-0.99	**0.32**	0.68	-0.38	0.18	-2.14	**0.03**	0.31
1-(2-Thienyl)-1-heptanone	197.1009522	109.8270569	HMDB40241	unique	0.027056929	0.011158975	3	7.56	C11H16OS	196.0921858	M+H	ISgroup_35_1_11	26.8	0.101	HILIC	-0.26	0.12	-2.12	0.03	0.21	-0.06	0.17	-0.37	**0.71**	0.93
Argininic acid	198.0850947	100.1133305	HMDB03148	multiple	0.013330514	0.023889206	3	0.91	C6H13N3O3	175.0956913	M+Na	ISgroup_9_1_9	26.7	0.085	HILIC	-0.54	0.12	-4.52	0.00	**0.00**	-0.76	0.20	-3.78	**0.00**	**0.01**
Citrulline	198.0850947	100.1133305	HMDB00904	multiple	0.013330514	0.023889206	3	0.91	C6H13N3O3	175.0956913	M+Na	ISgroup_9_1_9	26.7	0.085	HILIC	-0.54	0.12	-4.52	0.00	**0.00**	-0.76	0.20	-3.78	**0.00**	**0.01**
()-Tryptophan	205.0971622	59.83309866	HMDB30396	multiple	0.033098659	0.010580439	3	0.05	C11H12N2O2	204.0898776	M+H	ISgroup_5_1_10	30.2	0.097	HILIC	-0.36	0.12	-2.93	0.00	0.05	-0.17	0.20	-0.88	**0.38**	0.80
3-Hydroxymethylantipyrine	205.0971622	59.83309866	HMDB13840	multiple	0.033098659	0.010580439	3	0.05	C11H12N2O2	204.0898776	M+H	ISgroup_5_1_10	30.2	0.097	HILIC	-0.36	0.12	-2.93	0.00	0.05	-0.17	0.20	-0.88	**0.38**	0.80
4-Hydroxyantipyrine	205.0971622	59.83309866	HMDB60878	multiple	0.033098659	0.010580439	3	0.05	C11H12N2O2	204.0898776	M+H	ISgroup_5_1_10	30.2	0.097	HILIC	-0.36	0.12	-2.93	0.00	0.05	-0.17	0.20	-0.88	**0.38**	0.80
D-Tryptophan	205.0971622	59.83309866	HMDB13609	multiple	0.033098659	0.010580439	3	0.05	C11H12N2O2	204.0898776	M+H	ISgroup_5_1_10	30.2	0.097	HILIC	-0.36	0.12	-2.93	0.00	0.05	-0.17	0.20	-0.88	**0.38**	0.80
Ethotoin	205.0971622	59.83309866	HMDB14892	multiple	0.033098659	0.010580439	3	0.05	C11H12N2O2	204.0898776	M+H	ISgroup_5_1_10	30.2	0.097	HILIC	-0.36	0.12	-2.93	0.00	0.05	-0.17	0.20	-0.88	**0.38**	0.80
L-Tryptophan	205.0971622	59.83309866	HMDB00929	multiple	0.033098659	0.010580439	3	0.05	C11H12N2O2	204.0898776	M+H	ISgroup_5_1_10	30.2	0.097	HILIC	-0.36	0.12	-2.93	0.00	0.05	-0.17	0.20	-0.88	**0.38**	0.80
Nirvanol	205.0971622	59.83309866	HMDB60533	multiple	0.033098659	0.010580439	3	0.05	C11H12N2O2	204.0898776	M+H	ISgroup_5_1_10	30.2	0.097	HILIC	-0.36	0.12	-2.93	0.00	0.05	-0.17	0.20	-0.88	**0.38**	0.80
S-nirvanol	205.0971622	59.83309866	HMDB60967	multiple	0.033098659	0.010580439	3	0.05	C11H12N2O2	204.0898776	M+H	ISgroup_5_1_10	30.2	0.097	HILIC	-0.36	0.12	-2.93	0.00	0.05	-0.17	0.20	-0.88	**0.38**	0.80
3-Methoxytyrosine	212.0916745	60.92245152	HMDB01434	multiple	0.022451521	0.021282804	3	0.28	C10H13NO4	211.0844579	M+H	ISgroup_20_1_10	21.1	0.092	HILIC	-0.17	0.12	-1.45	0.15	0.48	-0.24	0.21	-1.11	**0.27**	0.73
3-O-Methyl-a-methyldopa	212.0916745	60.92245152	HMDB60747	multiple	0.022451521	0.021282804	3	0.28	C10H13NO4	211.0844579	M+H	ISgroup_20_1_10	21.1	0.092	HILIC	-0.17	0.12	-1.45	0.15	0.48	-0.24	0.21	-1.11	**0.27**	0.73
Methyldopa	212.0916745	60.92245152	HMDB11754	multiple	0.022451521	0.021282804	3	0.28	C10H13NO4	211.0844579	M+H	ISgroup_20_1_10	21.1	0.092	HILIC	-0.17	0.12	-1.45	0.15	0.48	-0.24	0.21	-1.11	**0.27**	0.73
1-(2-Thienyl)-1-heptanone	219.0828053	108.5870041	HMDB40241	unique	-0.012995854	-0.021531315	3	6.44	C11H16OS	196.0921858	M+Na	ISgroup_35_1_9	24.6	0.083	HILIC	-0.24	0.12	-2.00	0.05	0.25	0.11	0.20	0.57	**0.57**	0.88
L-Cystine	241.0310073	231.654675	HMDB00192	unique	-0.045324988	-0.011246489	3	0.46	C6H12N2O4S2	240.0238483	M+H	ISgroup_40_1_4	26.2	0.031	HILIC	0.26	0.11	2.35	0.02	0.15	0.09	0.21	0.44	**0.66**	0.92
()-Tryptophan	249.060912	60.03243254	HMDB30396	multiple	0.032432542	0.008000467	3	0.52	C11H12N2O2	204.0898776	M+2Na-H	ISgroup_5_1_7	25.5	0.061	HILIC	-0.33	0.12	-2.80	0.01	0.07	-0.28	0.19	-1.44	**0.15**	0.58
3-Hydroxymethylantipyrine	249.060912	60.03243254	HMDB13840	multiple	0.032432542	0.008000467	3	0.52	C11H12N2O2	204.0898776	M+2Na-H	ISgroup_5_1_7	25.5	0.061	HILIC	-0.33	0.12	-2.80	0.01	0.07	-0.28	0.19	-1.44	0.15	0.58
4-Hydroxyantipyrine	249.060912	60.03243254	HMDB60878	multiple	0.032432542	0.008000467	3	0.52	C11H12N2O2	204.0898776	M+2Na-H	ISgroup_5_1_7	25.5	0.061	HILIC	-0.33	0.12	-2.80	0.01	0.07	-0.28	0.19	-1.44	0.15	0.58
D-Tryptophan	249.060912	60.03243254	HMDB13609	multiple	0.032432542	0.008000467	3	0.52	C11H12N2O2	204.0898776	M+2Na-H	ISgroup_5_1_7	25.5	0.061	HILIC	-0.33	0.12	-2.80	0.01	0.07	-0.28	0.19	-1.44	0.15	0.58
Ethotoin	249.060912	60.03243254	HMDB14892	multiple	0.032432542	0.008000467	3	0.52	C11H12N2O2	204.0898776	M+2Na-H	ISgroup_5_1_7	25.5	0.061	HILIC	-0.33	0.12	-2.80	0.01	0.07	-0.28	0.19	-1.44	0.15	0.58
L-Tryptophan	249.060912	60.03243254	HMDB00929	multiple	0.032432542	0.008000467	3	0.52	C11H12N2O2	204.0898776	M+2Na-H	ISgroup_5_1_7	25.5	0.061	HILIC	-0.33	0.12	-2.80	0.01	0.07	-0.28	0.19	-1.44	0.15	0.58

Nirvanol	249.060912	60.03243254	HMDB60533	multiple	0.032432542	0.008000467	3	0.52	C11H12N2O2	204.0898776	M+2Na-H	ISgroup_5_1_7	25.5	0.061	HILIC	-0.33	0.12	-2.80	0.01	0.07	-0.28	0.19	-1.44	0.15	0.58
S-nirvanol	249.060912	60.03243254	HMDB60967	multiple	0.032432542	0.008000467	3	0.52	C11H12N2O2	204.0898776	M+2Na-H	ISgroup_5_1_7	25.5	0.061	HILIC	-0.33	0.12	-2.80	0.01	0.07	-0.28	0.19	-1.44	0.15	0.58
3-Methoxytyrosine	256.0553514	66.61527589	HMDB01434	multiple	0.015275885	0.005514927	3	1.05	C10H13NO4	211.0844579	M+2Na-H	ISgroup_20_1_6	14.7	0.055	HILIC	-0.15	0.11	-1.32	0.19	0.54	0.07	0.19	0.36	0.72	0.94
3-O-Methyl-a-methyldopa	256.0553514	66.61527589	HMDB60747	multiple	0.015275885	0.005514927	3	1.05	C10H13NO4	211.0844579	M+2Na-H	ISgroup_20_1_6	14.7	0.055	HILIC	-0.15	0.11	-1.32	0.19	0.54	0.07	0.19	0.36	0.72	0.94
Methyldopa	256.0553514	66.61527589	HMDB11754	multiple	0.015275885	0.005514927	3	1.05	C10H13NO4	211.0844579	M+2Na-H	ISgroup_20_1_6	14.7	0.055	HILIC	-0.15	0.11	-1.32	0.19	0.54	0.07	0.19	0.36	0.72	0.94
L-Cystine	263.0129184	231.8296207	HMDB00192	unique	0.029620744	-0.006266897	3	0.57	C6H12N2O4S2	240.0238483	M+Na	ISgroup_40_1_2	20.3	0.013	HILIC	0.17	0.12	1.48	0.14	0.46	-0.27	0.18	-1.47	0.14	0.58
(2E4Z8E)-Colneleic acid	295.2265593	36.8103884	HMDB30995	multiple	0.010388401	-0.002213619	3	0.71	C18H30O3	294.2194948	M+H	ISgroup_12_1_23	21.8	0.227	HILIC	-0.01	0.11	-0.10	0.92	0.97	-0.05	0.22	-0.24	0.81	0.96
(9S10E12Z15Z)-9-Hydroxy-101215-octadecatrienoic acid	295.2265593	36.8103884	HMDB31934	multiple	0.010388401	-0.002213619	3	0.71	C18H30O3	294.2194948	M+H	ISgroup_12_1_23	21.8	0.227	HILIC	-0.01	0.11	-0.10	0.92	0.97	-0.05	0.22	-0.24	0.81	0.96
(9Z12Z14E)-16-Hydroxy-91214-octadecatrienoic acid	295.2265593	36.8103884	HMDB34586	multiple	0.010388401	-0.002213619	3	0.71	C18H30O3	294.2194948	M+H	ISgroup_12_1_23	21.8	0.227	HILIC	-0.01	0.11	-0.10	0.92	0.97	-0.05	0.22	-0.24	0.81	0.96
10-Oxo-11-octadecen-13-olide	295.2265593	36.8103884	HMDB29786	multiple	0.010388401	-0.002213619	3	0.71	C18H30O3	294.2194948	M+H	ISgroup_12_1_23	21.8	0.227	HILIC	-0.01	0.11	-0.10	0.92	0.97	-0.05	0.22	-0.24	0.81	0.96
1213-Epoxy-915-octadecadienoic acid	295.2265593	36.8103884	HMDB31088	multiple	0.010388401	-0.002213619	3	0.71	C18H30O3	294.2194948	M+H	ISgroup_12_1_23	21.8	0.227	HILIC	-0.01	0.11	-0.10	0.92	0.97	-0.05	0.22	-0.24	0.81	0.96
13-HOTE	295.2265593	36.8103884	HMDB10203	multiple	0.010388401	-0.002213619	3	0.71	C18H30O3	294.2194948	M+H	ISgroup_12_1_23	21.8	0.227	HILIC	-0.01	0.11	-0.10	0.92	0.97	-0.05	0.22	-0.24	0.81	0.96
13-OxoODE	295.2265593	36.8103884	HMDB04668	multiple	0.010388401	-0.002213619	3	0.71	C18H30O3	294.2194948	M+H	ISgroup_12_1_23	21.8	0.227	HILIC	-0.01	0.11	-0.10	0.92	0.97	-0.05	0.22	-0.24	0.81	0.96
15(16)-EpODE	295.2265593	36.8103884	HMDB10206	multiple	0.010388401	-0.002213619	3	0.71	C18H30O3	294.2194948	M+H	ISgroup_12_1_23	21.8	0.227	HILIC	-0.01	0.11	-0.10	0.92	0.97	-0.05	0.22	-0.24	0.81	0.96
1516-Epoxy-912-octadecadienoic acid	295.2265593	36.8103884	HMDB30950	multiple	0.010388401	-0.002213619	3	0.71	C18H30O3	294.2194948	M+H	ISgroup_12_1_23	21.8	0.227	HILIC	-0.01	0.11	-0.10	0.92	0.97	-0.05	0.22	-0.24	0.81	0.96
17-Hydroxylinolenic acid	295.2265593	36.8103884	HMDB11108	multiple	0.010388401	-0.002213619	3	0.71	C18H30O3	294.2194948	M+H	ISgroup_12_1_23	21.8	0.227	HILIC	-0.01	0.11	-0.10	0.92	0.97	-0.05	0.22	-0.24	0.81	0.96
2-Hydroxylinolenic acid	295.2265593	36.8103884	HMDB31103	multiple	0.010388401	-0.002213619	3	0.71	C18H30O3	294.2194948	M+H	ISgroup_12_1_23	21.8	0.227	HILIC	-0.01	0.11	-0.10	0.92	0.97	-0.05	0.22	-0.24	0.81	0.96
34-Dimethyl-5-pentyl-2-furanheptanoic acid	295.2265593	36.8103884	HMDB39603	multiple	0.010388401	-0.002213619	3	0.71	C18H30O3	294.2194948	M+H	ISgroup_12_1_23	21.8	0.227	HILIC	-0.01	0.11	-0.10	0.92	0.97	-0.05	0.22	-0.24	0.81	0.96
9(10)-EpODE	295.2265593	36.8103884	HMDB10220	multiple	0.010388401	-0.002213619	3	0.71	C18H30O3	294.2194948	M+H	ISgroup_12_1_23	21.8	0.227	HILIC	-0.01	0.11	-0.10	0.92	0.97	-0.05	0.22	-0.24	0.81	0.96
9-HOTE	295.2265593	36.8103884	HMDB10224	multiple	0.010388401	-0.002213619	3	0.71	C18H30O3	294.2194948	M+H	ISgroup_12_1_23	21.8	0.227	HILIC	-0.01	0.11	-0.10	0.92	0.97	-0.05	0.22	-0.24	0.81	0.96
9-OxoODE	295.2265593	36.8103884	HMDB04669	multiple	0.010388401	-0.002213619	3	0.71	C18H30O3	294.2194948	M+H	ISgroup_12_1_23	21.8	0.227	HILIC	-0.01	0.11	-0.10	0.92	0.97	-0.05	0.22	-0.24	0.81	0.96
A-12(13)-EpODE	295.2265593	36.8103884	HMDB10200	multiple	0.010388401	-0.002213619	3	0.71	C18H30O3	294.2194948	M+H	ISgroup_12_1_23	21.8	0.227	HILIC	-0.01	0.11	-0.10	0.92	0.97	-0.05	0.22	-0.24	0.81	0.96
Squamostanal A	295.2265593	36.8103884	HMDB29969	multiple	0.010388401	-0.002213619	3	0.71	C18H30O3	294.2194948	M+H	ISgroup_12_1_23	21.8	0.227	HILIC	-0.01	0.11	-0.10	0.92	0.97	-0.05	0.22	-0.24	0.81	0.96
Sterebin D	295.2265593	36.8103884	HMDB36832	multiple	0.010388401	-0.002213619	3	0.71	C18H30O3	294.2194948	M+H	ISgroup_12_1_23	21.8	0.227	HILIC	-0.01	0.11	-0.10	0.92	0.97	-0.05	0.22	-0.24	0.81	0.96
D-Arginine	349.2304245	108.6430351	HMDB03416	multiple	0.043035054	0.012938197	3	0.6	C6H14N4O2	174.1116757	2M+H	ISgroup_23_1_24	23.2	0.23	HILIC	-0.13	0.12	-1.13	0.26	0.61	-0.07	0.18	-0.38	0.70	0.93
L-Arginine	349.2304245	108.6430351	HMDB00517	multiple	0.043035054	0.012938197	3	0.6	C6H14N4O2	174.1116757	2M+H	ISgroup_23_1_24	23.2	0.23	HILIC	-0.13	0.12	-1.13	0.26	0.61	-0.07	0.18	-0.38	0.70	0.93
Glycerol 1-octadecanoate	359.3152725	42.97940529	HMDB31075	multiple	-0.020594709	0.006957609	3	0.89	C21H42O4	358.3083098	M+H	ISgroup_33_1_32	23.8	0.315	HILIC	0.04	0.12	0.37	0.71	0.90	0.50	0.19	2.56	**0.01**	0.19
MG(0:018:00:0)	359.3152725	42.97940529	HMDB11535	multiple	-0.020594709	0.006957609	3	0.89	C21H42O4	358.3083098	M+H	ISgroup_33_1_32	23.8	0.315	HILIC	0.04	0.12	0.37	0.71	0.90	0.50	0.19	2.56	**0.01**	0.19
MG(18:00:00:0)	359.3152725	42.97940529	HMDB11131	multiple	-0.020594709	0.006957609	3	0.89	C21H42O4	358.3083098	M+H	ISgroup_33_1_32	23.8	0.315	HILIC	0.04	0.12	0.37	0.71	0.90	0.50	0.19	2.56	**0.01**	0.19
D-Arginine	371.212108	108.4958945	HMDB03416	multiple	-0.004105505	-0.005399211	3	1.24	C6H14N4O2	174.1116757	2M+Na	ISgroup_23_1_22	23.3	0.212	HILIC	-0.09	0.11	-0.75	0.45	0.77	-0.03	0.17	-0.19	0.85	0.97
L-Arginine	371.212108	108.4958945	HMDB00517	multiple	-0.004105505	-0.005399211	3	1.24	C6H14N4O2	174.1116757	2M+Na	ISgroup_23_1_22	23.3	0.212	HILIC	-0.09	0.11	-0.75	0.45	0.77	-0.03	0.17	-0.19	0.85	0.97
(2alpha3alpha5alpha22R23R)-232223-Tetrahydroxy-25-methylergost-24(28)en-6-one	477.3581052	38.1017066	HMDB39443	multiple	0.0017066	-0.009958355	3	1.38	C29H48O5	476.3501746	M+H	ISgroup_56_1_36	18.5	0.358	HILIC	0.05	0.11	0.41	0.68	0.89	0.33	0.18	1.79	0.07	0.44

Homodolichosterone	477.3581052	38.1017066	HMDB34079	multiple	0.0017066	-0.009958355	3	1.38	C29H48O5	476.3501746	M+H	ISgroup_56_1_36	18.5	0.358	HILIC	0.05	0.11	0.41	0.68	0.89	0.33	0.18	1.79	0.07	0.44
LysoPC(16:0)	496.3396257	49.17933844	HMDB10382	unique	-0.020661557	-0.008569717	3	0.28	C24H50NO7P	495.3324895	M+H	ISgroup_59_1_34	32.6	0.34	HILIC	-0.04	0.12	-0.33	0.74	0.91	0.03	0.21	0.16	0.87	0.98
(2alpha3alpha5alpha22R23R)-232223-Tetrahydroxy-25-methylergost-24(28)en-6-one	559.40556	38.39903583	HMDB39443	multiple	-0.000964173	6.02219E-05	3	8.92	C29H48O5	476.3501746	M+2ACN+H	ISgroup_56_1_41	14	0.406	HILIC	0.09	0.12	0.73	0.47	0.78	0.22	0.20	1.09	0.28	0.74
Homodolichosterone	559.40556	38.39903583	HMDB34079	multiple	-0.000964173	6.02219E-05	3	8.92	C29H48O5	476.3501746	M+2ACN+H	ISgroup_56_1_41	14	0.406	HILIC	0.09	0.12	0.73	0.47	0.78	0.22	0.20	1.09	0.28	0.74
Alpha-D-Glucose	179.0562404	36.80263892	HMDB03345	multiple	0.002638923	0.00233506	3	0.73	C6H12O6	180.0633881	M-H	ISgroup_27_1_6	28.3	0.056	C18	0.56	0.11	5.20	0.00	**0.00**	1.03	0.21	4.82	**0.00**	**0.00**
D-Glucose	179.0562404	36.80263892	HMDB00122	multiple	0.002638923	0.00233506	3	0.73	C6H12O6	180.0633881	M-H	ISgroup_27_1_6	28.3	0.056	C18	0.56	0.11	5.20	0.00	**0.00**	1.03	0.21	4.82	**0.00**	**0.00**
(S)-N-(45-Dihydro-1-methyl-4-oxo-1H-imidazol-2-yl)alanine	184.0729057	35.97729091	HMDB34912	unique	-0.022709087	-0.023446538	3	0.76	C7H11N3O3	185.0800412	M-H	ISgroup_1_1_8	23.9	0.073	C18	-0.22	0.12	-1.84	0.07	0.31	-0.62	0.19	-3.21	**0.00**	0.08
D-Galactose	215.0328304	30.9125315	HMDB00143	multiple	0.012531501	0.001660395	3	0.19	C6H12O6	180.0633881	M+Cl	ISgroup_27_1_4	32.3	0.033	C18	0.60	0.11	5.39	0.00	**0.00**	1.29	0.25	5.15	**0.00**	**0.00**
D-Glucose	215.0328304	30.9125315	HMDB00122	multiple	0.012531501	0.001660395	3	0.19	C6H12O6	180.0633881	M+Cl	ISgroup_27_1_4	32.3	0.033	C18	0.60	0.11	5.39	0.00	**0.00**	1.29	0.25	5.15	**0.00**	**0.00**
(S)-N-(45-Dihydro-1-methyl-4-oxo-1H-imidazol-2-yl)alanine	220.0489243	30.64211513	HMDB34912	unique	0.042115129	0.01943598	3	2.36	C7H11N3O3	185.0800412	M+Cl	ISgroup_1_1_5	28.2	0.049	C18	-0.39	0.12	-3.16	0.00	**0.03**	-0.71	0.20	-3.62	**0.00**	**0.02**
(E)-11-Hexadecenoic acid	253.2172518	217.5338764	HMDB37647	multiple	0.033876368	0.007235119	3	0.2	C16H30O2	254.2245802	M-H	ISgroup_35_4_22	29.1	0.217	C18	0.03	0.12	0.27	0.79	0.93	0.08	0.19	0.41	0.68	0.97
(E)-3-Hexadecenoic acid	253.2172518	217.5338764	HMDB33791	multiple	0.033876368	0.007235119	3	0.2	C16H30O2	254.2245802	M-H	ISgroup_35_4_22	29.1	0.217	C18	0.03	0.12	0.27	0.79	0.93	0.08	0.19	0.41	0.68	0.97
(E)-6-Hexadecenoic acid	253.2172518	217.5338764	HMDB31053	multiple	0.033876368	0.007235119	3	0.2	C16H30O2	254.2245802	M-H	ISgroup_35_4_22	29.1	0.217	C18	0.03	0.12	0.27	0.79	0.93	0.08	0.19	0.41	0.68	0.97
(Z)-13-Hexadecenoic acid	253.2172518	217.5338764	HMDB35877	multiple	0.033876368	0.007235119	3	0.2	C16H30O2	254.2245802	M-H	ISgroup_35_4_22	29.1	0.217	C18	0.03	0.12	0.27	0.79	0.93	0.08	0.19	0.41	0.68	0.97
(Z)-14-Methyl-6-pentadecenoic acid	253.2172518	217.5338764	HMDB41422	multiple	0.033876368	0.007235119	3	0.2	C16H30O2	254.2245802	M-H	ISgroup_35_4_22	29.1	0.217	C18	0.03	0.12	0.27	0.79	0.93	0.08	0.19	0.41	0.68	0.97
(Z)-5-Hexadecenoic acid	253.2172518	217.5338764	HMDB32638	multiple	0.033876368	0.007235119	3	0.2	C16H30O2	254.2245802	M-H	ISgroup_35_4_22	29.1	0.217	C18	0.03	0.12	0.27	0.79	0.93	0.08	0.19	0.41	0.68	0.97
15-Hexadecanolide	253.2172518	217.5338764	HMDB31711	multiple	0.033876368	0.007235119	3	0.2	C16H30O2	254.2245802	M-H	ISgroup_35_4_22	29.1	0.217	C18	0.03	0.12	0.27	0.79	0.93	0.08	0.19	0.41	0.68	0.97
5-Dodecyldihydro-2(3H)-furanone	253.2172518	217.5338764	HMDB31145	multiple	0.033876368	0.007235119	3	0.2	C16H30O2	254.2245802	M-H	ISgroup_35_4_22	29.1	0.217	C18	0.03	0.12	0.27	0.79	0.93	0.08	0.19	0.41	0.68	0.97
Citronellyl hexanoate	253.2172518	217.5338764	HMDB38958	multiple	0.033876368	0.007235119	3	0.2	C16H30O2	254.2245802	M-H	ISgroup_35_4_22	29.1	0.217	C18	0.03	0.12	0.27	0.79	0.93	0.08	0.19	0.41	0.68	0.97
Hexadecenoate (n-C16:1)	253.2172518	217.5338764	HMDB60082	multiple	0.033876368	0.007235119	3	0.2	C16H30O2	254.2245802	M-H	ISgroup_35_4_22	29.1	0.217	C18	0.03	0.12	0.27	0.79	0.93	0.08	0.19	0.41	0.68	0.97
Hypogeic acid	253.2172518	217.5338764	HMDB02186	multiple	0.033876368	0.007235119	3	0.2	C16H30O2	254.2245802	M-H	ISgroup_35_4_22	29.1	0.217	C18	0.03	0.12	0.27	0.79	0.93	0.08	0.19	0.41	0.68	0.97
Palmitelaidic acid	253.2172518	217.5338764	HMDB12328	multiple	0.033876368	0.007235119	3	0.2	C16H30O2	254.2245802	M-H	ISgroup_35_4_22	29.1	0.217	C18	0.03	0.12	0.27	0.79	0.93	0.08	0.19	0.41	0.68	0.97
Palmitoleic acid	253.2172518	217.5338764	HMDB03229	multiple	0.033876368	0.007235119	3	0.2	C16H30O2	254.2245802	M-H	ISgroup_35_4_22	29.1	0.217	C18	0.03	0.12	0.27	0.79	0.93	0.08	0.19	0.41	0.68	0.97
Trans-Hexa-dec-2-enoic acid	253.2172518	217.5338764	HMDB10735	multiple	0.033876368	0.007235119	3	0.2	C16H30O2	254.2245802	M-H	ISgroup_35_4_22	29.1	0.217	C18	0.03	0.12	0.27	0.79	0.93	0.08	0.19	0.41	0.68	0.97
(Z)-13-Octadecenoic acid	281.2484662	242.6443551	HMDB41480	multiple	0.044355143	-0.013351532	3	0.46	C18H34O2	282.2558803	M-H	ISgroup_35_4_25	31.3	0.248	C18	0.10	0.12	0.85	0.40	0.71	0.41	0.20	2.02	**0.04**	0.44
Elaidic acid	281.2484662	242.6443551	HMDB00573	multiple	0.044355143	-0.013351532	3	0.46	C18H34O2	282.2558803	M-H	ISgroup_35_4_25	31.3	0.248	C18	0.10	0.12	0.85	0.40	0.71	0.41	0.20	2.02	**0.04**	0.44
Ethyl 9-hexadecenoate	281.2484662	242.6443551	HMDB59871	multiple	0.044355143	-0.013351532	3	0.46	C18H34O2	282.2558803	M-H	ISgroup_35_4_25	31.3	0.248	C18	0.10	0.12	0.85	0.40	0.71	0.41	0.20	2.02	**0.04**	0.44
Oleic acid	281.2484662	242.6443551	HMDB00207	multiple	0.044355143	-0.013351532	3	0.46	C18H34O2	282.2558803	M-H	ISgroup_35_4_25	31.3	0.248	C18	0.10	0.12	0.85	0.40	0.71	0.41	0.20	2.02	**0.04**	0.44
Vaccenic acid	281.2484662	242.6443551	HMDB03231	multiple	0.044355143	-0.013351532	3	0.46	C18H34O2	282.2558803	M-H	ISgroup_35_4_25	31.3	0.248	C18	0.10	0.12	0.85	0.40	0.71	0.41	0.20	2.02	**0.04**	0.44

Supplementary Table 5. Pathway enrichment analysis results.

		PD Patients				non-PD Participants			
**pathway**	**Column**	**overlap size**	**pathway size**	**p-value**	**overlap metabolic features**	**overlap size**	**pathway size**	**p-value**	**overlap metabolic features**
1- and 2-Methylnaphthalene degradation	HILIC	0	0	0.46		0	0	0.37	
3-Chloroacrylic acid degradation	HILIC	0	0	0.46		0	0	0.37	
3-oxo-10R-octadecatrienoate beta-oxidation	HILIC	0	2	0.46		0	2	0.37	
Alanine and Aspartate Metabolism	HILIC	6	7	0.00	beta-Alanine/L-Alanine/Sarcosine; N-Methylglycine/D-Alanine$DL-Glutamate; DL-Glutaminic acid; 2-Aminoglutaric acid; Glutamate/D-Glutamate; D-Glutamic acid; D-Glutaminic acid; D-2-Aminoglutaric acid/L-4-Hydroxyglutamate semialdehyde/L-Glutamate$D-Arginine; D-2-Amino-5-guanidinovaleric acid/L-Arginine$D-Glutamine; D-2-Aminoglutaramic acid/L-Glutamine/3-Ureidoisobutyrate$L-Aspartate/D-Aspartate$L-Citrulline; 2-Amino-5-ureidovaleric acid; Citrulline	1	7	0.26	
Alkaloid biosynthesis II	HILIC	0	1	0.46		0	1	0.37	
Aminosugars metabolism	HILIC	3	5	0.02	DL-Glutamate; DL-Glutaminic acid; 2-Aminoglutaric acid; Glutamate/D-Glutamate; D-Glutamic acid; D-Glutaminic acid; D-2-Aminoglutaric acid/L-4-Hydroxyglutamate semialdehyde/L-Glutamate$N-Acetyl-D-mannosamine; 2-Acetamido-2-deoxy-D-mannose/N-Acetylgalactosamine; N-Acetylchondrosamine; 2-Acetamido-2-deoxygalactose; GalNAc/N-Acetyl-D-glucosamine/N-Acetyl-D-galactosamine$D-Glutamine; D-2-Aminoglutaramic acid/L-Glutamine/3-Ureidoisobutyrate	1	5	0.20	N-Acetyl-D-mannosamine; 2-Acetamido-2-deoxy-D-mannose/N-Acetylgalactosamine; N-Acetylchondrosamine; 2-Acetamido-2-deoxygalactose; GalNAc/N-Acetyl-D-glucosamine/N-Acetyl-D-galactosamine
Androgen and estrogen biosynthesis and metabolism	HILIC	0	1	0.46		0	1	0.37	
Arachidonic acid metabolism	HILIC	1	4	0.30	DL-Glutamate; DL-Glutaminic acid; 2-Aminoglutaric acid; Glutamate/D-Glutamate; D-Glutamic acid; D-Glutaminic acid; D-2-Aminoglutaric acid/L-4-Hydroxyglutamate semialdehyde/L-Glutamate	0	4	0.37	
Arginine and Proline Metabolism	HILIC	6	14	0.01	1-Pyrroline-4-hydroxy-2-carboxylate/5-Oxo-L-proline/L-1-Pyrroline-3-hydroxy-5-carboxylate/5-Oxoproline$DL-Glutamate; DL-Glutaminic acid; 2-Aminoglutaric acid; Glutamate/D-Glutamate; D-Glutamic acid; D-Glutaminic acid; D-2-Aminoglutaric acid/L-4-Hydroxyglutamate semialdehyde/L-Glutamate$D-Arginine; D-2-Amino-5-guanidinovaleric acid/L-Arginine$D-Glutamine; D-2-Aminoglutaramic acid/L-Glutamine/3-Ureidoisobutyrate$L-Aspartate/D-Aspartate$L-Citrulline; 2-Amino-5-ureidovaleric acid; Citrulline	1	14	0.34	
Ascorbate (Vitamin C) and Aldarate Metabolism	HILIC	2	5	0.12	$/Hypoxanthine	0	5	0.37	

Aspartate and asparagine metabolism	HILIC	7	27	0.13	$DL-Glutamate; DL-Glutaminic acid; 2-Aminoglutaric acid; Glutamate/D-Glutamate; D-Glutamic acid; D-Glutaminic acid; D-2-Aminoglutaric acid/L-4-Hydroxyglutamate semialdehyde/L-Glutamate$D-Arginine; D-2-Amino-5-guanidinovaleric acid/L-Arginine$D-Glutamine; D-2-Aminoglutaramic acid/L-Glutamine/3-Ureidoisobutyrate$L-Aspartate/D-Aspartate$1-Pyrroline-4-hydroxy-2-carboxylate/5-Oxo-L-proline/L-1-Pyrroline-3-hydroxy-5-carboxylate/5-Oxoproline$L-Citrulline; 2-Amino-5-ureidovaleric acid; Citrulline	3	27	0.28	$$
Atrazine degradation	HILIC	0	0	0.46		0	0	0.37	
Benzoate degradation via CoA ligation	HILIC	0	1	0.46		0	1	0.37	
Beta-Alanine metabolism	HILIC	3	9	0.12	DL-Glutamate; DL-Glutaminic acid; 2-Aminoglutaric acid; Glutamate/D-Glutamate; D-Glutamic acid; D-Glutaminic acid; D-2-Aminoglutaric acid/L-4-Hydroxyglutamate semialdehyde/L-Glutamate$L-Aspartate/D-Aspartate$beta-Alanine/L-Alanine/Sarcosine; N-Methylglycine/D-Alanine	0	9	0.37	
Bile acid biosynthesis	HILIC	0	4	0.46		0	4	0.37	
Biopterin metabolism	HILIC	1	3	0.24	6-Pyruvoyl-5,6,7,8-tetrahydropterin/Sepiapterin/fructoseglycine	0	3	0.37	
Blood Group Biosynthesis	HILIC	0	1	0.46		0	1	0.37	
Butanoate metabolism	HILIC	2	8	0.24	DL-Glutamate; DL-Glutaminic acid; 2-Aminoglutaric acid; Glutamate/D-Glutamate; D-Glutamic acid; D-Glutaminic acid; D-2-Aminoglutaric acid/L-4-Hydroxyglutamate semialdehyde/L-Glutamate$N6-Acetyl-L-lysine	0	8	0.37	
C21-steroid hormone biosynthesis and metabolism	HILIC	0	5	0.46		0	5	0.37	
C5-Branched dibasic acid metabolism	HILIC	0	0	0.46		0	0	0.37	
Caffeine metabolism	HILIC	2	3	0.03	D-Tagatose; lyxo-Hexulose/1,7-Dimethylxanthine; Paraxanthine/beta-D-Glucose/D-Fructose/alpha-D-Glucose//myo-Inositol/D-Hexose/beta-D-Galactose/L-Sorbose; L-xylo-Hexulose/alpha-D-Galactose/D-Glucose//Galactose/D-Mannose/beta-D-Fructose; beta-Fruit sugar; beta-D-arabino-Hexulose; beta-Levulose; Fructose/Glucose/D-Galactose$5-Acetylamino-6-formylamino-3-methyluracil; AFMU	1	3	0.13	D-Tagatose; lyxo-Hexulose/1,7-Dimethylxanthine; Paraxanthine/beta-D-Glucose/D-Fructose/alpha-D-Glucose//myo-Inositol/D-Hexose/beta-D-Galactose/L-Sorbose; L-xylo-Hexulose/alpha-D-Galactose/D-Glucose//Galactose/D-Mannose/beta-D-Fructose; beta-Fruit sugar; beta-D-arabino-Hexulose; beta-Levulose; Fructose/Glucose/D-Galactose
Carbon fixation	HILIC	1	1	0.08	L-Aspartate/D-Aspartate	0	1	0.37	

Carnitine shuttle	HILIC	7	24	0.06	$$$$$$	1	24	0.37	
Chondroitin sulfate degradation	HILIC	2	3	0.03	N-Acetyl-D-mannosamine; 2-Acetamido-2-deoxy-D-mannose/N-Acetylgalactosamine; N-Acetylchondrosamine; 2-Acetamido-2-deoxygalactose; GalNAc/N-Acetyl-D-glucosamine/N-Acetyl-D-galactosamine$D-Tagatose; lyxo-Hexulose/1,7-Dimethylxanthine; Paraxanthine/beta-D-Glucose/D-Fructose/alpha-D-Glucose//myo-Inositol/D-Hexose/beta-D-Galactose/L-Sorbose; L-xylo-Hexulose/alpha-D-Galactose/D-Glucose//Galactose/D-Mannose/beta-D-Fructose; beta-Fruit sugar; beta-D-arabino-Hexulose; beta-Levulose; Fructose/Glucose/D-Galactose	2	3	0.01	D-Tagatose; lyxo-Hexulose/1,7-Dimethylxanthine; Paraxanthine/beta-D-Glucose/D-Fructose/alpha-D-Glucose//myo-Inositol/D-Hexose/beta-D-Galactose/L-Sorbose; L-xylo-Hexulose/alpha-D-Galactose/D-Glucose//Galactose/D-Mannose/beta-D-Fructose; beta-Fruit sugar; beta-D-arabino-Hexulose; beta-Levulose; Fructose/Glucose/D-Galactose$N-Acetyl-D-mannosamine; 2-Acetamido-2-deoxy-D-mannose/N-Acetylgalactosamine; N-Acetylchondrosamine; 2-Acetamido-2-deoxygalactose; GalNAc/N-Acetyl-D-glucosamine/N-Acetyl-D-galactosamine
CoA Catabolism	HILIC	2	2	0.01	Pantetheine; (R)-Pantetheine$(R)-Pantothenate	0	2	0.37	
D4&E4-neuroprostanes formation	HILIC	0	0	0.46		0	0	0.37	
De novo fatty acid biosynthesis	HILIC	1	14	0.45		2	14	0.22	$Hexadecanoate (n-C16:0)
Dimethyl-branched-chain fatty acid mitochondrial beta-oxidation	HILIC	0	0	0.46		0	0	0.37	
Di-unsaturated fatty acid beta-oxidation	HILIC	1	2	0.17		0	2	0.37	
Drug metabolism - cytochrome P450	HILIC	2	8	0.24	$	2	8	0.09	$
Drug metabolism - other enzymes	HILIC	0	2	0.46		1	2	0.08	
Dynorphin metabolism	HILIC	0	0	0.46		0	0	0.37	
Electron transport chain	HILIC	0	0	0.46		0	0	0.37	
Fatty acid activation	HILIC	1	17	0.46		2	17	0.27	$Hexadecanoate (n-C16:0)
Fatty Acid Metabolism	HILIC	1	9	0.43		1	9	0.30	Hexadecanoate (n-C16:0)
Fatty acid oxidation	HILIC	0	5	0.46		1	5	0.20	
Fatty acid oxidation, peroxisome	HILIC	0	3	0.46		1	3	0.13	

Fructose and mannose metabolism	HILIC	3	4	0.01	D-Sorbitol/Galactitol/L-Iditol$6-Pyruvoyl-5,6,7,8-tetrahydropterin/Sepiapterin/fructoseglycine$D-Tagatose; lyxo-Hexulose/1,7-Dimethylxanthine; Paraxanthine/beta-D-Glucose/D-Fructose/alpha-D-Glucose//myo-Inositol/D-Hexose/beta-D-Galactose/L-Sorbose; L-xylo-Hexulose/alpha-D-Galactose/D-Glucose//Galactose/D-Mannose/beta-D-Fructose; beta-Fruit sugar; beta-D-arabino-Hexulose; beta-Levulose; Fructose/Glucose/D-Galactose	2	4	0.02	D-Tagatose; lyxo-Hexulose/1,7-Dimethylxanthine; Paraxanthine/beta-D-Glucose/D-Fructose/alpha-D-Glucose//myo-Inositol/D-Hexose/beta-D-Galactose/L-Sorbose; L-xylo-Hexulose/alpha-D-Galactose/D-Glucose//Galactose/D-Mannose/beta-D-Fructose; beta-Fruit sugar; beta-D-arabino-Hexulose; beta-Levulose; Fructose/Glucose/D-Galactose$L-Fucose 1-phosphate; 6-Deoxy-L-galactose 1-phosphate; beta-L-Fucose 1-phosphate
Galactose metabolism	HILIC	5	5	0.00	N-Acetyl-D-mannosamine; 2-Acetamido-2-deoxy-D-mannose/N-Acetylgalactosamine; N-Acetylchondrosamine; 2-Acetamido-2-deoxygalactose; GalNAc/N-Acetyl-D-glucosamine/N-Acetyl-D-galactosamine$alpha-Maltose; alpha-Malt sugar/1-alpha-D-Galactosyl-myo-inositol; 1-O-alpha-D-Galactosyl-D-myo-inositol; Galactinol/Isomaltose; Brachiose/Trehalose/Lactose/Sucrose/Maltose/Cellobiose/Epimelibiose/Melibiose/beta-D-Gal-(1->4)-D-Glc$alpha-Maltose; alpha-Malt sugar/1-alpha-D-Galactosyl-myo-inositol; 1-O-alpha-D-Galactosyl-D-myo-inositol; Galactinol/Isomaltose; Brachiose/Trehalose/Lactose/Sucrose/Maltose/Cellobiose/Epimelibiose/Melibiose/beta-D-Gal-(1->4)-D-Glc$D-Tagatose; lyxo-Hexulose/1,7-Dimethylxanthine; Paraxanthine/beta-D-Glucose/D-Fructose/alpha-D-Glucose//myo-Inositol/D-Hexose/beta-D-Galactose/L-Sorbose; L-xylo-Hexulose/alpha-D-Galactose/D-Glucose//Galactose/D-Mannose/beta-D-Fructose; beta-Fruit sugar; beta-D-arabino-Hexulose; beta-Levulose; Fructose/Glucose/D-Galactose$D-Sorbitol/Galactitol/L-Iditol	2	5	0.03	D-Tagatose; lyxo-Hexulose/1,7-Dimethylxanthine; Paraxanthine/beta-D-Glucose/D-Fructose/alpha-D-Glucose//myo-Inositol/D-Hexose/beta-D-Galactose/L-Sorbose; L-xylo-Hexulose/alpha-D-Galactose/D-Glucose//Galactose/D-Mannose/beta-D-Fructose; beta-Fruit sugar; beta-D-arabino-Hexulose; beta-Levulose; Fructose/Glucose/D-Galactose$N-Acetyl-D-mannosamine; 2-Acetamido-2-deoxy-D-mannose/N-Acetylgalactosamine; N-Acetylchondrosamine; 2-Acetamido-2-deoxygalactose; GalNAc/N-Acetyl-D-glucosamine/N-Acetyl-D-galactosamine
Geraniol degradation	HILIC	0	0	0.46		0	0	0.37	

Glutamate metabolism	HILIC	3	5	0.02	DL-Glutamate; DL-Glutaminic acid; 2-Aminoglutaric acid; Glutamate/D-Glutamate; D-Glutamic acid; D-Glutaminic acid; D-2-Aminoglutaric acid/L-4-Hydroxyglutamate semialdehyde/L-Glutamate$D-Glutamine; D-2-Aminoglutaramic acid/L-Glutamine/3-Ureidoisobutyrate$beta-Alanine/L-Alanine/Sarcosine; N-Methylglycine/D-Alanine	0	5	0.37	
Glutathione Metabolism	HILIC	3	5	0.02	DL-Glutamate; DL-Glutaminic acid; 2-Aminoglutaric acid; Glutamate/D-Glutamate; D-Glutamic acid; D-Glutaminic acid; D-2-Aminoglutaric acid/L-4-Hydroxyglutamate semialdehyde/L-Glutamate$beta-Alanine/L-Alanine/Sarcosine; N-Methylglycine/D-Alanine$1-Pyrroline-4-hydroxy-2-carboxylate/5-Oxo-L-proline/L-1-Pyrroline-3-hydroxy-5-carboxylate/5-Oxoproline	0	5	0.37	
Glycerolipid metabolism	HILIC	0	0	0.46		0	0	0.37	
Glycerophospholipid metabolism	HILIC	4	14	0.13	sn-glycero-3-Phospho-1-inositol; 1-(sn-glycero-3-Phospho)-1D-myo-inositol$$$D-Tagatose; lyxo-Hexulose/1,7-Dimethylxanthine; Paraxanthine/beta-D-Glucose/D-Fructose/alpha-D-Glucose//myo-Inositol/D-Hexose/beta-D-Galactose/L-Sorbose; L-xylo-Hexulose/alpha-D-Galactose/D-Glucose//Galactose/D-Mannose/beta-D-Fructose; beta-Fruit sugar; beta-D-arabino-Hexulose; beta-Levulose; Fructose/Glucose/D-Galactose	2	14	0.22	D-Tagatose; lyxo-Hexulose/1,7-Dimethylxanthine; Paraxanthine/beta-D-Glucose/D-Fructose/alpha-D-Glucose//myo-Inositol/D-Hexose/beta-D-Galactose/L-Sorbose; L-xylo-Hexulose/alpha-D-Galactose/D-Glucose//Galactose/D-Mannose/beta-D-Fructose; beta-Fruit sugar; beta-D-arabino-Hexulose; beta-Levulose; Fructose/Glucose/D-Galactose$Deoxyribose/(R)-glycerol 1-acetate/Dihydropteridine; 6,7-Dihydropteridine
Glycine, serine, alanine and threonine metabolism	HILIC	4	21	0.32	DL-Glutamate; DL-Glutaminic acid; 2-Aminoglutaric acid; Glutamate/D-Glutamate; D-Glutamic acid; D-Glutaminic acid; D-2-Aminoglutaric acid/L-4-Hydroxyglutamate semialdehyde/L-Glutamate$beta-Alanine/L-Alanine/Sarcosine; N-Methylglycine/D-Alanine$D-Arginine; D-2-Amino-5-guanidinovaleric acid/L-Arginine$	3	21	0.19	$$
Glycolysis and Gluconeogenesis	HILIC	1	3	0.24	D-Tagatose; lyxo-Hexulose/1,7-Dimethylxanthine; Paraxanthine/beta-D-Glucose/D-Fructose/alpha-D-Glucose//myo-Inositol/D-Hexose/beta-D-Galactose/L-Sorbose; L-xylo-Hexulose/alpha-D-Galactose/D-Glucose//Galactose/D-Mannose/beta-D-Fructose; beta-Fruit sugar; beta-D-arabino-Hexulose; beta-Levulose; Fructose/Glucose/D-Galactose	1	3	0.13	D-Tagatose; lyxo-Hexulose/1,7-Dimethylxanthine; Paraxanthine/beta-D-Glucose/D-Fructose/alpha-D-Glucose//myo-Inositol/D-Hexose/beta-D-Galactose/L-Sorbose; L-xylo-Hexulose/alpha-D-Galactose/D-Glucose//Galactose/D-Mannose/beta-D-Fructose;

									beta-Fruit sugar; beta-D-arabino-Hexulose; beta-Levulose; Fructose/Glucose/D-Galactose
Glycosaminoglycan degradation	HILIC	0	0	0.46		0	0	0.37	
Glycosphingolipid biosynthesis - ganglioseries	HILIC	2	3	0.03	N-Acetyl-D-mannosamine; 2-Acetamido-2-deoxy-D-mannose/N-Acetylgalactosamine; N-Acetylchondrosamine; 2-Acetamido-2-deoxygalactose; GalNAc/N-Acetyl-D-glucosamine/N-Acetyl-D-galactosamine$D-Tagatose; lyxo-Hexulose/1,7-Dimethylxanthine; Paraxanthine/beta-D-Glucose/D-Fructose/alpha-D-Glucose//myo-Inositol/D-Hexose/beta-D-Galactose/L-Sorbose; L-xylo-Hexulose/alpha-D-Galactose/D-Glucose//Galactose/D-Mannose/beta-D-Fructose; beta-Fruit sugar; beta-D-arabino-Hexulose; beta-Levulose; Fructose/Glucose/D-Galactose	2	3	0.01	D-Tagatose; lyxo-Hexulose/1,7-Dimethylxanthine; Paraxanthine/beta-D-Glucose/D-Fructose/alpha-D-Glucose//myo-Inositol/D-Hexose/beta-D-Galactose/L-Sorbose; L-xylo-Hexulose/alpha-D-Galactose/D-Glucose//Galactose/D-Mannose/beta-D-Fructose; beta-Fruit sugar; beta-D-arabino-Hexulose; beta-Levulose; Fructose/Glucose/D-Galactose$N-Acetyl-D-mannosamine; 2-Acetamido-2-deoxy-D-mannose/N-Acetylgalactosamine; N-Acetylchondrosamine; 2-Acetamido-2-deoxygalactose; GalNAc/N-Acetyl-D-glucosamine/N-Acetyl-D-galactosamine
Glycosphingolipid biosynthesis - globoseries	HILIC	2	3	0.03	N-Acetyl-D-mannosamine; 2-Acetamido-2-deoxy-D-mannose/N-Acetylgalactosamine; N-Acetylchondrosamine; 2-Acetamido-2-deoxygalactose; GalNAc/N-Acetyl-D-glucosamine/N-Acetyl-D-galactosamine$D-Tagatose; lyxo-Hexulose/1,7-Dimethylxanthine; Paraxanthine/beta-D-Glucose/D-Fructose/alpha-D-Glucose//myo-Inositol/D-Hexose/beta-D-Galactose/L-Sorbose; L-xylo-Hexulose/alpha-D-Galactose/D-Glucose//Galactose/D-Mannose/beta-D-Fructose; beta-Fruit sugar; beta-D-arabino-Hexulose; beta-Levulose; Fructose/Glucose/D-Galactose	2	3	0.01	D-Tagatose; lyxo-Hexulose/1,7-Dimethylxanthine; Paraxanthine/beta-D-Glucose/D-Fructose/alpha-D-Glucose//myo-Inositol/D-Hexose/beta-D-Galactose/L-Sorbose; L-xylo-Hexulose/alpha-D-Galactose/D-Glucose//Galactose/D-Mannose/beta-D-Fructose; beta-Fruit sugar; beta-D-arabino-Hexulose; beta-Levulose; Fructose/Glucose/D-

									Galactose$N-Acetyl-D-mannosamine; 2-Acetamido-2-deoxy-D-mannose/N-Acetylgalactosamine; N-Acetylchondrosamine; 2-Acetamido-2-deoxygalactose; GalNAc/N-Acetyl-D-glucosamine/N-Acetyl-D-galactosamine
Glycosphingolipid biosynthesis - lactoseries	HILIC	0	1	0.46		0	1	0.37	
Glycosphingolipid biosynthesis - neolactoseries	HILIC	0	1	0.46		0	1	0.37	
Glycosphingolipid metabolism	HILIC	2	14	0.41	N-Acetyl-D-mannosamine; 2-Acetamido-2-deoxy-D-mannose/N-Acetylgalactosamine; N-Acetylchondrosamine; 2-Acetamido-2-deoxygalactose; GalNAc/N-Acetyl-D-glucosamine/N-Acetyl-D-galactosamine$D-Tagatose; lyxo-Hexulose/1,7-Dimethylxanthine; Paraxanthine/beta-D-Glucose/D-Fructose/alpha-D-Glucose//myo-Inositol/D-Hexose/beta-D-Galactose/L-Sorbose; L-xylo-Hexulose/alpha-D-Galactose/D-Glucose//Galactose/D-Mannose/beta-D-Fructose; beta-Fruit sugar; beta-D-arabino-Hexulose; beta-Levulose; Fructose/Glucose/D-Galactose	3	14	0.08	D-Tagatose; lyxo-Hexulose/1,7-Dimethylxanthine; Paraxanthine/beta-D-Glucose/D-Fructose/alpha-D-Glucose//myo-Inositol/D-Hexose/beta-D-Galactose/L-Sorbose; L-xylo-Hexulose/alpha-D-Galactose/D-Glucose//Galactose/D-Mannose/beta-D-Fructose; beta-Fruit sugar; beta-D-arabino-Hexulose; beta-Levulose; Fructose/Glucose/D-Galactose$Hexadecanoate (n-C16:0)$N-Acetyl-D-mannosamine; 2-Acetamido-2-deoxy-D-mannose/N-Acetylgalactosamine; N-Acetylchondrosamine; 2-Acetamido-2-deoxygalactose; GalNAc/N-Acetyl-D-glucosamine/N-Acetyl-D-galactosamine
Glycosylphosphatidylinositol(GPI)-anchor biosynthesis	HILIC	0	0	0.46		0	0	0.37	
Glyoxylate and Dicarboxylate Metabolism	HILIC	0	1	0.46		0	1	0.37	
Heparan sulfate biosynthesis	HILIC	0	0	0.46		0	0	0.37	

Heparan sulfate degradation	HILIC	2	3	0.03	N-Acetyl-D-mannosamine; 2-Acetamido-2-deoxy-D-mannose/N-Acetylgalactosamine; N-Acetylchondrosamine; 2-Acetamido-2-deoxygalactose; GalNAc/N-Acetyl-D-glucosamine/N-Acetyl-D-galactosamine$D-Tagatose; lyxo-Hexulose/1,7-Dimethylxanthine; Paraxanthine/beta-D-Glucose/D-Fructose/alpha-D-Glucose//myo-Inositol/D-Hexose/beta-D-Galactose/L-Sorbose; L-xylo-Hexulose/alpha-D-Galactose/D-Glucose//Galactose/D-Mannose/beta-D-Fructose; beta-Fruit sugar; beta-D-arabino-Hexulose; beta-Levulose; Fructose/Glucose/D-Galactose	2	3	0.01	D-Tagatose; lyxo-Hexulose/1,7-Dimethylxanthine; Paraxanthine/beta-D-Glucose/D-Fructose/alpha-D-Glucose//myo-Inositol/D-Hexose/beta-D-Galactose/L-Sorbose; L-xylo-Hexulose/alpha-D-Galactose/D-Glucose//Galactose/D-Mannose/beta-D-Fructose; beta-Fruit sugar; beta-D-arabino-Hexulose; beta-Levulose; Fructose/Glucose/D-Galactose$N-Acetyl-D-mannosamine; 2-Acetamido-2-deoxy-D-mannose/N-Acetylgalactosamine; N-Acetylchondrosamine; 2-Acetamido-2-deoxygalactose; GalNAc/N-Acetyl-D-glucosamine/N-Acetyl-D-galactosamine
Hexose phosphorylation	HILIC	4	5	0.00	alpha-Maltose; alpha-Malt sugar/1-alpha-D-Galactosyl-myo-inositol; 1-O-alpha-D-Galactosyl-D-myo-inositol; Galactinol/Isomaltose; Brachiose/Trehalose/Lactose/Sucrose/Maltose/Cellobiose/Epimelibiose/Melibiose/beta-D-Gal-(1->4)-D-Glc$D-Tagatose; lyxo-Hexulose/1,7-Dimethylxanthine; Paraxanthine/beta-D-Glucose/D-Fructose/alpha-D-Glucose//myo-Inositol/D-Hexose/beta-D-Galactose/L-Sorbose; L-xylo-Hexulose/alpha-D-Galactose/D-Glucose//Galactose/D-Mannose/beta-D-Fructose; beta-Fruit sugar; beta-D-arabino-Hexulose; beta-Levulose; Fructose/Glucose/D-Galactose$alpha-Maltose; alpha-Malt sugar/1-alpha-D-Galactosyl-myo-inositol; 1-O-alpha-D-Galactosyl-D-myo-inositol; Galactinol/Isomaltose; Brachiose/Trehalose/Lactose/Sucrose/Maltose/Cellobiose/Epimelibiose/Melibiose/beta-D-Gal-(1->4)-D-Glc$D-Sorbitol/Galactitol/L-Iditol	2	5	0.03	D-Tagatose; lyxo-Hexulose/1,7-Dimethylxanthine; Paraxanthine/beta-D-Glucose/D-Fructose/alpha-D-Glucose//myo-Inositol/D-Hexose/beta-D-Galactose/L-Sorbose; L-xylo-Hexulose/alpha-D-Galactose/D-Glucose//Galactose/D-Mannose/beta-D-Fructose; beta-Fruit sugar; beta-D-arabino-Hexulose; beta-Levulose; Fructose/Glucose/D-Galactose$Phenylpyruvate/enol-Phenylpyruvate/4-Coumarate; p-Coumaric acid; trans-4-Hydroxycinnamate; trans-p-Hydroxycinnamate; 4-Hydroxycinnamic acid; 4-Hydroxycinnamate/cis-2-Hydroxycinnamate; 2-Coumarinate

Histidine metabolism	HILIC	2	13	0.39	DL-Glutamate; DL-Glutaminic acid; 2-Aminoglutaric acid; Glutamate/D-Glutamate; D-Glutamic acid; D-Glutaminic acid; D-2-Aminoglutaric acid/L-4-Hydroxyglutamate semialdehyde/L-Glutamate$beta-Alanine/L-Alanine/Sarcosine; N-Methylglycine/D-Alanine	1	13	0.33	
Hyaluronan Metabolism	HILIC	1	2	0.17	N-Acetyl-D-mannosamine; 2-Acetamido-2-deoxy-D-mannose/N-Acetylgalactosamine; N-Acetylchondrosamine; 2-Acetamido-2-deoxygalactose; GalNAc/N-Acetyl-D-glucosamine/N-Acetyl-D-galactosamine	1	2	0.08	N-Acetyl-D-mannosamine; 2-Acetamido-2-deoxy-D-mannose/N-Acetylgalactosamine; N-Acetylchondrosamine; 2-Acetamido-2-deoxygalactose; GalNAc/N-Acetyl-D-glucosamine/N-Acetyl-D-galactosamine
Keratan sulfate biosynthesis	HILIC	0	1	0.46		0	1	0.37	
Keratan sulfate degradation	HILIC	2	4	0.06	N-Acetyl-D-mannosamine; 2-Acetamido-2-deoxy-D-mannose/N-Acetylgalactosamine; N-Acetylchondrosamine; 2-Acetamido-2-deoxygalactose; GalNAc/N-Acetyl-D-glucosamine/N-Acetyl-D-galactosamine$D-Tagatose; lyxo-Hexulose/1,7-Dimethylxanthine; Paraxanthine/beta-D-Glucose/D-Fructose/alpha-D-Glucose//myo-Inositol/D-Hexose/beta-D-Galactose/L-Sorbose; L-xylo-Hexulose/alpha-D-Galactose/D-Glucose//Galactose/D-Mannose/beta-D-Fructose; beta-Fruit sugar; beta-D-arabino-Hexulose; beta-Levulose; Fructose/Glucose/D-Galactose	2	4	0.02	D-Tagatose; lyxo-Hexulose/1,7-Dimethylxanthine; Paraxanthine/beta-D-Glucose/D-Fructose/alpha-D-Glucose//myo-Inositol/D-Hexose/beta-D-Galactose/L-Sorbose; L-xylo-Hexulose/alpha-D-Galactose/D-Glucose//Galactose/D-Mannose/beta-D-Fructose; beta-Fruit sugar; beta-D-arabino-Hexulose; beta-Levulose; Fructose/Glucose/D-Galactose$N-Acetyl-D-mannosamine; 2-Acetamido-2-deoxy-D-mannose/N-Acetylgalactosamine; N-Acetylchondrosamine; 2-Acetamido-2-deoxygalactose; GalNAc/N-Acetyl-D-glucosamine/N-Acetyl-D-galactosamine
Leukotriene metabolism	HILIC	1	5	0.35	DL-Glutamate; DL-Glutaminic acid; 2-Aminoglutaric acid; Glutamate/D-Glutamate; D-Glutamic acid; D-Glutaminic acid; D-2-Aminoglutaric acid/L-4-Hydroxyglutamate semialdehyde/L-Glutamate	1	5	0.20	
Limonene and pinene degradation	HILIC	1	4	0.30		0	4	0.37	
Linoleate metabolism	HILIC	1	15	0.45		0	15	0.37	
Lipoate metabolism	HILIC	0	2	0.46		0	2	0.37	

Lysine metabolism	HILIC	2	7	0.20	DL-Glutamate; DL-Glutaminic acid; 2-Aminoglutaric acid; Glutamate/D-Glutamate; D-Glutamic acid; D-Glutaminic acid; D-2-Aminoglutaric acid/L-4-Hydroxyglutamate semialdehyde/L-Glutamate$	0	7	0.37	
Methionine and cysteine metabolism	HILIC	3	18	0.38	DL-Glutamate; DL-Glutaminic acid; 2-Aminoglutaric acid; Glutamate/D-Glutamate; D-Glutamic acid; D-Glutaminic acid; D-2-Aminoglutaric acid/L-4-Hydroxyglutamate semialdehyde/L-Glutamate$$	1	18	0.36	
Mono-unsaturated fatty acid beta-oxidation	HILIC	0	0	0.46		0	0	0.37	
N-Glycan biosynthesis	HILIC	1	2	0.17	D-Tagatose; lyxo-Hexulose/1,7-Dimethylxanthine; Paraxanthine/beta-D-Glucose/D-Fructose/alpha-D-Glucose//myo-Inositol/D-Hexose/beta-D-Galactose/L-Sorbose; L-xylo-Hexulose/alpha-D-Galactose/D-Glucose//Galactose/D-Mannose/beta-D-Fructose; beta-Fruit sugar; beta-D-arabino-Hexulose; beta-Levulose; Fructose/Glucose/D-Galactose	1	2	0.08	D-Tagatose; lyxo-Hexulose/1,7-Dimethylxanthine; Paraxanthine/beta-D-Glucose/D-Fructose/alpha-D-Glucose//myo-Inositol/D-Hexose/beta-D-Galactose/L-Sorbose; L-xylo-Hexulose/alpha-D-Galactose/D-Glucose//Galactose/D-Mannose/beta-D-Fructose; beta-Fruit sugar; beta-D-arabino-Hexulose; beta-Levulose; Fructose/Glucose/D-Galactose
N-Glycan Degradation	HILIC	2	2	0.01	N-Acetyl-D-mannosamine; 2-Acetamido-2-deoxy-D-mannose/N-Acetylgalactosamine; N-Acetylchondrosamine; 2-Acetamido-2-deoxygalactose; GalNAc/N-Acetyl-D-glucosamine/N-Acetyl-D-galactosamine$D-Tagatose; lyxo-Hexulose/1,7-Dimethylxanthine; Paraxanthine/beta-D-Glucose/D-Fructose/alpha-D-Glucose//myo-Inositol/D-Hexose/beta-D-Galactose/L-Sorbose; L-xylo-Hexulose/alpha-D-Galactose/D-Glucose//Galactose/D-Mannose/beta-D-Fructose; beta-Fruit sugar; beta-D-arabino-Hexulose; beta-Levulose; Fructose/Glucose/D-Galactose	2	2	0.00	D-Tagatose; lyxo-Hexulose/1,7-Dimethylxanthine; Paraxanthine/beta-D-Glucose/D-Fructose/alpha-D-Glucose//myo-Inositol/D-Hexose/beta-D-Galactose/L-Sorbose; L-xylo-Hexulose/alpha-D-Galactose/D-Glucose//Galactose/D-Mannose/beta-D-Fructose; beta-Fruit sugar; beta-D-arabino-Hexulose; beta-Levulose; Fructose/Glucose/D-Galactose$N-Acetyl-D-mannosamine; 2-Acetamido-2-deoxy-D-mannose/N-Acetylgalactosamine; N-Acetylchondrosamine; 2-Acetamido-2-deoxygalactose; GalNAc/N-Acetyl-D-glucosamine/N-

									Acetyl-D-galactosamine
Nitrogen metabolism	HILIC	3	3	0.00	DL-Glutamate; DL-Glutaminic acid; 2-Aminoglutaric acid; Glutamate/D-Glutamate; D-Glutamic acid; D-Glutaminic acid; D-2-Aminoglutaric acid/L-4-Hydroxyglutamate semialdehyde/L-Glutamate$L-Aspartate/D-Aspartate$D-Glutamine; D-2-Aminoglutaramic acid/L-Glutamine/3-Ureidoisobutyrate	0	3	0.37	
Nucleotide Sugar Metabolism	HILIC	0	0	0.46		0	0	0.37	
O-Glycan biosynthesis	HILIC	0	1	0.46		0	1	0.37	
Omega-3 fatty acid metabolism	HILIC	0	6	0.46		1	6	0.23	
Omega-6 fatty acid metabolism	HILIC	0	4	0.46		1	4	0.17	
Parathio degradation	HILIC	1	1	0.08	4-Nitrophenol; p-Nitrophenol; PNP; Niphen; 4-Hydroxynitrobenzene	0	1	0.37	
Pentose and Glucuronate Interconversions	HILIC	0	2	0.46		1	2	0.08	
Pentose phosphate pathway	HILIC	1	2	0.17	D-Tagatose; lyxo-Hexulose/1,7-Dimethylxanthine; Paraxanthine/beta-D-Glucose/D-Fructose/alpha-D-Glucose//myo-Inositol/D-Hexose/beta-D-Galactose/L-Sorbose; L-xylo-Hexulose/alpha-D-Galactose/D-Glucose//Galactose/D-Mannose/beta-D-Fructose; beta-Fruit sugar; beta-D-arabino-Hexulose; beta-Levulose; Fructose/Glucose/D-Galactose	2	2	0.00	Deoxyribose/(R)-glycerol 1-acetate/Dihydropteridine; 6,7-Dihydropteridine$D-Tagatose; lyxo-Hexulose/1,7-Dimethylxanthine; Paraxanthine/beta-D-Glucose/D-Fructose/alpha-D-Glucose//myo-Inositol/D-Hexose/beta-D-Galactose/L-Sorbose; L-xylo-Hexulose/alpha-D-Galactose/D-Glucose//Galactose/D-Mannose/beta-D-Fructose; beta-Fruit sugar; beta-D-arabino-Hexulose; beta-Levulose; Fructose/Glucose/D-Galactose

Phosphatidylinositol phosphate metabolism	HILIC	4	8	0.02	sn-glycero-3-Phospho-1-inositol; 1-(sn-glycero-3-Phospho)-1D-myo-inositol$alpha-Maltose; alpha-Malt sugar/1-alpha-D-Galactosyl-myo-inositol; 1-O-alpha-D-Galactosyl-D-myo-inositol; Galactinol/Isomaltose; Brachiose/Trehalose/Lactose/Sucrose/Maltose/Cellobiose/Epimelibiose/Melibiose/beta-D-Gal-(1->4)-D-Glc$alpha-Maltose; alpha-Malt sugar/1-alpha-D-Galactosyl-myo-inositol; 1-O-alpha-D-Galactosyl-D-myo-inositol; Galactinol/Isomaltose; Brachiose/Trehalose/Lactose/Sucrose/Maltose/Cellobiose/Epimelibiose/Melibiose/beta-D-Gal-(1->4)-D-Glc$D-Tagatose; lyxo-Hexulose/1,7-Dimethylxanthine; Paraxanthine/beta-D-Glucose/D-Fructose/alpha-D-Glucose//myo-Inositol/D-Hexose/beta-D-Galactose/L-Sorbose; L-xylo-Hexulose/alpha-D-Galactose/D-Glucose//Galactose/D-Mannose/beta-D-Fructose; beta-Fruit sugar; beta-D-arabino-Hexulose; beta-Levulose; Fructose/Glucose/D-Galactose	2	8	0.09	D-Tagatose; lyxo-Hexulose/1,7-Dimethylxanthine; Paraxanthine/beta-D-Glucose/D-Fructose/alpha-D-Glucose//myo-Inositol/D-Hexose/beta-D-Galactose/L-Sorbose; L-xylo-Hexulose/alpha-D-Galactose/D-Glucose//Galactose/D-Mannose/beta-D-Fructose; beta-Fruit sugar; beta-D-arabino-Hexulose; beta-Levulose; Fructose/Glucose/D-Galactose$Hexadecanoate (n-C16:0)
Phytanic acid peroxisomal oxidation	HILIC	0	0	0.46		0	0	0.37	
Polyunsaturated fatty acid biosynthesis	HILIC	0	3	0.46		1	3	0.13	
Porphyrin metabolism	HILIC	0	0	0.46		0	0	0.37	
Propanoate metabolism	HILIC	0	2	0.46		0	2	0.37	
Prostaglandin formation from arachidonate	HILIC	0	4	0.46		0	4	0.37	
Prostaglandin formation from dihomo gama-linoleic acid	HILIC	0	0	0.46		0	0	0.37	
Proteoglycan biosynthesis	HILIC	0	1	0.46		0	1	0.37	
Purine metabolism	HILIC	5	11	0.02	/Hypoxanthine$DL-Glutamate; DL-Glutaminic acid; 2-Aminoglutaric acid; Glutamate/D-Glutamate; D-Glutamic acid; D-Glutaminic acid; D-2-Aminoglutaric acid/L-4-Hydroxyglutamate semialdehyde/L-Glutamate$D-Glutamine; D-2-Aminoglutaramic acid/L-Glutamine/3-Ureidoisobutyrate$L-Aspartate/D-Aspartate$Deoxyadenosine/5'-Deoxyadenosine	2	11	0.15	$
Putative anti-Inflammatory metabolites formation from EPA	HILIC	1	2	0.17	DL-Glutamate; DL-Glutaminic acid; 2-Aminoglutaric acid; Glutamate/D-Glutamate; D-Glutamic acid; D-Glutaminic acid; D-2-Aminoglutaric acid/L-4-Hydroxyglutamate semialdehyde/L-Glutamate	0	2	0.37	
Pyrimidine metabolism	HILIC	6	14	0.01	5,6-Dihydrothymine$DL-Glutamate; DL-Glutaminic acid; 2-Aminoglutaric acid; Glutamate/D-Glutamate; D-Glutamic acid; D-Glutaminic acid; D-2-Aminoglutaric acid/L-4-Hydroxyglutamate semialdehyde/L-Glutamate$5,6-Dihydrothymine$D-Glutamine; D-2-Aminoglutaramic acid/L-Glutamine/3-Ureidoisobutyrate$L-Aspartate/D-Aspartate$beta-Alanine/L-Alanine/Sarcosine; N-Methylglycine/D-Alanine	3	14	0.08	$Deoxyribose/(R)-glycerol 1-acetate/Dihydropteridine; 6,7-Dihydropteridine$

Pyruvate Metabolism	HILIC	0	0	0.46		0	0	0.37	
R Group Synthesis	HILIC	0	0	0.46		0	0	0.37	
ROS Detoxification	HILIC	0	0	0.46		0	0	0.37	
Saturated fatty acids beta-oxidation	HILIC	1	4	0.30		1	4	0.17	Hexadecanoate (n-C16:0)
Selenoamino acid metabolism	HILIC	2	5	0.12	$beta-Alanine/L-Alanine/Sarcosine; N-Methylglycine/D-Alanine	0	5	0.37	
Sialic acid metabolism	HILIC	4	5	0.00	N-Acetyl-D-mannosamine; 2-Acetamido-2-deoxy-D-mannose/N-Acetylgalactosamine; N-Acetylchondrosamine; 2-Acetamido-2-deoxygalactose; GalNAc/N-Acetyl-D-glucosamine/N-Acetyl-D-galactosamine$alpha-Maltose; alpha-Malt sugar/1-alpha-D-Galactosyl-myo-inositol; 1-O-alpha-D-Galactosyl-D-myo-inositol; Galactinol/Isomaltose; Brachiose/Trehalose/Lactose/Sucrose/Maltose/Cellobiose/Epimelibiose/Melibiose/beta-D-Gal-(1->4)-D-Glc$alpha-Maltose; alpha-Malt sugar/1-alpha-D-Galactosyl-myo-inositol; 1-O-alpha-D-Galactosyl-D-myo-inositol; Galactinol/Isomaltose; Brachiose/Trehalose/Lactose/Sucrose/Maltose/Cellobiose/Epimelibiose/Melibiose/beta-D-Gal-(1->4)-D-Glc$D-Tagatose; lyxo-Hexulose/1,7-Dimethylxanthine; Paraxanthine/beta-D-Glucose/D-Fructose/alpha-D-Glucose//myo-Inositol/D-Hexose/beta-D-Galactose/L-Sorbose; L-xylo-Hexulose/alpha-D-Galactose/D-Glucose//Galactose/D-Mannose/beta-D-Fructose; beta-Fruit sugar; beta-D-arabino-Hexulose; beta-Levulose; Fructose/Glucose/D-Galactose	2	5	0.03	D-Tagatose; lyxo-Hexulose/1,7-Dimethylxanthine; Paraxanthine/beta-D-Glucose/D-Fructose/alpha-D-Glucose//myo-Inositol/D-Hexose/beta-D-Galactose/L-Sorbose; L-xylo-Hexulose/alpha-D-Galactose/D-Glucose//Galactose/D-Mannose/beta-D-Fructose; beta-Fruit sugar; beta-D-arabino-Hexulose; beta-Levulose; Fructose/Glucose/D-Galactose$N-Acetyl-D-mannosamine; 2-Acetamido-2-deoxy-D-mannose/N-Acetylgalactosamine; N-Acetylchondrosamine; 2-Acetamido-2-deoxygalactose; GalNAc/N-Acetyl-D-glucosamine/N-Acetyl-D-galactosamine
Sphingolipid metabolism	HILIC	0	0	0.46		0	0	0.37	
Squalene and cholesterol biosynthesis	HILIC	1	3	0.24		0	3	0.37	

Starch and Sucrose Metabolism	HILIC	3	4	0.01	alpha-Maltose; alpha-Malt sugar/1-alpha-D-Galactosyl-myo-inositol; 1-O-alpha-D-Galactosyl-D-myo-inositol; Galactinol/Isomaltose; Brachiose/Trehalose/Lactose/Sucrose/Maltose/Cellobiose/Epimelibiose/Melibiose/beta-D-Gal-(1->4)-D-Glc$D-Tagatose; lyxo-Hexulose/1,7-Dimethylxanthine; Paraxanthine/beta-D-Glucose/D-Fructose/alpha-D-Glucose//myo-Inositol/D-Hexose/beta-D-Galactose/L-Sorbose; L-xylo-Hexulose/alpha-D-Galactose/D-Glucose//Galactose/D-Mannose/beta-D-Fructose; beta-Fruit sugar; beta-D-arabino-Hexulose; beta-Levulose; Fructose/Glucose/D-Galactose$alpha-Maltose; alpha-Malt sugar/1-alpha-D-Galactosyl-myo-inositol; 1-O-alpha-D-Galactosyl-D-myo-inositol; Galactinol/Isomaltose; Brachiose/Trehalose/Lactose/Sucrose/Maltose/Cellobiose/Epimelibiose/Melibiose/beta-D-Gal-(1->4)-D-Glc	1	4	0.17	D-Tagatose; lyxo-Hexulose/1,7-Dimethylxanthine; Paraxanthine/beta-D-Glucose/D-Fructose/alpha-D-Glucose//myo-Inositol/D-Hexose/beta-D-Galactose/L-Sorbose; L-xylo-Hexulose/alpha-D-Galactose/D-Glucose//Galactose/D-Mannose/beta-D-Fructose; beta-Fruit sugar; beta-D-arabino-Hexulose; beta-Levulose; Fructose/Glucose/D-Galactose
TCA cycle	HILIC	0	2	0.46		0	2	0.37	
Trihydroxycoprostanoyl-CoA beta-oxidation	HILIC	0	0	0.46		0	0	0.37	
Tryptophan metabolism	HILIC	6	21	0.10	$Formyl-N-acetyl-5-methoxykynurenamine/alpha-N-Phenylacetyl-L-glutamine$DL-Glutamate; DL-Glutaminic acid; 2-Aminoglutaric acid; Glutamate/D-Glutamate; D-Glutamic acid; D-Glutaminic acid; D-2-Aminoglutaric acid/L-4-Hydroxyglutamate semialdehyde/L-Glutamate$beta-Alanine/L-Alanine/Sarcosine; N-Methylglycine/D-Alanine$L-Tryptophan/D-Tryptophan$D-Tagatose; lyxo-Hexulose/1,7-Dimethylxanthine; Paraxanthine/beta-D-Glucose/D-Fructose/alpha-D-Glucose//myo-Inositol/D-Hexose/beta-D-Galactose/L-Sorbose; L-xylo-Hexulose/alpha-D-Galactose/D-Glucose//Galactose/D-Mannose/beta-D-Fructose; beta-Fruit sugar; beta-D-arabino-Hexulose; beta-Levulose; Fructose/Glucose/D-Galactose	2	21	0.31	D-Tagatose; lyxo-Hexulose/1,7-Dimethylxanthine; Paraxanthine/beta-D-Glucose/D-Fructose/alpha-D-Glucose//myo-Inositol/D-Hexose/beta-D-Galactose/L-Sorbose; L-xylo-Hexulose/alpha-D-Galactose/D-Glucose//Galactose/D-Mannose/beta-D-Fructose; beta-Fruit sugar; beta-D-arabino-Hexulose; beta-Levulose; Fructose/Glucose/D-Galactose$
Tyrosine metabolism	HILIC	4	28	0.42	DL-Glutamate; DL-Glutaminic acid; 2-Aminoglutaric acid; Glutamate/D-Glutamate; D-Glutamic acid; D-Glutaminic acid; D-2-Aminoglutaric acid/L-4-Hydroxyglutamate semialdehyde/L-Glutamate$beta-Alanine/L-Alanine/Sarcosine; N-Methylglycine/D-Alanine$Formyl-N-acetyl-5-methoxykynurenamine/alpha-N-Phenylacetyl-L-glutamine$D-Glutamine; D-2-Aminoglutaramic acid/L-Glutamine/3-Ureidoisobutyrate	3	28	0.29	$Phenylpyruvate/enol-Phenylpyruvate/4-Coumarate; p-Coumaric acid; trans-4-Hydroxycinnamate; trans-p-Hydroxycinnamate; 4-Hydroxycinnamic acid; 4-Hydroxycinnamate/cis-2-Hydroxycinnamate; 2-Coumarinate$

Ubiquinone Biosynthesis	HILIC	0	2	0.46		1	2	0.08	Phenylpyruvate/enol-Phenylpyruvate/4-Coumarate; p-Coumaric acid; trans-4-Hydroxycinnamate; trans-p-Hydroxycinnamate; 4-Hydroxycinnamic acid; 4-Hydroxycinnamate/cis-2-Hydroxycinnamate; 2-Coumarinate
Urea cycle/amino group metabolism	HILIC	7	26	0.11	beta-Alanine/L-Alanine/Sarcosine; N-Methylglycine/D-Alanine$DL-Glutamate; DL-Glutaminic acid; 2-Aminoglutaric acid; Glutamate/D-Glutamate; D-Glutamic acid; D-Glutaminic acid; D-2-Aminoglutaric acid/L-4-Hydroxyglutamate semialdehyde/L-Glutamate$$D-Arginine; D-2-Amino-5-guanidinovaleric acid/L-Arginine$Deoxyadenosine/5'-Deoxyadenosine$L-Aspartate/D-Aspartate$L-Citrulline; 2-Amino-5-ureidovaleric acid; Citrulline	3	26	0.27	$$
Valine, leucine and isoleucine degradation	HILIC	1	7	0.40	DL-Glutamate; DL-Glutaminic acid; 2-Aminoglutaric acid; Glutamate/D-Glutamate; D-Glutamic acid; D-Glutaminic acid; D-2-Aminoglutaric acid/L-4-Hydroxyglutamate semialdehyde/L-Glutamate	0	7	0.37	
Vitamin A (retinol) metabolism	HILIC	1	6	0.38		0	6	0.37	
Vitamin B1 (thiamin) metabolism	HILIC	1	1	0.08		0	1	0.37	
Vitamin B12 (cyanocobalamin) metabolism	HILIC	0	0	0.46		0	0	0.37	
Vitamin B2 (riboflavin) metabolism	HILIC	0	1	0.46		1	1	0.03	Riboflavin
Vitamin B3 (nicotinate and nicotinamide) metabolism	HILIC	4	7	0.01	DL-Glutamate; DL-Glutaminic acid; 2-Aminoglutaric acid; Glutamate/D-Glutamate; D-Glutamic acid; D-Glutaminic acid; D-2-Aminoglutaric acid/L-4-Hydroxyglutamate semialdehyde/L-Glutamate$D-Glutamine; D-2-Aminoglutaramic acid/L-Glutamine/3-Ureidoisobutyrate$D-Arginine; D-2-Amino-5-guanidinovaleric acid/L-Arginine$L-Aspartate/D-Aspartate	0	7	0.37	
Vitamin B5 - CoA biosynthesis from pantothenate	HILIC	2	3	0.03	Pantetheine; (R)-Pantetheine$(R)-Pantothenate	0	3	0.37	
Vitamin B6 (pyridoxine) metabolism	HILIC	0	1	0.46		0	1	0.37	
Vitamin B9 (folate) metabolism	HILIC	1	2	0.17	DL-Glutamate; DL-Glutaminic acid; 2-Aminoglutaric acid; Glutamate/D-Glutamate; D-Glutamic acid; D-Glutaminic acid; D-2-Aminoglutaric acid/L-4-Hydroxyglutamate semialdehyde/L-Glutamate	0	2	0.37	
Vitamin D	HILIC	0	1	0.46		0	1	0.37	
Vitamin D3 (cholecalciferol) metabolism	HILIC	0	3	0.46		0	3	0.37	
Vitamin E metabolism	HILIC	1	5	0.35		0	5	0.37	
Vitamin H (biotin) metabolism	HILIC	0	1	0.46		0	1	0.37	

Vitamin K metabolism	HILIC	0	1	0.46		0	1	0.37	
Xenobiotics metabolism	HILIC	5	12	0.02	4-Nitrophenol; p-Nitrophenol; PNP; Niphen; 4-Hydroxynitrobenzene$$$$	4	12	0.01	$$$Hexadecanoate (n-C16:0)
1- and 2-Methylnaphthalene degradation	C18	0	0	0.50		0	0	0.37	
3-Chloroacrylic acid degradation	C18	0	0	0.50		0	0	0.37	
3-oxo-10R-octadecatrienoate beta-oxidation	C18	0	0	0.50		0	0	0.37	
Alanine and Aspartate Metabolism	C18	6	10	0.02	D-Glutamate; D-Glutamic acid; D-Glutaminic acid; D-2-Aminoglutaric acid/L-4-Hydroxyglutamate semialdehyde/L-Glutamate$2-Oxoglutarate$L-Citrulline; 2-Amino-5-ureidovaleric acid; Citrulline$Sarcosine; N-Methylglycine/D-Alanine/L-Alanine/beta-Alanine$D-Glutamine; D-2-Aminoglutaramic acid/L-Glutamine/3-Ureidoisobutyrate$D-Arginine; D-2-Amino-5-guanidinovaleric acid/L-Arginine	2	10	0.24	$D-Glutamine; D-2-Aminoglutaramic acid/L-Glutamine/3-Ureidoisobutyrate
Alkaloid biosynthesis II	C18	1	1	0.15		0	1	0.37	
Aminosugars metabolism	C18	4	7	0.05	D-Glutamate; D-Glutamic acid; D-Glutaminic acid; D-2-Aminoglutaric acid/L-4-Hydroxyglutamate semialdehyde/L-Glutamate$D-Glucosamine$D-Glutamine; D-2-Aminoglutaramic acid/L-Glutamine/3-Ureidoisobutyrate$D-Hexose 6-phosphate/D-Glucose 1-phosphate/beta-D-Fructose 6-phosphate/D-Fructose 6-phosphate/Fructose 1-phosphate/alpha-D-Hexose 1-phosphate/beta-D-glucose 6-phosphate/1D-myo-Inositol 1-phosphate/alpha-D-Hexose 6-phosphate/D-Mannose 1-phosphate/D-Glucose 6-phosphate; Glucose 6-phosphate; Robison ester/Dolichyl phosphate D-mannose/1D-myo-Inositol 3-phosphate/D-Tagatose 6-phosphate/D-Fructose 1-phosphate/alpha-D-Glucose 6-phosphate/1D-myo-Inositol 4-phosphate/alpha-D-Galactose 1-phosphate/D-Mannose 6-phosphate/sorbitol 3-phosphate	3	7	0.03	N-Acetylneuraminate; N-Acetylneuraminic acid; 5-Acetamido-3,5-dideoxy-D-glycero-D-galacto-2-nonulosonic acid; Neu5Ac$D-Hexose 6-phosphate/D-Glucose 1-phosphate/beta-D-Fructose 6-phosphate/D-Fructose 6-phosphate/Fructose 1-phosphate/alpha-D-Hexose 1-phosphate/beta-D-glucose 6-phosphate/1D-myo-Inositol 1-phosphate/alpha-D-Hexose 6-phosphate/D-Mannose 1-phosphate/D-Glucose 6-phosphate; Glucose 6-phosphate; Robison ester/Dolichyl phosphate D-mannose/1D-myo-Inositol 3-phosphate/D-Tagatose 6-phosphate/D-Fructose 1-phosphate/alpha-D-Glucose 6-phosphate/1D-myo-Inositol 4-phosphate/alpha-D-Galactose 1-phosphate/D-Mannose 6-phosphate/sorbitol 3-phosphate$D-Glutamine; D-2-Aminoglutaramic acid/L-Glutamine/3-Ureidoisobutyrate
Androgen and estrogen biosynthesis and metabolism	C18	1	5	0.45	Sulfate	0	5	0.37	

Arachidonic acid metabolism	C18	1	10	0.50	D-Glutamate; D-Glutamic acid; D-Glutaminic acid; D-2-Aminoglutaric acid/L-4-Hydroxyglutamate semialdehyde/L-Glutamate	0	10	0.37	
Arginine and Proline Metabolism	C18	8	13	0.01	D-Glutamate; D-Glutamic acid; D-Glutaminic acid; D-2-Aminoglutaric acid/L-4-Hydroxyglutamate semialdehyde/L-Glutamate$2-Oxoglutarate$L-Citrulline; 2-Amino-5-ureidovaleric acid; Citrulline$D-Arginine; D-2-Amino-5-guanidinovaleric acid/L-Arginine$5-Aminolevulinate; 5-Amino-4-oxopentanoate; 5-Amino-4-oxovaleric acid/cis-4-Hydroxy-D-proline/2-Amino-4-oxopentanoic acid; 2-Amino-4-oxopentanoate/L-Glutamate 5-semialdehyde; L-Glutamate gamma-semialdehyde/trans-4-Hydroxy-L-proline/5-Amino-2-oxopentanoic acid; 5-Amino-2-oxopentanoate; 2-Oxo-5-amino-pentanoate; 2-Oxo-5-aminopentanoate; alpha-Keto-delta-aminopentanoate; 2-Oxo-5-aminovalerate$D-Proline/acetamidopropanal/L-Proline$N4-Acetylaminobutanal/L-Pipecolate; Pipecolinic acid; Pipecolic acid; 2-Piperidinecarboxylic acid$D-Glutamine; D-2-Aminoglutaramic acid/L-Glutamine/3-Ureidoisobutyrate	3	13	0.17	$$D-Glutamine; D-2-Aminoglutaramic acid/L-Glutamine/3-Ureidoisobutyrate
Ascorbate (Vitamin C) and Aldarate Metabolism	C18	7	14	0.04	D-Gluconate/L-Gulonate$3-Keto-beta-D-galactose/L-Gulono-1,4-lactone; L-Gulono-gamma-lactone; gamma-Gulonolactone; L-Gulonic acid gamma-lactone; L-Gulonolactone/D-Glucono-1,5-lactone; Gluconic lactone; Gluconic acid lactone; 1,5-Gluconolactone; delta-Gluconolactone; D-Gluconolactone; Gluconolactone$Isocitrate/Citrate/$D-glucurono-6,3-lactone/L-Ascorbate/D-glucurono-6,3-lactone$Dehydroascorbate/Dehydroascorbate/cis-Aconitate; cis-Aconitic acid$Dihydrolipoate; Dihydrolipoic acid; Dihydrothioctic acid$3-Dehydro-L-gulonate/L-Iduronic acid/D-Glucuronate	0	14	0.37	

Aspartate and asparagine metabolism	C18	11	17	0.00	D-Glutamate; D-Glutamic acid; D-Glutaminic acid; D-2-Aminoglutaric acid/L-4-Hydroxyglutamate semialdehyde/L-Glutamate$2-Oxoglutarate$L-Citrulline; 2-Amino-5-ureidovaleric acid; Citrulline$3-Aminoisobutyric acid; 3-Aminoisobutanoate; 3-Amino-2-methylpropanoate/N,N-Dimethylglycine; Dimethylglycine/D-3-Amino-isobutanoate/(S)-2-Aminobutanoate; (S)-2-Aminobutanoic acid; (S)-2-Aminobutyric acid/4-Aminobutanoate/L-3-Amino-isobutanoate$(S)-3-Hydroxyisobutyrate/(R)-3-Hydroxybutanoate; (R)-3-Hydroxybutanoic acid; (R)-3-Hydroxybutyric acid/(S)-3-Hydroxybutanoate/4-Hydroxybutanoic acid; 4-Hydroxybutanoate; 4-Hydroxybutyric acid/2-Hydroxybutyrate$N4-Acetylaminobutanal/L-Pipecolate; Pipecolinic acid; Pipecolic acid; 2-Piperidinecarboxylic acid$$5-Aminolevulinate; 5-Amino-4-oxopentanoate; 5-Amino-4-oxovaleric acid/cis-4-Hydroxy-D-proline/2-Amino-4-oxopentanoic acid; 2-Amino-4-oxopentanoate/L-Glutamate 5-semialdehyde; L-Glutamate gamma-semialdehyde/trans-4-Hydroxy-L-proline/5-Amino-2-oxopentanoic acid; 5-Amino-2-oxopentanoate; 2-Oxo-5-amino-pentanoate; 2-Oxo-5-aminopentanoate; alpha-Keto-delta-aminopentanoate; 2-Oxo-5-aminovalerate$D-Proline/acetamidopropanal/L-Proline$D-Arginine; D-2-Amino-5-guanidinovaleric acid/L-Arginine$D-Glutamine; D-2-Aminoglutaramic acid/L-Glutamine/3-Ureidoisobutyrate	4	17	0.14	$$D-Glutamine; D-2-Aminoglutaramic acid/L-Glutamine/3-Ureidoisobutyrate$(S)-3-Hydroxyisobutyrate/(R)-3-Hydroxybutanoate; (R)-3-Hydroxybutanoic acid; (R)-3-Hydroxybutyric acid/(S)-3-Hydroxybutanoate/4-Hydroxybutanoic acid; 4-Hydroxybutanoate; 4-Hydroxybutyric acid/2-Hydroxybutyrate
Atrazine degradation	C18	0	0	0.50		0	0	0.37	
Benzoate degradation via CoA ligation	C18	0	1	0.50		0	1	0.37	
Beta-Alanine metabolism	C18	5	7	0.01	D-Glutamate; D-Glutamic acid; D-Glutaminic acid; D-2-Aminoglutaric acid/L-4-Hydroxyglutamate semialdehyde/L-Glutamate$3-Aminoisobutyric acid; 3-Aminoisobutanoate; 3-Amino-2-methylpropanoate/N,N-Dimethylglycine; Dimethylglycine/D-3-Amino-isobutanoate/(S)-2-Aminobutanoate; (S)-2-Aminobutanoic acid; (S)-2-Aminobutyric acid/4-Aminobutanoate/L-3-Amino-isobutanoate$Dihydroxyacetone/D-Lactate/L-Lactate/3-Hydroxypropanoate; 3-Hydroxypropanoic acid; 3-Hydroxypropionate; 3-Hydroxypropionic acid; Hydracrylic acid/D-Glyceraldehyde$Sarcosine; N-Methylglycine/D-Alanine/L-Alanine/beta-Alanine$2-Oxoglutarate	2	7	0.15	Dihydroxyacetone/D-Lactate/L-Lactate/3-Hydroxypropanoate; 3-Hydroxypropanoic acid; 3-Hydroxypropionate; 3-Hydroxypropionic acid; Hydracrylic acid/D-Glyceraldehyde$
Bile acid biosynthesis	C18	3	7	0.19	$$	2	7	0.15	$
Biopterin metabolism	C18	1	2	0.28	L-Tyrosine	1	2	0.14	L-Tyrosine
Blood Group Biosynthesis	C18	0	1	0.50		0	1	0.37	

Butanoate metabolism	C18	5	5	0.00	D-Glutamate; D-Glutamic acid; D-Glutaminic acid; D-2-Aminoglutaric acid/L-4-Hydroxyglutamate semialdehyde/L-Glutamate$2-Oxoglutarate$(S)-3-Hydroxyisobutyrate/(R)-3-Hydroxybutanoate; (R)-3-Hydroxybutanoic acid; (R)-3-Hydroxybutyric acid/(S)-3-Hydroxybutanoate/4-Hydroxybutanoic acid; 4-Hydroxybutanoate; 4-Hydroxybutyric acid/2-Hydroxybutyrate$3-Aminoisobutyric acid; 3-Aminoisobutanoate; 3-Amino-2-methylpropanoate/N,N-Dimethylglycine; Dimethylglycine/D-3-Amino-isobutanoate/(S)-2-Aminobutanoate; (S)-2-Aminobutanoic acid; (S)-2-Aminobutyric acid/4-Aminobutanoate/L-3-Amino-isobutanoate$2-Acetolactate/(S)-2-Acetolactate; (S)-2-Hydroxy-2-methyl-3-oxobutanoate	2	5	0.09	2-Acetolactate/(S)-2-Acetolactate; (S)-2-Hydroxy-2-methyl-3-oxobutanoate$(S)-3-Hydroxyisobutyrate/(R)-3-Hydroxybutanoate; (R)-3-Hydroxybutanoic acid; (R)-3-Hydroxybutyric acid/(S)-3-Hydroxybutanoate/4-Hydroxybutanoic acid; 4-Hydroxybutanoate; 4-Hydroxybutyric acid/2-Hydroxybutyrate
C21-steroid hormone biosynthesis and metabolism	C18	4	8	0.09	$$Sulfate$	0	8	0.37	
C5-Branched dibasic acid metabolism	C18	0	1	0.50		0	1	0.37	
Caffeine metabolism	C18	4	5	0.01	1,3,7-Trimethyluric acid/sedoheptulose$D-Tagatose; lyxo-Hexulose/1,7-Dimethylxanthine; Paraxanthine/beta-D-Glucose/D-Fructose/alpha-D-Glucose//D-Glucose/myo-Inositol/D-Hexose/beta-D-Galactose/alpha-D-Galactose/L-Sorbose; L-xylo-Hexulose//Galactose/D-Mannose/beta-D-Fructose; beta-Fruit sugar; beta-D-arabino-Hexulose; beta-Levulose; Fructose/Glucose/D-Galactose$5-Acetylamino-6-formylamino-3-methyluracil; AFMU$/1,7-Dimethyluric acid	3	5	0.01	D-Tagatose; lyxo-Hexulose/1,7-Dimethylxanthine; Paraxanthine/beta-D-Glucose/D-Fructose/alpha-D-Glucose//D-Glucose/myo-Inositol/D-Hexose/beta-D-Galactose/alpha-D-Galactose/L-Sorbose; L-xylo-Hexulose//Galactose/D-Mannose/beta-D-Fructose; beta-Fruit sugar; beta-D-arabino-Hexulose; beta-Levulose; Fructose/Glucose/D-Galactose$5-Acetylamino-6-formylamino-3-methyluracil; AFMU$1,3,7-Trimethyluric acid/sedoheptulose
Carbon fixation	C18	2	3	0.11	D-Hexose 6-phosphate/D-Glucose 1-phosphate/beta-D-Fructose 6-phosphate/D-Fructose 6-phosphate/Fructose 1-phosphate/alpha-D-Hexose 1-phosphate/beta-D-glucose 6-phosphate/1D-myo-Inositol 1-phosphate/alpha-D-Hexose 6-phosphate/D-Mannose 1-phosphate/D-Glucose 6-phosphate; Glucose 6-phosphate; Robison ester/Dolichyl phosphate D-mannose/1D-myo-Inositol 3-phosphate/D-Tagatose 6-phosphate/D-Fructose 1-phosphate/alpha-D-Glucose 6-phosphate/1D-myo-Inositol 4-phosphate/alpha-D-Galactose 1-phosphate/D-Mannose 6-phosphate/sorbitol 3-phosphate$L-Ribulose 5-phosphate/alpha-D-Ribose 5-phosphate/alpha-D-Ribose 5-phosphate/alpha-D-Ribose 1-phosphate/alpha-D-Ribose 1-phosphate/D-Xylulose 5-phosphate/D-Ribulose 5-phosphate	1	3	0.20	D-Hexose 6-phosphate/D-Glucose 1-phosphate/beta-D-Fructose 6-phosphate/D-Fructose 6-phosphate/Fructose 1-phosphate/alpha-D-Hexose 1-phosphate/beta-D-glucose 6-phosphate/1D-myo-Inositol 1-phosphate/alpha-D-Hexose 6-phosphate/D-Mannose 1-phosphate/D-Glucose 6-phosphate; Glucose 6-phosphate; Robison ester/Dolichyl phosphate D-

									mannose/1D-myo-Inositol 3-phosphate/D-Tagatose 6-phosphate/D-Fructose 1-phosphate/alpha-D-Glucose 6-phosphate/1D-myo-Inositol 4-phosphate/alpha-D-Galactose 1-phosphate/D-Mannose 6-phosphate/sorbitol 3-phosphate
Carnitine shuttle	C18	1	5	0.45	(4R,8R,12R)-trimethyl-2E-tridecenoyl-CoA/palmitoleoyl-CoA/Hexadecenoyl-CoA (n-C16:1CoA)	0	5	0.37	
Chondroitin sulfate degradation	C18	4	4	0.00	L-Xylulose/D-Xylulose/L-Ribulose; L-erythro-Pentulose; L-Arabinoketose; L-Arabinulose; L-Riboketose/L-Arabinose/D-Xylose/D-Ribulose; D-erythro-2-Pentulose; D-Arabinoketose; D-Arabinulose; D-Riboketose/D-Ribose$D-Tagatose; lyxo-Hexulose/1,7-Dimethylxanthine; Paraxanthine/beta-D-Glucose/D-Fructose/alpha-D-Glucose//D-Glucose/myo-Inositol/D-Hexose/beta-D-Galactose/alpha-D-Galactose/L-Sorbose; L-xylo-Hexulose//Galactose/D-Mannose/beta-D-Fructose; beta-Fruit sugar; beta-D-arabino-Hexulose; beta-Levulose; Fructose/Glucose/D-Galactose$3-Dehydro-L-gulonate/L-Iduronic acid/D-Glucuronate$Sulfate	1	4	0.24	D-Tagatose; lyxo-Hexulose/1,7-Dimethylxanthine; Paraxanthine/beta-D-Glucose/D-Fructose/alpha-D-Glucose//D-Glucose/myo-Inositol/D-Hexose/beta-D-Galactose/alpha-D-Galactose/L-Sorbose; L-xylo-Hexulose//Galactose/D-Mannose/beta-D-Fructose; beta-Fruit sugar; beta-D-arabino-Hexulose; beta-Levulose; Fructose/Glucose/D-Galactose
CoA Catabolism	C18	0	1	0.50		0	1	0.37	
D4&E4-neuroprostanes formation	C18	2	4	0.18	D-glucurono-6,3-lactone/L-Ascorbate/D-glucurono-6,3-lactone$Dehydroascorbate/Dehydroascorbate/cis-Aconitate; cis-Aconitic acid	0	4	0.37	
De novo fatty acid biosynthesis	C18	2	14	0.49	$(4R,8R,12R)-trimethyl-2E-tridecenoyl-CoA/palmitoleoyl-CoA/Hexadecenoyl-CoA (n-C16:1CoA)	3	14	0.19	linoleic acid (all cis C18:2) n-6$$
Dimethyl-branched-chain fatty acid mitochondrial beta-oxidation	C18	0	0	0.50		0	0	0.37	
Di-unsaturated fatty acid beta-oxidation	C18	1	2	0.28	(3S)-3-hydroxy-cis,cis-palmito-7,10-dienoyl-CoA/3-oxopalmitoleoyl-CoA/trans-2-Enoyl-OPC6-CoA	1	2	0.14	linoleic acid (all cis C18:2) n-6
Drug metabolism - cytochrome P450	C18	2	8	0.41	2-Phenyl-1,3-propanediol monocarbamate$4-Glutathionyl cyclophosphamide	1	8	0.33	
Drug metabolism - other enzymes	C18	2	6	0.32	5'-Deoxy-5-fluorocytidine$Thiopurine/6-Mercaptopurin; Mercaptopurine	2	6	0.12	$Isoniazid
Dynorphin metabolism	C18	0	1	0.50		0	1	0.37	

Electron transport chain	C18	0	1	0.50		0	1	0.37	
Fatty acid activation	C18	2	11	0.48	(4R,8R,12R)-trimethyl-2E-tridecenoyl-CoA/palmitoleoyl-CoA/Hexadecenoyl-CoA (n-C16:1CoA)$	3	11	0.12	$$linoleic acid (all cis C18:2) n-6
Fatty Acid Metabolism	C18	0	6	0.50		2	6	0.12	$linoleic acid (all cis C18:2) n-6
Fatty acid oxidation	C18	1	2	0.28	(4R,8R,12R)-trimethyl-2E-tridecenoyl-CoA/palmitoleoyl-CoA/Hexadecenoyl-CoA (n-C16:1CoA)	0	2	0.37	
Fatty acid oxidation, peroxisome	C18	1	2	0.28	2-Oxoglutarate	0	2	0.37	
Fructose and mannose metabolism	C18	5	5	0.00	Dihydroxyacetone/D-Lactate/L-Lactate/3-Hydroxypropanoate; 3-Hydroxypropanoic acid; 3-Hydroxypropionate; 3-Hydroxypropionic acid; Hydracrylic acid/D-Glyceraldehyde$D-Hexose 6-phosphate/D-Glucose 1-phosphate/beta-D-Fructose 6-phosphate/D-Fructose 6-phosphate/Fructose 1-phosphate/alpha-D-Hexose 1-phosphate/beta-D-glucose 6-phosphate/1D-myo-Inositol 1-phosphate/alpha-D-Hexose 6-phosphate/D-Mannose 1-phosphate/D-Glucose 6-phosphate; Glucose 6-phosphate; Robison ester/Dolichyl phosphate D-mannose/1D-myo-Inositol 3-phosphate/D-Tagatose 6-phosphate/D-Fructose 1-phosphate/alpha-D-Glucose 6-phosphate/1D-myo-Inositol 4-phosphate/alpha-D-Galactose 1-phosphate/D-Mannose 6-phosphate/sorbitol 3-phosphate$6-Deoxy-L-galactose; L-Fucose$D-Sorbitol/Galactitol/L-Iditol$D-Tagatose; lyxo-Hexulose/1,7-Dimethylxanthine; Paraxanthine/beta-D-Glucose/D-Fructose/alpha-D-Glucose//D-Glucose/myo-Inositol/D-Hexose/beta-D-Galactose/alpha-D-Galactose/L-Sorbose; L-xylo-Hexulose//Galactose/D-Mannose/beta-D-Fructose; beta-Fruit sugar; beta-D-arabino-Hexulose; beta-Levulose; Fructose/Glucose/D-Galactose	5	5	0.00	6-Deoxy-L-galactose; L-Fucose$D-Tagatose; lyxo-Hexulose/1,7-Dimethylxanthine; Paraxanthine/beta-D-Glucose/D-Fructose/alpha-D-Glucose//D-Glucose/myo-Inositol/D-Hexose/beta-D-Galactose/alpha-D-Galactose/L-Sorbose; L-xylo-Hexulose//Galactose/D-Mannose/beta-D-Fructose; beta-Fruit sugar; beta-D-arabino-Hexulose; beta-Levulose; Fructose/Glucose/D-Galactose$D-Sorbitol/Galactitol/L-Iditol$D-Hexose 6-phosphate/D-Glucose 1-phosphate/beta-D-Fructose 6-phosphate/D-Fructose 6-phosphate/Fructose 1-phosphate/alpha-D-Hexose 1-phosphate/beta-D-glucose 6-phosphate/1D-myo-Inositol 1-phosphate/alpha-D-Hexose 6-phosphate/D-Mannose 1-phosphate/D-Glucose 6-phosphate; Glucose 6-phosphate; Robison ester/Dolichyl phosphate D-mannose/1D-myo-Inositol 3-phosphate/D-Tagatose 6-phosphate/D-Fructose 1-phosphate/alpha-D-Glucose 6-phosphate/1D-myo-Inositol 4-phosphate/alpha-D-Galactose 1-phosphate/D-Mannose 6-phosphate/sorbitol 3-phosphate$Dihydroxyaceto

									ne/D-Lactate/L-Lactate/3-Hydroxypropanoate; 3-Hydroxypropanoic acid; 3-Hydroxypropionate; 3-Hydroxypropionic acid; Hydracrylic acid/D-Glyceraldehyde

Galactose metabolism	C18	6	8	0.01	D-Hexose 6-phosphate/D-Glucose 1-phosphate/beta-D-Fructose 6-phosphate/D-Fructose 6-phosphate/Fructose 1-phosphate/alpha-D-Hexose 1-phosphate/beta-D-glucose 6-phosphate/1D-myo-Inositol 1-phosphate/alpha-D-Hexose 6-phosphate/D-Mannose 1-phosphate/D-Glucose 6-phosphate; Glucose 6-phosphate; Robison ester/Dolichyl phosphate D-mannose/1D-myo-Inositol 3-phosphate/D-Tagatose 6-phosphate/D-Fructose 1-phosphate/alpha-D-Glucose 6-phosphate/1D-myo-Inositol 4-phosphate/alpha-D-Galactose 1-phosphate/D-Mannose 6-phosphate/sorbitol 3-phosphate$D-Sorbitol/Galactitol/L-Iditol$3-Keto-beta-D-galactose/L-Gulono-1,4-lactone; L-Gulono-gamma-lactone; gamma-Gulonolactone; L-Gulonic acid gamma-lactone; L-Gulonolactone/D-Glucono-1,5-lactone; Gluconic lactone; Gluconic acid lactone; 1,5-Gluconolactone; delta-Gluconolactone; D-Gluconolactone; Gluconolactone$D-Tagatose; lyxo-Hexulose/1,7-Dimethylxanthine; Paraxanthine/beta-D-Glucose/D-Fructose/alpha-D-Glucose//D-Glucose/myo-Inositol/D-Hexose/beta-D-Galactose/alpha-D-Galactose/L-Sorbose; L-xylo-Hexulose//Galactose/D-Mannose/beta-D-Fructose; beta-Fruit sugar; beta-D-arabino-Hexulose; beta-Levulose; Fructose/Glucose/D-Galactose$3-beta-D-Galactosyl-sn-glycerol; Galactosylglycerol$Dihydroxyacetone/D-Lactate/L-Lactate/3-Hydroxypropanoate; 3-Hydroxypropanoic acid; 3-Hydroxypropionate; 3-Hydroxypropionic acid; Hydracrylic acid/D-Glyceraldehyde	5	8	0.00	D-Sorbitol/Galactitol/L-Iditol$D-Tagatose; lyxo-Hexulose/1,7-Dimethylxanthine; Paraxanthine/beta-D-Glucose/D-Fructose/alpha-D-Glucose//D-Glucose/myo-Inositol/D-Hexose/beta-D-Galactose/alpha-D-Galactose/L-Sorbose; L-xylo-Hexulose//Galactose/D-Mannose/beta-D-Fructose; beta-Fruit sugar; beta-D-arabino-Hexulose; beta-Levulose; Fructose/Glucose/D-Galactose$3-beta-D-Galactosyl-sn-glycerol; Galactosylglycerol$D-Hexose 6-phosphate/D-Glucose 1-phosphate/beta-D-Fructose 6-phosphate/D-Fructose 6-phosphate/Fructose 1-phosphate/alpha-D-Hexose 1-phosphate/beta-D-glucose 6-phosphate/1D-myo-Inositol 1-phosphate/alpha-D-Hexose 6-phosphate/D-Mannose 1-phosphate/D-Glucose 6-phosphate; Glucose 6-phosphate; Robison ester/Dolichyl phosphate D-mannose/1D-myo-Inositol 3-phosphate/D-Tagatose 6-phosphate/D-Fructose 1-phosphate/alpha-D-Glucose 6-phosphate/1D-myo-Inositol 4-phosphate/alpha-D-Galactose 1-phosphate/D-Mannose 6-phosphate/sorbitol 3-phosphate$Dihydroxyacetone/D-Lactate/L-Lactate/3-Hydroxypropanoate; 3-Hydroxypropanoic acid; 3-Hydroxypropionate; 3-Hydroxypropionic acid; Hydracrylic acid/D-Glyceraldehyde
Geraniol degradation	C18	0	0	0.50		0	0	0.37	

Glutamate metabolism	C18	5	5	0.00	D-Glutamate; D-Glutamic acid; D-Glutaminic acid; D-2-Aminoglutaric acid/L-4-Hydroxyglutamate semialdehyde/L-Glutamate$2-Oxoglutarate$3-Aminoisobutyric acid; 3-Aminoisobutanoate; 3-Amino-2-methylpropanoate/N,N-Dimethylglycine; Dimethylglycine/D-3-Amino-isobutanoate/(S)-2-Aminobutanoate; (S)-2-Aminobutanoic acid; (S)-2-Aminobutyric acid/4-Aminobutanoate/L-3-Amino-isobutanoate$Sarcosine; N-Methylglycine/D-Alanine/L-Alanine/beta-Alanine$D-Glutamine; D-2-Aminoglutaramic acid/L-Glutamine/3-Ureidoisobutyrate	1	5	0.28	D-Glutamine; D-2-Aminoglutaramic acid/L-Glutamine/3-Ureidoisobutyrate
Glutathione Metabolism	C18	2	3	0.11	D-Glutamate; D-Glutamic acid; D-Glutaminic acid; D-2-Aminoglutaric acid/L-4-Hydroxyglutamate semialdehyde/L-Glutamate$Sarcosine; N-Methylglycine/D-Alanine/L-Alanine/beta-Alanine	0	3	0.37	
Glycerolipid metabolism	C18	0	0	0.50		0	0	0.37	
Glycerophospholipid metabolism	C18	5	18	0.37	D-Hexose 6-phosphate/D-Glucose 1-phosphate/beta-D-Fructose 6-phosphate/D-Fructose 6-phosphate/Fructose 1-phosphate/alpha-D-Hexose 1-phosphate/beta-D-glucose 6-phosphate/1D-myo-Inositol 1-phosphate/alpha-D-Hexose 6-phosphate/D-Mannose 1-phosphate/D-Glucose 6-phosphate; Glucose 6-phosphate; Robison ester/Dolichyl phosphate D-mannose/1D-myo-Inositol 3-phosphate/D-Tagatose 6-phosphate/D-Fructose 1-phosphate/alpha-D-Glucose 6-phosphate/1D-myo-Inositol 4-phosphate/alpha-D-Galactose 1-phosphate/D-Mannose 6-phosphate/sorbitol 3-phosphate$L-Serine/D-Serine$D-Tagatose; lyxo-Hexulose/1,7-Dimethylxanthine; Paraxanthine/beta-D-Glucose/D-Fructose/alpha-D-Glucose//D-Glucose/myo-Inositol/D-Hexose/beta-D-Galactose/alpha-D-Galactose/L-Sorbose; L-xylo-Hexulose//Galactose/D-Mannose/beta-D-Fructose; beta-Fruit sugar; beta-D-arabino-Hexulose; beta-Levulose; Fructose/Glucose/D-Galactose$3-beta-D-Galactosyl-sn-glycerol; Galactosylglycerol$Dihydroxyacetone/D-Lactate/L-Lactate/3-Hydroxypropanoate; 3-Hydroxypropanoic acid; 3-Hydroxypropionate; 3-Hydroxypropionic acid; Hydracrylic acid/D-Glyceraldehyde	7	18	0.01	D-Hexose 6-phosphate/D-Glucose 1-phosphate/beta-D-Fructose 6-phosphate/D-Fructose 6-phosphate/Fructose 1-phosphate/alpha-D-Hexose 1-phosphate/beta-D-glucose 6-phosphate/1D-myo-Inositol 1-phosphate/alpha-D-Hexose 6-phosphate/D-Mannose 1-phosphate/D-Glucose 6-phosphate; Glucose 6-phosphate; Robison ester/Dolichyl phosphate D-mannose/1D-myo-Inositol 3-phosphate/D-Tagatose 6-phosphate/D-Fructose 1-phosphate/alpha-D-Glucose 6-phosphate/1D-myo-Inositol 4-phosphate/alpha-D-Galactose 1-phosphate/D-Mannose 6-phosphate/sorbitol 3-phosphate$linoleic acid (all cis C18:2) n-6$L-Serine/D-Serine$D-Tagatose; lyxo-Hexulose/1,7-Dimethylxanthine; Paraxanthine/beta-D-Glucose/D-Fructose/alpha-D-Glucose//D-Glucose/myo-Inositol/D-Hexose/beta-D-Galactose/alpha-D-Galactose/L-Sorbose; L-xylo-

									Hexulose//Galactose/D-Mannose/beta-D-Fructose; beta-Fruit sugar; beta-D-arabino-Hexulose; beta-Levulose; Fructose/Glucose/D-Galactose$3-beta-D-Galactosyl-sn-glycerol; Galactosylglycerol$Ethanolamine phosphate$Dihydroxyacetone/D-Lactate/L-Lactate/3-Hydroxypropanoate; 3-Hydroxypropanoic acid; 3-Hydroxypropionate; 3-Hydroxypropionic acid; Hydracrylic acid/D-Glyceraldehyde

Glycine, serine, alanine and threonine metabolism	C18	10	15	0.00	D-Glutamate; D-Glutamic acid; D-Glutaminic acid; D-2-Aminoglutaric acid/L-4-Hydroxyglutamate semialdehyde/L-Glutamate$2-Oxoglutarate$3-Aminoisobutyric acid; 3-Aminoisobutanoate; 3-Amino-2-methylpropanoate/N,N-Dimethylglycine; Dimethylglycine/D-3-Amino-isobutanoate/(S)-2-Aminobutanoate; (S)-2-Aminobutanoic acid; (S)-2-Aminobutyric acid/4-Aminobutanoate/L-3-Amino-isobutanoate$L-Allo-threonine/L-Threonine$L-Serine/D-Serine$Sarcosine; N-Methylglycine/D-Alanine/L-Alanine/beta-Alanine$5-Aminolevulinate; 5-Amino-4-oxopentanoate; 5-Amino-4-oxovaleric acid/cis-4-Hydroxy-D-proline/2-Amino-4-oxopentanoic acid; 2-Amino-4-oxopentanoate/L-Glutamate 5-semialdehyde; L-Glutamate gamma-semialdehyde/trans-4-Hydroxy-L-proline/5-Amino-2-oxopentanoic acid; 5-Amino-2-oxopentanoate; 2-Oxo-5-amino-pentanoate; 2-Oxo-5-aminopentanoate; alpha-Keto-delta-aminopentanoate; 2-Oxo-5-aminovalerate$Dihydrolipoamide; Dihydrothioctamide$Dihydroxyacetone/D-Lactate/L-Lactate/3-Hydroxypropanoate; 3-Hydroxypropanoic acid; 3-Hydroxypropionate; 3-Hydroxypropionic acid; Hydracrylic acid/D-Glyceraldehyde$D-Arginine; D-2-Amino-5-guanidinovaleric acid/L-Arginine	3	15	0.21	Dihydroxyacetone/D-Lactate/L-Lactate/3-Hydroxypropanoate; 3-Hydroxypropanoic acid; 3-Hydroxypropionate; 3-Hydroxypropionic acid; Hydracrylic acid/D-Glyceraldehyde$L-Serine/D-Serine$

Glycolysis and Gluconeogenesis	C18	6	7	0.00	D-Hexose 6-phosphate/D-Glucose 1-phosphate/beta-D-Fructose 6-phosphate/D-Fructose 6-phosphate/Fructose 1-phosphate/alpha-D-Hexose 1-phosphate/beta-D-glucose 6-phosphate/1D-myo-Inositol 1-phosphate/alpha-D-Hexose 6-phosphate/D-Mannose 1-phosphate/D-Glucose 6-phosphate; Glucose 6-phosphate; Robison ester/Dolichyl phosphate D-mannose/1D-myo-Inositol 3-phosphate/D-Tagatose 6-phosphate/D-Fructose 1-phosphate/alpha-D-Glucose 6-phosphate/1D-myo-Inositol 4-phosphate/alpha-D-Galactose 1-phosphate/D-Mannose 6-phosphate/sorbitol 3-phosphate$L-Malate$D-Tagatose; lyxo-Hexulose/1,7-Dimethylxanthine; Paraxanthine/beta-D-Glucose/D-Fructose/alpha-D-Glucose//D-Glucose/myo-Inositol/D-Hexose/beta-D-Galactose/alpha-D-Galactose/L-Sorbose; L-xylo-Hexulose//Galactose/D-Mannose/beta-D-Fructose; beta-Fruit sugar; beta-D-arabino-Hexulose; beta-Levulose; Fructose/Glucose/D-Galactose$2-Acetolactate/(S)-2-Acetolactate; (S)-2-Hydroxy-2-methyl-3-oxobutanoate$Dihydroxyacetone/D-Lactate/L-Lactate/3-Hydroxypropanoate; 3-Hydroxypropanoic acid; 3-Hydroxypropionate; 3-Hydroxypropionic acid; Hydracrylic acid/D-Glyceraldehyde$Dihydrolipoamide; Dihydrothioctamide	4	7	0.01	D-Tagatose; lyxo-Hexulose/1,7-Dimethylxanthine; Paraxanthine/beta-D-Glucose/D-Fructose/alpha-D-Glucose//D-Glucose/myo-Inositol/D-Hexose/beta-D-Galactose/alpha-D-Galactose/L-Sorbose; L-xylo-Hexulose//Galactose/D-Mannose/beta-D-Fructose; beta-Fruit sugar; beta-D-arabino-Hexulose; beta-Levulose; Fructose/Glucose/D-Galactose$D-Hexose 6-phosphate/D-Glucose 1-phosphate/beta-D-Fructose 6-phosphate/D-Fructose 6-phosphate/Fructose 1-phosphate/alpha-D-Hexose 1-phosphate/beta-D-glucose 6-phosphate/1D-myo-Inositol 1-phosphate/alpha-D-Hexose 6-phosphate/D-Mannose 1-phosphate/D-Glucose 6-phosphate; Glucose 6-phosphate; Robison ester/Dolichyl phosphate D-mannose/1D-myo-Inositol 3-phosphate/D-Tagatose 6-phosphate/D-Fructose 1-phosphate/alpha-D-Glucose 6-phosphate/1D-myo-Inositol 4-phosphate/alpha-D-Galactose 1-phosphate/D-Mannose 6-phosphate/sorbitol 3-phosphate$Dihydroxyacetone/D-Lactate/L-Lactate/3-Hydroxypropanoate; 3-Hydroxypropanoic acid; 3-Hydroxypropionate; 3-Hydroxypropionic acid; Hydracrylic acid/D-Glyceraldehyde$2-Acetolactate/(S)-2-Acetolactate; (S)-2-Hydroxy-2-methyl-3-oxobutanoate
Glycosaminoglycan degradation	C18	0	0	0.50		0	0	0.37	

Glycosphingolipid biosynthesis - ganglioseries	C18	1	3	0.36	D-Tagatose; lyxo-Hexulose/1,7-Dimethylxanthine; Paraxanthine/beta-D-Glucose/D-Fructose/alpha-D-Glucose//D-Glucose/myo-Inositol/D-Hexose/beta-D-Galactose/alpha-D-Galactose/L-Sorbose; L-xylo-Hexulose//Galactose/D-Mannose/beta-D-Fructose; beta-Fruit sugar; beta-D-arabino-Hexulose; beta-Levulose; Fructose/Glucose/D-Galactose	1	3	0.20	D-Tagatose; lyxo-Hexulose/1,7-Dimethylxanthine; Paraxanthine/beta-D-Glucose/D-Fructose/alpha-D-Glucose//D-Glucose/myo-Inositol/D-Hexose/beta-D-Galactose/alpha-D-Galactose/L-Sorbose; L-xylo-Hexulose//Galactose/D-Mannose/beta-D-Fructose; beta-Fruit sugar; beta-D-arabino-Hexulose; beta-Levulose; Fructose/Glucose/D-Galactose
Glycosphingolipid biosynthesis - globoseries	C18	1	2	0.28	D-Tagatose; lyxo-Hexulose/1,7-Dimethylxanthine; Paraxanthine/beta-D-Glucose/D-Fructose/alpha-D-Glucose//D-Glucose/myo-Inositol/D-Hexose/beta-D-Galactose/alpha-D-Galactose/L-Sorbose; L-xylo-Hexulose//Galactose/D-Mannose/beta-D-Fructose; beta-Fruit sugar; beta-D-arabino-Hexulose; beta-Levulose; Fructose/Glucose/D-Galactose	1	2	0.14	D-Tagatose; lyxo-Hexulose/1,7-Dimethylxanthine; Paraxanthine/beta-D-Glucose/D-Fructose/alpha-D-Glucose//D-Glucose/myo-Inositol/D-Hexose/beta-D-Galactose/alpha-D-Galactose/L-Sorbose; L-xylo-Hexulose//Galactose/D-Mannose/beta-D-Fructose; beta-Fruit sugar; beta-D-arabino-Hexulose; beta-Levulose; Fructose/Glucose/D-Galactose
Glycosphingolipid biosynthesis - lactoseries	C18	0	1	0.50		0	1	0.37	
Glycosphingolipid biosynthesis - neolactoseries	C18	0	1	0.50		0	1	0.37	
Glycosphingolipid metabolism	C18	3	9	0.29	L-Serine/D-Serine$Sulfate$D-Tagatose; lyxo-Hexulose/1,7-Dimethylxanthine; Paraxanthine/beta-D-Glucose/D-Fructose/alpha-D-Glucose//D-Glucose/myo-Inositol/D-Hexose/beta-D-Galactose/alpha-D-Galactose/L-Sorbose; L-xylo-Hexulose//Galactose/D-Mannose/beta-D-Fructose; beta-Fruit sugar; beta-D-arabino-Hexulose; beta-Levulose; Fructose/Glucose/D-Galactose	4	9	0.02	D-Tagatose; lyxo-Hexulose/1,7-Dimethylxanthine; Paraxanthine/beta-D-Glucose/D-Fructose/alpha-D-Glucose//D-Glucose/myo-Inositol/D-Hexose/beta-D-Galactose/alpha-D-Galactose/L-Sorbose; L-xylo-Hexulose//Galactose/D-Mannose/beta-D-Fructose; beta-Fruit sugar; beta-D-arabino-Hexulose; beta-Levulose; Fructose/Glucose/D-Galactose$L-Serine/D-Serine$N-Acetylneuraminate; N-Acetylneuraminic acid; 5-

									Acetamido-3,5-dideoxy-D-glycero-D-galacto-2-nonulosonic acid; Neu5Ac$Ethanolamine phosphate
Glycosylphosphatidylinositol(GPI)-anchor biosynthesis	C18	1	1	0.15	D-Hexose 6-phosphate/D-Glucose 1-phosphate/beta-D-Fructose 6-phosphate/D-Fructose 6-phosphate/Fructose 1-phosphate/alpha-D-Hexose 1-phosphate/beta-D-glucose 6-phosphate/1D-myo-Inositol 1-phosphate/alpha-D-Hexose 6-phosphate/D-Mannose 1-phosphate/D-Glucose 6-phosphate; Glucose 6-phosphate; Robison ester/Dolichyl phosphate D-mannose/1D-myo-Inositol 3-phosphate/D-Tagatose 6-phosphate/D-Fructose 1-phosphate/alpha-D-Glucose 6-phosphate/1D-myo-Inositol 4-phosphate/alpha-D-Galactose 1-phosphate/D-Mannose 6-phosphate/sorbitol 3-phosphate	1	1	0.08	D-Hexose 6-phosphate/D-Glucose 1-phosphate/beta-D-Fructose 6-phosphate/D-Fructose 6-phosphate/Fructose 1-phosphate/alpha-D-Hexose 1-phosphate/beta-D-glucose 6-phosphate/1D-myo-Inositol 1-phosphate/alpha-D-Hexose 6-phosphate/D-Mannose 1-phosphate/D-Glucose 6-phosphate; Glucose 6-phosphate; Robison ester/Dolichyl phosphate D-mannose/1D-myo-Inositol 3-phosphate/D-Tagatose 6-phosphate/D-Fructose 1-phosphate/alpha-D-Glucose 6-phosphate/1D-myo-Inositol 4-phosphate/alpha-D-Galactose 1-phosphate/D-Mannose 6-phosphate/sorbitol 3-phosphate
Glyoxylate and Dicarboxylate Metabolism	C18	1	2	0.28	L-Xylulose/D-Xylulose/L-Ribulose; L-erythro-Pentulose; L-Arabinoketose; L-Arabinulose; L-Riboketose/L-Arabinose/D-Xylose/D-Ribulose; D-erythro-2-Pentulose; D-Arabinoketose; D-Arabinulose; D-Riboketose/D-Ribose	0	2	0.37	
Heparan sulfate biosynthesis	C18	0	0	0.50		0	0	0.37	

Heparan sulfate degradation	C18	4	4	0.00	L-Xylulose/D-Xylulose/L-Ribulose; L-erythro-Pentulose; L-Arabinoketose; L-Arabinulose; L-Riboketose/L-Arabinose/D-Xylose/D-Ribulose; D-erythro-2-Pentulose; D-Arabinoketose; D-Arabinulose; D-Riboketose/D-Ribose$D-Tagatose; lyxo-Hexulose/1,7-Dimethylxanthine; Paraxanthine/beta-D-Glucose/D-Fructose/alpha-D-Glucose//D-Glucose/myo-Inositol/D-Hexose/beta-D-Galactose/alpha-D-Galactose/L-Sorbose; L-xylo-Hexulose//Galactose/D-Mannose/beta-D-Fructose; beta-Fruit sugar; beta-D-arabino-Hexulose; beta-Levulose; Fructose/Glucose/D-Galactose$3-Dehydro-L-gulonate/L-Iduronic acid/D-Glucuronate$Sulfate	1	4	0.24	D-Tagatose; lyxo-Hexulose/1,7-Dimethylxanthine; Paraxanthine/beta-D-Glucose/D-Fructose/alpha-D-Glucose//D-Glucose/myo-Inositol/D-Hexose/beta-D-Galactose/alpha-D-Galactose/L-Sorbose; L-xylo-Hexulose//Galactose/D-Mannose/beta-D-Fructose; beta-Fruit sugar; beta-D-arabino-Hexulose; beta-Levulose; Fructose/Glucose/D-Galactose
Hexose phosphorylation	C18	4	5	0.01	D-Glucosamine$D-Hexose 6-phosphate/D-Glucose 1-phosphate/beta-D-Fructose 6-phosphate/D-Fructose 6-phosphate/Fructose 1-phosphate/alpha-D-Hexose 1-phosphate/beta-D-glucose 6-phosphate/1D-myo-Inositol 1-phosphate/alpha-D-Hexose 6-phosphate/D-Mannose 1-phosphate/D-Glucose 6-phosphate; Glucose 6-phosphate; Robison ester/Dolichyl phosphate D-mannose/1D-myo-Inositol 3-phosphate/D-Tagatose 6-phosphate/D-Fructose 1-phosphate/alpha-D-Glucose 6-phosphate/1D-myo-Inositol 4-phosphate/alpha-D-Galactose 1-phosphate/D-Mannose 6-phosphate/sorbitol 3-phosphate$D-Tagatose; lyxo-Hexulose/1,7-Dimethylxanthine; Paraxanthine/beta-D-Glucose/D-Fructose/alpha-D-Glucose//D-Glucose/myo-Inositol/D-Hexose/beta-D-Galactose/alpha-D-Galactose/L-Sorbose; L-xylo-Hexulose//Galactose/D-Mannose/beta-D-Fructose; beta-Fruit sugar; beta-D-arabino-Hexulose; beta-Levulose; Fructose/Glucose/D-Galactose$D-Sorbitol/Galactitol/L-Iditol	3	5	0.01	D-Tagatose; lyxo-Hexulose/1,7-Dimethylxanthine; Paraxanthine/beta-D-Glucose/D-Fructose/alpha-D-Glucose//D-Glucose/myo-Inositol/D-Hexose/beta-D-Galactose/alpha-D-Galactose/L-Sorbose; L-xylo-Hexulose//Galactose/D-Mannose/beta-D-Fructose; beta-Fruit sugar; beta-D-arabino-Hexulose; beta-Levulose; Fructose/Glucose/D-Galactose$D-Sorbitol/Galactitol/L-Iditol$D-Hexose 6-phosphate/D-Glucose 1-phosphate/beta-D-Fructose 6-phosphate/D-Fructose 6-phosphate/Fructose 1-phosphate/alpha-D-Hexose 1-phosphate/beta-D-glucose 6-phosphate/1D-myo-Inositol 1-phosphate/alpha-D-Hexose 6-phosphate/D-Mannose 1-phosphate/D-Glucose 6-phosphate; Glucose 6-phosphate; Robison ester/Dolichyl phosphate D-mannose/1D-myo-Inositol 3-phosphate/D-Tagatose 6-phosphate/D-Fructose 1-phosphate/alpha-D-Glucose 6-phosphate/1D-myo-Inositol 4-phosphate/alpha-

									D-Galactose 1-phosphate/D-Mannose 6-phosphate/sorbitol 3-phosphate
Histidine metabolism	C18	3	4	0.04	D-Glutamate; D-Glutamic acid; D-Glutaminic acid; D-2-Aminoglutaric acid/L-4-Hydroxyglutamate semialdehyde/L-Glutamate$2-Oxoglutarate$Sarcosine; N-Methylglycine/D-Alanine/L-Alanine/beta-Alanine	1	4	0.24	
Hyaluronan Metabolism	C18	1	2	0.28	3-Dehydro-L-gulonate/L-Iduronic acid/D-Glucuronate	0	2	0.37	
Keratan sulfate biosynthesis	C18	0	1	0.50		0	1	0.37	
Keratan sulfate degradation	C18	3	5	0.07	D-Tagatose; lyxo-Hexulose/1,7-Dimethylxanthine; Paraxanthine/beta-D-Glucose/D-Fructose/alpha-D-Glucose//D-Glucose/myo-Inositol/D-Hexose/beta-D-Galactose/alpha-D-Galactose/L-Sorbose; L-xylo-Hexulose//Galactose/D-Mannose/beta-D-Fructose; beta-Fruit sugar; beta-D-arabino-Hexulose; beta-Levulose; Fructose/Glucose/D-Galactose$6-Deoxy-L-galactose; L-Fucose$Sulfate	3	5	0.01	D-Tagatose; lyxo-Hexulose/1,7-Dimethylxanthine; Paraxanthine/beta-D-Glucose/D-Fructose/alpha-D-Glucose//D-Glucose/myo-Inositol/D-Hexose/beta-D-Galactose/alpha-D-Galactose/L-Sorbose; L-xylo-Hexulose//Galactose/D-Mannose/beta-D-Fructose; beta-Fruit sugar; beta-D-arabino-Hexulose; beta-Levulose; Fructose/Glucose/D-Galactose$N-Acetylneuraminate; N-Acetylneuraminic acid; 5-Acetamido-3,5-dideoxy-D-glycero-D-galacto-2-

									nonulosonic acid; Neu5Ac$6-Deoxy-L-galactose; L-Fucose
Leukotriene metabolism	C18	1	11	0.50	DL-Glutamate; DL-Glutaminic acid; 2-Aminoglutaric acid; Glutamate	1	11	0.36	
Limonene and pinene degradation	C18	0	0	0.50		0	0	0.37	
Linoleate metabolism	C18	1	12	0.50	D-glucurono-6,3-lactone/L-Ascorbate/D-glucurono-6,3-lactone	1	12	0.36	linoleic acid (all cis C18:2) n-6
Lipoate metabolism	C18	1	1	0.15	Dihydrolipoamide; Dihydrothioctamide	0	1	0.37	
Lysine metabolism	C18	5	6	0.01	D-Glutamate; D-Glutamic acid; D-Glutaminic acid; D-2-Aminoglutaric acid/L-4-Hydroxyglutamate semialdehyde/L-Glutamate$2-Oxoglutarate$Dihydrolipoamide; Dihydrothioctamide$N4-Acetylaminobutanal/L-Pipecolate; Pipecolinic acid; Pipecolic acid; 2-Piperidinecarboxylic acid$L-2-Aminoadipate; L-alpha-Aminoadipate; L-alpha-Aminoadipic acid; L-2-Aminoadipic acid; L-2-Aminohexanedioate	1	6	0.30	
Methionine and cysteine metabolism	C18	6	9	0.01	D-Glutamate; D-Glutamic acid; D-Glutaminic acid; D-2-Aminoglutaric acid/L-4-Hydroxyglutamate semialdehyde/L-Glutamate$2-Oxoglutarate$DL-Glutamate; DL-Glutaminic acid; 2-Aminoglutaric acid; Glutamate$L-Serine/D-Serine$L-Cystine; L-Dicysteine; L-alpha-Diamino-beta-dithiolactic acid$Sulfate	1	9	0.35	L-Serine/D-Serine
Mono-unsaturated fatty acid beta-oxidation	C18	2	2	0.04	(4R,8R,12R)-trimethyl-2E-tridecenoyl-CoA/palmitoleoyl-CoA/Hexadecenoyl-CoA (n-C16:1CoA)$(3S)-3-hydroxy-cis,cis-palmito-7,10-dienoyl-CoA/3-oxopalmitoleoyl-CoA/trans-2-Enoyl-OPC6-CoA	0	2	0.37	

N-Glycan biosynthesis	C18	2	3	0.11	D-Hexose 6-phosphate/D-Glucose 1-phosphate/beta-D-Fructose 6-phosphate/D-Fructose 6-phosphate/Fructose 1-phosphate/alpha-D-Hexose 1-phosphate/beta-D-glucose 6-phosphate/1D-myo-Inositol 1-phosphate/alpha-D-Hexose 6-phosphate/D-Mannose 1-phosphate/D-Glucose 6-phosphate; Glucose 6-phosphate; Robison ester/Dolichyl phosphate D-mannose/1D-myo-Inositol 3-phosphate/D-Tagatose 6-phosphate/D-Fructose 1-phosphate/alpha-D-Glucose 6-phosphate/1D-myo-Inositol 4-phosphate/alpha-D-Galactose 1-phosphate/D-Mannose 6-phosphate/sorbitol 3-phosphate$D-Tagatose; lyxo-Hexulose/1,7-Dimethylxanthine; Paraxanthine/beta-D-Glucose/D-Fructose/alpha-D-Glucose//D-Glucose/myo-Inositol/D-Hexose/beta-D-Galactose/alpha-D-Galactose/L-Sorbose; L-xylo-Hexulose//Galactose/D-Mannose/beta-D-Fructose; beta-Fruit sugar; beta-D-arabino-Hexulose; beta-Levulose; Fructose/Glucose/D-Galactose	2	3	0.03	D-Tagatose; lyxo-Hexulose/1,7-Dimethylxanthine; Paraxanthine/beta-D-Glucose/D-Fructose/alpha-D-Glucose//D-Glucose/myo-Inositol/D-Hexose/beta-D-Galactose/alpha-D-Galactose/L-Sorbose; L-xylo-Hexulose//Galactose/D-Mannose/beta-D-Fructose; beta-Fruit sugar; beta-D-arabino-Hexulose; beta-Levulose; Fructose/Glucose/D-Galactose$D-Hexose 6-phosphate/D-Glucose 1-phosphate/beta-D-Fructose 6-phosphate/D-Fructose 6-phosphate/Fructose 1-phosphate/alpha-D-Hexose 1-phosphate/beta-D-glucose 6-phosphate/1D-myo-Inositol 1-phosphate/alpha-D-Hexose 6-phosphate/D-Mannose 1-phosphate/D-Glucose 6-phosphate; Glucose 6-phosphate; Robison ester/Dolichyl phosphate D-mannose/1D-myo-Inositol 3-phosphate/D-Tagatose 6-phosphate/D-Fructose 1-phosphate/alpha-D-Glucose 6-phosphate/1D-myo-Inositol 4-phosphate/alpha-D-Galactose 1-phosphate/D-Mannose 6-phosphate/sorbitol 3-phosphate
N-Glycan Degradation	C18	2	3	0.11	D-Tagatose; lyxo-Hexulose/1,7-Dimethylxanthine; Paraxanthine/beta-D-Glucose/D-Fructose/alpha-D-Glucose//D-Glucose/myo-Inositol/D-Hexose/beta-D-Galactose/alpha-D-Galactose/L-Sorbose; L-xylo-Hexulose//Galactose/D-Mannose/beta-D-Fructose; beta-Fruit sugar; beta-D-arabino-Hexulose; beta-Levulose; Fructose/Glucose/D-Galactose$6-Deoxy-L-galactose; L-Fucose	3	3	0.00	D-Tagatose; lyxo-Hexulose/1,7-Dimethylxanthine; Paraxanthine/beta-D-Glucose/D-Fructose/alpha-D-Glucose//D-Glucose/myo-Inositol/D-Hexose/beta-D-Galactose/alpha-D-Galactose/L-Sorbose; L-xylo-Hexulose//Galactose/D-Mannose/beta-D-Fructose; beta-Fruit sugar; beta-D-arabino-Hexulose; beta-Levulose;

									Fructose/Glucose/D-Galactose$N-Acetylneuraminate; N-Acetylneuraminic acid; 5-Acetamido-3,5-dideoxy-D-glycero-D-galacto-2-nonulosonic acid; Neu5Ac$6-Deoxy-L-galactose; L-Fucose
Nitrogen metabolism	C18	2	3	0.11	D-Glutamate; D-Glutamic acid; D-Glutaminic acid; D-2-Aminoglutaric acid/L-4-Hydroxyglutamate semialdehyde/L-Glutamate$D-Glutamine; D-2-Aminoglutaramic acid/L-Glutamine/3-Ureidoisobutyrate	1	3	0.20	D-Glutamine; D-2-Aminoglutaramic acid/L-Glutamine/3-Ureidoisobutyrate
Nucleotide Sugar Metabolism	C18	1	1	0.15	D-Hexose 6-phosphate/D-Glucose 1-phosphate/beta-D-Fructose 6-phosphate/D-Fructose 6-phosphate/Fructose 1-phosphate/alpha-D-Hexose 1-phosphate/beta-D-glucose 6-phosphate/1D-myo-Inositol 1-phosphate/alpha-D-Hexose 6-phosphate/D-Mannose 1-phosphate/D-Glucose 6-phosphate; Glucose 6-phosphate; Robison ester/Dolichyl phosphate D-mannose/1D-myo-Inositol 3-phosphate/D-Tagatose 6-phosphate/D-Fructose 1-phosphate/alpha-D-Glucose 6-phosphate/1D-myo-Inositol 4-phosphate/alpha-D-Galactose 1-phosphate/D-Mannose 6-phosphate/sorbitol 3-phosphate	1	1	0.08	D-Hexose 6-phosphate/D-Glucose 1-phosphate/beta-D-Fructose 6-phosphate/D-Fructose 6-phosphate/Fructose 1-phosphate/alpha-D-Hexose 1-phosphate/beta-D-glucose 6-phosphate/1D-myo-Inositol 1-phosphate/alpha-D-Hexose 6-phosphate/D-Mannose 1-phosphate/D-Glucose 6-phosphate; Glucose 6-phosphate; Robison ester/Dolichyl phosphate D-mannose/1D-myo-Inositol 3-phosphate/D-Tagatose 6-phosphate/D-Fructose 1-phosphate/alpha-D-Glucose 6-phosphate/1D-myo-Inositol 4-phosphate/alpha-D-Galactose 1-phosphate/D-Mannose 6-phosphate/sorbitol 3-phosphate
O-Glycan biosynthesis	C18	0	1	0.50		0	1	0.37	
Omega-3 fatty acid metabolism	C18	1	4	0.42	(3S)-3-hydroxy-cis,cis-palmito-7,10-dienoyl-CoA/3-oxopalmitoleoyl-CoA/trans-2-Enoyl-OPC6-CoA	0	4	0.37	
Omega-6 fatty acid metabolism	C18	0	2	0.50		0	2	0.37	
Parathio degradation	C18	0	0	0.50		0	0	0.37	

Pentose and Glucuronate Interconversions	C18	4	5	0.01	L-Xylulose/D-Xylulose/L-Ribulose; L-erythro-Pentulose; L-Arabinoketose; L-Arabinulose; L-Riboketose/L-Arabinose/D-Xylose/D-Ribulose; D-erythro-2-Pentulose; D-Arabinoketose; D-Arabinulose; D-Riboketose/D-Ribose$L-Ribulose 5-phosphate/alpha-D-Ribose 5-phosphate/alpha-D-Ribose 5-phosphate/alpha-D-Ribose 1-phosphate/alpha-D-Ribose 1-phosphate/D-Xylulose 5-phosphate/D-Ribulose 5-phosphate$3-Dehydro-L-gulonate/L-Iduronic acid/D-Glucuronate$D-Gluconate/L-Gulonate	0	5	0.37	
Pentose phosphate pathway	C18	7	10	0.00	D-Hexose 6-phosphate/D-Glucose 1-phosphate/beta-D-Fructose 6-phosphate/D-Fructose 6-phosphate/Fructose 1-phosphate/alpha-D-Hexose 1-phosphate/beta-D-glucose 6-phosphate/1D-myo-Inositol 1-phosphate/alpha-D-Hexose 6-phosphate/D-Mannose 1-phosphate/D-Glucose 6-phosphate; Glucose 6-phosphate; Robison ester/Dolichyl phosphate D-mannose/1D-myo-Inositol 3-phosphate/D-Tagatose 6-phosphate/D-Fructose 1-phosphate/alpha-D-Glucose 6-phosphate/1D-myo-Inositol 4-phosphate/alpha-D-Galactose 1-phosphate/D-Mannose 6-phosphate/sorbitol 3-phosphate$L-Ribulose 5-phosphate/alpha-D-Ribose 5-phosphate/alpha-D-Ribose 5-phosphate/alpha-D-Ribose 1-phosphate/alpha-D-Ribose 1-phosphate/D-Xylulose 5-phosphate/D-Ribulose 5-phosphate$L-Xylulose/D-Xylulose/L-Ribulose; L-erythro-Pentulose; L-Arabinoketose; L-Arabinulose; L-Riboketose/L-Arabinose/D-Xylose/D-Ribulose; D-erythro-2-Pentulose; D-Arabinoketose; D-Arabinulose; D-Riboketose/D-Ribose$D-Tagatose; lyxo-Hexulose/1,7-Dimethylxanthine; Paraxanthine/beta-D-Glucose/D-Fructose/alpha-D-Glucose//D-Glucose/myo-Inositol/D-Hexose/beta-D-Galactose/alpha-D-Galactose/L-Sorbose; L-xylo-Hexulose//Galactose/D-Mannose/beta-D-Fructose; beta-Fruit sugar; beta-D-arabino-Hexulose; beta-Levulose; Fructose/Glucose/D-Galactose$3-Keto-beta-D-galactose/L-Gulono-1,4-lactone; L-Gulono-gamma-lactone; gamma-Gulonolactone; L-Gulonic acid gamma-lactone; L-Gulonolactone/D-Glucono-1,5-lactone; Gluconic lactone; Gluconic acid lactone; 1,5-Gluconolactone; delta-Gluconolactone; D-Gluconolactone; Gluconolactone$1,3,7-Trimethyluric acid/sedoheptulose$D-Gluconate/L-Gulonate	3	10	0.10	D-Tagatose; lyxo-Hexulose/1,7-Dimethylxanthine; Paraxanthine/beta-D-Glucose/D-Fructose/alpha-D-Glucose//D-Glucose/myo-Inositol/D-Hexose/beta-D-Galactose/alpha-D-Galactose/L-Sorbose; L-xylo-Hexulose//Galactose/D-Mannose/beta-D-Fructose; beta-Fruit sugar; beta-D-arabino-Hexulose; beta-Levulose; Fructose/Glucose/D-Galactose$1,3,7-Trimethyluric acid/sedoheptulose$D-Hexose 6-phosphate/D-Glucose 1-phosphate/beta-D-Fructose 6-phosphate/D-Fructose 6-phosphate/Fructose 1-phosphate/alpha-D-Hexose 1-phosphate/beta-D-glucose 6-phosphate/1D-myo-Inositol 1-phosphate/alpha-D-Hexose 6-phosphate/D-Mannose 1-phosphate/D-Glucose 6-phosphate; Glucose 6-phosphate; Robison ester/Dolichyl phosphate D-mannose/1D-myo-Inositol 3-phosphate/D-Tagatose 6-phosphate/D-Fructose 1-phosphate/alpha-D-Glucose 6-phosphate/1D-myo-Inositol 4-phosphate/alpha-D-Galactose 1-phosphate/D-Mannose 6-phosphate/sorbitol 3-phosphate

Phosphatidylinositol phosphate metabolism	C18	3	8	0.24	D-Hexose 6-phosphate/D-Glucose 1-phosphate/beta-D-Fructose 6-phosphate/D-Fructose 6-phosphate/Fructose 1-phosphate/alpha-D-Hexose 1-phosphate/beta-D-glucose 6-phosphate/1D-myo-Inositol 1-phosphate/alpha-D-Hexose 6-phosphate/D-Mannose 1-phosphate/D-Glucose 6-phosphate; Glucose 6-phosphate; Robison ester/Dolichyl phosphate D-mannose/1D-myo-Inositol 3-phosphate/D-Tagatose 6-phosphate/D-Fructose 1-phosphate/alpha-D-Glucose 6-phosphate/1D-myo-Inositol 4-phosphate/alpha-D-Galactose 1-phosphate/D-Mannose 6-phosphate/sorbitol 3-phosphate$D-Tagatose; lyxo-Hexulose/1,7-Dimethylxanthine; Paraxanthine/beta-D-Glucose/D-Fructose/alpha-D-Glucose//D-Glucose/myo-Inositol/D-Hexose/beta-D-Galactose/alpha-D-Galactose/L-Sorbose; L-xylo-Hexulose//Galactose/D-Mannose/beta-D-Fructose; beta-Fruit sugar; beta-D-arabino-Hexulose; beta-Levulose; Fructose/Glucose/D-Galactose$3-Dehydro-L-gulonate/L-Iduronic acid/D-Glucuronate	2	8	0.19	D-Tagatose; lyxo-Hexulose/1,7-Dimethylxanthine; Paraxanthine/beta-D-Glucose/D-Fructose/alpha-D-Glucose//D-Glucose/myo-Inositol/D-Hexose/beta-D-Galactose/alpha-D-Galactose/L-Sorbose; L-xylo-Hexulose//Galactose/D-Mannose/beta-D-Fructose; beta-Fruit sugar; beta-D-arabino-Hexulose; beta-Levulose; Fructose/Glucose/D-Galactose$D-Hexose 6-phosphate/D-Glucose 1-phosphate/beta-D-Fructose 6-phosphate/D-Fructose 6-phosphate/Fructose 1-phosphate/alpha-D-Hexose 1-phosphate/beta-D-glucose 6-phosphate/1D-myo-Inositol 1-phosphate/alpha-D-Hexose 6-phosphate/D-Mannose 1-phosphate/D-Glucose 6-phosphate; Glucose 6-phosphate; Robison ester/Dolichyl phosphate D-mannose/1D-myo-Inositol 3-phosphate/D-Tagatose 6-phosphate/D-Fructose 1-phosphate/alpha-D-Glucose 6-phosphate/1D-myo-Inositol 4-phosphate/alpha-D-Galactose 1-phosphate/D-Mannose 6-phosphate/sorbitol 3-phosphate
Phytanic acid peroxisomal oxidation	C18	2	3	0.11	2-Oxoglutarate$(4R,8R,12R)-trimethyl-2E-tridecenoyl-CoA/palmitoleoyl-CoA/Hexadecenoyl-CoA (n-C16:1CoA)	0	3	0.37	
Polyunsaturated fatty acid biosynthesis	C18	0	2	0.50		1	2	0.14	

Porphyrin metabolism	C18	5	7	0.01	D-glucurono-6,3-lactone/L-Ascorbate/D-glucurono-6,3-lactone$5-Aminolevulinate; 5-Amino-4-oxopentanoate; 5-Amino-4-oxovaleric acid/cis-4-Hydroxy-D-proline/2-Amino-4-oxopentanoic acid; 2-Amino-4-oxopentanoate/L-Glutamate 5-semialdehyde; L-Glutamate gamma-semialdehyde/trans-4-Hydroxy-L-proline/5-Amino-2-oxopentanoic acid; 5-Amino-2-oxopentanoate; 2-Oxo-5-amino-pentanoate; 2-Oxo-5-aminopentanoate; alpha-Keto-delta-aminopentanoate; 2-Oxo-5-aminovalerate$Biliverdin; Biliverdin IXalpha$Dehydroascorbate/Dehydroascorbate/cis-Aconitate; cis-Aconitic acid$3-Dehydro-L-gulonate/L-Iduronic acid/D-Glucuronate	0	7	0.37	
Propanoate metabolism	C18	2	2	0.04	(S)-3-Hydroxyisobutyrate/(R)-3-Hydroxybutanoate; (R)-3-Hydroxybutanoic acid; (R)-3-Hydroxybutyric acid/(S)-3-Hydroxybutanoate/4-Hydroxybutanoic acid; 4-Hydroxybutanoate; 4-Hydroxybutyric acid/2-Hydroxybutyrate$Dihydroxyacetone/D-Lactate/L-Lactate/3-Hydroxypropanoate; 3-Hydroxypropanoic acid; 3-Hydroxypropionate; 3-Hydroxypropionic acid; Hydracrylic acid/D-Glyceraldehyde	2	2	0.01	(S)-3-Hydroxyisobutyrate/(R)-3-Hydroxybutanoate; (R)-3-Hydroxybutanoic acid; (R)-3-Hydroxybutyric acid/(S)-3-Hydroxybutanoate/4-Hydroxybutanoic acid; 4-Hydroxybutanoate; 4-Hydroxybutyric acid/2-Hydroxybutyrate$Dihydroxyacetone/D-Lactate/L-Lactate/3-Hydroxypropanoate; 3-Hydroxypropanoic acid; 3-Hydroxypropionate; 3-Hydroxypropionic acid; Hydracrylic acid/D-Glyceraldehyde
Prostaglandin formation from arachidonate	C18	2	10	0.46	D-glucurono-6,3-lactone/L-Ascorbate/D-glucurono-6,3-lactone$Dehydroascorbate/Dehydroascorbate/cis-Aconitate; cis-Aconitic acid	0	10	0.37	
Prostaglandin formation from dihomo gama-linoleic acid	C18	0	3	0.50		0	3	0.37	
Proteoglycan biosynthesis	C18	0	1	0.50		0	1	0.37	
Purine metabolism	C18	7	25	0.36	Xanthine$D-Glutamate; D-Glutamic acid; D-Glutaminic acid; D-2-Aminoglutaric acid/L-4-Hydroxyglutamate semialdehyde/L-Glutamate$L-Ribulose 5-phosphate/alpha-D-Ribose 5-phosphate/alpha-D-Ribose 5-phosphate/alpha-D-Ribose 1-phosphate/alpha-D-Ribose 1-phosphate/D-Xylulose 5-phosphate/D-Ribulose 5-phosphate$L-Xylulose/D-Xylulose/L-Ribulose; L-erythro-Pentulose; L-Arabinoketose; L-Arabinulose; L-Riboketose/L-Arabinose/D-Xylose/D-Ribulose; D-erythro-2-Pentulose; D-Arabinoketose; D-Arabinulose; D-Riboketose/D-Ribose$D-glucurono-6,3-lactone/L-Ascorbate/D-glucurono-6,3-lactone$D-Glutamine; D-2-Aminoglutaramic acid/L-Glutamine/3-Ureidoisobutyrate$Inosine	2	25	0.37	$D-Glutamine; D-2-Aminoglutaramic acid/L-Glutamine/3-Ureidoisobutyrate

Putative anti-Inflammatory metabolites formation from EPA	C18	1	2	0.28	D-Glutamate; D-Glutamic acid; D-Glutaminic acid; D-2-Aminoglutaric acid/L-4-Hydroxyglutamate semialdehyde/L-Glutamate	0	2	0.37	
Pyrimidine metabolism	C18	9	14	0.00	D-Glutamine; D-2-Aminoglutaramic acid/L-Glutamine/3-Ureidoisobutyrate$3-Aminoisobutyric acid; 3-Aminoisobutanoate; 3-Amino-2-methylpropanoate/N,N-Dimethylglycine; Dimethylglycine/D-3-Amino-isobutanoate/(S)-2-Aminobutanoate; (S)-2-Aminobutanoic acid; (S)-2-Aminobutyric acid/4-Aminobutanoate/L-3-Amino-isobutanoate$L-Ribulose 5-phosphate/alpha-D-Ribose 5-phosphate/alpha-D-Ribose 5-phosphate/alpha-D-Ribose 1-phosphate/alpha-D-Ribose 1-phosphate/D-Xylulose 5-phosphate/D-Ribulose 5-phosphate$D-Hexose 6-phosphate/D-Glucose 1-phosphate/beta-D-Fructose 6-phosphate/D-Fructose 6-phosphate/Fructose 1-phosphate/alpha-D-Hexose 1-phosphate/beta-D-glucose 6-phosphate/1D-myo-Inositol 1-phosphate/alpha-D-Hexose 6-phosphate/D-Mannose 1-phosphate/D-Glucose 6-phosphate; Glucose 6-phosphate; Robison ester/Dolichyl phosphate D-mannose/1D-myo-Inositol 3-phosphate/D-Tagatose 6-phosphate/D-Fructose 1-phosphate/alpha-D-Glucose 6-phosphate/1D-myo-Inositol 4-phosphate/alpha-D-Galactose 1-phosphate/D-Mannose 6-phosphate/sorbitol 3-phosphate$Sarcosine; N-Methylglycine/D-Alanine/L-Alanine/beta-Alanine$D-Glutamate; D-Glutamic acid; D-Glutaminic acid; D-2-Aminoglutaric acid/L-4-Hydroxyglutamate semialdehyde/L-Glutamate$Deoxyuridine$Uridine$5,6-Dihydrothymine	4	14	0.08	$$D-Glutamine; D-2-Aminoglutaramic acid/L-Glutamine/3-Ureidoisobutyrate$D-Hexose 6-phosphate/D-Glucose 1-phosphate/beta-D-Fructose 6-phosphate/D-Fructose 6-phosphate/Fructose 1-phosphate/alpha-D-Hexose 1-phosphate/beta-D-glucose 6-phosphate/1D-myo-Inositol 1-phosphate/alpha-D-Hexose 6-phosphate/D-Mannose 1-phosphate/D-Glucose 6-phosphate; Glucose 6-phosphate; Robison ester/Dolichyl phosphate D-mannose/1D-myo-Inositol 3-phosphate/D-Tagatose 6-phosphate/D-Fructose 1-phosphate/alpha-D-Glucose 6-phosphate/1D-myo-Inositol 4-phosphate/alpha-D-Galactose 1-phosphate/D-Mannose 6-phosphate/sorbitol 3-phosphate
Pyruvate Metabolism	C18	2	4	0.18	Dihydroxyacetone/D-Lactate/L-Lactate/3-Hydroxypropanoate; 3-Hydroxypropanoic acid; 3-Hydroxypropionate; 3-Hydroxypropionic acid; Hydracrylic acid/D-Glyceraldehyde$L-Malate	1	4	0.24	Dihydroxyacetone/D-Lactate/L-Lactate/3-Hydroxypropanoate; 3-Hydroxypropanoic acid; 3-Hydroxypropionate; 3-Hydroxypropionic acid; Hydracrylic acid/D-Glyceraldehyde
R Group Synthesis	C18	0	1	0.50		0	1	0.37	
ROS Detoxification	C18	0	0	0.50		0	0	0.37	
Saturated fatty acids beta-oxidation	C18	0	1	0.50		0	1	0.37	
Selenoamino acid metabolism	C18	2	2	0.04	L-Serine/D-Serine$Sarcosine; N-Methylglycine/D-Alanine/L-Alanine/beta-Alanine	1	2	0.14	L-Serine/D-Serine

Sialic acid metabolism	C18	5	8	0.03	D-Hexose 6-phosphate/D-Glucose 1-phosphate/beta-D-Fructose 6-phosphate/D-Fructose 6-phosphate/Fructose 1-phosphate/alpha-D-Hexose 1-phosphate/beta-D-glucose 6-phosphate/1D-myo-Inositol 1-phosphate/alpha-D-Hexose 6-phosphate/D-Mannose 1-phosphate/D-Glucose 6-phosphate; Glucose 6-phosphate; Robison ester/Dolichyl phosphate D-mannose/1D-myo-Inositol 3-phosphate/D-Tagatose 6-phosphate/D-Fructose 1-phosphate/alpha-D-Glucose 6-phosphate/1D-myo-Inositol 4-phosphate/alpha-D-Galactose 1-phosphate/D-Mannose 6-phosphate/sorbitol 3-phosphate$L-Serine/D-Serine$D-Tagatose; lyxo-Hexulose/1,7-Dimethylxanthine; Paraxanthine/beta-D-Glucose/D-Fructose/alpha-D-Glucose//D-Glucose/myo-Inositol/D-Hexose/beta-D-Galactose/alpha-D-Galactose/L-Sorbose; L-xylo-Hexulose//Galactose/D-Mannose/beta-D-Fructose; beta-Fruit sugar; beta-D-arabino-Hexulose; beta-Levulose; Fructose/Glucose/D-Galactose$3-beta-D-Galactosyl-sn-glycerol; Galactosylglycerol$3-Keto-beta-D-galactose/L-Gulono-1,4-lactone; L-Gulono-gamma-lactone; gamma-Gulonolactone; L-Gulonic acid gamma-lactone; L-Gulonolactone/D-Glucono-1,5-lactone; Gluconic lactone; Gluconic acid lactone; 1,5-Gluconolactone; delta-Gluconolactone; D-Gluconolactone; Gluconolactone	5	8	0.00	N-Acetylneuraminate; N-Acetylneuraminic acid; 5-Acetamido-3,5-dideoxy-D-glycero-D-galacto-2-nonulosonic acid; Neu5Ac$L-Serine/D-Serine$D-Tagatose; lyxo-Hexulose/1,7-Dimethylxanthine; Paraxanthine/beta-D-Glucose/D-Fructose/alpha-D-Glucose//D-Glucose/myo-Inositol/D-Hexose/beta-D-Galactose/alpha-D-Galactose/L-Sorbose; L-xylo-Hexulose//Galactose/D-Mannose/beta-D-Fructose; beta-Fruit sugar; beta-D-arabino-Hexulose; beta-Levulose; Fructose/Glucose/D-Galactose$3-beta-D-Galactosyl-sn-glycerol; Galactosylglycerol$D-Hexose 6-phosphate/D-Glucose 1-phosphate/beta-D-Fructose 6-phosphate/D-Fructose 6-phosphate/Fructose 1-phosphate/alpha-D-Hexose 1-phosphate/beta-D-glucose 6-phosphate/1D-myo-Inositol 1-phosphate/alpha-D-Hexose 6-phosphate/D-Mannose 1-phosphate/D-Glucose 6-phosphate; Glucose 6-phosphate; Robison ester/Dolichyl phosphate D-mannose/1D-myo-Inositol 3-phosphate/D-Tagatose 6-phosphate/D-Fructose 1-phosphate/alpha-D-Glucose 6-phosphate/1D-myo-Inositol 4-phosphate/alpha-D-Galactose 1-phosphate/D-Mannose 6-phosphate/sorbitol 3-phosphate
Sphingolipid metabolism	C18	0	0	0.50		0	0	0.37	
Squalene and cholesterol biosynthesis	C18	1	6	0.47		1	6	0.30	

Starch and Sucrose Metabolism	C18	3	4	0.04	D-Hexose 6-phosphate/D-Glucose 1-phosphate/beta-D-Fructose 6-phosphate/D-Fructose 6-phosphate/Fructose 1-phosphate/alpha-D-Hexose 1-phosphate/beta-D-glucose 6-phosphate/1D-myo-Inositol 1-phosphate/alpha-D-Hexose 6-phosphate/D-Mannose 1-phosphate/D-Glucose 6-phosphate; Glucose 6-phosphate; Robison ester/Dolichyl phosphate D-mannose/1D-myo-Inositol 3-phosphate/D-Tagatose 6-phosphate/D-Fructose 1-phosphate/alpha-D-Glucose 6-phosphate/1D-myo-Inositol 4-phosphate/alpha-D-Galactose 1-phosphate/D-Mannose 6-phosphate/sorbitol 3-phosphate$D-Tagatose; lyxo-Hexulose/1,7-Dimethylxanthine; Paraxanthine/beta-D-Glucose/D-Fructose/alpha-D-Glucose//D-Glucose/myo-Inositol/D-Hexose/beta-D-Galactose/alpha-D-Galactose/L-Sorbose; L-xylo-Hexulose//Galactose/D-Mannose/beta-D-Fructose; beta-Fruit sugar; beta-D-arabino-Hexulose; beta-Levulose; Fructose/Glucose/D-Galactose$Cellulose; (1,4-beta-D-Glucosyl)n; (1,4-beta-D-Glucosyl)n+1; (1,4-beta-D-Glucosyl)n-1; 1,4-beta-D-Glucan; Microcrystalline cellulose	3	4	0.01	Cellulose; (1,4-beta-D-Glucosyl)n; (1,4-beta-D-Glucosyl)n+1; (1,4-beta-D-Glucosyl)n-1; 1,4-beta-D-Glucan; Microcrystalline cellulose$D-Tagatose; lyxo-Hexulose/1,7-Dimethylxanthine; Paraxanthine/beta-D-Glucose/D-Fructose/alpha-D-Glucose//D-Glucose/myo-Inositol/D-Hexose/beta-D-Galactose/alpha-D-Galactose/L-Sorbose; L-xylo-Hexulose//Galactose/D-Mannose/beta-D-Fructose; beta-Fruit sugar; beta-D-arabino-Hexulose; beta-Levulose; Fructose/Glucose/D-Galactose$D-Hexose 6-phosphate/D-Glucose 1-phosphate/beta-D-Fructose 6-phosphate/D-Fructose 6-phosphate/Fructose 1-phosphate/alpha-D-Hexose 1-phosphate/beta-D-glucose 6-phosphate/1D-myo-Inositol 1-phosphate/alpha-D-Hexose 6-phosphate/D-Mannose 1-phosphate/D-Glucose 6-phosphate; Glucose 6-phosphate; Robison ester/Dolichyl phosphate D-mannose/1D-myo-Inositol 3-phosphate/D-Tagatose 6-phosphate/D-Fructose 1-phosphate/alpha-D-Glucose 6-phosphate/1D-myo-Inositol 4-phosphate/alpha-D-Galactose 1-phosphate/D-Mannose 6-phosphate/sorbitol 3-phosphate
TCA cycle	C18	5	9	0.04	2-Oxoglutarate$Dihydrolipoamide; Dihydrothioctamide$Dehydroascorbate/Dehydroascorbate/cis-Aconitate; cis-Aconitic acid$L-Malate$Isocitrate/Citrate/	0	9	0.37	
Trihydroxycoprostanoyl-CoA beta-oxidation	C18	0	0	0.50		0	0	0.37	

Tryptophan metabolism	C18	9	18	0.03	D-Glutamate; D-Glutamic acid; D-Glutaminic acid; D-2-Aminoglutaric acid/L-4-Hydroxyglutamate semialdehyde/L-Glutamate$2-Oxoglutarate$5-hydroxytryptophol/1,2-dehydrosalsolinol$L-Kynurenine/Formyl-5-hydroxykynurenamine$D-Tagatose; lyxo-Hexulose/1,7-Dimethylxanthine; Paraxanthine/beta-D-Glucose/D-Fructose/alpha-D-Glucose//D-Glucose/myo-Inositol/D-Hexose/beta-D-Galactose/alpha-D-Galactose/L-Sorbose; L-xylo-Hexulose//Galactose/D-Mannose/beta-D-Fructose; beta-Fruit sugar; beta-D-arabino-Hexulose; beta-Levulose; Fructose/Glucose/D-Galactose$methyl indole-3-acetate$/Indole-3-acetaldehyde$Sarcosine; N-Methylglycine/D-Alanine/L-Alanine/beta-Alanine$L-Tryptophan/D-Tryptophan	3	18	0.27	L-Tryptophan/D-Tryptophan$D-Tagatose; lyxo-Hexulose/1,7-Dimethylxanthine; Paraxanthine/beta-D-Glucose/D-Fructose/alpha-D-Glucose//D-Glucose/myo-Inositol/D-Hexose/beta-D-Galactose/alpha-D-Galactose/L-Sorbose; L-xylo-Hexulose//Galactose/D-Mannose/beta-D-Fructose; beta-Fruit sugar; beta-D-arabino-Hexulose; beta-Levulose; Fructose/Glucose/D-Galactose$Formyl-5-hydroxykynurenamine
Tyrosine metabolism	C18	14	36	0.06	D-Glutamine; D-2-Aminoglutaramic acid/L-Glutamine/3-Ureidoisobutyrate$2-Oxoglutarate$5-hydroxytryptophol/1,2-dehydrosalsolinol$L-Tyrosine$D-glucurono-6,3-lactone/L-Ascorbate/D-glucurono-6,3-lactone$$Sulfate$2-Phenyl-1,3-propanediol monocarbamate$D-Glutamate; D-Glutamic acid; D-Glutaminic acid; D-2-Aminoglutaric acid/L-4-Hydroxyglutamate semialdehyde/L-Glutamate$$Dehydroascorbate/Dehydroascorbate/cis-Aconitate; cis-Aconitic acid$$Sarcosine; N-Methylglycine/D-Alanine/L-Alanine/beta-Alanine$3-Iodo-L-tyrosine	5	36	0.33	D-Glutamine; D-2-Aminoglutaramic acid/L-Glutamine/3-Ureidoisobutyrate$$L-Tyrosine$$
Ubiquinone Biosynthesis	C18	0	2	0.50		0	2	0.37	

Urea cycle/amino group metabolism	C18	12	21	0.00	D-Glutamate; D-Glutamic acid; D-Glutaminic acid; D-2-Aminoglutaric acid/L-4-Hydroxyglutamate semialdehyde/L-Glutamate$Peptide 2-(3-carboxy-3-aminopropyl)-L-histidine$L-Citrulline; 2-Amino-5-ureidovaleric acid; Citrulline$3-Aminoisobutyric acid; 3-Aminoisobutanoate; 3-Amino-2-methylpropanoate/N,N-Dimethylglycine; Dimethylglycine/D-3-Amino-isobutanoate/(S)-2-Aminobutanoate; (S)-2-Aminobutanoic acid; (S)-2-Aminobutyric acid/4-Aminobutanoate/L-3-Amino-isobutanoate$Thiopurine/6-Mercaptopurin; Mercaptopurine$Sarcosine; N-Methylglycine/D-Alanine/L-Alanine/beta-Alanine$5-Aminolevulinate; 5-Amino-4-oxopentanoate; 5-Amino-4-oxovaleric acid/cis-4-Hydroxy-D-proline/2-Amino-4-oxopentanoic acid; 2-Amino-4-oxopentanoate/L-Glutamate 5-semialdehyde; L-Glutamate gamma-semialdehyde/trans-4-Hydroxy-L-proline/5-Amino-2-oxopentanoic acid; 5-Amino-2-oxopentanoate; 2-Oxo-5-amino-pentanoate; 2-Oxo-5-aminopentanoate; alpha-Keto-delta-aminopentanoate; 2-Oxo-5-aminovalerate$D-Arginine; D-2-Amino-5-guanidinovaleric acid/L-Arginine$2-Oxoglutarate$D-Proline/acetamidopropanal/L-Proline$N-Acetyl-L-glutamate; N-Acetyl-L-glutamic acid$N4-Acetylaminobutanal/L-Pipecolate; Pipecolinic acid; Pipecolic acid; 2-Piperidinecarboxylic acid	3	21	0.31	$$
Valine, leucine and isoleucine degradation	C18	5	7	0.01	D-Glutamate; D-Glutamic acid; D-Glutaminic acid; D-2-Aminoglutaric acid/L-4-Hydroxyglutamate semialdehyde/L-Glutamate$2-Oxoglutarate$(S)-3-Hydroxyisobutyrate/(R)-3-Hydroxybutanoate; (R)-3-Hydroxybutanoic acid; (R)-3-Hydroxybutyric acid/(S)-3-Hydroxybutanoate/4-Hydroxybutanoic acid; 4-Hydroxybutanoate; 4-Hydroxybutyric acid/2-Hydroxybutyrate$3-Hydroxy-2-methylpropanoate; 3-Hydroxyisobutyrate; 3-Hydroxyisobutyric acid$3-Aminoisobutyric acid; 3-Aminoisobutanoate; 3-Amino-2-methylpropanoate/N,N-Dimethylglycine; Dimethylglycine/D-3-Amino-isobutanoate/(S)-2-Aminobutanoate; (S)-2-Aminobutanoic acid; (S)-2-Aminobutyric acid/4-Aminobutanoate/L-3-Amino-isobutanoate	3	7	0.03	3-Hydroxy-2-methylpropanoate; 3-Hydroxyisobutyrate; 3-Hydroxyisobutyric acid$(S)-3-Methyl-2-oxopentanoate/Adipate semialdehyde; Hexan-1-one-6-carboxylate; 6-Oxohexanoate/3-Methyl-2-oxopentanoate; 2-Oxo-3-methylvalerate/4-Methyl-2-oxopentanoate$(S)-3-Hydroxyisobutyrate/(R)-3-Hydroxybutanoate; (R)-3-Hydroxybutanoic acid; (R)-3-Hydroxybutyric acid/(S)-3-Hydroxybutanoate/4-Hydroxybutanoic acid; 4-Hydroxybutanoate; 4-Hydroxybutyric acid/2-Hydroxybutyrate
Vitamin A (retinol) metabolism	C18	1	9	0.49		0	9	0.37	
Vitamin B1 (thiamin) metabolism	C18	1	3	0.36	2-Oxoglutarate	0	3	0.37	
Vitamin B12 (cyanocobalamin) metabolism	C18	0	0	0.50		0	0	0.37	
Vitamin B2 (riboflavin) metabolism	C18	0	0	0.50		0	0	0.37	

Vitamin B3 (nicotinate and nicotinamide) metabolism	C18	4	7	0.05	D-Glutamate; D-Glutamic acid; D-Glutaminic acid; D-2-Aminoglutaric acid/L-4-Hydroxyglutamate semialdehyde/L-Glutamate$D-Glutamine; D-2-Aminoglutaramic acid/L-Glutamine/3-Ureidoisobutyrate$D-Arginine; D-2-Amino-5-guanidinovaleric acid/L-Arginine$L-Ribulose 5-phosphate/alpha-D-Ribose 5-phosphate/alpha-D-Ribose 5-phosphate/alpha-D-Ribose 1-phosphate/alpha-D-Ribose 1-phosphate/D-Xylulose 5-phosphate/D-Ribulose 5-phosphate	1	7	0.32	D-Glutamine; D-2-Aminoglutaramic acid/L-Glutamine/3-Ureidoisobutyrate
Vitamin B5 - CoA biosynthesis from pantothenate	C18	0	1	0.50		0	1	0.37	
Vitamin B6 (pyridoxine) metabolism	C18	0	2	0.50		0	2	0.37	
Vitamin B9 (folate) metabolism	C18	2	4	0.18	D-Glutamate; D-Glutamic acid; D-Glutaminic acid; D-2-Aminoglutaric acid/L-4-Hydroxyglutamate semialdehyde/L-Glutamate$L-Serine/D-Serine	1	4	0.24	L-Serine/D-Serine
Vitamin D	C18	0	0	0.50		0	0	0.37	
Vitamin D3 (cholecalciferol) metabolism	C18	1	3	0.36		1	3	0.20	
Vitamin E metabolism	C18	1	6	0.47		0	6	0.37	
Vitamin H (biotin) metabolism	C18	0	1	0.50		0	1	0.37	
Vitamin K metabolism	C18	0	0	0.50		0	0	0.37	
Xenobiotics metabolism	C18	4	10	0.18	Chloral hydrate$$$/Indole-3-acetaldehyde	1	10	0.35	

Supplementary Table 6. Annotation of metabolic pathways.

Pathways	Participant Group	Chemical Group	Energy Metabolism	Reference	DNA/RNA	Reference	Nitrogen balance	Reference	Connective Tissue	Reference	Antioxidant	Reference	Immune System	Reference	Signaling Pathway	Reference	Glycolysis	Reference	Urea Cycle	Reference	TCA Cycle	Reference
Alanine and Aspartate Metabolism	PD Patient	Amino Acid	1	Engelking 2015	0		1	Kohlmeier 2015	0		0		0		0		1	Harris 2021	0		1	Martínez-Reyes and Chandel 2020
Aldarate Metabolism	PD Patient	Simple Sugar	1	Pal 2020	0		0		0		0		0		0		0		0		0	
Aminosugars metabolism	PD Patient	Aminosugars	0		0		0		0		0		1	Varki 2008	0		0		0		0	
Arginine and Proline Metabolism	PD Patient	Amino Acid	1	Engelking 2015	0		1	Kohlmeier 2015	1	Karna et al., 2020	1		0		0		0		1	Barmore et al., 2022	0	
Ascorbate (Vitamin C) Metabolism	PD Patient	Vitamin	0		0		0		1	Padayatty and Levine 2016	1	Padayatty and Levine 2016	0		0		0		0		0	
Aspartate and asparagine metabolism	PD Patient	Amino Acid	1	Engelking 2015	0		1	Kohlmeier 2015	0		0		0		0		0		1	Barmore et al., 2022	0	
Beta-Alanine metabolism	PD Patient	Amino Acid	1	Engelking 2015	0		1	Kohlmeier 2015	0		0		0		0		0		0		0	
Butanoate metabolism	PD Patient	Fatty Acid	1	Panov et al., 2014	0		0		0		0		0		0		0		0		0	
Caffeine metabolism	PD Patient	Other	0		0		0		0		0		0		0		0		0		0	
Chondroitin sulfate degradation	PD Patient	Glycosaminoglycan	0		0		0		1	Tang et al., 2019	0		0		0		0		0		0	
CoA Catabolism	PD Patient	Other	1	Martínez-Reyes and Chandel 2020	0		0		0		0		0		0		0		0		1	Martínez-Reyes and Chandel 2020
Fructose and mannose metabolism	PD Patient	Simple Sugar	1	Pal 2020	0		0		0		1	Girard et al., 2006	0		0		0		0		0	
Galactose metabolism	PD Patient	Simple Sugar	1	Pal 2020	0		0		0		0		0		0		0		0		0	
Glutamate metabolism	PD Patient	Amino Acid	1	Engelking 2015	0		1	Kohlmeier 2015	0		1		0		0		0		1		0	
Glutathione Metabolism	PD Patient	Amino Acid	1	Engelking 2015	0		1	Kohlmeier 2015	0		1	Netto et al., 2007	1	Zavašnik-Bergant and Turk 2006	0		0		0		0	
Glycine, serine, alanine and threonine metabolism	PD Patient	Amino Acid	1	Engelking 2015	0		1	Kohlmeier 2015	0		1	Wang et al., 2013	0		0		0		0		0	
Glycolysis and Gluconeogenesis	PD Patient	Simple Sugar	1	Pal 2020	0		0		0		0		0		0		1	Harris 2021	0		0	
Glycosphingolipid biosynthesis - ganglioseries	PD Patient	Glycosphingolipid	0		0		0		0		0		1	Nakayama et al., 2018	1	Chatterjee and Wei 2003	0		0		0	
Glycosphingolipid biosynthesis - globoseries	PD Patient	Glycosphingolipid	0		0		0		0		0		1	Nakayama et al., 2018	1	Chatterjee and Wei 2003	0		0		0	
Heparan sulfate degradation	PD Patient	Glycosaminoglycan	0		0		0		1	Tang et al., 2019	0		1	Collins and Troeberg 2019	1	Ohtsubo and Marth 2006	0		0		0	
Hexose phosphorylation	PD Patient	Simple Sugar	1	Pal 2020	0		0		0		0		0		0		0		0		0	
Histidine metabolism	PD Patient	Amino Acid	1	Engelking 2015	0		1	Kohlmeier 2015	0		0		0		0		0		0		0	
Lysine metabolism	PD Patient	Amino Acid	1	Engelking 2015	0		1	Kohlmeier 2015	0		0		0		0		0		0		0	
Methionine and cysteine metabolism	PD Patient	Amino Acid	1	Engelking 2015	0		1	Kohlmeier 2015	0		1	Netto et al., 2007; Martínez et al., 2017	1	Zavašnik-Bergant and Turk 2006; Martínez et al., 2017	0		0		0		0	

Mono-unsaturated fatty acid beta-oxidation	PD Patient	Fatty Acid	1	Panov et al,. 2014	0		0		0		0		0		0		0		0		0	
N-Glycan Degradation	PD Patient	N-Glycan	0		0		0		0		0		1	Maverakis et al., 2014	0		0		0		0	
Nitrogen metabolism	PD Patient	Other	1	Wright 2011	0		1	Kohlmeier 2015	0		0		0		0		0		0		0	
Pentose and Glucuronate Interconversions	PD Patient	Simple Sugar	1	Pal 2020	1	Fornalewicz et al., 2017	0		0		0		1	Britt et al., 2022	0		0		0		0	
Pentose phosphate pathway	PD Patient	Simple Sugar	1	Pal 2020	1	Fornalewicz et al., 2017	0		0		0		1	Britt et al., 2022	0		0		0		0	
Phosphatidylinositol phosphate metabolism	PD Patient	Glycerophospholipid	0		0		0		0		0		1	Nakayama et al., 2018	1	Chatterjee and Wei 2003	0		0		0	
Porphyrin metabolism	PD Patient	Other	1		0		0		0		0		0		0		0		0		0	
Propanoate metabolism	PD Patient	Fatty Acid	1	Panov et al., 2014	0		0		0		0		0		0		0		0		0	
Purine metabolism	PD Patient	Nucleic Acid	0		1	Dudley et al., 2010	0		0		0		1	Peeters et al., 2021	0		0		0		0	
Pyrimidine metabolism	PD Patient	Nucleic Acid	0		1	Dudley et al., 2010	0		0		0		1	Peeters et al., 2021	0		0		0		0	
Selenoamino acid metabolism	PD Patient	Amino Acid	0		0		1	Kohlmeier 2015	0		0		1	Ferreira et al. 2021	1	McKenzie et al., 2002	0		0		0	
Sialic acid metabolism	PD Patient	Aminosugars	0		0		0		0		0		1	Varki 2008	0		0		0		0	
Starch and Sucrose Metabolism	PD Patient	Complex Sugar	1	Lovegrove et al., 2017	0		0		0		0		0		0		0		0		0	
TCA cycle	PD Patient	Other	1	Martínez-Reyes and Chandel 2020	0		0		0		0		0		0		0		0		1	Martínez-Reyes and Chandel 2020
Tryptophan metabolism	PD Patient	Amino Acid	1	Engelking 2015	0		1	Kohlmeier 2015	0		1	Xu et al., 2018	1	Sorgdrager et al., 2019	0		0		0		0	
Urea cycle/amino group metabolism	PD Patient	Other	1	Barmore et al., 2022	0		0		0		0		0		0		0		1	Barmore et al., 2022	0	
Valine, leucine and isoleucine degradation	PD Patient	Amino Acid	1	Engelking 2015	0		1	Kohlmeier 2015	0		0		1	Ananieva et al., 2016	0		0		0		0	
Vitamin B3 (nicotinate and nicotinamide) metabolism	PD Patient	Vitamin	1	Alice Callahan et al. 2020	0		0		0		0		0		0		0		0		0	
Vitamin B5 - CoA biosynthesis from pantothenate	PD Patient	Vitamin	1	Alice Callahan et al. 2020	0		0		0		0		0		0		0		0		1	Martínez-Reyes and Chandel 2020
Xenobiotics metabolism	PD Patient	Other	0		0		0		0		0		0		0		0		0		0	
Aminosugars metabolism	non-PD	Aminosugars	0		0		0		0		0		1	Varki 2008	0		0		0		0	
Caffeine metabolism	non-PD	Other	0		0		0		0		0		0		0		0		0		0	
Chondroitin sulfate degradation	non-PD	Glycosaminoglycan	0		0		0		1	Tang et al., 2019	0		0		0		0		0		0	
Fructose and mannose metabolism	non-PD	Simple Sugar	1	Pal 2020	0		0		0		1	Girard et al., 2006	0		0		0		0		0	
Galactose metabolism	non-PD	Simple Sugar	1	Pal 2020	0		0		0		0		0		0		0		0		0	
Glycerophospholipid metabolism	non-PD	Glycerophospholipid	0		0		0		0		0		0		1	Blanco and Blanco 2017	0		0		0	
Glycolysis and Gluconeogenesis	non-PD	Simple Sugar	1	Pal 2020	0		0		0		0		0		0		1	Harris 2021	0		0	
Glycosphingolipid biosynthesis - ganglioseries	non-PD	Glycosphingolipid	0		0		0		0		0		1	Nakayama et al., 2018	1	Chatterjee and Wei	0		0		0	

																2003			
Glycosphingolipid biosynthesis - globoseries	non-PD	Glycosphingolipid	0		0		0		0		0		1	Nakayama et al., 2018	1	Chatterjee and Wei 2003	0	0	0
Glycosphingolipid metabolism	non-PD	Glycosphingolipid	0		0		0		0		0		1	Nakayama et al., 2018	1	Chatterjee and Wei 2003	0	0	0
Heparan sulfate degradation	non-PD	Glycosaminoglycan	0		0		0		1	Tang et al., 2019	0		1	Collins and Troeberg 2019	1	Ohtsubo and Marth 2006	0	0	0
Hexose phosphorylation	non-PD	Simple Sugar	1	Pal 2020	0		0		0		0		0		0		0	0	0
Keratan sulfate degradation	non-PD	Glycosaminoglycan	0		0		0		1	Tang et al., 2019	0		1	Caterson and Melrose 2018	1	Ohtsubo and Marth 2006	0	0	0
N-Glycan biosynthesis	non-PD	N-Glycan	0		0		0		0		0		1	Maverakis et al., 2014	0		0	0	0
N-Glycan Degradation	non-PD	N-Glycan	0		0		0		0		0		1	Maverakis et al., 2014	0		0	0	0
Pentose phosphate pathway	non-PD	Simple Sugar	1	Pal 2020	1	Fornalewicz et al., 2017	0		0		0		1	Britt et al., 2022	0		0	0	0
Propanoate metabolism	non-PD	Fatty Acid	1	Panov et al., 2014	0		0		0		0		0		0		0	0	0
Sialic acid metabolism	non-PD	Aminosugars	0		0		0		0		0		1	Varki 2008	0		0	0	0
Starch and Sucrose Metabolism	non-PD	Complex Sugar	1	Lovegrove et al., 2017	0		0		0		0		0		0		0	0	0
Valine, leucine and isoleucine degradation	non-PD	Amino Acid	1	Engelking 2015	0		1	Kohlmeier 2015	0		0		1	Ananieva et al., 2016	0		0	0	0
Vitamin B2 (riboflavin) metabolism	non-PD	Vitamin	1	Alice Callahan et al. 2020	0		0		0		0		0		0		0	0	0
Xenobiotics metabolism	non-PD	Other	0		0		0		0		0		0		0		0	0	0

Supplementary Table 7. Metabolic pathways associated with diabetes mellitus (DM) using metabolites among non-PD elder participants using significance level of 0.15 for pathway enrichment analysis.

Pathways	overlap size	pathway size	p-value	Column
Fructose and mannose metabolism	4	4	0.00092	HILIC
Glycosphingolipid biosynthesis - ganglioseries	3	3	0.00294	HILIC
Glycosphingolipid biosynthesis - globoseries	3	3	0.00294	HILIC
Keratan sulfate degradation	3	4	0.01134	HILIC
Saturated fatty acids beta-oxidation	3	4	0.01134	HILIC
Carnitine shuttle	10	24	0.0158	HILIC
Hyaluronan Metabolism	2	2	0.01655	HILIC
N-Glycan biosynthesis	2	2	0.01655	HILIC
N-Glycan Degradation	2	2	0.01655	HILIC
Pentose phosphate pathway	2	2	0.01655	HILIC
Galactose metabolism	3	5	0.03344	HILIC
Hexose phosphorylation	3	5	0.03344	HILIC
Fatty acid oxidation	3	5	0.03344	HILIC
Phosphatidylinositol phosphate metabolism	4	8	0.03966	HILIC
Fructose and mannose metabolism	5	5	0.00025	C18
Glycerophospholipid metabolism	9	18	0.00403	C18
N-Glycan Degradation	3	3	0.00521	C18
Sialic acid metabolism	5	8	0.00891	C18
Galactose metabolism	5	8	0.00891	C18
Starch and Sucrose Metabolism	3	4	0.0163	C18
Propanoate metabolism	2	2	0.0242	C18
Glycolysis and Gluconeogenesis	4	7	0.02437	C18
Hexose phosphorylation	3	5	0.04025	C18
Keratan sulfate degradation	3	5	0.04025	C18
Caffeine metabolism	3	5	0.04025	C18

Supplementary Table 8. Overlapping metabolites associated with type 2 diabetes mellitus and Parkinson's diseases.

						MWAS Results for Type 2 Diabetes among PD Patients			MWAS Results for Parkinson's Disease		
**Chemical Name**	**Library**	**Indicator of Match**	**Mass to Charge Ratio**	**Retention Time**	**column**	**OR [95% CI]**	**p value**	**FDR p value**	**OR [95% CI]**	**p value**	**FDR p value**
Indoleacetaldehyde	in-house	unique	158.061	87.220	C18	0.61 [0.48, 0.78]	0.00	**0.00**	0.69 [0.5, 0.94]	0.02	0.16
L-Glutamic acid	in-house	unique	148.060	89.479	HILIC	1.47 [1.18,1.84]	0.00	**0.02**	1.63 [1.29,2.08]	0.00	**0.01**
6-Chloro-N-(1-methylethyl)-135-triazine-24-diamine/Indoleacrylic acid	xMSannotator	multiple	188.071	59.806	HILIC	0.68 [0.54,0.87]	0.00	**0.03**	0.47 [0.3,0.75]	0.00	**0.04**
3-Methyldioxyindole	in-house	unique	162.056	40.608	C18	0.67 [0.53, 0.87]	0.00	**0.04**	1.13 [1.02, 1.25]	0.02	0.16
3-Hydroxymethylantipyrine/4-Hydroxyantipyrine/D-Tryptophan/L-Tryptophan/Nirvanol/S-nirvanol/Ethotoin/()-Tryptophan	xMSannotator	multiple	205.097	59.833	HILIC	0.7 [0.55,0.89]	0.00	**0.05**	0.46 [0.3,0.71]	0.00	**0.02**
N-Methyl-D-aspartic acid/O-Acetylserine/L-Glutamic acid/N-Acetylserine/3-(Carboxymethylamino)propanoic acid/D-Glutamic acid/L-4-Hydroxyglutamate semialdehyde/DL-Glutamate	xMSannotator	multiple	192.024	88.823	HILIC	1.38 [1.1,1.73]	0.01	0.07	1.43 [1.07,1.9]	0.02	0.18
Uridine	in-house	unique	243.062	33.439	C18	1.38 [1.09, 1.73]	0.01	0.08	1.34 [1.06, 1.68]	0.01	0.12
L-Serine	in-house	unique	104.035	30.101	C18	0.75 [0.59, 0.94]	0.01	0.13	2.2 [1.39, 3.51]	0.00	**0.02**
Oxoglutaric acid	in-house	unique	145.014	28.164	C18	1.3 [1.04, 1.63]	0.02	0.17	1.61 [1.31, 1.98]	0.00	**0.00**
L-Threonine/L-Homoserine/L-Allothreonine	in-house	multiple	120.066	87.232	HILIC	0.77 [0.61,0.97]	0.03	0.18	1.57 [1.12,2.19]	0.01	0.13
gamma-Aminobutyric acid/2-Aminoisobutyric acid/3-Aminoisobutanoic acid/gamma-Aminobutyric acid	in-house	multiple	102.056	29.529	C18	1.28 [1.02, 1.61]	0.03	0.20	1.46 [1.12, 1.9]	0.01	0.08
Squamolone/L-Cyclo(alanylglycyl)/Dihydrothymine	xMSannotator	multiple	173.030	86.515	HILIC	0.75 [0.57,0.98]	0.03	0.21	1.4 [1.12,1.76]	0.00	0.07
4-(Methylthio)-1-butanol/4-(Methylthio)-2-butanol/3-Mercapto-3-methyl-1-butanol/3-Mercapto-2-methyl-1-butanol/xi-2-Mercapto-3-methyl-1-butanol	xMSannotator	multiple	121.069	87.491	HILIC	0.79 [0.63,0.99]	0.04	0.24	1.83 [1.28,2.63]	0.00	**0.04**

Supplementary Table 8. Type 2 diabetes mellitus associated metabolites that overlapped with main analysis stratified by sex among Parkinson's disease patients.

HILIC

		Male (n = 524)						Female (n = 307)					
mz	rt	beta	se	zvalue	pvalue	Full MWAS FDR p value	Replication FDR p value	beta	se	zvalue	pvalue	Full MWAS FDR p value	Replication FDR p value
335.094	86.335	1.053515768	0.16026641	6.573528235	4.91368E-11	1.43135E-07	9.43426E-09	0.71853583	0.220809778	3.254094249	0.001137545	0.236690681	0.02184087
130.136	49.642	0.969509317	0.157273524	6.164478877	7.07156E-10	1.02997E-06	6.7887E-08	1.207443743	0.240804915	5.01419891	5.32549E-07	0.001551314	0.000102249
496.686	67.924	-1.029332293	0.186291227	-5.525393279	3.28748E-08	3.19215E-05	2.10399E-06	-0.127521046	0.197176297	-0.646736188	0.517802698	0.936389262	0.643661932
204.056	74.501	0.736820729	0.141115915	5.221386472	1.77588E-07	0.000129329	8.52425E-06	0.557316736	0.184252437	3.02474553	0.002488424	0.329489958	0.031851827
203.053	75.296	0.720265473	0.14087455	5.112814723	3.17394E-07	0.000184913	1.21879E-05	0.533112615	0.182011022	2.9290128	0.003400404	0.380976055	0.036270978
221.988	85.683	-0.761736288	0.154231398	-4.938918389	7.85571E-07	0.000381395	2.51383E-05	-0.421344897	0.221883552	-1.898946056	0.057571569	0.714657298	0.184229021
322.81	69.374	-0.818297756	0.172419526	-4.745969163	2.0751E-06	0.000804859	5.30494E-05	0.025177977	0.200287571	0.125709135	0.89996217	0.993360688	0.92402533
744.59	42.524	-0.760991669	0.160778407	-4.733170851	2.21039E-06	0.000804859	5.30494E-05	-0.571765942	0.23157307	-2.469051954	0.013547155	0.497258147	0.089691508
326.805	68.896	-0.811138204	0.172456068	-4.703448325	2.55804E-06	0.000827952	5.45715E-05	0.019904044	0.200943347	0.099053012	0.92109618	0.993360688	0.935716754
444.719	68.358	-0.757147579	0.16432108	-4.607732487	4.07084E-06	0.001078032	7.10546E-05	0.175720069	0.232401603	0.756105239	0.449586101	0.918233633	0.61220235
668.521	68.55	-0.755177543	0.163375273	-4.62234909	3.79419E-06	0.001078032	7.10546E-05	-0.244380702	0.214733869	-1.138063148	0.255094115	0.861528104	0.445255182
258.15	74.904	-0.598159647	0.131792543	-4.538645602	5.66167E-06	0.00137437	9.05867E-05	-0.272391819	0.192018158	-1.41857323	0.156023475	0.803344788	0.354053935
231.055	75.316	-0.69621888	0.15432252	-4.511453562	6.43849E-06	0.001442717	9.50915E-05	-0.256984745	0.201834	-1.27324804	0.202930038	0.815358899	0.381985955
508.711	67.545	-0.701857782	0.157129609	-4.46674429	7.9419E-06	0.001652482	0.000108917	0.00410715	0.198527025	0.020688117	0.983494448	0.997534585	0.988643634
190.119	93.248	0.636032913	0.145739749	4.36416914	1.27607E-05	0.002478124	0.000163337	0.35946748	0.217861737	1.649979873	0.098947053	0.768620706	0.271397631
257.147	74.625	-0.671955221	0.154771382	-4.341598642	1.4145E-05	0.002575269	0.00016974	-0.261441464	0.207520744	-1.259832916	0.207729643	0.823953408	0.387224189
745.593	42.325	-0.679549315	0.157090831	-4.325836926	1.51954E-05	0.002603773	0.000171618	-0.118694877	0.178734312	-0.664085571	0.506635545	0.936173443	0.643661932
672.558	68.111	-0.651752873	0.151438864	-4.303735876	1.67942E-05	0.002717859	0.000179138	-0.059005973	0.210513415	-0.280295549	0.779250765	0.98151707	0.831200816
402.083	87.844	-0.596921325	0.139200722	-4.288205673	1.80122E-05	0.002761559	0.000182018	-0.083956731	0.204706256	-0.41013271	0.681708599	0.967501194	0.756171993
313.154	42.894	-0.654249504	0.153098144	-4.273399307	1.92515E-05	0.002803985	0.000184815	-0.45084702	0.173840203	-2.593456588	0.009501653	0.480537095	0.07931815
261.043	87.544	-0.571018616	0.136415707	-4.185871481	2.84074E-05	0.003766952	0.000248285	-0.153707769	0.177066517	-0.868079248	0.385350951	0.90173972	0.546248259
382.766	68.554	-0.704663269	0.16835674	-4.185536438	2.84493E-05	0.003766952	0.000248285	-0.041877084	0.205566857	-0.203715155	0.838576099	0.989638608	0.875035929
165.033	87.089	-0.607343356	0.147088801	-4.129093135	3.64197E-05	0.004382851	0.00028888	-0.312073008	0.214384706	-1.455668239	0.145484354	0.801869091	0.340646292
244.079	71.325	0.580264952	0.140438256	4.13181544	3.59909E-05	0.004382851	0.00028888	0.385534537	0.199865964	1.928965435	0.053735153	0.695601427	0.175621481
570.665	67.778	-0.676847291	0.164217005	-4.121663834	3.76146E-05	0.004382851	0.00028888	0.044556926	0.193619629	0.230126079	0.817993801	0.984249726	0.858223004
210.888	69.976	-0.675737248	0.165840286	-4.074626651	4.60882E-05	0.004998045	0.000329428	0.098330966	0.200931395	0.489375821	0.624575651	0.959808266	0.706038576
666.523	68.093	-0.633693692	0.155567604	-4.073429644	4.63259E-05	0.004998045	0.000329428	-0.313923022	0.221679469	-1.4161123	0.156742628	0.803344788	0.354053935
614.6	67.891	-0.705695911	0.173953513	-4.056807459	4.97481E-05	0.005175575	0.00034113	-0.113131222	0.215913588	-0.523965273	0.600302694	0.956857133	0.698534044
664.526	68.434	-0.608669023	0.150614802	-4.041229779	5.31716E-05	0.005340998	0.000352033	-0.302231013	0.221325024	-1.365552831	0.17207936	0.803344788	0.359054501
326.987	76.127	-0.578605889	0.143541596	-4.030928353	5.5557E-05	0.005394583	0.000355565	-0.220392858	0.218666505	-1.00789491	0.313504915	0.884131049	0.470960924
160.133	55.358	0.561388624	0.139919918	4.012213792	6.0152E-05	0.005619776	0.000370407	0.838438412	0.238010129	3.522700554	0.000427174	0.210561227	0.019082789
722.486	66.165	-0.646349267	0.161342051	-4.006080646	6.17346E-05	0.005619776	0.000370407	-0.236033033	0.223290906	-1.057065141	0.290481859	0.868504775	0.454509667

205.068	73.03	0.694667136	0.175085429	3.967589657	7.26032E-05	0.005770274	0.000380327	0.232565091	0.232140833	1.001827587	0.316426871	0.884131049	0.470960924
312.782	71.919	-0.636334782	0.16029258	-3.969833056	7.1923E-05	0.005770274	0.000380327	-0.130291685	0.224407802	-0.580602294	0.561508526	0.942756082	0.676920227
334.836	67.586	-0.669306144	0.168032809	-3.98318727	6.79971E-05	0.005770274	0.000380327	0.052655174	0.198397729	0.265402098	0.79069972	0.98151707	0.838753294
360.15	108.476	0.615972619	0.155016206	3.97360143	7.0794E-05	0.005770274	0.000380327	0.309561593	0.220095323	1.406488739	0.159579046	0.803344788	0.356269499
608.565	68.642	-0.63552555	0.160270231	-3.965337452	7.32922E-05	0.005770274	0.000380327	-0.264970873	0.229371827	-1.155202351	0.248007572	0.858707894	0.43768636
130.123	27.204	0.547533794	0.138315252	3.958593031	7.53926E-05	0.005779436	0.000380931	0.363384698	0.214203037	1.696449789	0.089800731	0.748808418	0.253555006
390.798	67.798	-0.646666117	0.163630295	-3.951995055	7.75023E-05	0.005788829	0.00038155	0.103883541	0.201064384	0.516668041	0.605387909	0.956857133	0.700207702
604.571	68.42	-0.629535945	0.160237116	-3.928777318	8.53789E-05	0.006217715	0.000409818	-0.157051459	0.220819778	-0.711220077	0.476947869	0.926772739	0.635930492
346.862	70.229	-0.517631458	0.131957019	-3.922727722	8.75521E-05	0.006220468	0.00041	0.107451395	0.197158776	0.544999303	0.585753997	0.952790558	0.689967898
316.776	71.543	-0.62996002	0.160946007	-3.914107784	9.07391E-05	0.006293405	0.000414807	-0.141665483	0.222756397	-0.63596595	0.524798616	0.936389262	0.643661932
250.064	59.738	-0.644859715	0.165278758	-3.901649094	9.55396E-05	0.006372925	0.000420049	-0.296424179	0.222171397	-1.33421396	0.182133746	0.803344788	0.360512156
349.064	96.841	-0.529950789	0.135890819	-3.899827755	9.62611E-05	0.006372925	0.000420049	-0.124750033	0.185791147	-0.671453053	0.501931957	0.933437416	0.643661932
336.834	67.349	-0.599838506	0.154465172	-3.883325277	0.000103038	0.006669966	0.000439627	0.126471163	0.199600423	0.633621716	0.526327725	0.936389262	0.643661932
170.055	95.669	-0.598821401	0.154951075	-3.864583709	0.000111279	0.006948953	0.000464468	-0.332323589	0.268894713	-1.235887405	0.216500419	0.831255244	0.399693082
546.83	77.148	-0.578228178	0.149893081	-3.857604209	0.000114504	0.006948953	0.00046776	-0.214433477	0.203147058	-1.055557874	0.291170256	0.869035814	0.454509667
165.013	74.843	0.551030425	0.14334104	3.844191612	0.00012095	0.007086187	0.000476593	0.472913619	0.224206155	2.109280275	0.034920396	0.656278159	0.145368299
207.897	68.486	-0.659207582	0.171542819	-3.842816539	0.00012163	0.007086187	0.000476593	0.060456767	0.200964541	0.300833005	0.763541839	0.98151707	0.823595692
363.017	77.333	-0.467572907	0.122208993	-3.826010642	0.000130237	0.007438812	0.000500109	-0.164276724	0.196550625	-0.835798534	0.403268251	0.905383093	0.561068872
566.67	67.811	-0.608433862	0.159229571	-3.821110978	0.000132852	0.007442258	0.000500148	-0.002651636	0.198129685	-0.013383336	0.989321961	0.997558273	0.989321961
1037.653	51.282	-0.531779016	0.139637164	-3.808291446	0.00013993	0.007548462	0.000506917	-0.852344601	0.245970924	-3.465225028	0.000529788	0.210561227	0.019082789
486.656	68.489	-0.592159269	0.155381005	-3.811014529	0.000138398	0.007548462	0.000506917	-0.140344888	0.230564092	-0.608702279	0.542721796	0.941699578	0.659510031
198.085	100.113	-0.565260312	0.149078194	-3.791703515	0.000149617	0.007924285	0.000531973	-0.469732394	0.208963846	-2.247912274	0.024581784	0.585449383	0.115114698
106.999	75.195	-0.577958819	0.153208995	-3.772355662	0.000161714	0.008253142	0.00055352	-0.21894927	0.200228994	-1.093494332	0.274176816	0.861528104	0.449931186
315.161	42.821	-0.556255225	0.147612173	-3.768356049	0.000164326	0.008253142	0.00055352	-0.319164555	0.190908806	-1.671816833	0.094560446	0.756703339	0.263124721
329.03	87.116	-0.543991221	0.14410113	-3.775065605	0.000159966	0.008253142	0.00055352	-0.159019668	0.191914972	-0.828594383	0.407333967	0.905383093	0.562648358
254.824	72.613	-0.592897292	0.157959212	-3.753483471	0.000174394	0.00832803	0.00056752	-0.211482122	0.223637991	-0.945644884	0.344329743	0.889294418	0.500843262
314.779	72.2	-0.599431717	0.159695532	-3.753591028	0.000174319	0.00832803	0.00056752	-0.167339848	0.22601775	-0.740383656	0.459067233	0.921614646	0.616369992
146.6	96.634	-0.510468495	0.136389449	-3.742727149	0.000182034	0.008552655	0.000582508	-0.134461218	0.200213418	-0.671589446	0.501845099	0.933437416	0.643661932
1152.248	67.801	-0.475763009	0.128178047	-3.711735509	0.000205843	0.009369074	0.000647899	-0.269123545	0.168608226	-1.596147183	0.110455936	0.776526796	0.282767197
438.727	68.044	-0.590412888	0.159468405	-3.702381596	0.000213585	0.009571896	0.000661425	-0.07959083	0.208270256	-0.382151686	0.702348856	0.971874724	0.766198752
482.36	49.589	-0.603576621	0.16331571	-3.695765827	0.000219225	0.009675793	0.000668114	-0.422488751	0.229401344	-1.841701293	0.065518855	0.725410221	0.198741312
101.071	96.528	-0.533296367	0.14462782	-3.687370559	0.000226583	0.009706428	0.000669292	-0.13622643	0.196463611	-0.693392682	0.488063154	0.928727062	0.643661932
202.119	68.492	0.664597691	0.18004395	3.69130809	0.000223104	0.009700016	0.000669292	0.154401037	0.233345293	0.661684815	0.508173239	0.936311603	0.643661932
219.026	74.325	0.597766351	0.163182029	3.663187394	0.000249096	0.010007873	0.000687903	0.570315251	0.190090648	3.000227821	0.002697777	0.341679375	0.032373329
225.034	75.303	0.528311681	0.144023702	3.668227336	0.000244238	0.010007873	0.000687903	0.462543234	0.201398878	2.296652484	0.021638602	0.552923226	0.106528502
362.062	90.079	0.539382084	0.146780385	3.674755895	0.000238077	0.010007873	0.000687903	0.295170463	0.197840651	1.491960634	0.135709466	0.784861297	0.321681696

365.152	102.353	-0.449479403	0.12276016	-3.661443618	0.000250798	0.010007873	0.000687903	0.021583103	0.216194235	0.099832001	0.920477702	0.993360688	0.935716754
373.057	87.578	-0.482990743	0.131706632	-3.667171002	0.000245249	0.010007873	0.000687903	-0.472232234	0.183301044	-2.576265926	0.00998738	0.480537095	0.079899042
148.08	94.797	-0.537080957	0.146895456	-3.65621218	0.00025597	0.010076205	0.000692199	-0.111062961	0.196705272	-0.564616084	0.572334923	0.945664392	0.679474973
386.76	68.644	-0.581559425	0.159382811	-3.648821486	0.000263446	0.010097611	0.000702523	-0.010927678	0.206672085	-0.052874473	0.957831924	0.996806745	0.967914365
606.568	68.596	-0.57245028	0.157245256	-3.640493171	0.000272116	0.010294479	0.000715703	-0.242385085	0.227197417	-1.066847892	0.286040528	0.867562099	0.454509667
146.118	59.646	-0.533979418	0.147351657	-3.623844004	0.000290257	0.010839972	0.000753098	-0.470937545	0.232203778	-2.028121807	0.042547818	0.676759991	0.16338362
131.111	109.554	-0.507153504	0.140105495	-3.619797376	0.000294834	0.01087153	0.000754775	-0.278648605	0.212750634	-1.309742767	0.19028287	0.803344788	0.366995122
130.05	94.746	-0.548107179	0.152696164	-3.589528149	0.000331277	0.011565947	0.000829032	-0.079161989	0.197947131	-0.399914809	0.689219264	0.96832232	0.756171993
169.059	96.539	-0.51575639	0.143859037	-3.585151142	0.000336883	0.011565947	0.000829032	-0.222738812	0.219048804	-1.016845598	0.309226887	0.884131049	0.470960924
428.698	68.62	-0.570205598	0.159280774	-3.579877116	0.000343756	0.011565947	0.000829032	-0.148320859	0.225552742	-0.657588364	0.510802674	0.936389262	0.643661932
662.53	68.617	-0.556536172	0.155517527	-3.578607398	0.00034543	0.011565947	0.000829032	-0.310481792	0.224943967	-1.380262808	0.167505742	0.803344788	0.359054501
740.546	67.144	-0.536657426	0.149898071	-3.580148974	0.000343398	0.011565947	0.000829032	0.037059086	0.194218854	0.190810963	0.8486737	0.989638608	0.880785677
159.092	58.364	-0.507893373	0.143101179	-3.549190687	0.000386417	0.012525609	0.000915952	-0.287287134	0.21404005	-1.34221205	0.179527251	0.803344788	0.359054501
288.119	128.322	0.656982809	0.185838901	3.535227583	0.000407424	0.013042048	0.000953969	0.657932498	0.291507539	2.256999937	0.024008079	0.585449383	0.115114698
205.043	58.162	-0.430446881	0.122366129	-3.517696311	0.00043531	0.013783247	0.001006983	-0.105440529	0.197252459	-0.53454608	0.592963777	0.95567101	0.694201495
572.662	68.132	-0.501860089	0.143263235	-3.503062653	0.000459941	0.014253286	0.001038926	-0.351566313	0.194584852	-1.806750677	0.070801162	0.725972088	0.209135741
670.518	68.336	-0.489635966	0.139737486	-3.503970051	0.000458377	0.014253286	0.001038926	-0.392909705	0.196762978	-1.996868051	0.045839519	0.676759991	0.16433413
149.077	96.666	-0.448056909	0.128322067	-3.491659069	0.000480031	0.014542171	0.001059378	-0.129404037	0.194698918	-0.664636654	0.506282919	0.936173443	0.643661932
307.019	63.703	-0.494615683	0.141608154	-3.492847479	0.000477899	0.014542171	0.001059378	-0.318868577	0.20423017	-1.561319649	0.11844835	0.78238464	0.299237936
173.092	90.691	0.490073188	0.140559702	3.486583862	0.000489232	0.014542171	0.001067415	0.20370843	0.208499348	0.977021902	0.328558309	0.889294418	0.485255349
199.088	100.117	-0.509279347	0.146810827	-3.468949514	0.000522498	0.015220356	0.001114662	-0.4866336	0.2034236	-2.392218015	0.016746891	0.524555853	0.100464689
249.108	93.074	-0.553590233	0.159509314	-3.470582499	0.000519331	0.015220356	0.001114662	0.034266434	0.210191697	0.163024681	0.870499002	0.989638608	0.898579615
149.081	97.299	-0.500629579	0.145034592	-3.45179431	0.000556872	0.01594579	0.001174939	-0.125993506	0.198780441	-0.633832512	0.526190133	0.936389262	0.643661932
130.077	96.602	-0.518768111	0.150929903	-3.437146001	0.000587879	0.01615557	0.001200773	-0.112550945	0.195142761	-0.57676208	0.564100189	0.942756082	0.676920227
206.094	58.321	-0.516533741	0.150232903	-3.438219788	0.000585552	0.01615557	0.001200773	-0.219118354	0.21574528	-1.015634519	0.309803461	0.884131049	0.470960924
265.035	59.429	-0.502023204	0.145854454	-3.441946343	0.000577545	0.01615557	0.001200773	-0.174629098	0.201835333	-0.865205786	0.38692585	0.90173972	0.546248259
223.985	85.258	-0.472233778	0.139289563	-3.390302676	0.000698155	0.018116278	0.001367956	-0.151488528	0.203659831	-0.743831159	0.456978627	0.920646556	0.616369992
238.107	39.303	0.454051206	0.134006233	3.388284236	0.000703313	0.018116278	0.001367956	0.218956478	0.185806617	1.178410551	0.238632973	0.850841923	0.428201223
343.123	109.031	0.48599789	0.143136124	3.395354565	0.000685398	0.018116278	0.001367956	0.327812967	0.242313026	1.352849132	0.176103833	0.803344788	0.359054501
430.696	70.699	-0.53223931	0.15685102	-3.393279236	0.000690612	0.018116278	0.001367956	-0.246116878	0.229786481	-1.071067705	0.284138988	0.865127696	0.454509667
476.306	44.819	0.478375045	0.14121813	3.387490291	0.000705352	0.018116278	0.001367956	0.365555744	0.224993646	1.624738078	0.10421838	0.776526796	0.27791568
331.092	96.557	-0.430135193	0.128405912	-3.349808322	0.000808675	0.020133934	0.001552656	-0.357760257	0.201270612	-1.777508665	0.075484586	0.733212768	0.219591524
104.992	76.36	0.462617386	0.138785697	3.333321768	0.000858156	0.021006802	0.001615353	0.449770799	0.192669662	2.334414223	0.019574043	0.532889594	0.100464689
214.18	26.723	-0.4258496	0.127673204	-3.335465753	0.000851566	0.021006802	0.001615353	-0.397269863	0.169389696	-2.34530123	0.019011713	0.532889594	0.100464689
138.013	74.729	-0.453186721	0.136418989	-3.322020808	0.00089368	0.021338448	0.001665889	-0.166887794	0.191457177	-0.871671656	0.383387527	0.90173972	0.546248259

121.063	90.503	-0.449553036	0.135964204	-3.306407295	0.000945006	0.021959031	0.001720436	-0.093274623	0.208264624	-0.447865899	0.654249981	0.963445601	0.73032556
226.038	75.461	0.559032515	0.169148417	3.30498224	0.000949824	0.021959031	0.001720436	0.816056884	0.326798868	2.497122736	0.012520561	0.483682634	0.089691508
483.364	49.807	-0.437196936	0.132218204	-3.306631929	0.000944249	0.021959031	0.001720436	-0.106125853	0.216997495	-0.489064879	0.624795765	0.959808266	0.706038576
130.041	96.347	-0.41936054	0.127084361	-3.299859537	0.000967332	0.022187709	0.001735774	-0.112238173	0.199290846	-0.563187802	0.573307008	0.945664392	0.679474973
147.555	96.563	-0.461966279	0.140234144	-3.294249634	0.000986848	0.022458505	0.001754397	-0.252083179	0.192841436	-1.307204426	0.191143293	0.803344788	0.366995122
220.067	99.688	-0.471525492	0.143489709	-3.286127602	0.00101575	0.022915073	0.001789211	-0.499421104	0.198980517	-2.509899516	0.012076552	0.483682634	0.089691508
151.048	94.49	-0.483635255	0.147357138	-3.282061945	0.00103051	0.022915073	0.001798708	-0.064132058	0.218000052	-0.29418368	0.76861755	0.98151707	0.824438937
147.076	98.78	-0.478539606	0.146781526	-3.260216865	0.001113271	0.024201173	0.001925657	-0.098929786	0.198642694	-0.498028816	0.618463732	0.958671909	0.706038576
188.071	59.806	-0.502678212	0.154471555	-3.254179791	0.001137203	0.02453831	0.001949491	-0.256587585	0.217093107	-1.181924148	0.2372358	0.850021998	0.428201223
98.058	59.679	0.528619509	0.162859072	3.245870819	0.00117092	0.024896998	0.001989527	0.269034998	0.283084265	0.950370724	0.341923914	0.889294418	0.500843262
556.859	81.955	-0.48027595	0.149090505	-3.221371804	0.001275785	0.026545446	0.002148691	-0.264180724	0.193965791	-1.361996481	0.173198996	0.803344788	0.359054501
199.119	92.991	-0.433603581	0.135165293	-3.207950597	0.001336845	0.027232366	0.002231949	-0.054360418	0.21598243	-0.251689077	0.801281401	0.98151707	0.845307851
314.158	42.873	-0.519039014	0.162240114	-3.199202718	0.001378082	0.027877457	0.002280964	-0.421178571	0.174540507	-2.413070624	0.015818754	0.520764665	0.100464689
117.077	78.853	0.440348603	0.137877916	3.193757321	0.001404341	0.028019492	0.00228503	0.525745598	0.212015798	2.479747283	0.013147553	0.497258147	0.089691508
682.586	67.347	-0.472306679	0.147809359	-3.195377347	0.001396481	0.028019492	0.00228503	-0.094326687	0.202385785	-0.466073677	0.641162764	0.959808266	0.719902051
212.101	91.485	0.514878395	0.16151886	3.187729257	0.001433947	0.028223571	0.002294316	0.283087676	0.245298551	1.154053602	0.248478194	0.858707894	0.43768636
221.07	102.078	-0.442835194	0.138856207	-3.189163842	0.00142685	0.028223571	0.002294316	-0.373353511	0.182120418	-2.050036543	0.040360865	0.66801818	0.16144346
374.734	70.26	-0.502068918	0.157742043	-3.182847828	0.001458342	0.028321009	0.002314064	-0.238137059	0.216618464	-1.099338691	0.271620362	0.861528104	0.44957853
161.137	55	0.453630155	0.142633166	3.180397443	0.001470732	0.028372463	0.002314594	0.544536936	0.270773428	2.011042739	0.044320943	0.676759991	0.16433413
164.029	86.645	-0.461202365	0.145209064	-3.17612656	0.001492558	0.028604092	0.002329847	-0.298393544	0.219763829	-1.35779189	0.174529729	0.803344788	0.359054501
289.975	84.825	-0.443757975	0.140337824	-3.162069646	0.001566521	0.02982533	0.002425581	-0.200887834	0.199710771	-1.00589384	0.31446664	0.884131049	0.470960924
131.053	97.331	-0.470782819	0.149137894	-3.15669484	0.001595682	0.029988535	0.002450968	-0.080108868	0.198770688	-0.403021534	0.686932383	0.967957852	0.756171993
189.074	58.358	-0.478331436	0.152053823	-3.145803418	0.001656313	0.030928459	0.002523905	-0.228042292	0.215555295	-1.057929435	0.290087614	0.868504775	0.454509667
301.118	42.143	-0.429122753	0.137202247	-3.127665626	0.001762005	0.032281264	0.002663819	-0.160491508	0.196978625	-0.814766109	0.415206204	0.912024751	0.569425651
151.144	58.911	0.539402464	0.173096041	3.116203359	0.001831959	0.032941335	0.002726637	0.373275007	0.23120166	1.614499687	0.106419085	0.776526796	0.279896771
365.105	100.434	0.423012177	0.135722463	3.116744041	0.001828603	0.032941335	0.002726637	0.262064821	0.202871475	1.29177757	0.196434185	0.809229963	0.373419441
298.101	114.873	-0.420021261	0.135172042	-3.107308697	0.001887991	0.033534871	0.002767132	-0.184455568	0.181245216	-1.017712756	0.308814483	0.884131049	0.470960924
480.345	47	-0.509633074	0.163921809	-3.109001036	0.001877211	0.033534871	0.002767132	-0.248490234	0.219847782	-1.130283108	0.25835695	0.861528104	0.446887697
247.14	110.194	0.438780713	0.141425262	3.102562479	0.00191853	0.033771011	0.002778186	0.219102405	0.197591512	1.10886547	0.267488222	0.861528104	0.44957853
369.894	67.014	-0.383573504	0.123667681	-3.101647096	0.001924472	0.033771011	0.002778186	-0.430105733	0.18420953	-2.334872319	0.019550092	0.532889594	0.100464689
276.119	116.59	-0.432095643	0.13977951	-3.091265979	0.00199305	0.034765001	0.002855714	-0.317297169	0.211123682	-1.502897095	0.132865582	0.78270212	0.318877398
281.008	53.407	-0.423278313	0.137862765	-3.070287422	0.002138529	0.03613612	0.003041463	-0.310391599	0.193131978	-1.607147624	0.108021988	0.776526796	0.280273267
150.084	96.708	-0.378619411	0.123418579	-3.06776674	0.002156649	0.03613612	0.003044681	-0.096267822	0.197035516	-0.488581063	0.625138322	0.959808266	0.706038576
252.108	109.021	0.471293009	0.153900557	3.062321646	0.002196273	0.036558535	0.003077989	0.284559285	0.217383519	1.309019587	0.190527716	0.803344788	0.366995122
120.003	86.774	-0.475580129	0.156064147	-3.047337499	0.002308783	0.037997093	0.00321222	-0.138727062	0.21520066	-0.644640504	0.51916018	0.936389262	0.643661932
246.955	138.586	-0.418468574	0.13821472	-3.027670094	0.00246447	0.039662986	0.003379844	-0.206905257	0.194571433	-1.0633897	0.287605262	0.867760313	0.454509667

504.871	71.618	0.451681126	0.149183114	3.027696054	0.002464258	0.039662986	0.003379844	0.358316074	0.21919046	1.634724773	0.102106757	0.774445305	0.27611968
388.254	41.378	0.406453123	0.13452069	3.021491512	0.002515327	0.040259051	0.003425126	0.394860059	0.198859243	1.985625876	0.047074881	0.678857072	0.16433413
159.076	102.224	-0.462553526	0.15348366	-3.01369883	0.002580839	0.040690232	0.003445563	-0.708421433	0.215384873	-3.289095572	0.001005099	0.225219465	0.021442109
189.123	85.138	0.39613219	0.131460938	3.013307185	0.002584172	0.040690232	0.003445563	0.398920572	0.20055967	1.989036844	0.046697136	0.676759991	0.16433413
548.827	78.227	-0.433108087	0.143596507	-3.016146397	0.002560096	0.040690232	0.003445563	-0.228054143	0.206254874	-1.105690931	0.268860317	0.861528104	0.44957853
1150.25	67.735	-0.353789759	0.118433948	-2.987232666	0.002815154	0.043014142	0.003727652	-0.12404505	0.179645491	-0.690499099	0.489880376	0.928727062	0.643661932
412.853	73.358	-0.411413244	0.137823597	-2.985071152	0.002835124	0.043014142	0.003728382	-0.063078252	0.19941843	-0.316311045	0.751766428	0.981468845	0.815475448
1034.331	67.999	-0.438966272	0.147256031	-2.980973123	0.00287334	0.043263135	0.003751013	-0.319350625	0.210222529	-1.519107521	0.128735434	0.78270212	0.318877398
113.071	101.512	-0.445015677	0.149444599	-2.977796994	0.002903282	0.043263135	0.003751013	-0.676502232	0.211400137	-3.200103093	0.001373784	0.25011462	0.023978782
324.99	75.983	-0.451200508	0.151562693	-2.976989256	0.002910942	0.043263135	0.003751013	-0.269585948	0.199067676	-1.354242707	0.175658958	0.803344788	0.359054501
1128.196	68.006	-0.372969528	0.125711592	-2.966866626	0.003008514	0.044261628	0.003850898	-0.26105674	0.191810158	-1.361016242	0.173508557	0.803344788	0.359054501
127.03	48.917	-0.372437366	0.125740496	-2.961952412	0.00305695	0.044748222	0.003886983	-0.19964996	0.178709236	-1.117177627	0.263918385	0.861528104	0.44957853
214.023	130.222	-0.372518398	0.126066996	-2.954924037	0.00312746	0.045551461	0.003950476	-0.269261935	0.18933657	-1.422133798	0.154987413	0.803344788	0.354053935
520.12	55.829	-0.381107259	0.12999893	-2.93161843	0.003372008	0.047682808	0.004231539	-0.398949674	0.195871874	-2.036788976	0.041671193	0.672872087	0.163283044
1162.277	67.73	-0.439504116	0.150356751	-2.923075384	0.003465926	0.048539626	0.004321154	-0.531199533	0.212962178	-2.494337433	0.012619252	0.483682634	0.089691508
160.08	100.482	-0.435627945	0.149520403	-2.91350168	0.003573999	0.049108771	0.004427147	-0.733712841	0.218549771	-3.357188792	0.000787393	0.210561227	0.019082789
542.153	102.319	-0.401241638	0.138207397	-2.903184963	0.003693884	0.050047834	0.004546319	-0.41384694	0.187959433	-2.201788614	0.027680244	0.585449383	0.120786518
358.057	88.539	-0.368500709	0.129109439	-2.854173253	0.004314899	0.055863563	0.005276819	-0.248147494	0.184691451	-1.343578666	0.179084672	0.803344788	0.359054501
177.106	100.743	-0.427763354	0.150156492	-2.848783617	0.004388672	0.056318065	0.005299528	-0.738222363	0.217153338	-3.399544167	0.000674983	0.210561227	0.019082789
318.97	76.169	0.403713966	0.141662842	2.84982257	0.004374362	0.056318065	0.005299528	0.271481939	0.225249587	1.20524944	0.228107022	0.845450259	0.417109982
372.1	37.886	0.364645525	0.128149183	2.845476791	0.004434499	0.056409152	0.005321399	0.179569065	0.190933289	0.940480657	0.346971067	0.889294418	0.500890563
1134.187	68.175	-0.384511595	0.137535905	-2.795717931	0.005178457	0.063946902	0.006137431	-0.301361696	0.20045243	-1.503407541	0.132733983	0.78270212	0.318877398
353.9	79.616	-0.410057572	0.146639428	-2.796366428	0.005168077	0.063946902	0.006137431	-0.270408451	0.199244125	-1.357171518	0.174726718	0.803344788	0.359054501
176.103	102.372	-0.429786583	0.154297252	-2.785445474	0.005345423	0.065425282	0.006296449	-0.666865231	0.215712721	-3.091450649	0.001991811	0.276292624	0.027316264
176.06	99.313	-0.374338251	0.134825339	-2.776468091	0.005495303	0.066978315	0.006433525	-0.784036254	0.233727455	-3.354489328	0.000795116	0.210561227	0.019082789
271.04	74.974	0.419208279	0.152080714	2.756485473	0.005842621	0.070039318	0.006798686	0.437902291	0.227233675	1.927101213	0.053967018	0.695601427	0.175621481
509.379	48.096	-0.42919469	0.156836773	-2.736569255	0.006208353	0.072339724	0.007180745	-0.185762004	0.220481019	-0.842530592	0.399491024	0.903507261	0.559870632
163.06	105.755	0.38254227	0.140898427	2.71502159	0.006627145	0.075129695	0.007575237	0.399167711	0.217323067	1.836748012	0.066247104	0.725410221	0.198741312
496.84	72.752	0.394173208	0.14518552	2.71496227	0.006628332	0.075129695	0.007575237	0.595114702	0.255574571	2.328536444	0.019883636	0.53630586	0.100464689
568.426	36.345	-0.401786146	0.152032736	-2.642760744	0.008223312	0.089382493	0.009342461	-0.433540431	0.220421673	-1.966868434	0.04919838	0.681347224	0.168680162
1154.245	67.712	-0.305188536	0.115881808	-2.633619038	0.008448022	0.091483595	0.009541295	-0.185804483	0.168218304	-1.10454379	0.269357319	0.861528104	0.44957853
1041.685	49.43	-0.417665253	0.159399381	-2.620243873	0.008786691	0.093075017	0.009865758	-0.409605489	0.21103061	-1.940976658	0.052261109	0.695601427	0.175621481
207.024	79.355	0.443203085	0.169556867	2.613890512	0.008951772	0.0938004	0.009992675	0.705065985	0.299009037	2.358008946	0.01837325	0.532889594	0.100464689
1047.732	48.709	-0.37584436	0.144778013	-2.596004414	0.009431485	0.097288601	0.010467313	-0.316929278	0.209497363	-1.512807958	0.130328447	0.78270212	0.318877398
85.052	96.569	-0.309243342	0.121803646	-2.538867693	0.011121187	0.110190538	0.012271655	-0.347608659	0.196652731	-1.767626911	0.077123296	0.733212768	0.221010042
159.084	121.116	0.388444268	0.153769501	2.526146381	0.011532142	0.113346558	0.012652407	0.544586332	0.229802601	2.369800554	0.017797683	0.532889594	0.100464689

551.425	36.279	-0.361378502	0.144314367	-2.504106212	0.012276118	0.119599767	0.013392128	-0.433984839	0.195567463	-2.219105528	0.026479546	0.585449383	0.12064073
351.088	56.365	0.309394608	0.123669605	2.501783743	0.012356938	0.119985867	0.013404136	0.376542825	0.179166309	2.101638573	0.035584948	0.656425275	0.145368299
90.055	84.601	0.35456643	0.142028918	2.496438298	0.012544749	0.121002828	0.013531415	0.587882075	0.21349705	2.753584072	0.005894663	0.475910817	0.056588763
193.028	89.085	0.357066052	0.144259462	2.475165567	0.013317442	0.124738615	0.014284631	0.478649305	0.220701962	2.168758718	0.030101007	0.618365982	0.128430963
148.06	89.479	0.343866939	0.142715233	2.409462062	0.015976057	0.14275538	0.017041127	0.556764236	0.2069656	2.690129353	0.007142433	0.475910817	0.065302243
174.05	76.594	-0.323061161	0.136750705	-2.362409478	0.018156575	0.157075197	0.019260013	-0.427642271	0.182037715	-2.349195987	0.018814001	0.532889594	0.100464689
238.092	127.057	0.361867079	0.153651188	2.355120606	0.018516695	0.158045884	0.019534096	0.454196076	0.242241251	1.874974118	0.060796284	0.714657298	0.188272364
277.953	141.319	-0.283518092	0.130785168	-2.167815325	0.03017274	0.218078223	0.031656646	-0.368503627	0.196182219	-1.878374242	0.060329987	0.714657298	0.188272364
216.063	122.209	0.306269203	0.143893499	2.128443638	0.033300318	0.231147828	0.034748158	0.858951071	0.290793273	2.953820299	0.003138667	0.37891493	0.035448476
335.84	67.832	-0.295196981	0.139975686	-2.108916119	0.034951823	0.238441825	0.036274324	-0.498924628	0.225630083	-2.211250476	0.027018497	0.585449383	0.12064073
1038.657	51.615	-0.258039025	0.12545088	-2.05689291	0.039696528	0.260230308	0.040977061	-0.704404625	0.201409401	-3.497377084	0.000469857	0.210561227	0.019082789
245.049	81.304	0.271253285	0.138115304	1.96396256	0.049534434	0.296900836	0.050858884	0.704159659	0.227700305	3.09248448	0.001984886	0.276292624	0.027316264
177.1	100.826	-0.242527491	0.124127488	-1.953858054	0.050718006	0.301513373	0.051797113	-0.588574381	0.187186685	-3.144317559	0.001664747	0.276292624	0.026635949
101.977	134.09	-0.275242918	0.142303816	-1.934192113	0.053089512	0.309738621	0.053724516	-0.410729554	0.205191377	-2.001690126	0.045318069	0.676759991	0.16433413
272.185	35.432	0.257891491	0.133375188	1.933579212	0.053164885	0.309738621	0.053724516	0.528360367	0.201403411	2.623393341	0.00870587	0.475910817	0.075978501
585.195	102.316	-0.232065782	0.141100469	-1.64468469	0.100034852	0.430421869	0.100558594	-0.564488728	0.203828733	-2.769426669	0.005615504	0.475910817	0.056588763
232.027	52.237	0.200306963	0.131736721	1.520509706	0.128382921	0.485687597	0.128382921	0.653261909	0.182761449	3.574396634	0.000351036	0.210561227	0.019082789

Supplementary Table 8. Type 2 diabetes mellitus associated metabolites that overlapped with main analysis stratified by sex among Parkinson's disease patients.													
C18													
		**Male (n = 524)**						**Female (n = 307)**					
mz	rt	beta	se	zvalue	pvalue	Full MWAS FDR p value	Replication FDR p value	beta	se	zvalue	pvalue	Full MWAS FDR p value	Replication FDR p value
199.038	31.744	-1.09467	0.157416	-6.95396	3.55E-12	3.95E-09	2.36E-10	-0.3955	0.186811	-2.11711	0.034251	0.430406	0.074162
209.067	36.808	-1.10304	0.156814	-7.0341	2.01E-12	3.95E-09	2.36E-10	-0.48896	0.191969	-2.54706	0.010863	0.335132	0.032813
200.041	32.191	-0.77422	0.123335	-6.27736	3.44E-10	2.55E-07	1.53E-08	-0.44014	0.166124	-2.64947	0.008062	0.291649	0.026806
223.082	38.976	-0.86812	0.144842	-5.99358	2.05E-09	1.14E-06	6.83E-08	-0.66511	0.194297	-3.42317	0.000619	0.098238	0.00686
113.024	36.458	0.707492	0.142814	4.953945	7.27E-07	3.23E-04	1.93E-05	0.543167	0.17332	3.1339	0.001725	0.165151	0.011742
143.035	36.297	0.684564	0.143982	4.754527	1.99E-06	7.37E-04	4.41E-05	0.606738	0.179704	3.376324	0.000735	0.108821	0.007516
269.088	35.076	0.683174	0.146904	4.65048	3.31E-06	0.001051	6.29E-05	0.839156	0.212651	3.946164	7.94E-05	0.087819	0.005782
225.062	37.895	0.648624	0.142231	4.560344	5.11E-06	0.001418	8.49E-05	0.642639	0.182198	3.527151	0.00042	0.087819	0.005782
496.807	33.723	-0.58073	0.132412	-4.38577	1.16E-05	0.002853	0.000171	-0.01402	0.192093	-0.07301	0.941799	0.984433	0.942372
215.091	31.459	0.60096	0.139859	4.296889	1.73E-05	0.003804	0.000228	0.562114	0.179759	3.127052	0.001766	0.165151	0.011742
218.033	30.739	0.590318	0.137979	4.278323	1.88E-05	0.003804	0.000228	0.553062	0.178546	3.097582	0.001951	0.172215	0.012357
179.056	36.803	0.573653	0.134994	4.249483	2.14E-05	3.97E-03	0.000237	0.527345	0.180505	2.921499	0.003484	0.203694	0.015976
217.03	30.51	0.57985	0.138281	4.193282	2.75E-05	0.004699	0.000281	0.588704	0.179162	3.28588	0.001017	0.128819	0.009014
215.033	30.913	0.577797	0.138446	4.173454	3.00E-05	0.004762	0.000285	0.599179	0.18058	3.318073	0.000906	0.125877	0.008611
145.062	30.621	-0.60507	0.146098	-4.14153	3.45E-05	5.11E-03	0.000306	-0.28279	0.219633	-1.28755	0.197903	0.718562	0.257595
195.051	31.406	0.570461	0.139145	4.099752	4.14E-05	0.005744	0.000344	0.689674	0.198822	3.468795	0.000523	0.096805	0.006321
216.035	31.278	0.556516	0.136226	4.085229	4.40E-05	0.005755	0.000344	0.572868	0.179586	3.189936	0.001423	0.150571	0.010515
425.291	227.677	0.67719	0.167638	4.039607	5.35E-05	0.006609	0.000396	0.690147	0.229868	3.002367	0.002679	0.186015	0.014252
494.81	33.188	-0.49158	0.122301	-4.01947	5.83E-05	0.006821	0.000408	-0.29998	0.175788	-1.70649	0.087917	0.56404	0.139201
158.061	87.22	-0.62346	0.156727	-3.97799	6.95E-05	7.02E-03	0.00044	-0.26326	0.217508	-1.21035	0.226145	0.736794	0.281096
174.088	30.043	-0.56912	0.142967	-3.98077	6.87E-05	7.02E-03	0.00044	-0.75334	0.214136	-3.51804	0.000435	0.087819	0.005782
280.622	30.778	-0.49461	0.125374	-3.94507	7.98E-05	0.007386	0.000461	-0.53852	0.17673	-3.04716	0.00231	0.172215	0.013125
414.204	35.694	-0.60782	0.153733	-3.95374	7.69E-05	0.007386	0.000461	-0.11925	0.212327	-0.56161	0.57438	0.921496	0.626168
213.049	35.964	-0.56454	0.143899	-3.92316	8.74E-05	0.007768	0.000484	-0.09244	0.214069	-0.43182	0.665873	0.941343	0.702866
415.322	215.543	-0.5702	0.145739	-3.91245	9.14E-05	0.007808	0.000486	-0.07302	0.197498	-0.36972	0.711594	0.952662	0.739391
253.093	34.817	-0.47225	0.121984	-3.87144	0.000108	0.008904	0.000553	-0.30972	0.18082	-1.71288	0.086735	0.56404	0.139201
279.614	29.271	-0.51244	0.132947	-3.85448	0.000116	0.009203	0.000571	-0.22677	0.185212	-1.22439	0.220806	0.733527	0.28017
180.06	36.11	0.520446	0.137774	3.77753	1.58E-04	1.21E-02	0.000752	0.535115	0.183031	2.923635	0.00346	0.203694	0.015976
226.065	36.217	0.54724	0.145313	3.765949	0.000166	0.012289	0.000761	0.604188	0.19718	3.064139	0.002183	0.172215	0.013125
239.077	35.736	0.524627	0.139776	3.753336	0.000174	0.012507	0.000774	0.379565	0.18801	2.018856	0.043502	0.452431	0.085085
118.051	31.331	-0.55818	0.150552	-3.70753	2.09E-04	1.40E-02	8.64E-04	0.015207	0.210359	0.072289	0.942372	0.984433	0.942372
162.056	40.608	-0.61753	0.167183	-3.69375	2.21E-04	1.40E-02	0.000864	0.025164	0.196863	0.127824	0.898288	0.978678	0.912002
177.04	33.332	0.574441	0.154694	3.713403	2.04E-04	1.40E-02	0.000864	0.533108	0.204248	2.610105	0.009051	0.304319	0.028192
311.14	35.236	-0.48817	0.132077	-3.69608	0.000219	0.014029	0.000864	-0.36993	0.153823	-2.40491	0.016177	0.36678	0.042186
103.04	32.211	0.498761	0.135538	3.679876	2.33E-04	1.44E-02	8.87E-04	0.194262	0.199058	0.975904	0.329112	0.791927	0.39082
261.041	34.747	-0.4621	0.125928	-3.66952	0.000243	0.014594	0.000898	-0.4639	0.16958	-2.73557	0.006227	0.267689	0.023664
149.046	31.935	0.518055	0.142407	3.637857	2.75E-04	1.61E-02	0.000988	0.357279	0.212103	1.68446	0.092093	0.575564	0.141205
161.987	32.772	0.478539	0.133873	3.574581	3.51E-04	2.00E-02	0.001228	0.563601	0.195668	2.88039	0.003972	0.206365	0.017134
203.021	32.471	-0.45567	0.12901	-3.53207	0.000412	0.022904	0.001406	-0.2427	0.214963	-1.12905	0.258878	0.759534	0.315878

127.051	32.393	-0.54891	0.157281	-3.49001	4.83E-04	2.39E-02	0.00146	-0.12181	0.206499	-0.5899	0.555255	0.907856	0.610322
160.062	29.915	0.47274	0.134598	3.512224	4.44E-04	2.39E-02	0.00146	0.523899	0.200903	2.607719	0.009115	0.304319	0.028192
226.018	34.225	-0.50177	0.143282	-3.50201	0.000462	0.023855	0.00146	-0.06943	0.206217	-0.3367	0.73634	0.953666	0.759173
229.144	107.471	-0.46262	0.132559	-3.48995	0.000483	0.023855	0.00146	-0.29564	0.188115	-1.57159	0.116045	0.613816	0.169604
322.932	37.944	-0.51179	0.146213	-3.50034	0.000465	0.023855	0.00146	-0.16379	0.191742	-0.85422	0.392986	0.830887	0.454496
452.705	33.294	-0.49252	0.141505	-3.48061	0.0005	0.024165	0.001479	-0.07949	0.19254	-0.41285	0.679717	0.943368	0.71183
254.623	31.407	-0.48824	0.144205	-3.38572	0.00071	0.032285	0.002053	-0.13342	0.199397	-0.66909	0.503435	0.895093	0.562663
415.306	235.89	0.561672	0.168332	3.336696	0.000848	0.036227	0.002399	0.612957	0.22242	2.755851	0.005854	0.267689	0.023593
151.007	30.177	0.510411	0.153796	3.31875	9.04E-04	3.79E-02	0.002505	0.13896	0.223073	0.622934	0.533328	0.90424	0.591105
149.061	32.211	-0.47904	0.144801	-3.30824	9.39E-04	3.86E-02	0.002548	-0.18761	0.202903	-0.92463	0.355161	0.810552	0.418021
146.065	30.227	-0.43584	0.132152	-3.29804	9.74E-04	3.93E-02	0.002585	-0.03569	0.2151	-0.16593	0.868216	0.978249	0.888252
416.953	38.484	-0.4005	0.121623	-3.29299	0.000991	0.039333	0.002585	-0.15711	0.193132	-0.81348	0.415942	0.843165	0.472823
220.049	30.642	-0.49637	0.152171	-3.26191	0.001107	0.041751	0.00273	-0.10339	0.212715	-0.48605	0.626931	0.937791	0.677902
566.582	32.607	-0.53041	0.162633	-3.26141	0.001109	0.041751	0.00273	-0.42911	0.197547	-2.17217	0.029843	0.426506	0.069112
94.908	31.854	-0.52167	0.159768	-3.26515	0.001094	0.041751	0.00273	-0.33184	0.215234	-1.54178	0.123127	0.624633	0.176085
324.921	34.963	-0.43198	0.133367	-3.23907	0.001199	0.042296	0.002848	-0.40298	0.195262	-2.06377	0.03904	0.446706	0.080225
581.24	42.898	-0.4082	0.125845	-3.24371	0.00118	0.042284	0.002848	-0.28244	0.197371	-1.43102	0.152425	0.676443	0.204773
221.053	30.668	-0.49034	0.152376	-3.21795	0.001291	0.044826	0.003008	-0.09493	0.212026	-0.44772	0.654354	0.941343	0.696232
402.293	232.497	0.522839	0.162733	3.212869	0.001314	0.044904	0.003008	0.57513	0.234257	2.455126	0.014084	0.363202	0.039853
503.631	32.36	-0.44767	0.139524	-3.20852	0.001334	0.044904	0.003008	-0.40506	0.173586	-2.33347	0.019624	0.414365	0.050191
223.05	30.675	-0.48525	0.152125	-3.18983	0.001424	0.046309	0.003135	-0.09836	0.212345	-0.46322	0.643208	0.941343	0.689892
431.244	82.667	0.475691	0.149264	3.186908	0.001438	0.046309	0.003135	0.165353	0.215365	0.767778	0.442619	0.859643	0.498884
224.952	56.909	-0.42119	0.133581	-3.15307	0.001616	0.051285	0.003466	-0.27795	0.186042	-1.49404	0.135164	0.649665	0.187258
498.638	32.269	-0.51022	0.162825	-3.13357	0.001727	0.053295	0.003646	-0.28906	0.198997	-1.45256	0.146345	0.666004	0.200658
268.96	35.981	-0.41223	0.132608	-3.10863	0.00188	0.056438	0.003906	-0.34533	0.201832	-1.71096	0.087088	0.56404	0.139201
424.247	181.74	0.50539	0.16357	3.089745	0.002003	0.057068	0.004099	0.188345	0.225349	0.835791	0.403272	0.834331	0.462373
96.922	31.526	-0.48253	0.157407	-3.06548	0.002173	0.061125	0.004379	-0.19518	0.215803	-0.90445	0.365759	0.822587	0.426718
189.067	63.388	-0.42308	0.138601	-3.05247	2.27E-03	6.17E-02	0.004436	-0.23791	0.195086	-1.21954	0.222641	0.733527	0.28017
401.291	235.525	0.486344	0.159183	3.055244	0.002249	0.061738	0.004436	0.620084	0.220204	2.815947	0.004863	0.236747	0.020213
416.309	235.341	0.519226	0.170334	3.048282	0.002302	0.061738	0.004436	0.595803	0.222286	2.680343	0.007355	0.281987	0.025741
195.976	28.201	-0.45006	0.148751	-3.02559	0.002481	0.064868	0.004715	-0.26886	0.220772	-1.21782	0.223293	0.733527	0.28017
94.942	31.882	-0.4747	0.157156	-3.02055	0.002523	0.065192	0.004726	-0.37426	0.204151	-1.83327	0.066763	0.513772	0.118393
359.121	49.326	0.471375	0.158426	2.975359	0.002926	0.071457	0.005406	0.217144	0.211201	1.028141	0.303884	0.778314	0.364113
369.154	47.826	-0.41158	0.140551	-2.92837	0.003407	0.078869	0.006208	-0.22709	0.19206	-1.18241	0.237044	0.754131	0.291915
144.97	29.085	-0.43207	0.148203	-2.91542	3.55E-03	8.14E-02	0.006384	-0.47198	0.240042	-1.96625	4.93E-02	4.59E-01	0.092294
254.982	28.027	0.428398	0.147532	2.903766	0.003687	0.083598	0.006538	0.392197	0.217249	1.805286	0.07103	0.519874	0.124302
501.634	32.333	-0.37957	0.132111	-2.87312	0.004064	0.089417	0.007113	-0.34499	0.171623	-2.01018	0.044412	0.452431	0.085607
229.012	27.859	0.463266	0.162123	2.857492	0.00427	0.091309	0.007281	0.265125	0.236216	1.122384	0.261699	0.761454	0.316418
286.06	27.608	0.541046	0.189143	2.86051	0.00423	0.091309	0.007281	0.448884	0.26671	1.683039	0.092367	0.575564	0.141205
256.934	34.638	-0.37823	0.132989	-2.84406	0.004454	0.092499	0.007499	-0.46741	0.215567	-2.16826	0.030139	0.426506	0.069112
415.216	36.104	-0.4173	0.147595	-2.8273	0.004694	0.094505	0.007752	-0.45693	0.240064	-1.90335	0.056995	0.48153	0.105282
442.676	32.194	-0.44847	0.158723	-2.82548	0.004721	0.094505	0.007752	-0.31551	0.19066	-1.65483	0.097958	0.575564	0.14805
217.048	30.99	0.501734	0.178648	2.808507	0.004977	0.095339	0.007975	0.327536	0.2363	1.386101	0.165716	0.692145	0.220402

446.67	32.191	-0.44876	0.159651	-2.81087	0.004941	0.095339	0.007975	-0.38739	0.189717	-2.04196	0.041156	0.448274	0.081697
163.077	35.967	-0.36699	0.131175	-2.79768	5.15E-03	9.78E-02	0.008149	-0.31826	0.184424	-1.72569	0.084403	0.556507	0.139201
295.984	32.846	-0.35922	0.128651	-2.79222	0.005235	0.098573	0.008191	-0.28803	0.182575	-1.57762	0.114653	0.613816	0.169431
373.26	213.573	0.459928	0.16612	2.768649	0.005629	0.101091	0.008605	0.533822	0.281509	1.896291	0.057922	0.487507	0.105528
508.623	32.501	-0.36913	0.133224	-2.77073	0.005593	0.101091	0.008605	-0.4343	0.201208	-2.15847	0.030891	0.426506	0.069636
266.963	36.467	-0.36651	0.132767	-2.76058	0.00577	0.101091	0.008646	-0.28126	0.218553	-1.28691	0.198126	0.718562	0.257595
312.143	35.633	-0.36158	0.131196	-2.75602	0.005851	0.101091	0.008646	-0.39709	0.178313	-2.22691	0.025954	0.426506	0.06276
387.275	216.137	0.462705	0.167883	2.756124	0.005849	0.101091	0.008646	0.354113	0.234235	1.511781	0.13059	0.639141	0.182825
423.275	218.074	0.539316	0.197239	2.734326	0.006251	0.103652	0.009136	0.308482	0.215466	1.431696	0.152231	0.676443	0.204773
193.123	84.259	-0.38317	0.142539	-2.68819	0.007184	0.110007	0.010189	-0.60081	0.202389	-2.96862	0.002991	0.196436	0.015242
194.039	27.536	0.385265	0.143549	2.683862	0.007278	0.110007	0.010189	0.315589	0.20852	1.513471	0.13016	0.638661	0.182825
225.054	30.806	-0.3885	0.144687	-2.68508	0.007251	0.110007	0.010189	-0.24188	0.188524	-1.283	0.199491	0.719591	0.257595
281.124	29.849	-0.37691	0.140281	-2.68679	0.007214	0.110007	0.010189	-0.52184	0.207707	-2.51238	0.011992	0.33971	0.034672
255.63	33.175	-0.31959	0.120443	-2.65348	0.007967	0.11607	0.011037	-0.27311	0.175388	-1.55719	0.119426	0.618568	0.172649
282.975	35.721	-0.32089	0.122606	-2.61727	0.008864	0.125034	0.012153	-0.4294	0.175454	-2.44738	0.01439	0.363202	0.039872
506.626	32.343	-0.41954	0.160601	-2.61233	0.008993	0.125034	0.012205	-0.38015	0.185074	-2.05402	0.039974	0.446706	0.080553
300.877	34.139	-0.33281	0.128161	-2.59678	0.00941	0.128278	0.012642	-0.40736	0.197555	-2.062	0.039208	0.446706	0.080225
114.056	36.877	0.350962	0.135589	2.588425	9.64E-03	1.30E-01	1.28E-02	0.477251	0.1964	2.429995	0.015099	0.363202	0.040983
505.628	32.397	-0.33894	0.131397	-2.57951	0.009894	0.131789	0.013029	-0.35457	0.171498	-2.06746	0.03869	0.445441	0.080225
444.673	32.115	-0.40355	0.157343	-2.56481	0.010323	0.133535	0.013461	-0.32672	0.187609	-1.74152	0.081593	0.554699	0.139201
424.278	219.234	0.419063	0.164141	2.553062	0.010678	0.134238	0.013788	0.476153	0.223378	2.131606	0.033039	0.426506	0.073237
327.091	28.264	0.326769	0.128636	2.54027	0.011077	0.138272	0.014165	0.495968	0.188136	2.636215	0.008384	0.295691	0.027196
156.878	31.938	-0.38321	0.153499	-2.49652	1.25E-02	1.49E-01	0.015886	-0.36154	0.211701	-1.70777	0.08768	0.56404	0.139201
564.083	212.912	-0.33002	0.134534	-2.45303	0.014166	0.162249	0.017774	-0.33278	0.191911	-1.73401	0.082916	0.554699	0.139201
270.091	34.705	0.372827	0.153627	2.426834	0.015231	0.168377	0.018932	1.1157	0.305335	3.654026	0.000258	0.087819	0.005782
216.971	30.784	0.350575	0.145781	2.404799	0.016181	0.174663	0.019927	0.712355	0.223143	3.192367	0.001411	0.150571	0.010515
562.588	32.428	-0.39939	0.166597	-2.39736	0.016513	0.174687	0.020149	-0.28247	0.172877	-1.63394	0.102271	0.580411	0.152832
440.679	32.243	-0.36957	0.155404	-2.3781	0.017402	0.179063	0.021041	-0.50703	0.226984	-2.23375	0.0255	0.426506	0.06276
260.023	28.879	0.310616	0.132282	2.348134	0.018868	0.188001	0.022607	0.670862	0.189947	3.531835	0.000413	0.087819	0.005782
439.306	227.232	0.411493	0.176181	2.335634	0.01951	0.190978	0.023168	0.825247	0.306726	2.690503	0.007134	0.281987	0.025645
406.925	35.575	-0.2889	0.124223	-2.32567	0.020036	0.193567	0.023582	-0.31143	0.166221	-1.87358	0.060988	0.501908	0.109613
302.874	34.257	-0.29342	0.126845	-2.31319	0.020712	0.196678	0.024164	-0.39581	0.182476	-2.16912	0.030074	0.426506	0.069112
397.259	210.47	0.36399	0.15894	2.290104	0.022015	0.206405	0.025461	0.470776	0.222764	2.113341	0.034572	0.430608	0.074162
89.024	31.295	0.298447	0.132287	2.256048	0.024068	0.220134	0.027595	0.769602	0.210248	3.660441	0.000252	0.087819	0.005782
188.057	31.438	0.314967	0.140022	2.249408	2.45E-02	2.23E-01	0.027835	0.343339	0.201873	1.700767	0.088987	0.566557	0.139238
556.597	32.309	-0.36432	0.16331	-2.23088	0.025689	0.229242	0.028955	-0.37975	0.192459	-1.97316	0.048478	0.45836	0.092108
89.04	33.444	0.294091	0.132323	2.222527	0.026248	0.23236	0.029336	0.814593	0.218604	3.726343	0.000194	0.087819	0.005782
390.709	32.22	-0.31125	0.141219	-2.20404	0.027522	0.238879	0.030503	-0.48237	0.199689	-2.41562	0.015708	0.363202	0.041784
90.028	31.729	0.291112	0.132633	2.194863	0.028173	0.240774	0.030967	0.746499	0.211193	3.534682	0.000408	0.087819	0.005782
89.009	34.171	0.284902	0.132192	2.155212	0.031145	0.257267	0.033953	0.793091	0.214932	3.689968	0.000224	0.087819	0.005782
412.278	210.722	0.332218	0.155177	2.140902	0.032282	0.261423	0.034906	0.352799	0.203895	1.730294	0.083578	0.556017	0.139201
374.263	214.626	0.334025	0.158196	2.111466	0.034732	0.267969	0.037253	0.537767	0.232649	2.311498	0.020805	0.41671	0.05221
276	31.551	0.262561	0.125753	2.087909	0.036806	0.275364	0.039162	0.577053	0.189841	3.039673	0.002368	0.172215	0.013125

356.098	28.295	0.273994	0.132696	2.06482	0.03894	0.278812	0.041103	0.546934	0.19924	2.745102	0.006049	0.267689	0.023663
445.988	31.336	-0.28888	0.140535	-2.05558	0.039823	0.280782	0.041705	-0.63314	0.214026	-2.95822	0.003094	0.196436	0.015242
216.909	24.258	0.304038	0.151888	2.001722	0.045315	0.297897	0.04672	0.644198	0.253674	2.539467	0.011102	0.335132	0.032813
219.034	30.853	0.265004	0.132333	2.002561	0.045224	0.297897	0.04672	0.541547	0.198881	2.722966	0.00647	0.269683	0.023903
326.087	28.546	0.243579	0.130669	1.864096	0.062308	0.347861	0.063746	0.638278	0.195597	3.263227	0.001102	0.128819	0.009156
418.95	37.272	-0.21783	0.129621	-1.6805	0.09286	0.426312	0.094278	-0.45531	0.171661	-2.65238	0.007993	0.291649	0.026806
148.98	33.213	0.194672	0.136499	1.426181	1.54E-01	5.26E-01	0.154981	0.875708	0.240797	3.636702	0.000276	0.087819	0.005782
403.296	242.279	0.206409	0.154671	1.334509	0.182037	0.563657	0.182037	0.859075	0.298428	2.87867	0.003994	0.206365	0.017134

Supplementary Table 10. Type 2 diabetes mellitus associated metabolites that overlapped with main analysis stratified by race among Parkinson's disease patients.

HILIC

		**European Ancestry (n = 637)**						**Non-European Ancestry (n = 194)**					
mz	rt	beta	se	zvalue	pvalue	Full MWAS FDR p value	Replication FDR p value	beta	se	zvalue	pvalue	Full MWAS FDR p value	Replication FDR p value
130.136	49.642	1.016874003	0.157094035	6.473027448	9.60585E-11	2.79818E-07	1.84432E-08	1.037739453	0.245545939	4.226253775	2.37614E-05	0.041576902	0.002740393
335.094	86.335	0.926787584	0.153735662	6.028448921	1.65541E-09	2.4111E-06	1.58919E-07	1.018318358	0.243339268	4.1847679	2.85458E-05	0.041576902	0.002740393
203.053	75.296	0.686227416	0.134226579	5.11245553	3.17998E-07	0.000295421	1.94717E-05	0.653559577	0.196539172	3.325340027	0.000883108	0.233862955	0.018839629
204.056	74.501	0.676360398	0.133502302	5.066282664	4.05659E-07	0.000295421	1.94717E-05	0.71960876	0.201837398	3.565289527	0.000363455	0.175204881	0.013472672
221.988	85.683	-0.748495706	0.151887873	-4.927949101	8.30973E-07	0.000484125	3.19093E-05	-0.355307501	0.208243965	-1.706207912	0.087969376	0.593182391	0.126045673
160.133	55.358	0.672944637	0.153252942	4.391071564	1.12793E-05	0.00547612	0.000360939	0.557560673	0.19921178	2.798833844	0.005128752	0.383058261	0.036129553
313.154	42.894	-0.598371214	0.138823984	-4.310287005	1.63043E-05	0.00678491	0.000447203	-0.45337246	0.216319674	-2.095844785	0.036095962	0.477943352	0.069956784
1037.653	51.282	-0.587121291	0.138341606	-4.243996509	2.19574E-05	0.007228244	0.000476424	-0.708312041	0.245901944	-2.880465402	0.003970885	0.368303077	0.0332627
160.08	100.482	-0.631201035	0.148861252	-4.240197009	2.23324E-05	0.007228244	0.000476424	-0.317751085	0.212840088	-1.492909953	0.135460759	0.664305038	0.171108328
159.076	102.224	-0.618309673	0.147231628	-4.199571	2.67421E-05	0.007789979	0.000513449	-0.377679445	0.228927734	-1.649775841	0.098988792	0.613372895	0.136589851
177.106	100.743	-0.595987729	0.145425015	-4.098247679	4.1629E-05	0.01027093	0.000676972	-0.363696377	0.223558322	-1.626852329	0.103768465	0.615126012	0.139325491
198.085	100.113	-0.592505245	0.144708115	-4.094485263	4.23107E-05	0.01027093	0.000676972	-0.48191034	0.219925517	-2.191243415	0.028434182	0.463128604	0.064181531
113.071	101.512	-0.593242021	0.146019666	-4.062754275	4.84971E-05	0.010867075	0.000716264	-0.376550948	0.214824071	-1.752834055	0.079630496	0.575813805	0.117608117
176.103	102.372	-0.585063405	0.147458401	-3.967650531	7.25847E-05	0.011535695	0.000760334	-0.326866907	0.228611591	-1.429791487	0.152776873	0.671001552	0.183332248
190.119	93.248	0.580428317	0.146003808	3.975432721	7.02514E-05	0.011535695	0.000760334	0.531962821	0.20233519	2.629116667	0.008560699	0.422666356	0.04200008
307.019	63.703	-0.556799047	0.138839823	-4.01036991	6.06237E-05	0.011535695	0.000760334	-0.070393758	0.190149933	-0.370201328	0.711232487	0.954580228	0.722521892
362.062	90.079	0.588310864	0.147674226	3.983842532	6.78098E-05	0.011535695	0.000760334	0.207158225	0.186484459	1.110860531	0.266628387	0.771932314	0.299372224
373.057	87.578	-0.503904285	0.126429797	-3.985644968	6.72971E-05	0.011535695	0.000760334	-0.417798116	0.194334806	-2.149888253	0.031564055	0.463128604	0.064594706
744.59	42.524	-0.607387161	0.15341653	-3.959072478	7.52414E-05	0.011535695	0.000760334	-0.902724965	0.255980056	-3.526544129	0.000421021	0.175204881	0.013472672
165.033	87.089	-0.557440126	0.143410446	-3.887025944	0.00010148	0.013436863	0.000885643	-0.393594384	0.212396831	-1.853108551	0.063866801	0.547176824	0.099694519
250.064	59.738	-0.551582428	0.141211154	-3.906082562	9.38045E-05	0.013436863	0.000885643	-0.382697537	0.228190087	-1.67709975	0.093522978	0.604063047	0.131068699
520.12	55.829	-0.505705646	0.129923217	-3.892342394	9.9281E-05	0.013436863	0.000885643	-0.140918799	0.186478767	-0.755682811	0.449839393	0.861615392	0.474555843
199.088	100.117	-0.54651762	0.14238068	-3.838425413	0.000123826	0.015682809	0.001033676	-0.47586179	0.216747621	-2.195464883	0.028130267	0.463128604	0.064181531
159.092	58.364	-0.534204628	0.140155219	-3.811521479	0.000138114	0.016763593	0.001104912	-0.095142138	0.197778601	-0.481053754	0.630478295	0.923370172	0.647336003
257.147	74.625	-0.58287575	0.154069355	-3.783203678	0.000154823	0.018039932	0.001189038	-0.47277731	0.215547063	-2.19338321	0.028279781	0.463128604	0.064181531
482.36	49.589	-0.577227092	0.152976947	-3.77329462	0.000161106	0.018050052	0.001189705	-0.53826613	0.239992662	-2.242844117	0.024906868	0.463128604	0.061309215
161.137	55	0.626028307	0.166974276	3.749250025	0.000177364	0.019135625	0.001261256	0.243641223	0.197245262	1.235219645	0.21674877	0.741761124	0.24919619
231.055	75.316	-0.540014569	0.145500149	-3.711436544	0.000206086	0.021440339	0.001413163	-0.610354638	0.211804265	-2.881691919	0.003955463	0.368303077	0.0332627
1041.685	49.43	-0.542568317	0.148629042	-3.650486546	0.000261744	0.022765933	0.001500535	-0.143075991	0.217673445	-0.657296489	0.510990293	0.886647679	0.536120964
1152.248	67.801	-0.421609682	0.115375168	-3.654249792	0.000257935	0.022765933	0.001500535	-0.38686355	0.190329324	-2.032600869	0.042092864	0.508782207	0.077709902
164.029	86.645	-0.546120349	0.148987661	-3.665540785	0.000246816	0.022765933	0.001500535	-0.10599397	0.199705433	-0.530751557	0.595590953	0.910738292	0.618126827
220.067	99.688	-0.506844842	0.138705128	-3.654117557	0.000258068	0.022765933	0.001500535	-0.484671621	0.209420065	-2.314351406	0.020648454	0.458365787	0.056934689
265.035	59.429	-0.509638	0.139593741	-3.650866	0.000261357	0.022765933	0.001500535	-0.079742	0.192787826	-0.413630	0.679144463	0.938540344	0.693594345

		16		833				996		868			
402.083	87.844	-0.496253504	0.136086149	-3.646612874	0.00026572	0.022765933	0.001500535	-0.386183285	0.195929254	-1.971034326	0.04871995	0.533538397	0.085818627
288.119	128.322	0.70981216	0.195053182	3.639069878	0.000273625	0.02277338	0.001501026	0.498144474	0.250376072	1.989584989	0.046636671	0.522510088	0.082909637
664.526	68.434	-0.563572695	0.155293161	-3.629088955	0.000284423	0.023014579	0.001516924	-0.455766691	0.214664988	-2.123153362	0.033741003	0.474683085	0.066786314
130.123	27.204	0.499833629	0.138439666	3.610479885	0.000305631	0.023227383	0.001570205	0.467888753	0.196398014	2.382349719	0.017202552	0.458365787	0.054525047
189.123	85.138	0.497171602	0.13812399	3.599458739	0.00031888	0.023227383	0.001570205	0.195274128	0.184740487	1.057018585	0.290503105	0.7858821	0.322408071
369.894	67.014	-0.439212727	0.122023753	-3.599403531	0.000318948	0.023227383	0.001570205	-0.161883306	0.176899887	-0.915112548	0.360132548	0.827992197	0.392871871
668.521	68.55	-0.573202587	0.15974015	-3.588343856	0.000332785	0.023643983	0.001597369	-0.649623034	0.227699504	-2.852983962	0.004331081	0.371071712	0.0332627
176.06	99.313	-0.482490169	0.135101186	-3.571324454	0.000355181	0.024208935	0.001663285	-0.402969624	0.195579324	-2.060389699	0.039361301	0.494221851	0.074825443
106.999	75.195	-0.507734445	0.14317082	-3.546354254	0.000390601	0.024208935	0.001666563	-0.459934748	0.210311137	-2.186925306	0.028747977	0.463128604	0.064181531
149.077	96.666	-0.442109374	0.124418989	-3.55339146	0.000380298	0.024208935	0.001666563	-0.194334249	0.188342881	-1.031810962	0.302160683	0.790401572	0.331513435
188.071	59.806	-0.535907906	0.151038559	-3.548152942	0.000387943	0.024208935	0.001666563	-0.071941496	0.198226733	-0.362925295	0.71666069	0.955716009	0.724204487
219.026	74.325	0.51396115	0.144438452	3.558340189	0.000373206	0.024208935	0.001666563	0.793018961	0.25708196	3.084693151	0.002037623	0.339565997	0.0332627
322.81	69.374	-0.572392513	0.162742891	-3.517158313	0.000436194	0.026471495	0.001820634	-0.425143845	0.212518417	-2.000503533	0.045445919	0.515112691	0.081547817
258.15	74.904	-0.482266954	0.13810029	-3.49215019	0.000479149	0.028484904	0.001957374	-0.531799802	0.191381837	-2.778737054	0.005457068	0.383058261	0.036129553
670.518	68.336	-0.464556785	0.133859415	-3.470482716	0.000519524	0.02969299	0.002036989	-0.485999736	0.21724143	-2.237141123	0.025277118	0.463128604	0.061432996
745.593	42.325	-0.444783578	0.12816823	-3.470310686	0.000519857	0.02969299	0.002036989	-0.44557123	0.207223362	-2.150197861	0.031539567	0.463128604	0.064594706
189.074	58.358	-0.518987416	0.150258035	-3.453974464	0.00055239	0.030944445	0.002121176	-0.043220283	0.195112534	-0.22151464	0.824691739	0.973344729	0.829009497
117.077	78.853	0.494371296	0.143365761	3.448321923	0.000564081	0.031003185	0.0021236	0.413356749	0.1909637	2.164582844	0.030419646	0.463128604	0.064594706
314.158	42.873	-0.472295461	0.137450118	-3.436122629	0.000590104	0.031501286	0.002178845	-0.472432927	0.211975534	-2.228714404	0.025832913	0.463128604	0.061998992
568.426	36.345	-0.535838767	0.15709912	-3.410832392	0.000647649	0.033689308	0.0023462	-0.197361641	0.190877441	-1.033970487	0.301149961	0.790401572	0.331513435
121.063	90.503	-0.448959865	0.132206955	-3.395886898	0.000684066	0.033786752	0.002346233	-0.008974379	0.190890655	-0.047013193	0.962502713	0.993038724	0.962502713
261.043	87.544	-0.468891274	0.13808033	-3.395786152	0.000684318	0.033786752	0.002346233	-0.343668833	0.19253515	-1.784966708	0.074266717	0.567310582	0.111400076
331.092	96.557	-0.41117025	0.120990545	-3.398366773	0.000677895	0.033786752	0.002346233	-0.398676572	0.209184376	-1.905862084	0.056668101	0.542246239	0.094611091
146.118	59.646	-0.539400743	0.159258284	-3.386955644	0.000706728	0.03389374	0.002373044	-0.57607592	0.228751241	-2.518351012	0.011790575	0.445938751	0.04585224
326.805	68.896	-0.545840006	0.161345557	-3.38304951	0.000716857	0.03389374	0.002373044	-0.437373722	0.215138992	-2.032982113	0.04205433	0.508782207	0.077709902
551.425	36.279	-0.482738631	0.1435371	-3.36316276	0.000770549	0.034532458	0.002504892	-0.160803966	0.185141277	-0.868547356	0.385094759	0.83852592	0.417729908
666.523	68.093	-0.536800203	0.159818403	-3.358813466	0.000782779	0.034549012	0.002504892	-0.559167927	0.219904327	-2.542778196	0.010997502	0.445938751	0.04585224
101.071	96.528	-0.455193769	0.136663177	-3.33077116	0.000866058	0.034984508	0.002512376	-0.353250963	0.212984334	-1.658577214	0.097201016	0.611547648	0.135236197
1038.657	51.615	-0.390341753	0.117146164	-3.332091631	0.000861959	0.034984508	0.002512376	-0.392454894	0.182998882	-2.144575365	0.031986814	0.465887941	0.064647034
127.03	48.917	-0.381408269	0.114054576	-3.344085625	0.000825543	0.034984508	0.002512376	-0.097303536	0.192611368	-0.505180647	0.613431962	0.916843153	0.63322009
146.6	96.634	-0.441729407	0.132482914	-3.3342368	0.000855338	0.034984508	0.002512376	-0.318041034	0.198953915	-1.598566355	0.109916991	0.619367155	0.145545257
177.1	100.826	-0.392340885	0.11784209	-3.329378187	0.000870401	0.034984508	0.002512376	-0.201327313	0.187000652	-1.076612892	0.281653242	0.781295409	0.314403619
214.18	26.723	-0.399431308	0.11981088	-3.333848385	0.000856533	0.034984508	0.002512376	-0.503336345	0.201912821	-2.492839945	0.012672596	0.450930086	0.046244752
382.766	68.554	-0.548541644	0.164857668	-3.327365047	0.000876714	0.034984508	0.002512376	-0.355080174	0.209037069	-1.698646927	0.089385729	0.594824622	0.12712637

353.9	79.616	-0.48878162	0.147250146	-3.319396512	0.000902122	0.035511925	0.002547169	-0.109701872	0.188929891	-0.580648573	0.561477328	0.899166277	0.585889386
225.034	75.303	0.472677224	0.143803265	3.286971438	0.001012711	0.038637219	0.002801298	0.592241074	0.201798672	2.934811557	0.003337503	0.3600795	0.0332627
496.686	67.924	-0.502416779	0.152961766	-3.284590612	0.001021307	0.038637219	0.002801298	-0.878850863	0.260479844	-3.373968794	0.000740928	0.217278627	0.017782264
170.055	95.669	-0.584377193	0.179201198	-3.261011642	0.001110155	0.040423512	0.002960413	-0.536844952	0.23435895	-2.290695334	0.021981041	0.458365787	0.058616108
206.094	58.321	-0.482217096	0.147747012	-3.263802695	0.001099277	0.040423512	0.002960413	-0.170109933	0.201104561	-0.845878044	0.397620782	0.844835404	0.428894327
205.043	58.162	-0.372126851	0.115371956	-3.22545325	0.001257734	0.044470922	0.003287632	-0.147132014	0.1860684	-0.790741544	0.429094828	0.856311752	0.460258139
476.306	44.819	0.490811949	0.152268744	3.223326959	0.001267108	0.044470922	0.003287632	0.366103407	0.195648998	1.871225568	0.061313822	0.547176824	0.098102116
363.017	77.333	-0.380775173	0.118636699	-3.209590086	0.001329244	0.046096278	0.003402864	-0.305950045	0.181211638	-1.688357594	0.091342606	0.595497664	0.128954267
169.059	96.539	-0.461825394	0.144249553	-3.201572439	0.001366797	0.046104749	0.003433483	-0.406988503	0.21815956	-1.865554284	0.062103751	0.547176824	0.098544794
244.079	71.325	0.443759592	0.138699339	3.199435523	0.00137697	0.046104749	0.003433483	0.655715699	0.204602967	3.20482009	0.001351469	0.328069164	0.02594821
281.008	53.407	-0.441575424	0.138501119	-3.188244449	0.001431395	0.047382418	0.003523433	-0.290953299	0.19473463	-1.494101477	0.135149098	0.663894304	0.171108328
608.565	68.642	-0.524591068	0.164954907	-3.18020894	0.001471689	0.048168876	0.003576052	-0.534158038	0.225343389	-2.370418051	0.017767982	0.458365787	0.055023429
90.055	84.601	0.475326364	0.149632744	3.176619969	0.001490021	0.048227028	0.003576052	0.380226215	0.195796212	1.941948777	0.052143302	0.540452117	0.090193819
326.987	76.127	-0.449972085	0.142638858	-3.154624846	0.001607046	0.050883969	0.00376284	-0.528204324	0.202229739	-2.611902323	0.009003998	0.427423799	0.04200008
496.84	72.752	0.509702811	0.161418274	3.157652461	0.00159045	0.050883969	0.00376284	0.366087605	0.197464693	1.853939559	0.063747808	0.547176824	0.099694519
272.185	35.432	0.441644113	0.140272677	3.148468553	0.001641284	0.051409245	0.003796705	0.227841443	0.187924153	1.212411708	0.225354821	0.741761124	0.257548367
85.052	96.569	-0.396891353	0.126303213	-3.142369418	0.001675864	0.05193397	0.003830547	-0.139040909	0.183473031	-0.757827506	0.448554254	0.861615392	0.474555843
148.08	94.797	-0.434929144	0.1388811	-3.131665461	0.001738178	0.052588037	0.003890969	-0.39547612	0.217958324	-1.814457521	0.069607317	0.566316992	0.105786434
508.711	67.545	-0.480383846	0.153434097	-3.130880656	0.00174283	0.052588037	0.003890969	-0.40099012	0.199726838	-2.00769273	0.044675954	0.513064096	0.080922482
148.06	89.479	0.461238905	0.14766808	3.123484131	0.001787235	0.052588037	0.003899422	0.277111502	0.190849152	1.451992318	0.146503741	0.6699793	0.180312297
249.108	93.074	-0.481367909	0.153994881	-3.125869545	0.001772802	0.052588037	0.003899422	-0.221496392	0.191092562	-1.159105253	0.246413285	0.752412892	0.279948821
254.824	72.613	-0.4934883	0.159424961	-3.095426816	0.001965299	0.056682327	0.004239745	-0.396665377	0.211862282	-1.872279355	0.061167965	0.547176824	0.098102116
173.092	90.691	0.445880139	0.144725035	3.080877749	0.002063914	0.057372373	0.00426943	0.340553943	0.191667933	1.776791439	0.075602561	0.570964665	0.112524742
246.955	138.586	-0.399506132	0.129697656	-3.080287997	0.002068005	0.057372373	0.00426943	-0.150219262	0.191530829	-0.784308526	0.432859098	0.856844348	0.461716371
301.118	42.143	-0.413010518	0.134014671	-3.081830622	0.002057319	0.057372373	0.00426943	-0.229229966	0.200501817	-1.143281241	0.252921841	0.762692881	0.285652903
662.53	68.617	-0.4954505	0.160358153	-3.089649576	0.002003928	0.057229819	0.00426943	-0.464273075	0.217557254	-2.134027091	0.032840563	0.466656394	0.065681126
315.161	42.821	-0.426957402	0.139110702	-3.069191634	0.002146389	0.058412183	0.004384113	-0.480418646	0.200602556	-2.394877994	0.016625897	0.458365787	0.054525047
138.013	74.729	-0.385996835	0.126485535	-3.05170734	0.002275438	0.060012666	0.004598781	-0.276631598	0.188729206	-1.465759344	0.142713861	0.6699793	0.177570844
221.07	102.078	-0.40598331	0.133567025	-3.039547437	0.002369339	0.061623965	0.004738678	-0.524042708	0.201235296	-2.604129189	0.009210803	0.427423799	0.04200008
130.077	96.602	-0.450359992	0.150761258	-2.987239546	0.00281509	0.070692747	0.005572138	-0.309547561	0.188441477	-1.642672119	0.10045079	0.613444763	0.136784055
365.105	100.434	0.424917634	0.142460494	2.982705041	0.002857132	0.07113526	0.005597646	0.273622104	0.182736391	1.497359686	0.134299692	0.662636461	0.171108328
130.05	94.746	-0.426339652	0.143419822	-2.972668945	0.002952227	0.071665307	0.005668276	-0.379806821	0.220150244	-1.725216445	0.084488473	0.586621414	0.122892325
193.028	89.085	0.464375199	0.156149315	2.973917622	0.00294024	0.071665307	0.005668276	0.233228338	0.183998268	1.26755725	0.204956107	0.727207234	0.237057666
360.15	108.476	0.492107305	0.166059781	2.963434622	0.003042267	0.073240688	0.005783319	0.5158379	0.190723768	2.70463354	0.006837978	0.383058261	0.038614463
238.107	39.303	0.389466899	0.13182781	2.954360693	0.003133176	0.074810991	0.005897743	0.396148921	0.18817144	2.105255301	0.035269091	0.474683085	0.069098627
212.101	91.485	0.525245623	0.178045539	2.950063367	0.003177088	0.075242733	0.005922338	0.299125395	0.204401079	1.463423754	0.143351463	0.6699793	0.177570844
604.571	68.42	-0.485039328	0.164842087	-2.94244836	0.003256281	0.07588437	0.006011596	-0.468340993	0.217921708	-2.149125015	0.031624491	0.463128604	0.064594706

151.048	94.49	-0.424755106	0.14464733	-2.936487698	0.00331952	0.076744138	0.006069979	-0.323685285	0.213698658	-1.514680943	0.129853223	0.65219097	0.167874828
147.555	96.563	-0.409611016	0.140183856	-2.921955694	0.00347841	0.078547355	0.006300517	-0.396920213	0.189729688	-2.092030079	0.036435825	0.47809711	0.069956784
606.568	68.596	-0.473062243	0.162708573	-2.907420513	0.003644229	0.081035423	0.006539178	-0.48023767	0.221672984	-2.166423991	0.030278802	0.463128604	0.064594706
570.665	67.778	-0.446414172	0.154207113	-2.894900011	0.003792793	0.083700051	0.006742744	-0.32317575	0.199276141	-1.621748335	0.104857245	0.616513563	0.13980966
1134.187	68.175	-0.401333526	0.13961565	-2.874559729	0.004045912	0.087301798	0.007126745	-0.276719035	0.194446126	-1.423114157	0.154703065	0.671001552	0.184490612
346.862	70.229	-0.341827694	0.119300118	-2.865275396	0.004166467	0.087948688	0.007206862	-0.25468639	0.181230167	-1.405320062	0.159926125	0.683670397	0.188379239
722.486	66.165	-0.46468136	0.162101556	-2.866606406	0.004148987	0.087948688	0.007206862	-0.610934292	0.231406946	-2.640086229	0.008288494	0.422532617	0.04200008
298.101	114.873	-0.370104028	0.12960336	-2.855666923	0.004294654	0.088725726	0.007297111	-0.268362034	0.186226205	-1.441054085	0.149569404	0.6699793	0.18061211
374.734	70.26	-0.46383103	0.162400786	-2.856088572	0.004288955	0.088725726	0.007297111	-0.281332094	0.202751312	-1.38757225	0.165267336	0.688732119	0.193483711
120.003	86.774	-0.452600067	0.159881844	-2.830840932	0.00464258	0.093334539	0.007689766	-0.277839156	0.212308933	-1.308655046	0.190651226	0.712047778	0.221848699
131.111	109.554	-0.411377464	0.14533162	-2.830612254	0.004645901	0.093334539	0.007689766	-0.5489006	0.19881083	-2.760919005	0.005763897	0.383058261	0.03688894
149.081	97.299	-0.389506079	0.137383141	-2.835181051	0.004579972	0.093334539	0.007689766	-0.414227445	0.216538432	-1.912951159	0.055754305	0.542246239	0.094611091
614.6	67.891	-0.424079091	0.150125679	-2.824827133	0.004730613	0.093743373	0.007763057	-0.80664229	0.268691155	-3.002117016	0.002681091	0.339565997	0.0332627
546.83	77.148	-0.407550294	0.144716591	-2.816196063	0.0048596	0.095006811	0.007840699	-0.570770623	0.210547197	-2.710891571	0.006710257	0.383058261	0.038614463
672.558	68.111	-0.433896215	0.154005464	-2.817407925	0.004841299	0.095006811	0.007840699	-0.537560941	0.213836102	-2.513892353	0.011940687	0.445938751	0.04585224
329.03	87.116	-0.387795748	0.138292922	-2.804161941	0.005044756	0.096407601	0.007939288	-0.55208192	0.206831253	-2.669238384	0.007602347	0.407244855	0.041008751
343.123	109.031	0.446799986	0.159220053	2.806179101	0.005013282	0.096407601	0.007939288	0.462266147	0.201781757	2.290921406	0.02196796	0.458365787	0.058616108
542.153	102.319	-0.376557443	0.13410424	-2.807945846	0.004985861	0.096407601	0.007939288	-0.518778769	0.200376689	-2.589017572	0.009625018	0.427423799	0.04200008
324.99	75.983	-0.385236098	0.138052056	-2.790513303	0.005262454	0.098900179	0.008214562	-0.417162016	0.221990316	-1.879190155	0.060218533	0.547176824	0.098102116
216.063	122.209	0.441718632	0.15862508	2.784670819	0.005358209	0.100054248	0.008296582	0.508823467	0.225194266	2.259486774	0.023853122	0.463128604	0.061034957
104.992	76.36	0.38796751	0.139495266	2.781223478	0.005415444	0.100478914	0.008318123	0.593023619	0.195162216	3.038619005	0.002376652	0.339565997	0.0332627
130.041	96.347	-0.356848212	0.129016637	-2.76590849	0.005676446	0.101444709	0.008383674	-0.286250098	0.179734673	-1.592625916	0.111244136	0.619367155	0.146293658
163.06	105.755	0.40982397	0.148100371	2.767204203	0.005653933	0.101444709	0.008383674	0.367446791	0.188554464	1.948756786	0.051324477	0.539740796	0.089584541
226.038	75.461	0.536100289	0.193766293	2.76673657	0.005662049	0.101444709	0.008383674	0.765225745	0.26407112	2.897801719	0.003757881	0.368303077	0.0332627
312.782	71.919	-0.460637541	0.166415993	-2.767988418	0.005640346	0.101444709	0.008383674	-0.516177056	0.220190509	-2.344229359	0.019066442	0.458365787	0.056009216
349.064	96.841	-0.377877639	0.13645249	-2.76929823	0.005617719	0.101444709	0.008383674	-0.43464877	0.185794172	-2.339410143	0.019314217	0.458365787	0.056009216
245.049	81.304	0.412892557	0.149511996	2.761601531	0.005751863	0.102165712	0.008430211	0.389937034	0.1937757	2.012311315	0.04418713	0.513064096	0.080799324
1047.732	48.709	-0.367086027	0.133519102	-2.749314674	0.005972002	0.104784361	0.008621236	-0.346751293	0.240352027	-1.442680961	0.149110365	0.6699793	0.18061211
509.379	48.096	-0.39223125	0.142546443	-2.751603202	0.005930433	0.104699099	0.008621236	-0.391170082	0.27037077	-1.446791312	0.147955381	0.6699793	0.18061211
165.013	74.843	0.405655768	0.148002166	2.740877235	0.00612754	0.105618481	0.008779758	0.794638668	0.224370005	3.541643945	0.000397642	0.175204881	0.013472672
444.719	68.358	-0.376287938	0.137854333	-2.72960545	0.006341017	0.107391752	0.009018335	-0.37685554	0.204927348	-1.838971445	0.065919386	0.550482389	0.102068727
289.975	84.825	-0.382209409	0.140622167	-2.717988325	0.006568016	0.10995764	0.009272493	-0.276894374	0.188472609	-1.469149154	0.141792338	0.6699793	0.177570844
205.068	73.03	0.472117252	0.174400275	2.707090069	0.006787584	0.111707526	0.009512526	0.758191609	0.256820423	2.952224746	0.003154932	0.354881475	0.0332627
428.698	68.62	-0.441746872	0.16351188	-2.701619434	0.006900269	0.11292407	0.009600375	-0.411947361	0.215763091	-1.909257784	0.056228845	0.542246239	0.094611091
276.119	116.59	-0.36681849	0.136579208	-2.685756448	0.007236582	0.117766271	0.009995854	-0.464821416	0.200792734	-2.31493146	0.020616683	0.458365787	0.056934689
147.076	98.78	-0.369932288	0.138455943	-2.671841173	0.007543634	0.119055477	0.010295863	-0.386394886	0.221315028	-1.745904421	0.080827562	0.575813805	0.118464823

98.058	59.679	0.466225408	0.174546414	2.671068385	0.007561024	0.119055477	0.010295863	0.404577794	0.236078624	1.713741749	0.086576166	0.590623819	0.124982134
232.027	52.237	0.345876449	0.129680168	2.667149915	0.007649754	0.119805019	0.010343329	0.428513166	0.179276242	2.390239562	0.016837386	0.458365787	0.054525047
334.836	67.586	-0.38651894	0.145057893	-2.664584001	0.007708361	0.119940598	0.010349688	-0.308492631	0.217571592	-1.417890215	0.15622282	0.673339313	0.185152972
207.897	68.486	-0.434625375	0.163307813	-2.661387517	0.007781934	0.119940598	0.010375912	-0.294725757	0.194794698	-1.513007076	0.130277862	0.65219097	0.167874828
1154.245	67.712	-0.300973479	0.113441395	-2.65311864	0.007975183	0.120780694	0.010523561	-0.266997145	0.169796601	-1.572452829	0.115845571	0.625125407	0.1513085
438.727	68.044	-0.376943772	0.142137101	-2.651973125	0.008002291	0.120780694	0.010523561	-0.534085109	0.214470842	-2.490245779	0.012765478	0.450930086	0.046244752
480.345	47	-0.407875883	0.154937997	-2.632510366	0.008475644	0.125327665	0.011070228	-0.570342478	0.239662032	-2.37977819	0.017323062	0.458365787	0.054525047
335.84	67.832	-0.380399962	0.144644609	-2.629893811	0.008541154	0.125658497	0.011080416	-0.363252096	0.198912779	-1.826187829	0.067821974	0.560699042	0.104174552
131.053	97.331	-0.373652834	0.142454464	-2.622963325	0.008716866	0.126329499	0.011083697	-0.352326439	0.216512995	-1.627276178	0.103678455	0.615126012	0.139325491
151.144	58.911	0.439654745	0.167537734	2.624213263	0.008684939	0.126329499	0.011083697	0.566852745	0.242469586	2.3378303	0.019396053	0.458365787	0.056009216
314.779	72.2	-0.43329367	0.164956001	-2.626722685	0.008621156	0.126198131	0.011083697	-0.503552261	0.221156034	-2.276909441	0.022791634	0.463128604	0.059945119
388.254	41.378	0.358542338	0.13805211	2.597152181	0.009400027	0.132923679	0.011873718	0.487481873	0.194654688	2.504341804	0.012267945	0.44670656	0.046185206
566.67	67.811	-0.368004079	0.142563262	-2.581338796	0.009841794	0.135832901	0.012350487	-0.49692985	0.21917311	-2.267293872	0.02337228	0.463128604	0.06064159
556.859	81.955	-0.361587538	0.140950673	-2.56534808	0.010307235	0.139651045	0.012850578	-0.523882339	0.213420421	-2.454696398	0.014100362	0.458365787	0.048449413
430.696	70.699	-0.415586201	0.162781253	-2.553034778	0.010678883	0.142043776	0.013228036	-0.501721254	0.223251404	-2.247337513	0.024618465	0.463128604	0.061309215
316.776	71.543	-0.425227532	0.167183706	-2.543474743	0.010975599	0.144669323	0.01350843	-0.556038614	0.224635572	-2.475291906	0.013312732	0.450930086	0.047334157
486.656	68.489	-0.368297291	0.145502553	-2.531208445	0.011367027	0.147822102	0.013901078	-0.53414768	0.217671723	-2.453913962	0.014131079	0.458365787	0.048449413
210.888	69.976	-0.392816262	0.156258496	-2.513887384	0.011940856	0.153910233	0.014510407	-0.395727839	0.211270039	-1.873090196	0.061055931	0.547176824	0.098102116
277.953	141.319	-0.336687615	0.135124646	-2.491681758	0.01271399	0.158528068	0.015304633	-0.296204702	0.179865561	-1.64681165	0.099596766	0.613372895	0.136589851
572.662	68.132	-0.355256477	0.142640669	-2.490569336	0.012753861	0.158528068	0.015304633	-0.601640492	0.197430969	-3.047346092	0.002308717	0.339565997	0.0332627
386.76	68.644	-0.321835317	0.135607495	-2.373285624	0.017630624	0.198293469	0.021025341	-0.536668238	0.223371735	-2.402578992	0.016279918	0.458365787	0.054525047
390.798	67.798	-0.361543859	0.152521225	-2.370449483	0.017766472	0.199052815	0.021056559	-0.398888646	0.220127096	-1.812083351	0.069973318	0.566316992	0.105786434
1034.331	67.999	-0.314662882	0.133604963	-2.355173597	0.018514054	0.203517031	0.021807966	-0.595894729	0.200940958	-2.965521491	0.003021702	0.354881475	0.0332627
336.834	67.349	-0.343891233	0.146157915	-2.352874518	0.018628919	0.203517031	0.021809467	-0.410209746	0.219079171	-1.872426953	0.061147559	0.547176824	0.098102116
548.827	78.227	-0.331337171	0.14147848	-2.341961623	0.019182687	0.20568383	0.022321672	-0.469198065	0.208108807	-2.254580536	0.02415967	0.463128604	0.061034957
159.084	121.116	0.368253409	0.158493139	2.323465929	0.020154136	0.211183446	0.023310808	0.572866571	0.218825682	2.617912884	0.008846939	0.427423799	0.04200008
223.985	85.258	-0.311302331	0.134259252	-2.31866576	0.020413166	0.213131018	0.02346903	-0.535802197	0.21058541	-2.544346242	0.01094825	0.445938751	0.04585224
214.023	130.222	-0.292388588	0.126875562	-2.304530399	0.021192888	0.213615511	0.024158053	-0.46284319	0.184043093	-2.514863132	0.01190786	0.445938751	0.04585224
504.871	71.618	0.343738061	0.14924727	2.303144709	0.021270703	0.213660539	0.024158053	0.635754681	0.221917069	2.864830015	0.004172331	0.368303077	0.0332627
682.586	67.347	-0.314502587	0.136679055	-2.301029868	0.021389943	0.214119944	0.024158053	-0.459323032	0.196714577	-2.334972014	0.019544883	0.458365787	0.056009216
351.088	56.365	0.29073765	0.126504309	2.298243055	0.021547959	0.214229372	0.0241942	0.362524411	0.176597383	2.052830031	0.040089065	0.496832581	0.075461769
207.024	79.355	0.409498103	0.179317396	2.283649625	0.022392131	0.216844673	0.024995867	0.750501608	0.276769867	2.711644939	0.006695026	0.383058261	0.038614463
740.546	67.144	-0.307972557	0.136065283	-2.263417605	0.023609959	0.221975008	0.02620296	-0.514667549	0.20461213	-2.515332545	0.011892016	0.445938751	0.04585224
101.977	134.09	-0.322187472	0.144420801	-2.230893819	0.025688163	0.225952568	0.028345559	-0.394956046	0.204332474	-1.932908846	0.053247428	0.540452117	0.091281305
202.119	68.492	0.40265216	0.182014192	2.212201999	0.026952711	0.229724076	0.029570974	0.689301518	0.261591343	2.635031838	0.008412939	0.422532617	0.04200008
1128.196	68.006	-0.282411144	0.127824771	-2.209361623	0.027149498	0.229902582	0.029617634	-0.48689923	0.187590631	-2.595541296	0.009444205	0.427423799	0.04200008

247.14	110.194	0.303447503	0.140372249	2.161734287	0.030638663	0.244024096	0.03323516	0.452417423	0.203522094	2.222940091	0.02621984	0.463128604	0.062150732
271.04	74.974	0.334003622	0.155820921	2.143509486	0.03207221	0.250224382	0.034594744	0.599428288	0.214942015	2.788790675	0.005290525	0.383058261	0.036129553
412.853	73.358	-0.266835102	0.124684829	-2.140076732	0.032348566	0.251283662	0.034697903	-0.430627867	0.196185887	-2.194999209	0.028163655	0.463128604	0.064181531
483.364	49.807	-0.257430038	0.126019539	-2.042778758	0.041074344	0.293979275	0.043812634	-0.751072236	0.267415943	-2.80862924	0.004975291	0.383058261	0.036129553
238.092	127.057	0.326339725	0.161803599	2.016887924	0.043707211	0.305192926	0.04636345	0.481724924	0.22282091	2.16193769	0.030622979	0.463128604	0.064594706
365.152	102.353	-0.238877078	0.119573998	-1.9977343	0.045745473	0.311325756	0.047995251	-0.55848685	0.209530244	-2.665423571	0.007689141	0.407244855	0.041008751
585.195	102.316	-0.281828335	0.141043155	-1.998170952	0.04569813	0.311325756	0.047995251	-0.457222961	0.211163837	-2.165252195	0.030368377	0.463128604	0.064594706
372.1	37.886	0.263599934	0.13249068	1.989573406	0.046637948	0.315211932	0.048665685	0.399965305	0.185020865	2.161730813	0.030638931	0.463128604	0.064594706
150.084	96.708	-0.243766863	0.124424042	-1.959162064	0.05009381	0.325721584	0.051989252	-0.462046711	0.195705551	-2.360927983	0.01822927	0.458365787	0.055555871
199.119	92.991	-0.259497483	0.133888589	-1.938159818	0.052603727	0.334970617	0.054300622	-0.460937448	0.199335722	-2.312367517	0.020757439	0.458365787	0.056934689
174.05	76.594	-0.25642737	0.135869147	-1.887311258	0.059118473	0.353526952	0.060699181	-0.587298724	0.204932424	-2.865816513	0.004159352	0.368303077	0.0332627
1162.277	67.73	-0.266467964	0.148450897	-1.7949906	0.072655198	0.386918813	0.074201053	-0.996351548	0.257683611	-3.866569327	0.000110377	0.107176161	0.007064134
252.108	109.021	0.273174231	0.155498286	1.756766837	0.07895756	0.399388006	0.080210854	0.622285492	0.210872945	2.9509973	0.003167497	0.354881475	0.0332627
318.97	76.169	0.22813404	0.145520056	1.56771545	0.116947554	0.461610062	0.118178581	0.644717582	0.214655737	3.003495704	0.002668973	0.339565997	0.0332627
1150.25	67.735	-0.190526072	0.123997818	-1.536527613	0.124409031	0.476725588	0.125060387	-0.537584532	0.196097768	-2.741410766	0.006117598	0.383058261	0.037889636
358.057	88.539	-0.169459135	0.133900257	-1.265562438	0.205669781	0.583696652	0.205669781	-0.777363281	0.223353888	-3.480410788	0.000500646	0.182297551	0.013731991

Supplementary Table 10. Type 2 diabetes mellitus associated metabolites that overlapped with main analysis stratified by race among Parkinson's disease patients.

C18

		European Ancestry (n = 637)						Non-European Ancestry (n = 194)					
mz	rt	beta	se	zvalue	pvalue	Full MWAS FDR p value	Replication FDR p value	beta	se	zvalue	pvalue	Full MWAS FDR p value	Replication FDR p value
209.067	36.808	-0.954507317	0.14700705	-6.49293566	8.41796E-11	1.87047E-07	1.11959E-08	-0.773971922	0.202285053	-3.826144893	0.000130166	0.069118986	0.004137185
199.038	31.744	-0.887948724	0.141981954	-6.253954825	4.00187E-10	4.44608E-07	2.66124E-08	-0.765856972	0.202019435	-3.791006399	0.000150038	0.069118986	0.004137185
223.082	38.976	-0.871155577	0.141615706	-6.151546337	7.67311E-10	5.68322E-07	3.40175E-08	-0.617792168	0.199253076	-3.100540175	0.00193168	0.187021795	0.011194374
200.041	32.191	-0.655680723	0.113110642	-5.796808422	6.75888E-09	3.75456E-06	2.24733E-07	-0.675587364	0.180810178	-3.736445436	0.00018664	0.069118986	0.004137185
269.088	35.076	0.772545603	0.145604614	5.305776928	1.12194E-07	4.98591E-05	2.98436E-06	0.663962636	0.206426734	3.216456634	0.001297841	0.159798162	0.009564876
113.024	36.458	0.658800824	0.13353003	4.933727826	8.06748E-07	0.000298766	1.78829E-05	0.690244796	0.201794753	3.420528954	0.000624995	0.115728208	0.006927026
143.035	36.297	0.665907017	0.137075453	4.857959643	1.18602E-06	0.000376475	2.25343E-05	0.71507639	0.204231171	3.501308771	0.000462979	0.099680796	0.005966492
174.088	30.043	-0.705282813	0.151262489	-4.662641855	3.12176E-06	0.000770727	4.61326E-05	-0.512754698	0.193482874	-2.650129637	0.008046089	0.410035086	0.030468299
225.062	37.895	0.632637852	0.135010537	4.68584058	2.78813E-06	0.000770727	4.61326E-05	0.73947949	0.2048815	3.609303383	0.00030702	0.08527491	0.005104214
261.041	34.747	-0.549468909	0.121070118	-4.53843541	5.66731E-06	0.001259277	7.53753E-05	-0.084787132	0.185082376	-0.458104838	0.646877123	0.98715165	0.684633658
425.291	227.677	0.752854316	0.169436512	4.443282665	8.85966E-06	0.001789652	0.000107121	0.571959159	0.219265111	2.6085279	0.00909326	0.410035086	0.031539312
215.033	30.913	0.595761364	0.136219265	4.373547059	1.22244E-05	0.002201808	0.000131791	0.615541858	0.195864021	3.142700003	0.001673973	0.177122295	0.01060183
215.091	31.459	0.595903319	0.137069039	4.34746842	1.37718E-05	0.002201808	0.000131791	0.626439687	0.195463531	3.204892926	0.001351127	0.159798162	0.009564876
217.03	30.51	0.586517562	0.135336716	4.333765293	1.4658E-05	0.002201808	0.000131791	0.62616214	0.195574591	3.201653836	0.001366411	0.159798162	0.009564876
218.033	30.739	0.577812336	0.133422425	4.330698808	1.48637E-05	0.002201808	0.000131791	0.638682606	0.197790746	3.229082339	0.001241881	0.159798162	0.009564876
216.035	31.278	0.571937672	0.13349507	4.284335522	1.83286E-05	0.002545386	0.000152357	0.598369279	0.194558795	3.075519042	0.002101365	0.194551385	0.011645065
195.976	28.201	-0.669370046	0.157779339	-4.242444241	2.21098E-05	0.002889887	0.000172977	0.102916308	0.192704713	0.534062225	0.593298483	0.98715165	0.63636047
158.061	87.22	-0.66631609	0.162254313	-4.106615574	4.01499E-05	0.004695421	0.000281049	-0.185781427	0.190920775	-0.973081252	0.330512921	0.936734325	0.366318488
280.622	30.778	-0.505037048	0.122805512	-4.112494935	3.91406E-05	0.004695421	0.000281049	-0.492744305	0.179446496	-2.745912101	0.006034293	0.368879829	0.024755989
216.971	30.784	0.645798851	0.158325963	4.078919466	4.52455E-05	0.005026776	0.000300883	0.089927732	0.205154893	0.438340664	0.661139353	0.98715165	0.688241678
226.065	36.217	0.585651115	0.144838694	4.043471396	5.26656E-05	0.00557252	0.000333549	0.5618585	0.197928983	2.838687356	0.004529952	0.314548521	0.020775296
581.24	42.898	-0.523956389	0.131467264	-3.985451387	6.7352E-05	0.006802549	0.000407173	-0.019472897	0.184005957	-0.105827537	0.915719188	0.991899116	0.922656454
144.97	29.085	-0.662585014	0.167337235	-3.959579065	7.5082E-05	0.007253573	0.00043417	-0.081862092	0.187479215	-0.436646229	0.662367931	0.98715165	0.688241678
103.04	32.211	0.536803872	0.136435902	3.934476668	8.33782E-05	0.007463815	0.000446754	0.119697816	0.184480319	0.64883786	0.516443183	0.978799566	0.563007732
564.083	212.912	-0.546771987	0.139030117	-3.932759297	8.39763E-05	0.007463815	0.000446754	0.045440654	0.193470222	0.234871566	0.814308419	0.98715165	0.833100152
253.093	34.817	-0.466353572	0.120799904	-3.86054588	0.000113134	0.009668605	0.000578724	-0.267643456	0.176105228	-1.519792793	0.128563061	0.808185226	0.162846544
179.056	36.803	0.495972961	0.12946229	3.831022624	0.000127612	0.010501976	0.000628606	0.705829994	0.202575766	3.484276566	0.000493469	0.099680796	0.005966492
311.14	35.236	-0.453743842	0.119589017	-3.794193271	0.000148124	0.011754718	0.00070359	-0.369613694	0.178989411	-2.06500313	0.038922704	0.61337765	0.069955671
89.04	33.444	0.519746566	0.138964539	3.740138092	0.000183919	0.014092015	0.000843491	0.321844492	0.182032528	1.768060335	0.077050818	0.70526798	0.113511705
415.322	215.543	-0.546397006	0.146718306	-3.724122926	0.000195995	0.014516727	0.000868913	-0.08718693	0.191322119	-0.455707528	0.648600308	0.98715165	0.684633658
415.306	235.89	0.6083359	0.163744603	3.71515085	0.000203083	0.01455643	0.000871289	0.572302111	0.231148603	2.475905552	0.013289873	0.454309196	0.035351062
89.009	34.171	0.511531815	0.139106715	3.677261843	0.000235751	0.016243622	0.000972278	0.299079889	0.182115563	1.642253327	0.100537515	0.767873055	0.136478402
89.024	31.295	0.508937389	0.138622815	3.671382572	0.000241242	0.016243622	0.000972278	0.322616927	0.182942736	1.763485852	0.077818581	0.70526798	0.113511705
503.631	32.36	-0.476645927	0.130428956	-3.654448688	0.000257735	0.016843753	0.001008199	-0.354469585	0.202053347	-1.75433661	0.079372846	0.707706536	0.113511705
195.051	31.406	0.490187517	0.134666729	3.640004633	0.000272633	0.017308309	0.001036006	0.828080052	0.213548542	3.87771344	0.000105443	0.069118986	0.004137185

401.291	235.525	0.571872002	0.158535653	3.607213843	0.000309503	0.018097756	0.001112536	0.498339809	0.217841728	2.287623286	0.022159469	0.518298309	0.046050146
90.028	31.729	0.501712093	0.139083496	3.607272658	0.000309432	0.018097756	0.001112536	0.299601655	0.18244696	1.642130158	0.100563033	0.767873055	0.136478402
260.023	28.879	0.453308427	0.125991267	3.597935309	0.000320753	0.018274718	0.001122637	0.39818623	0.209515182	1.900512536	0.057365891	0.617679827	0.089667324
494.81	33.188	-0.423620999	0.117962908	-3.591137298	0.000329238	0.018289178	0.001122786	-0.402408477	0.173432997	-2.320253261	0.020327181	0.502841342	0.043605082
418.95	37.272	-0.43303786	0.121747325	-3.55685728	0.000375318	0.020340401	0.001247932	-0.027460343	0.189010437	-0.145284797	0.884486017	0.98715165	0.897989621
312.143	35.633	-0.453817244	0.128766887	-3.524331861	0.000424552	0.022460831	0.001377206	-0.195527432	0.182397598	-1.071984687	0.28372691	0.920352108	0.322527171
402.293	232.497	0.574976837	0.16445865	3.496178741	0.000471972	0.024388901	0.001494579	0.499308153	0.22077278	2.261638203	0.023719766	0.524986856	0.047798922
160.062	29.915	0.468719955	0.134658831	3.480796261	0.000499926	0.025181901	0.001546282	0.463408868	0.195670352	2.368314176	0.017869354	0.495218884	0.041675666
149.061	32.211	-0.518288412	0.149657676	-3.463159561	0.000533872	0.025538744	0.001596587	-0.174512484	0.192129273	-0.908307624	0.363715716	0.953038113	0.399786696
369.154	47.826	-0.47169822	0.136329418	-3.459988498	0.000540198	0.025538744	0.001596587	-0.007563235	0.204862207	-0.036918644	0.970549874	0.999019928	0.970549874
224.952	56.909	-0.470239747	0.136655891	-3.441049946	0.000579462	0.026824241	0.0016754	-0.295817898	0.181536763	-1.629520617	0.103202854	0.769529557	0.138646258
416.309	235.341	0.553602571	0.161471125	3.428492693	0.000606943	0.027523001	0.001717519	0.604294531	0.247933624	2.437323839	0.014796422	0.485248328	0.038232582
229.012	27.859	0.564551282	0.166297798	3.394821154	0.000686734	0.030518479	0.001902827	0.046958958	0.199521257	0.235358169	0.81393075	0.98715165	0.833100152
193.123	84.259	-0.47495605	0.142094774	-3.342530041	0.000830184	0.035474387	0.002253356	-0.368517852	0.189916342	-1.94042202	0.052328423	0.617679827	0.085542403
508.623	32.501	-0.449194453	0.134681738	-3.335229097	0.000852292	0.03573192	0.002267095	-0.263408913	0.187008015	-1.408543445	0.158970212	0.840640395	0.192209438
282.975	35.721	-0.393300488	0.118280154	-3.325160432	0.000883677	0.03636166	0.00230449	-0.2453487	0.17813902	-1.377287806	0.168423284	0.840640395	0.201804475
566.582	32.607	-0.503574308	0.15268347	-3.298158652	0.000973211	0.038615634	0.002489175	-0.528645269	0.217842534	-2.426731179	0.01523554	0.485248328	0.038232582
163.077	35.967	-0.411534799	0.125671488	-3.274687087	0.001057791	0.039416045	0.002573767	-0.116312407	0.186160751	-0.624795541	0.532105257	0.978799566	0.575365847
180.06	36.11	0.4329036	0.132000711	3.279555058	0.001039709	0.039416045	0.002573767	0.738069274	0.211772667	3.485196109	0.000491776	0.099680796	0.005966492
445.988	31.336	-0.485495277	0.148336004	-3.27294293	0.00106434	0.039416045	0.002573767	-0.236688193	0.19132829	-1.237078914	0.216057787	0.888372882	0.247721428
145.062	30.621	-0.477603858	0.146460094	-3.260982866	0.001110267	0.03995706	0.002601465	-0.624303832	0.201394922	-3.099898581	0.001935869	0.187021795	0.011194374
279.614	29.271	-0.410614545	0.125963163	-3.259798614	0.001114913	0.03995706	0.002601465	-0.349858287	0.187159346	-1.869307055	0.061580107	0.62766513	0.093069935
295.984	32.846	-0.415408684	0.127704822	-3.252881735	0.00114241	0.040292631	0.002619665	-0.229521813	0.180519189	-1.271453819	0.203567246	0.871667248	0.235429946
281.124	29.849	-0.463536498	0.143229932	-3.236310254	0.001210857	0.04139268	0.002684066	-0.298632309	0.187722638	-1.590816706	0.111650832	0.771888293	0.144193403
322.932	37.944	-0.461642869	0.142557337	-3.238296109	0.001202459	0.04139268	0.002684066	-0.281634783	0.19117487	-1.473178891	0.140702805	0.835940196	0.176091057
148.98	33.213	0.471218257	0.14653689	3.215697131	0.001301281	0.043809789	0.002837219	0.195590566	0.187418685	1.043602276	0.29666944	0.927861439	0.334381657
324.921	34.963	-0.425513759	0.134673404	-3.159597569	0.001579872	0.051624638	0.00338908	-0.4631006	0.194720763	-2.378280529	0.017393588	0.495218884	0.041675666
114.056	36.877	0.435913276	0.138366809	3.150417931	0.001630371	0.052502657	0.003441893	0.279115897	0.193088472	1.445533718	0.148308029	0.840640395	0.181065688
501.634	32.333	-0.394886446	0.126233597	-3.12821987	0.001758686	0.055825708	0.003654769	-0.370017842	0.195696529	-1.890773658	0.058654565	0.617679827	0.089667324
414.204	35.694	-0.490589801	0.158261762	-3.099863138	0.001936101	0.060234819	0.00396156	-0.364822855	0.230990749	-1.579382967	0.114248242	0.782944499	0.146105925
270.091	34.705	0.523899344	0.170090463	3.080121802	0.00206916	0.061302301	0.00416967	0.563125889	0.221748533	2.539479654	0.01110175	0.429042621	0.032864064
256.934	34.638	-0.429226424	0.140019952	-3.06546615	0.00217331	0.061911465	0.004314182	-0.357350334	0.188589104	-1.894862039	0.058110682	0.617679827	0.089667324
229.144	107.471	-0.386284133	0.126844333	-3.045340089	0.002324173	0.063461715	0.004387073	-0.368608032	0.192895021	-1.910925588	0.056014144	0.617679827	0.088689062
239.077	35.736	0.406556475	0.133443333	3.046660076	0.002313992	0.063461715	0.004387073	0.651659669	0.199634155	3.26426943	0.001097467	0.159798162	0.009564876
423.275	218.074	0.60710322	0.199480135	3.043426951	0.002339002	0.063461715	0.004387073	0.287283985	0.223344295	1.286283066	0.19834429	0.861835491	0.231401672
496.807	33.723	-0.389770463	0.128085663	-3.043045206	0.002341971	0.063461715	0.004387073	-0.415592418	0.178016745	-2.334569242	0.019565935	0.495218884	0.042660153
118.051	31.331	-0.469613824	0.157218109	-2.987021192	0.002817102	0.073642359	0.005203813	-0.196884312	0.190278697	-1.034715477	0.300801806	0.929416182	0.336190254

431.244	82.667	0.456538107	0.1530701	2.982542686	0.002858648	0.073859482	0.005208221	0.25769356	0.197635972	1.30387984	0.192274555	0.861580981	0.22630545
268.96	35.981	-0.409539874	0.137981324	-2.968081927	0.002996644	0.076534989	0.005385861	-0.385161489	0.185456806	-2.076825854	0.037817635	0.608918732	0.068900623
254.982	28.027	0.435615877	0.14788864	2.945566857	0.003223635	0.081396777	0.005716579	0.287630939	0.199020329	1.445233961	0.14839218	0.840640395	0.181065688
390.709	32.22	-0.421707026	0.14525789	-2.903160901	0.003694168	0.089222185	0.006380835	-0.337620256	0.188713786	-1.789059846	0.073605178	0.697213244	0.109994255
424.278	219.234	0.470152819	0.161789393	2.905955762	0.003661332	0.089222185	0.006380835	0.473586626	0.22691877	2.08703152	0.036885285	0.608918732	0.068135319
254.623	31.407	-0.418161848	0.14582819	-2.867496655	0.004137332	0.097799481	0.007054681	-0.248322231	0.188816014	-1.315154501	0.188457983	0.861580981	0.223793855
177.04	33.332	0.410625885	0.143510485	2.861295358	0.004219138	0.098683414	0.007103106	0.906596054	0.242458863	3.739174723	0.000184625	0.069118986	0.004137185
194.039	27.536	0.403698981	0.14220684	2.83881549	0.004528133	0.103266656	0.007528021	0.30211447	0.2055745	1.469610625	0.141667241	0.837193111	0.176091057
415.216	36.104	-0.46794215	0.16547007	-2.827956435	0.004684618	0.104092214	0.007692027	-0.419895706	0.210399096	-1.995710605	0.045965432	0.617679827	0.080439506
446.67	32.191	-0.422469791	0.1519213	-2.780846338	0.005421739	0.109241822	0.008793797	-0.490285938	0.208948192	-2.346447382	0.018953342	0.495218884	0.042660153
203.021	32.471	-0.356916185	0.1292532	-2.761372125	0.005755905	0.111214099	0.009006299	-0.501402903	0.191479127	-2.618577341	0.008829727	0.410035086	0.031539312
226.018	34.225	-0.41156544	0.148889275	-2.764238325	0.005705585	0.111214099	0.009006299	-0.296189738	0.186196443	-1.590737894	0.111668575	0.771888293	0.144193403
498.638	32.269	-0.422227808	0.152492309	-2.768846572	0.005625512	0.111214099	0.009006299	-0.523575523	0.221174003	-2.367256165	0.017920524	0.495218884	0.041675666
213.049	35.964	-0.398974824	0.14513852	-2.748924442	0.005979117	0.111643675	0.00903662	-0.558489062	0.19810748	-2.819121525	0.004815528	0.320552536	0.021348842
300.877	34.139	-0.376155833	0.136565858	-2.75439146	0.005880139	0.111643675	0.00903662	-0.358316258	0.183492362	-1.952758439	0.050848225	0.617679827	0.084535175
506.626	32.343	-0.410165306	0.149169417	-2.749660846	0.005965698	0.111643675	0.00903662	-0.4674211	0.212278731	-2.201921494	0.027670854	0.580043759	0.054928711
149.046	31.935	0.375848102	0.137031813	2.742779903	0.00609215	0.112336291	0.009104	0.611305139	0.209097049	2.923547426	0.003460675	0.265159307	0.017047029
424.247	181.74	0.479339375	0.1750569	2.738191836	0.006177803	0.112336291	0.00912942	0.353689994	0.213684295	1.655198826	0.097884173	0.76048473	0.135610365
387.275	216.137	0.438414938	0.16224709	2.702143617	0.0068894	0.119463162	0.010069123	0.458965145	0.237583604	1.93180479	0.053383605	0.617679827	0.085542403
161.987	32.772	0.3634533	0.13563017	2.679737853	0.007367984	0.12459113	0.010651542	0.817066963	0.19329057	4.227143428	2.36677E-05	0.052589588	0.003147802
94.942	31.882	-0.404234614	0.151681112	-2.665029335	0.007698161	0.128611377	0.011009198	-0.509454404	0.204849656	-2.486967338	0.012883723	0.454309196	0.035052685
302.874	34.257	-0.335689315	0.128847017	-2.605332455	0.009178515	0.139500063	0.012986623	-0.354094392	0.182932665	-1.935654255	0.052910061	0.617679827	0.085542403
416.953	38.484	-0.312703175	0.120389669	-2.597425335	0.009392554	0.139500063	0.013149575	-0.357687668	0.182121328	-1.964007582	0.049529213	0.617679827	0.084453657
216.909	24.258	0.421210926	0.162432676	2.593141575	0.009510361	0.139500063	0.013175813	0.354623886	0.206671839	1.715879091	0.086184174	0.718634312	0.121941438
373.26	213.573	0.449322504	0.173735256	2.586248267	0.009702702	0.139500063	0.013303704	0.562324839	0.237338647	2.369293187	0.017822119	0.495218884	0.041675666
266.963	36.467	-0.376287762	0.146106841	-2.575428766	0.010011589	0.139910387	0.013587157	-0.310245967	0.18540999	-1.673296934	0.094268856	0.743509159	0.131976398
412.278	210.722	0.377071076	0.149513269	2.521990707	0.011669279	0.156199628	0.01567691	0.349195507	0.216199029	1.615157608	0.106276568	0.769529557	0.139948352
442.676	32.194	-0.381342344	0.151893364	-2.51059252	0.012052873	0.159413599	0.016030322	-0.489583523	0.207270887	-2.362046741	0.018174351	0.495218884	0.041675666
225.054	30.806	-0.348543782	0.139177807	-2.504305748	0.012269196	0.159615045	0.016156466	-0.335478184	0.191170179	-1.754866713	0.079282109	0.707706536	0.113511705
162.056	40.608	-0.409910249	0.165264501	-2.480328487	0.01312614	0.166638751	0.017115456	-0.41593924	0.212711567	-1.955414301	0.05053419	0.617679827	0.084535175
286.06	27.608	0.458002508	0.186011179	2.462231096	0.013807567	0.168341699	0.017829188	0.558099596	0.254611327	2.191966876	0.028381898	0.589388583	0.055511654
403.296	242.279	0.439070239	0.1792086	2.450051164	0.014283592	0.170293545	0.018266516	0.352115997	0.216953826	1.622999712	0.104589466	0.769529557	0.13910399
217.048	30.99	0.444669289	0.185706297	2.394476098	0.016644128	0.180872343	0.021042523	0.434873129	0.219079909	1.984997763	0.04714472	0.617679827	0.08143179
94.908	31.854	-0.378499084	0.158255524	-2.391695881	0.016770732	0.180895957	0.021042523	-0.598585442	0.215187687	-2.781690025	0.005407666	0.343309554	0.023200633
562.588	32.428	-0.342011656	0.143602436	-2.381656369	0.017234972	0.183675039	0.021422909	-0.442321405	0.216134245	-2.046512363	0.040705987	0.617679827	0.072185284
156.878	31.938	-0.362825134	0.152638784	-2.377017979	0.017453237	0.183796652	0.021493339	-0.433679987	0.204635401	-2.119281343	0.034066697	0.608918732	0.064726725
444.673	32.115	-0.350603026	0.149785185	-2.340705623	0.019247336	0.197998063	0.023485282	-0.472706608	0.208538577	-2.266758573	0.023404978	0.524986856	0.047798922

556.597	32.309	-0.361350261	0.154697576	-2.33584953	0.019499085	0.198747556	0.023576167	-0.466418254	0.220903454	-2.111412232	0.034736895	0.608918732	0.065070521
151.007	30.177	0.349221605	0.152009386	2.297368697	0.021597746	0.209798187	0.02587838	0.549042457	0.216895619	2.531367208	0.011361883	0.429042621	0.032864064
439.306	227.232	0.387204894	0.170352771	2.272959177	0.02302864	0.217743142	0.02734651	1.099604154	0.327955829	3.352903216	0.000799687	0.126921695	0.007597023
127.051	32.393	-0.337966166	0.149963003	-2.253663625	0.024217337	0.224212182	0.028503592	-0.604913633	0.210750021	-2.870289781	0.004100958	0.303744274	0.01947955
440.679	32.243	-0.359765506	0.159888415	-2.250103654	0.024442366	0.225356588	0.028516094	-0.56434205	0.218681756	-2.580654459	0.009861323	0.410035086	0.031539312
397.259	210.47	0.347931556	0.15519645	2.241878315	0.024969238	0.228276114	0.028877466	0.612233031	0.241872309	2.531224157	0.011366518	0.429042621	0.032864064
374.263	214.626	0.34820501	0.157202275	2.215012538	0.026759204	0.236180215	0.030680811	0.534171083	0.231737751	2.305067174	0.021162812	0.511127906	0.044677047
452.705	33.294	-0.292403982	0.133271522	-2.194046983	0.028232032	0.244296296	0.032092823	-0.411044547	0.190110845	-2.162130976	0.030608082	0.606057199	0.058998187
189.067	63.388	-0.301482169	0.138635262	-2.174642754	0.029656898	0.252843702	0.033354673	-0.486342296	0.19562238	-2.486128102	0.012914147	0.454309196	0.035052685
219.034	30.853	0.292723814	0.134761674	2.172159233	0.029843654	0.253101527	0.033354673	0.469748387	0.193448242	2.428289765	0.015170217	0.485248328	0.038232582
220.049	30.642	-0.324750615	0.152628839	-2.127714639	0.03336075	0.27053864	0.036974832	-0.502267521	0.199923848	-2.512294189	0.011994905	0.444211308	0.033943029
276	31.551	0.281552378	0.133239834	2.113124659	0.0345901	0.274497147	0.038020523	0.56979511	0.180131095	3.1632246	0.001560319	0.173351444	0.010376122
505.628	32.397	-0.266332442	0.127590614	-2.08739838	0.036852138	0.286219944	0.040174872	-0.538865316	0.197478444	-2.728729797	0.006357878	0.371768523	0.024870521
221.053	30.668	-0.308319387	0.151731334	-2.032008679	0.042152777	0.29829131	0.045579832	-0.518749323	0.201296052	-2.577046686	0.009964849	0.410035086	0.031539312
223.05	30.675	-0.306461056	0.15196981	-2.016591692	0.043738141	0.299234017	0.046912683	-0.517455324	0.201416664	-2.569079015	0.01019692	0.411955585	0.031539312
255.63	33.175	-0.251602554	0.126128704	-1.994808043	0.04606382	0.303945675	0.049011904	-0.409491723	0.175051045	-2.339270371	0.019321445	0.495218884	0.042660153
96.922	31.526	-0.310931832	0.157171831	-1.978292351	0.047895735	0.310274998	0.05055661	-0.528948847	0.204808019	-2.582656916	0.009804277	0.410035086	0.031539312
326.087	28.546	0.263573304	0.136010406	1.937890714	0.052636557	0.32595228	0.055123324	0.620008543	0.184138484	3.367077473	0.000759693	0.126921695	0.007597023
188.057	31.438	0.261568167	0.139635701	1.873218411	0.061038231	0.354117361	0.063422537	0.523216205	0.201846077	2.592154441	0.009537694	0.410035086	0.031539312
356.098	28.295	0.247191454	0.138190411	1.788774292	0.073651173	0.378564181	0.07593493	0.553929935	0.185031531	2.993705617	0.002756117	0.226818244	0.0140986
327.091	28.264	0.232055715	0.136021934	1.706016869	0.088004939	0.40824003	0.090035822	0.677538369	0.18475316	3.667262689	0.000245161	0.077821075	0.004658057
146.065	30.227	-0.24045309	0.141563545	-1.698552332	0.089403564	0.412986922	0.090768504	-0.524358912	0.198486656	-2.641784199	0.008247058	0.410035086	0.030468299
359.121	49.326	0.248223516	0.152619759	1.626417953	0.103860774	0.43231005	0.104647598	0.732047803	0.243474996	3.006665217	0.002641305	0.22572997	0.014051741
406.925	35.575	-0.191484309	0.127230848	-1.505014805	0.13232027	0.471178912	0.13232027	-0.498944253	0.182091269	-2.740077845	0.006142463	0.368879829	0.024755989

Supplementary Table 11. Replicated metabolic feature from each cross-validation in HILIC and C18 column.

PD Patients

HILIC

First validation

Mass to charge ratio	Retention Time	beta	se	zvalue	pvalue
101.071	96.528	-0.777365452	0.392111673	-1.982510353	0.04742215
1037.653	51.282	-0.348823509	0.132312503	-2.6363609	0.008380055
104.992	76.36	1.263303383	0.435375285	2.901642392	0.00371212
106.999	75.195	-0.778343096	0.357105764	-2.179587042	0.029288086
113.071	101.512	-1.130832498	0.373581326	-3.027004883	0.0024699
130.136	49.642	0.416651772	0.092898091	4.485041275	7.29E-06
147.555	96.563	-0.683582341	0.360457378	-1.896430434	0.057903151
149.077	96.666	-0.483709228	0.268618108	-1.800732016	0.071745129
160.133	55.358	0.755903012	0.265020843	2.852239859	0.004341233
165.013	74.843	1.031650828	0.317119884	3.253188719	0.001141177
165.033	87.089	-0.578675885	0.280910353	-2.060001984	0.039398351
169.059	96.539	-0.727120511	0.438473608	-1.658299377	0.097257055
173.092	90.691	0.280582983	0.163216803	1.719081483	0.085599536
190.119	93.248	0.451673015	0.197709256	2.284531461	0.022340316
198.085	100.113	-1.289814777	0.359518018	-3.587622071	0.000333708
203.053	75.296	1.021686306	0.305027292	3.349491447	0.000809601
204.056	74.501	1.081601552	0.307255882	3.520198037	0.000431225
205.068	73.03	0.178986283	0.081045595	2.208464057	0.027211941
214.18	26.723	-0.380768842	0.157478408	-2.417911436	0.015609876
219.026	74.325	0.286914896	0.110599429	2.594180623	0.009481666
220.067	99.688	-0.74362361	0.307187085	-2.420751544	0.015488459
221.07	102.078	-0.218468873	0.125337331	-1.743047105	0.081325387
221.988	85.683	-0.922916299	0.353040698	-2.614192368	0.008943866
225.034	75.303	1.115314772	0.360212323	3.096270452	0.001959715
231.055	75.316	-1.110648047	0.402595171	-2.758721728	0.005802792
244.079	71.325	0.632151434	0.303449659	2.083216821	0.037231464
254.824	72.613	-1.011597758	0.600596311	-1.684322296	0.092119399
257.147	74.625	-0.674619614	0.224829354	-3.000585115	0.002694614
258.15	74.904	-0.257472674	0.080574929	-3.19544402	0.001396159
261.043	87.544	-0.277966358	0.160282635	-1.734226279	0.082877942
278.183	269.785	0.402070012	0.225637052	1.78193257	0.074760228
312.782	71.919	-0.969050573	0.572867291	-1.691579515	0.090726175
313.154	42.894	-0.295113131	0.105143796	-2.806757436	0.005004291
314.158	42.873	-0.231517968	0.097497138	-2.374612973	0.017567359
315.161	42.821	-0.14065596	0.058766913	-2.393454979	0.01669053
316.776	71.543	-1.014794274	0.55611302	-1.824798624	0.068031424
322.81	69.374	-1.460918014	0.781208035	-1.870075508	0.061473333
326.805	68.896	-1.478190939	0.811538488	-1.821467448	0.068535831
326.987	76.127	-0.486396494	0.184478844	-2.636597698	0.008374208

C18

First validation

Mass to charge ratio	Retention Time	beta	se	zvalue	pvalue
113.024	36.458	1.136537119	0.376176935	3.021283374	0.002517057
143.035	36.297	1.155033689	0.384231521	3.006087803	0.002646326
144.97	29.085	-0.242735351	0.118801239	-2.043205553	0.041032094
145.062	30.621	-0.746211919	0.330077718	-2.260715816	0.02377686
158.061	87.22	-0.873890408	0.388087905	-2.251784703	0.024335881
161.987	32.772	0.069694539	0.035408629	1.968292507	0.049034392
177.04	33.332	0.540760552	0.264001219	2.048325967	0.04052807
179.056	36.803	0.817834014	0.328604705	2.488807983	0.012817217
180.06	36.11	0.767394167	0.325620474	2.356713501	0.018437466
193.123	84.259	-0.441899857	0.195566813	-2.259585097	0.023847013
195.051	31.406	0.569084678	0.233482537	2.437375765	0.014794297
199.038	31.744	-0.736859529	0.235039186	-3.135049703	0.00171825
200.041	32.191	-0.171448158	0.04765223	-3.59790417	0.000320792
203.021	32.471	-0.359475452	0.166663176	-2.156897889	0.031013621
209.067	36.808	-0.819723713	0.260961502	-3.141167207	0.001682759
213.049	35.964	-0.579819067	0.319683954	-1.813725899	0.069719936
215.033	30.913	0.975962038	0.368117711	2.651222721	0.008020094
215.091	31.459	1.042696396	0.387101533	2.693599241	0.00706851
216.035	31.278	0.998893992	0.374350315	2.668340196	0.007622703
217.03	30.51	0.987836917	0.374878838	2.635083172	0.008411667
218.033	30.739	0.994574441	0.37506693	2.651725231	0.008008168
223.082	38.976	-0.718168485	0.172324576	-4.167533746	3.08E-05
225.062	37.895	1.322821988	0.447111478	2.958595459	0.003090445
226.065	36.217	0.44341222	0.219680511	2.01844132	0.04354532
239.077	35.736	0.557034214	0.337211873	1.651881972	0.098558614
261.041	34.747	-0.322399942	0.139169782	-2.316594432	0.020525835
269.088	35.076	0.95054895	0.290323573	3.274101854	0.001059984
270.091	34.705	0.391453538	0.176970159	2.211974833	0.026968404
280.622	30.778	-0.190678055	0.048135592	-3.96126955	7.46E-05
300.877	34.139	-0.653502788	0.38521939	-1.696443128	0.089801992
324.921	34.963	-0.662402299	0.362015732	-1.829761084	0.067285672
425.291	227.677	0.189310628	0.095817961	1.975732168	0.04818512
566.582	32.607	-1.138976291	0.580968918	-1.960477154	0.049940046
89.009	34.171	0.742961029	0.321753841	2.309097625	0.020938165
89.024	31.295	0.806338034	0.333733706	2.416112066	0.015687232
89.04	33.444	0.785539071	0.322396508	2.436561969	0.014827629

Second validation

Mass to charge ratio	Retention Time	beta	se	zvalue	pvalue
113.024	36.458	0.949770899	0.261182532	3.636425805	0.000276447

335.094	86.335	0.717553964	0.192048961	3.736307455	0.000186742
362.062	90.079	0.165716949	0.071957046	2.302998215	0.021278944
373.057	87.578	-0.33608308	0.113319707	-2.965795519	0.003019011
382.766	68.554	-1.31819826	0.777096763	-1.696311608	0.089826883
482.36	49.589	-0.956822052	0.290709883	-3.291329632	0.00099715
496.686	67.924	-1.956724798	0.728118301	-2.687372087	0.007201668
496.84	72.752	0.354274728	0.18595791	1.905133953	0.056762661
508.711	67.545	-1.537332903	0.832413831	-1.846837289	0.064770726
572.662	68.132	-0.143997922	0.074090213	-1.943548492	0.051949922
604.571	68.42	-0.952890891	0.546424203	-1.743866551	0.081182362
606.568	68.596	-1.009163495	0.533160066	-1.892796479	0.05838494
608.565	68.642	-1.063997485	0.57043888	-1.865226094	0.062149719
614.6	67.891	-1.278072265	0.736734723	-1.734779459	0.082779876
662.53	68.617	-0.986643389	0.51139999	-1.929298806	0.053693778
664.526	68.434	-0.976882992	0.507914027	-1.923323514	0.054439433
666.523	68.093	-1.146264473	0.536857594	-2.135136928	0.032749827
668.521	68.55	-1.287975129	0.548122414	-2.349794673	0.01878377
672.558	68.111	-1.217267615	0.595101515	-2.045478938	0.040807664
744.59	42.524	-0.645095637	0.18678921	-3.453602266	0.000553153
745.593	42.325	-0.182931507	0.109265522	-1.674192401	0.094092792

Second validation

Mass to charge ratio	Retention Time	beta	se	zvalue	pvalue
1034.331	67.999	-0.493676551	0.293387556	-1.6826772	0.092437598
1037.653	51.282	-0.290041155	0.098394245	-2.947745107	0.003201009
113.071	101.512	-0.617984212	0.338196284	-1.827294505	0.067655501
130.136	49.642	0.374054963	0.086229099	4.337920325	1.44E-05
146.118	59.646	-1.090324294	0.448256682	-2.43236596	0.015000543
160.133	55.358	1.279617698	0.315191103	4.059815416	4.91E-05
165.013	74.843	0.925222474	0.321675329	2.876261842	0.004024157
190.119	93.248	0.883071243	0.281746796	3.134272531	0.001722808
198.085	100.113	-0.508059404	0.304327053	-1.669451991	0.095027837
203.053	75.296	1.165982669	0.267007883	4.366847355	1.26E-05
204.056	74.501	1.223614154	0.274302133	4.460826251	8.16E-06
220.067	99.688	-0.473793067	0.259468057	-1.826016941	0.06784771
221.07	102.078	-0.232726466	0.1233543	-1.886650618	0.059207334
221.988	85.683	-1.109267531	0.353556075	-3.137458555	0.001704194
225.034	75.303	0.899909875	0.307499023	2.926545473	0.003427494
226.038	75.461	0.408670549	0.180290427	2.266734604	0.023406443
231.055	75.316	-0.879007453	0.333026417	-2.639452628	0.008304003
257.147	74.625	-0.443099077	0.197717643	-2.241069995	0.025021541
258.15	74.904	-0.216985146	0.080056733	-2.710392204	0.006720369
313.154	42.894	-0.266435205	0.112971567	-2.358427093	0.018352564
314.158	42.873	-0.285172197	0.13093302	-2.1780006	0.029405992
326.987	76.127	-0.250003421	0.144510931	-1.729996606	0.083630882

143.035	36.297	0.962350611	0.271842329	3.540105821	0.000399967
145.062	30.621	-0.766169517	0.292859352	-2.616168855	0.008892257
161.987	32.772	0.06068801	0.03036887	1.998362457	0.045677379
177.04	33.332	0.480821939	0.206473177	2.328738033	0.019872948
179.056	36.803	0.721334479	0.252554659	2.856151937	0.004288099
180.06	36.11	0.666895085	0.238169738	2.800083215	0.005108943
195.051	31.406	0.629373107	0.196867535	3.196936995	0.001388953
199.038	31.744	-1.190539071	0.233493257	-5.098815651	3.42E-07
200.041	32.191	-0.218852254	0.048782946	-4.486245094	7.25E-06
209.067	36.808	-1.33850727	0.253793012	-5.274011522	1.33E-07
215.033	30.913	0.926289037	0.273016907	3.392790016	0.000691847
215.091	31.459	1.002899364	0.286790301	3.496977964	0.000470561
216.035	31.278	0.875489216	0.270235182	3.239730697	0.001196426
217.03	30.51	0.932044637	0.274669149	3.393335734	0.00069047
218.033	30.739	0.930051126	0.275492276	3.375960801	0.000735584
223.082	38.976	-0.728174145	0.174722568	-4.167602118	3.08E-05
225.062	37.895	1.047938816	0.281032646	3.72888641	0.000192328
226.065	36.217	0.443028927	0.146119243	3.031968407	0.002429646
260.023	28.879	0.253750367	0.119509626	2.123262994	0.03373182
269.088	35.076	0.782517189	0.218511972	3.58111815	0.000342127
280.622	30.778	-0.168089865	0.050064368	-3.357475	0.000786579
425.291	227.677	0.238655474	0.067845266	3.517643697	0.000435397

Third validation

Mass to charge ratio	Retention Time	beta	se	zvalue	pvalue
113.024	36.458	1.553363451	0.437155145	3.553345917	0.000380364
143.035	36.297	1.481824327	0.437027589	3.390688286	0.000697173
145.062	30.621	-1.235117453	0.418208165	-2.953355661	0.003143396
158.061	87.22	-0.890156659	0.40668252	-2.188824489	0.028609599
174.088	30.043	-0.800099146	0.276060364	-2.898276069	0.003752201
177.04	33.332	0.911616799	0.299782547	3.040926863	0.002358511
179.056	36.803	1.461071123	0.430567215	3.393363623	0.000690399
180.06	36.11	1.054417439	0.402255439	2.621263347	0.008760456
195.051	31.406	0.909736797	0.302153791	3.010840253	0.002605259
199.038	31.744	-0.848208835	0.221862824	-3.823122862	0.000131772
200.041	32.191	-0.178325898	0.05366053	-3.323222798	0.000889838
209.067	36.808	-1.033786847	0.251373388	-4.112554856	3.91E-05
215.033	30.913	1.325947212	0.423565799	3.130439748	0.001745448
215.091	31.459	1.246944519	0.428285349	2.911480679	0.003597202
216.035	31.278	1.368573419	0.431949106	3.168367294	0.001532977
217.03	30.51	1.363689344	0.431329953	3.161592039	0.001569092
218.033	30.739	1.375003995	0.434976024	3.161102953	0.001571729
223.082	38.976	-0.68672966	0.211053544	-3.253817245	0.001138655
225.062	37.895	1.437485938	0.444213373	3.236025802	0.001212064
226.065	36.217	1.089276834	0.300132785	3.629316379	0.000284173

335.094	86.335	1.588206824	0.300149385	5.291387907	1.21E-07
360.15	108.476	0.284657864	0.153042959	1.859986679	0.062887411
363.017	77.333	-0.200832541	0.119985706	-1.673803888	0.094169148
402.083	87.844	-0.140182305	0.065540552	-2.138863657	0.032446712
744.59	42.524	-0.505880157	0.185725426	-2.723806692	0.006453427

239.077	35.736	0.693690292	0.418244897	1.658574432	0.097201577
269.088	35.076	0.962210137	0.274739175	3.502267699	0.000461316
311.14	35.236	-0.397124571	0.125571979	-3.162525373	0.001564071
324.921	34.963	-0.82844595	0.344037059	-2.408013697	0.016039579
401.291	235.525	0.699021718	0.256477343	2.725471613	0.00642097
415.306	235.89	0.705186537	0.250340531	2.816909165	0.004848824
425.291	227.677	0.298185103	0.09195653	3.242674588	0.001184134
89.009	34.171	0.49404225	0.299138695	1.651549124	0.098626499
89.024	31.295	0.63604599	0.314742537	2.020845342	0.043295777
89.04	33.444	0.553147181	0.299791901	1.845103816	0.065022438
90.028	31.729	0.498961064	0.302817641	1.647727859	0.099408529

Third validation

Mass to charge ratio	Retention Time	beta	se	zvalue	pvalue
1037.653	51.282	-0.63167556	0.173465728	-3.64150064	0.000271053
106.999	75.195	-0.764467581	0.35718409	-2.14026213	0.032333589
113.071	101.512	-1.196782186	0.392822006	-3.046627146	0.002314246
130.123	27.204	0.527666047	0.19105897	2.761796776	0.005748425
130.136	49.642	0.471416506	0.100302494	4.699948003	2.60E-06
146.118	59.646	-0.85940412	0.512635442	-1.676443043	0.093651444
159.076	102.224	-1.174657996	0.406221187	-2.891670928	0.003831991
160.08	100.482	-1.047180888	0.354785311	-2.951590316	0.003161421
160.133	55.358	0.758092131	0.311927566	2.430346704	0.015084386
165.013	74.843	0.650412822	0.34954144	1.860760263	0.062778039
165.033	87.089	-0.635756949	0.317343611	-2.003370881	0.045137495
170.055	95.669	-0.997991263	0.523035982	-1.908073816	0.056381676
176.103	102.372	-1.020407959	0.407831393	-2.502033874	0.012348211
177.106	100.743	-1.044867615	0.384500033	-2.717470805	0.006578296
190.119	93.248	0.761692329	0.225296989	3.380836702	0.000722655
198.085	100.113	-1.077650403	0.343288096	-3.139201198	0.001694091
199.088	100.117	-0.881665484	0.314853388	-2.800241379	0.005106441
203.053	75.296	0.961868549	0.336702165	2.856734079	0.004280243
204.056	74.501	0.901985139	0.329285066	2.739222731	0.006158464
205.068	73.03	0.197824693	0.092405258	2.140838062	0.032287099
219.026	74.325	0.253375838	0.139395186	1.817679975	0.06911306
221.988	85.683	-1.256460916	0.411281905	-3.054987102	0.002250702
225.034	75.303	0.732831341	0.38551394	1.900920475	0.05731243
231.055	75.316	-1.229423351	0.446111886	-2.755863246	0.005853747
244.079	71.325	0.737344189	0.342209689	2.154656088	0.031188756
257.147	74.625	-0.49654961	0.20782991	-2.389211492	0.016884579
258.15	74.904	-0.169819319	0.086260482	-1.968680385	0.048989805
313.154	42.894	-0.534419502	0.174266187	-3.066684998	0.002164468
315.161	42.821	-0.129145475	0.069299326	-1.863589174	0.062379419
322.81	69.374	-1.610466376	0.828838935	-1.943039001	0.052011446
326.805	68.896	-1.639459794	0.8442942	-1.941811034	0.052159981
331.092	96.557	-0.373078003	0.211590881	-1.763204548	0.077865997
335.094	86.335	1.166336958	0.281534939	4.14277874	3.43E-05
402.083	87.844	-0.126995509	0.067690705	-1.876114428	0.060639569
496.686	67.924	-1.088017119	0.521869326	-2.084845888	0.037083292
504.871	71.618	0.236142798	0.139816779	1.688944625	0.091230042
608.565	68.642	-1.062045679	0.555074243	-1.913339868	0.055704557

614.6	67.891	-1.175250973	0.574804703	-2.044609181	0.040893404
666.523	68.093	-1.086607332	0.547744631	-1.983784543	0.047279864
668.521	68.55	-1.057015365	0.512394115	-2.06289521	0.039122585
722.486	66.165	-0.934412371	0.549562414	-1.700284348	0.089077453
744.59	42.524	-0.551497889	0.177389279	-3.108969666	0.00187741

Supplementary Table 11. Replicated metabolic feature from each cross-validation in HILIC and C18 column.

Non-PD Participants

HILIC

First validation

Mass to charge ratio	Retention Time	beta	se	zvalue	pvalue
104.992	76.36	2.547162348	0.90022181	2.829483045	0.004662327
116.992	75.398	1.07315941	0.333485535	3.218008869	0.001290838
203.053	75.296	1.608572514	0.523898999	3.070386694	0.002137818
204.056	74.501	1.582541036	0.523023559	3.025754784	0.002480134
219.026	74.325	0.894593591	0.311818374	2.868957274	0.004118274
225.034	75.303	2.150626336	0.74150866	2.900338799	0.003727595
226.038	75.461	1.146119228	0.430435457	2.662697064	0.007751717
244.079	71.325	1.585627257	0.554159948	2.861316956	0.00421885
271.04	74.974	1.113003697	0.361920016	3.075275326	0.002103083
293.021	90.496	0.654854348	0.255413795	2.563895764	0.010350462
324.99	75.983	-0.715204129	0.285780955	-2.502630481	0.012327418
326.987	76.127	-0.549350714	0.26673871	-2.059508772	0.039445526
378.925	74.581	1.203506885	0.382828935	3.143719755	0.001668151
382.948	74.924	-0.589231063	0.263427563	-2.236785916	0.025300335
384.945	75.296	-0.576346421	0.263793285	-2.184841136	0.028900497
492.846	74.043	2.357875811	0.728133564	3.23824629	0.001202669
494.843	73.994	1.929101921	0.589379406	3.273107103	0.001063722
504.871	71.618	0.849924752	0.288199443	2.949085341	0.00318716
508.894	71.963	-0.610281088	0.260315403	-2.344390999	0.01905818
552.801	72.015	1.211943354	0.381025889	3.180737556	0.001469006

Second validation

Mass to charge ratio	Retention Time	beta	se	zvalue	pvalue
104.992	76.36	3.019750698	1.124082137	2.686414629	0.00722234
106.999	75.195	-1.111993851	0.554914107	-2.00390265	0.04508049
116.992	75.398	1.744908827	0.601880416	2.899095534	0.003742408
160.133	55.358	1.361784287	0.698406747	1.949844117	0.051194702
165.013	74.843	1.624160445	0.719914064	2.256047667	0.024067646
203.053	75.296	1.353744737	0.515846298	2.624318025	0.008682267
204.056	74.501	1.426636171	0.548158589	2.602597495	0.00925205
207.024	79.355	0.71268176	0.336030524	2.120883994	0.033931566
219.026	74.325	1.252706533	0.445540413	2.811656354	0.004928713
225.034	75.303	2.333451951	0.849244915	2.74767845	0.006001884
226.038	75.461	2.176582968	0.789113996	2.758261771	0.005810964
230.051	75.122	-0.451728464	0.266284508	-1.696412862	0.08980772
231.055	75.316	-1.313698085	0.640776312	-2.050166433	0.040348192
244.079	71.325	1.294365196	0.54948438	2.355599617	0.018492838
271.04	74.974	1.619304657	0.561187165	2.885498385	0.003907945
293.021	90.496	1.244057957	0.453268057	2.7446407	0.006057718
320.967	76.018	1.03975264	0.445822788	2.332210616	0.019689614

C18

First validation

Mass to charge ratio	Retention Time	beta	se	zvalue	pvalue
143.035	36.297	3.815142422	1.214133112	3.142276891	0.001676394
199.038	31.744	-0.674479196	0.364887327	-1.848458817	0.064535997
200.041	32.191	-0.132597902	0.072255039	-1.835137083	0.066485381
209.067	36.808	-0.681162486	0.375070861	-1.816090122	0.069356549
215.033	30.913	4.271324069	1.308035178	3.265450456	0.001092901
217.03	30.51	4.213864733	1.2950899	3.253723723	0.00113903
218.033	30.739	3.952575726	1.244932308	3.174932245	0.001498715
225.062	37.895	3.586716734	1.080992342	3.317985333	0.000906692
359.121	49.326	2.175896447	0.670949379	3.243011341	0.001182735

Second validation

Mass to charge ratio	Retention Time	beta	se	zvalue	pvalue
113.024	36.458	3.125569943	1.211701826	2.579487689	0.009894699
130.059	33.489	-1.187026166	0.710614149	-1.670422925	0.094835717
143.035	36.297	3.732567141	1.583879492	2.356597936	0.018443204
179.056	36.803	4.556570641	1.775220385	2.56676336	0.010265264
180.06	36.11	3.116142123	1.120218884	2.781726115	0.005407065
200.041	32.191	-0.182575196	0.092955727	-1.964109163	0.049517434
209.067	36.808	-1.307332442	0.489610342	-2.670148749	0.007581765
215.033	30.913	3.026339455	1.26333372	2.395518624	0.016596871
215.091	31.459	2.289651333	0.99172421	2.30875813	0.020957007
216.035	31.278	3.025252281	1.304999181	2.318202436	0.020438322
216.971	30.784	1.022389049	0.535747893	1.908339843	0.056347307
217.03	30.51	3.031718197	1.256625675	2.412586546	0.015839776
218.033	30.739	3.027663809	1.248497834	2.425045304	0.015306477
219.034	30.853	1.647274299	0.804169294	2.048417305	0.040519127
225.062	37.895	3.432966213	1.377528533	2.492119859	0.012698318
226.065	36.217	2.025000889	0.77427081	2.615365146	0.008913211
239.077	35.736	2.81813855	1.091999506	2.580714125	0.009859619
241.023	55.244	0.65945687	0.334078153	1.97395988	0.048386305
359.121	49.326	1.450391649	0.555802716	2.609544012	0.009066298

Third validation

Mass to charge ratio	Retention Time	beta	se	zvalue	pvalue
113.024	36.458	1.332546064	0.655209055	2.033772355	0.041974553
143.035	36.297	1.708037588	0.714967093	2.38897371	0.016895511
179.056	36.803	1.175889577	0.63888873	1.840523274	0.06569145
180.06	36.11	1.161509556	0.628719807	1.847420016	0.06468629
215.033	30.913	1.827284145	0.718360251	2.543687716	0.01096891
215.091	31.459	1.567370683	0.701383944	2.234682867	0.025438176
216.035	31.278	1.61323117	0.69390953	2.324843657	0.020080322

324.99	75.983	-0.620970906	0.278010379	-2.233624902	0.025507764
326.987	76.127	-0.674160475	0.300794798	-2.241263743	0.025008996
376.928	74.723	0.805086446	0.388439177	2.072619071	0.038207745
378.925	74.581	1.074210543	0.447161608	2.402287055	0.016292918
382.948	74.924	-0.722885134	0.30622079	-2.360666411	0.018242132
384.945	75.296	-0.580488625	0.275294152	-2.10861226	0.034978064
400.918	71.845	-0.710492428	0.390099636	-1.821310155	0.068559724
492.846	74.043	1.865203584	0.758630015	2.458647228	0.013946158
494.843	73.994	1.486858727	0.568693355	2.614517497	0.008935358
502.874	72.075	0.958681561	0.461163878	2.078830554	0.037632928
504.871	71.618	1.305829404	0.489717673	2.666494342	0.00766469
508.894	71.963	-0.603587254	0.28235238	-2.137709108	0.03254036
552.801	72.015	0.794267079	0.439603285	1.806781493	0.070796355

216.971	30.784	1.032939679	0.465684582	2.218110109	0.026547326
217.03	30.51	1.812980218	0.717717297	2.526036682	0.011535744
218.033	30.739	1.79088189	0.722378222	2.479147123	0.013169697
219.034	30.853	1.260227106	0.504170181	2.499606589	0.012433129
225.062	37.895	1.646849143	0.708287481	2.325114007	0.020065865
226.065	36.217	0.807822704	0.432981744	1.86572001	0.062080549
239.077	35.736	2.096059677	0.863238286	2.428135673	0.015176665
265.002	30.312	-0.721127421	0.282841693	-2.549579637	0.010785288
269.088	35.076	0.74000321	0.442378676	1.672782281	0.094370164
359.121	49.326	0.875624832	0.374557116	2.337760506	0.019399675

Third validation

Mass to charge ratio	Retention Time	beta	se	zvalue	pvalue
104.992	76.36	2.927353753	0.907579067	3.225453143	0.001257734
116.992	75.398	0.88067015	0.310218031	2.838874793	0.004527292
203.053	75.296	1.850912287	0.547119419	3.383013329	0.000716952
204.056	74.501	1.827130635	0.546588836	3.342788062	0.000829412
207.024	79.355	0.996469322	0.329303931	3.025986715	0.002478232
219.026	74.325	0.724874192	0.268352917	2.701197367	0.006909033
225.034	75.303	2.719862903	0.802597321	3.388826293	0.000701925
226.038	75.461	1.317121809	0.421520045	3.124695551	0.001779892
231.055	75.316	-1.701995154	0.652855449	-2.607001531	0.009133895
244.079	71.325	2.484349852	0.737291602	3.369562118	0.000752877
271.04	74.974	1.157324446	0.341301638	3.39091384	0.0006966
293.021	90.496	1.317497708	0.42845715	3.074981262	0.002105158
318.97	76.169	0.905106519	0.339812761	2.663544821	0.007732211
320.967	76.018	0.828934805	0.339574989	2.441094992	0.014642803
376.928	74.723	0.914087889	0.325975312	2.804162936	0.00504474
378.925	74.581	0.961869484	0.347129806	2.770921616	0.005589788
492.846	74.043	1.15287964	0.442997006	2.602454697	0.009255904
494.843	73.994	1.123761804	0.411198159	2.732896001	0.006278015
496.331	49.015	-0.260231774	0.149431306	-1.741480959	0.081599308
496.84	72.752	1.058799726	0.408104693	2.594431634	0.009474745
502.874	72.075	0.797588675	0.341412011	2.336147083	0.019483577
504.871	71.618	1.122423657	0.393887237	2.849606563	0.004377334
552.801	72.015	1.377985684	0.450625722	3.057938367	0.002228654

Supplementary Table 12. Sensitivity analysis of additionally adjusting for PD medication use among PD patients.

				HILIC			
mz	rt	beta	se	zvalue	pvalue	Full MWAS FDR p value	Replication FDR p value
130.136	49.642	1.016482785	0.132491454	7.672063031	1.69E-14	4.9303E-11	3.24963E-12
335.094	86.335	0.92734373	0.127185162	7.291288671	3.07004E-13	4.47152E-10	2.94724E-11
204.056	74.501	0.671948466	0.110806219	6.064176448	1.32631E-09	1.28785E-06	8.48842E-08
203.053	75.296	0.653104363	0.110311374	5.920553266	3.2086E-09	2.33667E-06	1.54013E-07
744.59	42.524	-0.687284882	0.13154293	-5.224795275	1.74348E-07	0.000101575	6.69496E-06
1037.653	51.282	-0.620481222	0.121227935	-5.118302316	3.08E-07	1.40E-04	9.21E-06
160.133	55.358	0.615852517	0.121205153	5.081075347	3.75E-07	0.000139712	9.20864E-06
221.988	85.683	-0.619345251	0.121993402	-5.076874972	3.83693E-07	0.000139712	9.20864E-06
190.119	93.248	0.561348693	0.117779541	4.766096797	1.87829E-06	0.00060794	4.00702E-05
219.026	74.325	0.585399344	0.123983427	4.721593506	2.34004E-06	0.000619685	4.08443E-05
313.154	42.894	-0.550703478	0.116259188	-4.736859831	2.17055E-06	0.000619685	4.08443E-05
231.055	75.316	-0.556882175	0.118678113	-4.692374717	2.70052E-06	0.000655551	4.32083E-05
244.079	71.325	0.515824917	0.111940992	4.608007371	4.06546E-06	0.000910976	6.00438E-05
496.686	67.924	-0.598130851	0.130966909	-4.567038016	4.94664E-06	0.001029255	6.78397E-05
165.013	74.843	0.546261471	0.120223495	4.543716441	5.53E-06	0.00106043	6.98945E-05
198.085	100.113	-0.5446339	0.120157665	-4.532660489	5.82454E-06	0.00106043	6.98945E-05
668.521	68.55	-0.5677292	0.126955107	-4.471889404	7.75315E-06	0.001328525	8.7565E-05
373.057	87.578	-0.472036281	0.106125355	-4.44791237	8.67089E-06	0.001403239	9.24895E-05
225.034	75.303	0.509037916	0.116191437	4.381027798	1.18121E-05	0.001783648	0.000117563
258.15	74.904	-0.488781912	0.111768601	-4.373159448	1.22461E-05	0.001783648	0.000117563
199.088	100.117	-0.512405899	0.117872479	-4.347120764	1.37936E-05	0.001913373	0.000126113
165.033	87.089	-0.511285216	0.119195182	-4.289478887	1.79E-05	0.002371352	0.000156299
130.123	27.204	0.480487222	0.112786783	4.260137684	2.04E-05	0.00257524	0.000169738
159.076	102.224	-0.525548247	0.123609529	-4.251680695	2.12E-05	0.00257524	0.000169738
113.071	101.512	-0.506835539	0.120256743	-4.21461221	2.50E-05	0.00262275	0.000172869
160.08	100.482	-0.513870567	0.121861062	-4.216856149	2.48E-05	0.00262275	0.000172869
220.067	99.688	-0.486735028	0.114965609	-4.233744623	2.29832E-05	0.00262275	0.000172869
257.147	74.625	-0.52724491	0.125149799	-4.212910569	2.52101E-05	0.00262275	0.000172869
146.118	59.646	-0.542662247	0.130641947	-4.153813215	3.27E-05	0.003072558	0.000202517
177.106	100.743	-0.506990521	0.121782033	-4.163097875	3.13959E-05	0.003072558	0.000202517
214.18	26.723	-0.421331811	0.101297472	-4.15935168	3.19152E-05	0.003072558	0.000202517
326.987	76.127	-0.479990265	0.116218312	-4.130074306	3.62646E-05	0.003301212	0.000217588
226.038	75.461	0.628411484	0.15391243	4.082915734	4.44742E-05	0.003654254	0.000240857
482.36	49.589	-0.53962986	0.13176824	-4.095295349	4.21631E-05	0.003654254	0.000240857
666.523	68.093	-0.512925486	0.125517483	-4.086486384	4.37955E-05	0.003654254	0.000240857
670.518	68.336	-0.460949804	0.11299574	-4.079355601	4.51607E-05	0.003654254	0.000240857
104.992	76.36	0.453880761	0.111545848	4.069006329	4.72E-05	0.003677122	0.000242364
106.999	75.195	-0.475146168	0.117409583	-4.046911305	5.19E-05	0.003677122	0.000242364
170.055	95.669	-0.575724694	0.14181109	-4.059800207	4.91E-05	0.003677122	0.000242364
176.06	99.313	-0.451720817	0.111633998	-4.046444863	5.20E-05	0.003677122	0.000242364
314.158	42.873	-0.465264145	0.114856215	-4.050839949	5.10341E-05	0.003677122	0.000242364

402.083	87.844	-0.451843601	0.111789578	-4.041911666	5.30172E-05	0.003677122	0.000242364
288.119	128.322	0.627072792	0.15619055	4.014793412	5.94979E-05	0.003961494	0.000261108
664.526	68.434	-0.495357646	0.123424337	-4.013451954	5.98372E-05	0.003961494	0.000261108
117.077	78.853	0.459818023	0.115173939	3.992379069	6.54E-05	0.004142393	0.000273031
205.068	73.03	0.554168233	0.138782611	3.993066782	6.52242E-05	0.004142393	0.000273031
745.593	42.325	-0.435908899	0.109349864	-3.986368926	6.70922E-05	0.004158287	0.000274079
221.07	102.078	-0.431022336	0.108772903	-3.962589258	7.41413E-05	0.004499449	0.000296565
131.111	109.554	-0.45498585	0.11544511	-3.941144406	8.11E-05	0.004724524	0.0003114
614.6	67.891	-0.498455054	0.126321166	-3.945934547	7.94893E-05	0.004724524	0.0003114
1038.657	51.615	-0.383305208	0.097719179	-3.9225177	8.76E-05	0.004727068	0.000311568
1152.248	67.801	-0.393967655	0.10034981	-3.925943206	8.64E-05	0.004727068	0.000311568
322.81	69.374	-0.496367422	0.126180293	-3.933795135	8.36151E-05	0.004727068	0.000311568
362.062	90.079	0.456430168	0.116152105	3.929590172	8.50908E-05	0.004727068	0.000311568
176.103	102.372	-0.486319162	0.124186869	-3.916027238	9.00E-05	0.004767791	0.000314252
326.805	68.896	-0.493601642	0.126355499	-3.906451616	9.36614E-05	0.004872065	0.000321125
1162.277	67.73	-0.476391528	0.122693742	-3.882769578	1.03E-04	0.005277815	0.000347868
261.043	87.544	-0.425692922	0.110477842	-3.853197307	0.000116585	0.005567429	0.000366957
331.092	96.557	-0.408532367	0.105811584	-3.860941788	0.000112951	0.005567429	0.000366957
608.565	68.642	-0.499805737	0.129368189	-3.863436131	0.000111803	0.005567429	0.000366957
722.486	66.165	-0.492025532	0.12766209	-3.854124071	0.000116145	0.005567429	0.000366957
250.064	59.738	-0.485747965	0.126382553	-3.843473272	0.000121305	0.005608922	0.000369692
546.83	77.148	-0.451783942	0.117443909	-3.846806056	0.000119668	0.005608922	0.000369692
315.161	42.821	-0.437789085	0.114081577	-3.837509054	0.000124289	0.005641298	0.000371826
360.15	108.476	0.481528048	0.125581543	3.834385491	0.000125879	0.005641298	0.000371826
169.059	96.539	-0.459044632	0.120276272	-3.816585144	1.35E-04	0.005972153	0.000393633
572.662	68.132	-0.436644584	0.114753533	-3.805064417	0.000141767	0.006163703	0.000406259
101.071	96.528	-0.438920997	0.115599473	-3.796911762	1.47E-04	6.24E-03	4.11E-04
520.12	55.829	-0.407601797	0.107409808	-3.794828459	0.000147746	0.006237433	0.000411118
146.6	96.634	-0.419531125	0.111235537	-3.771556626	1.62E-04	0.006656096	0.000438713
147.555	96.563	-0.430870006	0.114167616	-3.77401248	1.61E-04	0.006656096	0.000438713
672.558	68.111	-0.451674653	0.12110128	-3.729726492	0.000191688	0.007755366	0.000511167
149.077	96.666	-0.388244715	0.104504852	-3.71508793	2.03E-04	0.008105843	0.000534268
148.08	94.797	-0.435071848	0.117593392	-3.699798435	2.16E-04	0.008493787	0.000559838
329.03	87.116	-0.420154276	0.113694565	-3.695464921	0.000219485	0.008524789	0.000561881
542.153	102.319	-0.403311879	0.109350123	-3.688261762	0.000225791	0.008654343	0.00057042
276.119	116.59	-0.414889568	0.113781172	-3.646381572	0.000265959	0.009606726	0.000633193
476.306	44.819	0.435834766	0.119491402	3.647415276	0.000264892	0.009606726	0.000633193
504.871	71.618	0.448826118	0.12282677	3.654139215	0.000258046	0.009606726	0.000633193
508.711	67.545	-0.437746675	0.120086757	-3.645253529	0.000267128	0.009606726	0.000633193
90.055	84.601	0.428948162	0.117364778	3.654828734	0.000257354	0.009606726	0.000633193
496.84	72.752	0.451523806	0.124791729	3.618219013	0.000296637	0.010410898	0.000686197
662.53	68.617	-0.454153683	0.125454682	-3.620061653	0.000294533	0.010410898	0.000686197
382.766	68.554	-0.457098406	0.126516463	-3.61295592	0.000302726	0.010498112	0.000691946
161.137	55	0.452423419	0.125625742	3.601359187	3.17E-04	0.010848624	0.000715048

438.727	68.044	-0.418545368	0.116973671	-3.578116039	0.00034608	0.011722447	0.000772643
349.064	96.841	-0.383671652	0.10734471	-3.574201769	0.000351298	0.011762425	0.000775278
363.017	77.333	-0.353903833	0.09929129	-3.564298878	0.00036483	0.011941011	0.000787049
556.859	81.955	-0.411327663	0.115339812	-3.566224483	0.000362161	0.011941011	0.000787049
312.782	71.919	-0.460269338	0.129298573	-3.559740283	0.000371222	0.01201521	0.00079194
148.06	89.479	0.407720774	0.115385405	3.533555857	4.10E-04	0.012036399	0.000793336
149.081	97.299	-0.411672728	0.116571341	-3.531508888	4.13E-04	0.012036399	0.000793336
159.092	58.364	-0.412303854	0.116338508	-3.544001571	3.94E-04	0.012036399	0.000793336
173.092	90.691	0.405365521	0.114696817	3.534235138	4.09E-04	0.012036399	0.000793336
216.063	122.209	0.463179887	0.130480142	3.549811334	0.000385507	0.012036399	0.000793336
254.824	72.613	-0.445205176	0.125490015	-3.547733853	0.000388561	0.012036399	0.000793336
388.254	41.378	0.398599425	0.112165881	3.553660169	0.00037991	0.012036399	0.000793336
568.426	36.345	-0.437041945	0.123673535	-3.533835645	0.000409576	0.012036399	0.000793336
604.571	68.42	-0.450726663	0.127480225	-3.535659452	0.000406759	0.012036399	0.000793336
606.568	68.596	-0.451621754	0.12760113	-3.539324099	0.000401153	0.012036399	0.000793336
130.05	94.746	-0.425003491	0.120465849	-3.527999787	4.19E-04	0.012076332	0.000795968
130.077	96.602	-0.418215334	0.119420373	-3.502043428	4.62E-04	0.013057716	0.000860653
151.144	58.911	0.474848035	0.135551575	3.503080185	4.60E-04	0.013057716	0.000860653
238.107	39.303	0.375187418	0.107229551	3.498918109	0.00046715	0.013084693	0.000862431
343.123	109.031	0.432333268	0.123692837	3.495216692	0.000473677	0.013141154	0.000866152
207.024	79.355	0.515393017	0.14820556	3.477555209	0.000506009	0.01384095	0.000912277
307.019	63.703	-0.398941894	0.114760857	-3.476288921	0.000508404	0.01384095	0.000912277
551.425	36.279	-0.402950831	0.116147447	-3.469304257	0.000521808	0.014074325	0.000927659
316.776	71.543	-0.450157904	0.130019682	-3.46222892	0.000535721	0.014186874	0.000935077
369.894	67.014	-0.350745277	0.101259745	-3.463817511	0.000532568	0.014186874	0.000935077
232.027	52.237	0.361546509	0.104672136	3.454085522	0.000552162	0.014234059	0.000938187
281.008	53.407	-0.390104126	0.11290997	-3.455001592	0.00055029	0.014234059	0.000938187
314.779	72.2	-0.445454086	0.128853094	-3.457069383	0.000546084	0.014234059	0.000938187
202.119	68.492	0.491285562	0.143181971	3.431197095	0.000600924	0.015355185	0.001012082
386.76	68.644	-0.391586265	0.114352589	-3.42437602	0.000616213	0.015608942	0.001028808
174.05	76.594	-0.372749803	0.109029512	-3.4187973	6.29E-04	0.015795131	0.00104108
159.084	121.116	0.436105283	0.127917028	3.409282476	6.51E-04	0.015944146	0.001050901
245.049	81.304	0.397416882	0.116498144	3.411358065	0.000646401	0.015944146	0.001050901
271.04	74.974	0.429941138	0.125949121	3.413609664	0.000641084	0.015944146	0.001050901
298.101	114.873	-0.360961018	0.106819142	-3.379179146	0.000727026	0.01764856	0.001163242
1041.685	49.43	-0.419209962	0.124911866	-3.356045954	7.91E-04	0.018759589	0.001236471
127.03	48.917	-0.33164671	0.098835699	-3.355535643	7.92E-04	0.018759589	0.001236471
486.656	68.489	-0.39236236	0.116921378	-3.355779467	0.000791417	0.018759589	0.001236471
147.076	98.78	-0.396299107	0.118250517	-3.35135199	8.04E-04	0.018891748	0.001245182
212.101	91.485	0.446612062	0.133442391	3.346852966	0.000817346	0.01889625	0.001245479
566.67	67.811	-0.398000699	0.118841019	-3.349017907	0.000810986	0.01889625	0.001245479
151.048	94.49	-0.398685206	0.119413522	-3.338693964	8.42E-04	0.019306823	0.00127254
1034.331	67.999	-0.39496729	0.11873352	-3.326501978	8.79E-04	2.00E-02	1.32E-03
430.696	70.699	-0.423658846	0.12758031	-3.320722809	0.000897847	0.020274629	0.00133633

324.99	75.983	-0.386000102	0.116591603	-3.310702431	0.000930621	0.020853072	0.001374456
177.1	100.826	-0.325224194	0.098445373	-3.303600602	9.55E-04	0.020906079	0.00137795
189.123	85.138	0.364003234	0.110060027	3.307315508	0.000941947	0.020906079	0.00137795
223.985	85.258	-0.367354123	0.111188982	-3.303871635	0.000953595	0.020906079	0.00137795
480.345	47	-0.419587575	0.127558387	-3.289376619	0.001004096	0.021827841	0.001438704
164.029	86.645	-0.392162584	0.119784652	-3.273896774	1.06E-03	0.022888704	0.001508627
138.013	74.729	-0.343265255	0.105347076	-3.25842223	1.12E-03	0.023996604	0.001581651
193.028	89.085	0.390040887	0.119849324	3.254427087	0.001136213	0.024159045	0.001592357
130.041	96.347	-0.340315147	0.104774753	-3.248064428	1.16E-03	0.024526818	0.001616598
1128.196	68.006	-0.33428962	0.103002052	-3.245465629	1.17E-03	0.024573716	0.001619689
301.118	42.143	-0.36067369	0.111433387	-3.236675275	0.001209309	0.025162273	0.001658481
163.06	105.755	0.376333431	0.116612313	3.227218656	1.25E-03	0.025824447	0.001702126
570.665	67.778	-0.391643771	0.121495347	-3.223528972	0.001266215	0.025975239	0.001712065
318.97	76.169	0.380302869	0.11810767	3.219967586	0.001282051	0.026116184	0.001721355
740.546	67.144	-0.360635212	0.112113782	-3.21668938	0.001296789	0.026232964	0.001729052
483.364	49.807	-0.350429507	0.109060546	-3.213164803	0.001312809	0.026373887	0.001738341
428.698	68.62	-0.409130604	0.127756957	-3.202413505	0.001362812	0.027190904	0.001792191
365.152	102.353	-0.31116516	0.097524691	-3.190629529	0.001419632	0.028013784	0.001846429
98.058	59.679	0.44841853	0.140575091	3.189886112	0.001423289	0.028013784	0.001846429
131.053	97.331	-0.378156389	0.11902783	-3.177041778	1.49E-03	0.02901264	0.001912265
206.094	58.321	-0.382218151	0.120424377	-3.173926736	0.001503916	0.02901264	0.001912265
214.023	130.222	-0.329789762	0.10390476	-3.173962039	0.001503734	0.02901264	0.001912265
335.84	67.832	-0.370862712	0.117014821	-3.169365281	0.001527723	0.029278	0.001929755
252.108	109.021	0.393058591	0.124318296	3.161711547	0.001568448	0.029862029	0.001968249
444.719	68.358	-0.356699612	0.1129014	-3.159390503	0.001580995	0.029905444	0.001971111
346.862	70.229	-0.307326402	0.097944117	-3.137772971	0.001702367	0.031788426	0.002095221
358.057	88.539	-0.330859075	0.10540143	-3.139037823	0.001695036	0.031788426	0.002095221
210.888	69.976	-0.385540023	0.123018792	-3.133992927	0.00172445	0.031995689	0.002108882
1047.732	48.709	-0.36388195	0.116704201	-3.11798503	1.82E-03	0.033571781	0.002212764
246.955	138.586	-0.339199032	0.109509446	-3.097440865	0.001951993	0.035761992	0.002357124
374.734	70.26	-0.387254445	0.125288919	-3.09089142	0.001995566	0.036106109	0.002379805
85.052	96.569	-0.315648058	0.102104119	-3.091433131	0.001991928	0.036106109	0.002379805
205.043	58.162	-0.307404408	0.099573572	-3.087208809	0.002020456	0.036330795	0.002394615
150.084	96.708	-0.310602159	0.100866862	-3.079328063	2.07E-03	0.037076965	0.002443796
188.071	59.806	-0.378368487	0.123131644	-3.072877733	0.002120053	0.037304349	0.002458783
247.14	110.194	0.349050562	0.113620766	3.072066607	0.002125823	0.037304349	0.002458783
289.975	84.825	-0.346683823	0.112743664	-3.074973885	0.00210521	0.037304349	0.002458783
1134.187	68.175	-0.342115043	0.112224219	-3.048495646	2.30E-03	0.039878666	0.002644199
265.035	59.429	-0.352993392	0.116070578	-3.041196122	0.002356403	0.040516142	0.002686279
682.586	67.347	-0.349018364	0.114802437	-3.040165118	0.002364485	0.040516142	0.002686279
548.827	78.227	-0.356775249	0.117500696	-3.036367102	0.002394476	0.040790115	0.00270435
121.063	90.503	-0.338154622	0.111583513	-3.030507047	2.44E-03	0.041348255	0.00274126
353.9	79.616	-0.350678874	0.116223908	-3.017269669	0.002550628	0.042947857	0.002847213
249.108	93.074	-0.371051896	0.123165857	-3.012619778	0.002590032	0.043112933	0.002857966

336.834	67.349	-0.360562971	0.119674213	-3.01287104	0.002587889	0.043112933	0.002857966
390.798	67.798	-0.370557538	0.123278989	-3.005845037	0.002648439	0.043704774	0.002905716
372.1	37.886	0.31715236	0.106127865	2.988398564	0.002804436	0.045895064	0.003059384
334.836	67.586	-0.35773961	0.119983847	-2.98156475	0.002867794	0.046669742	0.003110827
120.003	86.774	-0.376855924	0.126745119	-2.973336774	2.95E-03	0.046891506	0.003159752
351.088	56.365	0.301902319	0.101522454	2.973749226	0.002941854	0.046891506	0.003159752
207.897	68.486	-0.368081403	0.124964144	-2.945496122	0.003224372	0.050497824	0.003401535
272.185	35.432	0.326139824	0.110692928	2.94634742	0.00321551	0.050497824	0.003401535
365.105	100.434	0.325626318	0.110507902	2.946633801	0.003212534	0.050497824	0.003401535
277.953	141.319	-0.318834642	0.108442496	-2.940126372	0.003280784	0.051024728	0.003436224
509.379	48.096	-0.372128478	0.126618666	-2.938970138	0.003293048	0.051024728	0.003436224
101.977	134.09	-0.338455896	0.116387222	-2.908015924	3.64E-03	5.41E-02	3.75E-03
199.119	92.991	-0.319701536	0.109925545	-2.908346146	0.003633459	0.054058416	0.00375463
1154.245	67.712	-0.272200304	0.093725081	-2.904241862	3.68E-03	0.054161739	0.003779871
189.074	58.358	-0.353935189	0.121965658	-2.901924976	0.003708773	0.054289735	0.003787684
585.195	102.316	-0.325532949	0.113460169	-2.869138581	0.004115914	0.059062355	0.004181246
238.092	127.057	0.367323878	0.13005681	2.824334047	0.004737898	0.065409933	0.00478777
412.853	73.358	-0.301393519	0.107442869	-2.805151453	0.005029294	0.06807049	0.005055626
1150.25	67.735	-0.266100835	0.095989314	-2.772192265	5.57E-03	0.072156966	0.005568014

Supplementary Table 12. Sensitivity analysis of additionally adjusting for PD medication use among PD patients.

				C18			
mz	rt	beta	se	zvalue	pvalue	Full MWAS FDR p value	Replication FDR p value
209.067	36.808	-0.88277	0.118188	-7.46915	8.07E-14	1.79E-10	1.07E-11
199.038	31.744	-0.83184	0.115571	-7.19766	6.13E-13	6.81E-10	4.07E-11
200.041	32.191	-0.65309	0.095515	-6.8376	8.05E-12	5.96E-09	3.57E-10
223.082	38.976	-0.78044	0.115294	-6.76913	1.30E-11	7.20E-09	4.31E-10
269.088	35.076	0.748142	0.120301	6.218898	5.01E-10	2.22E-07	1.33E-08
143.035	36.297	0.669829	0.112485	5.954853	2.60E-09	9.64E-07	5.77E-08
113.024	36.458	0.653153	0.110166	5.928793	3.05E-09	9.69E-07	5.80E-08
225.062	37.895	0.654682	0.111099	5.892765	3.80E-09	1.05E-06	6.31E-08
215.033	30.913	0.597742	0.110416	5.413558	6.18E-08	1.17E-05	7.02E-07
215.091	31.459	0.597016	0.110372	5.409135	6.33E-08	1.17E-05	7.02E-07
217.03	30.51	0.595638	0.109883	5.420635	5.94E-08	1.17E-05	7.02E-07
218.033	30.739	0.594037	0.10914	5.442905	5.24E-08	1.17E-05	7.02E-07
216.035	31.278	0.576323	0.108543	5.309647	1.10E-07	1.88E-05	1.12E-06
179.056	36.803	0.558534	0.107271	5.20676	1.92E-07	2.86E-05	1.71E-06
195.051	31.406	0.587142	0.112777	5.206211	1.93E-07	2.86E-05	1.71E-06
174.088	30.043	-0.61275	0.118457	-5.17274	2.31E-07	3.20E-05	1.92E-06
425.291	227.677	0.700016	0.135676	5.15947	2.48E-07	3.24E-05	1.94E-06
226.065	36.217	0.582518	0.116346	5.006789	5.53E-07	6.83E-05	4.09E-06
161.987	32.772	0.525857	0.110274	4.768626	1.85E-06	2.06E-04	1.23E-05
280.622	30.778	-0.48138	0.100787	-4.77624	1.79E-06	2.06E-04	1.23E-05
180.06	36.11	0.519777	0.109256	4.757422	1.96E-06	2.07E-04	1.24E-05
145.062	30.621	-0.54075	0.119831	-4.51261	6.40E-06	6.47E-04	3.87E-05
239.077	35.736	0.492832	0.110498	4.460103	8.19E-06	7.91E-04	4.74E-05
177.04	33.332	0.535318	0.121273	4.414166	1.01E-05	9.01E-04	5.39E-05
415.306	235.89	0.594848	0.13454	4.421338	9.81E-06	9.01E-04	5.39E-05
261.041	34.747	-0.43468	0.101041	-4.30201	1.69E-05	1.45E-03	8.66E-05
160.062	29.915	0.471085	0.110797	4.251782	2.12E-05	1.63E-03	9.73E-05
311.14	35.236	-0.42136	0.098993	-4.25646	2.08E-05	1.63E-03	9.73E-05
401.291	235.525	0.551618	0.12933	4.265215	2.00E-05	1.63E-03	9.73E-05
494.81	33.188	-0.40849	0.097226	-4.20147	2.65E-05	0.001964	0.000118
402.293	232.497	0.558466	0.134335	4.157253	3.22E-05	0.002238	0.000134
416.309	235.341	0.563625	0.135582	4.15709	3.22E-05	0.002238	0.000134
89.04	33.444	0.457566	0.11117	4.115929	3.86E-05	0.002597	0.000155
89.024	31.295	0.45241	0.111294	4.064993	4.80E-05	0.003139	0.000188
89.009	34.171	0.442157	0.1111	3.979821	6.90E-05	0.004378	0.000262
90.028	31.729	0.439253	0.111399	3.943042	8.05E-05	0.004966	0.000297
260.023	28.879	0.426363	0.1086	3.926009	8.64E-05	0.005187	0.00031
253.093	34.817	-0.38948	0.09956	-3.91201	9.15E-05	0.0053	0.000317
270.091	34.705	0.547512	0.140096	3.908114	9.30E-05	0.0053	0.000317
213.049	35.964	-0.45427	0.11695	-3.88431	0.000103	0.005701	0.000341
158.061	87.22	-0.48576	0.125368	-3.87463	0.000107	0.005787	0.000346

149.046	31.935	0.446593	0.115737	3.858698	0.000114	0.006031	0.000361
193.123	84.259	-0.43858	0.114806	-3.82015	0.000133	0.006892	0.000413
203.021	32.471	-0.41079	0.108107	-3.79982	0.000145	0.00715	0.000428
496.807	33.723	-0.39153	0.102943	-3.80342	0.000143	0.00715	0.000428
324.921	34.963	-0.41506	0.109702	-3.78356	0.000155	0.007468	0.000447
566.582	32.607	-0.47199	0.125323	-3.76618	0.000166	0.007837	0.000469
503.631	32.36	-0.42233	0.112947	-3.7392	0.000185	0.008546	0.000512
439.306	227.232	0.560537	0.150485	3.724881	0.000195	0.008861	0.00053
103.04	32.211	0.40617	0.109678	3.70331	0.000213	0.009457	0.000566
224.952	56.909	-0.39413	0.108173	-3.64357	0.000269	0.011715	0.000701
216.971	30.784	0.444284	0.123258	3.604505	0.000313	0.013364	0.0008
279.614	29.271	-0.38137	0.106032	-3.59676	0.000322	0.013508	0.000809
424.278	219.234	0.470413	0.131375	3.580698	0.000343	0.014101	0.000844
268.96	35.981	-0.39154	0.109988	-3.55987	0.000371	0.01499	0.000897
276	31.551	0.371154	0.104534	3.550545	0.000384	0.015102	0.000904
312.143	35.633	-0.37278	0.105173	-3.54445	0.000393	0.015102	0.000904
326.087	28.546	0.380019	0.107231	3.543933	0.000394	0.015102	0.000904
229.144	107.471	-0.37053	0.105382	-3.51606	0.000438	0.016495	0.000987
114.056	36.877	0.391973	0.112531	3.483245	0.000495	0.017776	0.001064
256.934	34.638	-0.39151	0.112238	-3.48819	0.000486	0.017776	0.001064
414.204	35.694	-0.44967	0.129108	-3.48291	0.000496	0.017776	0.001064
327.091	28.264	0.369104	0.106257	3.473681	0.000513	0.018107	0.001084
501.634	32.333	-0.36196	0.104366	-3.46816	0.000524	0.018194	0.001089
94.908	31.854	-0.43632	0.127603	-3.41935	0.000628	0.021458	0.001284
373.26	213.573	0.491055	0.144004	3.410007	0.00065	0.021686	0.001298
415.216	36.104	-0.43489	0.127601	-3.40821	0.000654	0.021686	0.001298
322.932	37.944	-0.38606	0.113429	-3.40356	0.000665	0.021734	0.001301
127.051	32.393	-0.40774	0.120108	-3.39474	0.000687	0.022121	0.001324
446.67	32.191	-0.41312	0.122587	-3.36998	0.000752	0.023863	0.001428
281.124	29.849	-0.38323	0.114034	-3.36066	0.000778	0.024329	0.001456
445.988	31.336	-0.38994	0.116161	-3.35686	0.000788	0.024329	0.001456
581.24	42.898	-0.35316	0.105405	-3.35048	0.000807	0.024555	0.00147
148.98	33.213	0.386682	0.115578	3.345641	0.000821	0.02465	0.001475
219.034	30.853	0.370707	0.111291	3.330972	0.000865	0.025303	0.001515
282.975	35.721	-0.33215	0.099627	-3.33396	0.000856	0.025303	0.001515
498.638	32.269	-0.41802	0.125722	-3.32491	0.000884	0.025523	0.001528
94.942	31.882	-0.41088	0.123786	-3.3193	0.000902	0.025708	0.001539
144.97	29.085	-0.40857	0.123302	-3.31355	0.000921	0.02591	0.001551
286.06	27.608	0.50351	0.15228	3.306469	0.000945	0.026242	0.001571
149.061	32.211	-0.38509	0.117196	-3.2859	0.001017	0.02747	0.001644
505.628	32.397	-0.34121	0.103922	-3.28327	0.001026	0.02747	0.001644
508.623	32.501	-0.35723	0.108724	-3.28567	0.001017	0.02747	0.001644
151.007	30.177	0.411738	0.125689	3.275848	0.001053	0.027866	0.001668
387.275	216.137	0.438099	0.134361	3.2606	0.001112	0.029063	0.00174

300.877	34.139	-0.35042	0.107663	-3.25478	0.001135	0.029186	0.001747
415.322	215.543	-0.37697	0.115892	-3.2528	0.001143	0.029186	0.001747
359.121	49.326	0.405175	0.125126	3.238127	0.001203	0.03038	0.001818
506.626	32.343	-0.39396	0.121834	-3.23357	0.001223	0.030522	0.001827
254.982	28.027	0.388434	0.121002	3.210147	0.001327	0.032394	0.001939
397.259	210.47	0.411577	0.128117	3.212506	0.001316	0.032394	0.001939
416.953	38.484	-0.32179	0.100402	-3.20505	0.00135	0.032615	0.001952
188.057	31.438	0.365373	0.114832	3.181807	0.001464	0.034969	0.002093
194.039	27.536	0.373252	0.117456	3.177811	0.001484	0.035077	0.0021
302.874	34.257	-0.33168	0.104507	-3.17378	0.001505	0.035194	0.002107
442.676	32.194	-0.38445	0.121636	-3.16065	0.001574	0.036436	0.002181
423.275	218.074	0.460287	0.146657	3.138524	0.001698	0.038897	0.002328
424.247	181.74	0.411867	0.131952	3.12134	0.0018	0.040819	0.002443
163.077	35.967	-0.32844	0.10539	-3.1164	0.001831	0.040837	0.002444
374.263	214.626	0.403323	0.129467	3.115256	0.001838	0.040837	0.002444
162.056	40.608	-0.39767	0.128669	-3.09062	0.001997	0.041828	0.002504
216.909	24.258	0.398864	0.128928	3.093688	0.001977	0.041828	0.002504
220.049	30.642	-0.37888	0.122284	-3.09837	0.001946	0.041828	0.002504
295.984	32.846	-0.32263	0.104199	-3.0963	0.00196	0.041828	0.002504
390.709	32.22	-0.35205	0.113728	-3.09557	0.001964	0.041828	0.002504
431.244	82.667	0.381443	0.123519	3.08813	0.002014	0.041828	0.002504
440.679	32.243	-0.40061	0.129478	-3.09407	0.001974	0.041828	0.002504
146.065	30.227	-0.34201	0.111104	-3.07829	0.002082	0.042055	0.00254
189.067	63.388	-0.34619	0.112446	-3.07876	0.002079	0.042055	0.00254
564.083	212.912	-0.33698	0.110033	-3.06252	0.002195	0.043936	0.002654
217.048	30.99	0.434941	0.142189	3.058889	0.002222	0.044075	0.002662
221.053	30.668	-0.37192	0.121878	-3.05157	0.002276	0.044764	0.002703
223.05	30.675	-0.36983	0.121969	-3.03215	0.002428	0.046962	0.002836
369.154	47.826	-0.3425	0.112965	-3.03186	0.002431	0.046962	0.002836
195.976	28.201	-0.36688	0.121242	-3.02605	0.002478	0.047055	0.002841
403.296	242.279	0.414204	0.136857	3.026546	0.002474	0.047055	0.002841
229.012	27.859	0.395146	0.131188	3.012065	0.002595	0.048861	0.00295
118.051	31.331	-0.36727	0.122195	-3.00558	0.002651	0.049082	0.002963
225.054	30.806	-0.34445	0.114525	-3.00761	0.002633	0.049082	0.002963
226.018	34.225	-0.35147	0.11739	-2.99404	0.002753	0.050142	0.003051
356.098	28.295	0.332503	0.111581	2.979925	0.002883	0.051665	0.003169
156.878	31.938	-0.36382	0.122289	-2.97512	0.002929	0.051741	0.003173
452.705	33.294	-0.32175	0.108166	-2.97457	0.002934	0.051741	0.003173
418.95	37.272	-0.30361	0.102263	-2.96887	0.002989	0.05205	0.003206
255.63	33.175	-0.29292	0.098949	-2.9603	0.003073	0.05205	0.003213
266.963	36.467	-0.33601	0.113576	-2.95843	0.003092	0.05205	0.003213
412.278	210.722	0.360257	0.121755	2.958856	0.003088	0.05205	0.003213
96.922	31.526	-0.37622	0.127014	-2.962	0.003056	0.05205	0.003213
444.673	32.115	-0.35793	0.12112	-2.95519	0.003125	0.052204	0.003222

254.623	31.407	-0.34649	0.117607	-2.94613	0.003218	0.052572	0.003292
406.925	35.575	-0.28548	0.098686	-2.89284	0.003818	0.059322	0.003876
556.597	32.309	-0.35708	0.126186	-2.82976	0.004658	0.066454	0.004666
562.588	32.428	-0.33647	0.118924	-2.82926	0.004666	0.066454	0.004666

Supplementary Table 13. Sensitivity analysis of additionally adjusting for sample collection year among PD patients.

HILIC

mz	rt	beta	se	zvalue	pvalue	Full MWAS FDR p value	Replication FDR p value
130.136	49.642	1.019145029	0.131450275	7.753084009	8.96872E-15	2.61259E-11	1.722E-12
335.094	86.335	0.932146673	0.126680682	7.358238525	1.86353E-13	2.71423E-10	1.78899E-11
204.056	74.501	0.674038451	0.110964896	6.074339525	1.24499E-09	1.20889E-06	7.96794E-08
203.053	75.296	0.654118524	0.110546574	5.917130669	3.27606E-09	2.38579E-06	1.57251E-07
744.59	42.524	-0.692537916	0.130574174	-5.303789355	1.13423E-07	6.60803E-05	4.35545E-06
1037.653	51.282	-0.62186962	0.12088832	-5.144166271	2.68711E-07	0.000130459	8.59877E-06
221.988	85.683	-0.621529503	0.122295867	-5.082179115	3.73129E-07	0.000155275	1.02344E-05
160.133	55.358	0.600034642	0.120920655	4.962217933	6.96927E-07	0.000253769	1.67263E-05
219.026	74.325	0.594473814	0.124576079	4.771974016	1.82429E-06	0.000590462	3.89182E-05
313.154	42.894	-0.551082442	0.116138342	-4.745051736	2.08453E-06	0.000607224	4.0023E-05
190.119	93.248	0.550627128	0.117894069	4.670524417	3.00432E-06	0.000734085	4.83846E-05
231.055	75.316	-0.555758009	0.119026886	-4.669180468	3.02404E-06	0.000734085	4.83846E-05
244.079	71.325	0.513769797	0.111847249	4.593495139	4.35883E-06	0.000935885	6.16855E-05
668.521	68.55	-0.584555178	0.127439033	-4.586939853	4.4979E-06	0.000935885	6.16855E-05
496.686	67.924	-0.604931204	0.132675333	-4.559485096	5.12792E-06	0.000995842	6.56374E-05
165.013	74.843	0.544017279	0.120030833	4.532312775	5.83414E-06	0.000999697	6.58914E-05
198.085	100.113	-0.546263227	0.120243256	-4.542984341	5.54634E-06	0.000999697	6.58914E-05
258.15	74.904	-0.500119728	0.110810599	-4.513284225	6.38314E-06	0.001033005	6.80868E-05
373.057	87.578	-0.467032938	0.106069367	-4.403089685	1.0672E-05	0.001636186	0.000107843
159.076	102.224	-0.53939516	0.123895462	-4.353631273	1.33901E-05	0.001695883	0.000111778
199.088	100.117	-0.513495554	0.117901406	-4.35529626	1.32887E-05	0.001695883	0.000111778
225.034	75.303	0.508153662	0.11630712	4.369067528	1.24778E-05	0.001695883	0.000111778
257.147	74.625	-0.543945838	0.124102794	-4.383026527	1.17042E-05	0.001695883	0.000111778
113.071	101.512	-0.518993447	0.120774209	-4.297220833	1.72953E-05	0.001799328	0.000118596
130.123	27.204	0.483676183	0.112238777	4.309350091	1.63735E-05	0.001799328	0.000118596
160.08	100.482	-0.527106147	0.122317969	-4.309310803	1.63764E-05	0.001799328	0.000118596
165.033	87.089	-0.51210626	0.11910825	-4.299502859	1.71182E-05	0.001799328	0.000118596
220.067	99.688	-0.493055094	0.114263543	-4.315069198	1.59553E-05	0.001799328	0.000118596
177.106	100.743	-0.517965579	0.121941496	-4.247656416	2.16018E-05	0.002169867	0.000143019
146.118	59.646	-0.551948303	0.131035779	-4.212195391	2.52901E-05	0.002414261	0.000159127
666.523	68.093	-0.531174118	0.126210715	-4.208629325	2.56924E-05	0.002414261	0.000159127
482.36	49.589	-0.546037854	0.130175072	-4.194642239	2.73303E-05	0.002487908	0.000163982
664.526	68.434	-0.516229622	0.124057339	-4.161217912	3.16555E-05	0.002794316	0.000184177
1152.248	67.801	-0.412152749	0.100426229	-4.104034918	4.06006E-05	0.003379254	0.000222731
670.518	68.336	-0.465308412	0.113378511	-4.104026493	4.06021E-05	0.003379254	0.000222731
104.992	76.36	0.446201378	0.111338223	4.007620806	6.13335E-05	0.003646213	0.000240327
1162.277	67.73	-0.4944695	0.122628424	-4.032258465	5.52434E-05	0.003646213	0.000240327
170.055	95.669	-0.567172229	0.141298422	-4.014002561	5.96977E-05	0.003646213	0.000240327
176.06	99.313	-0.448325619	0.111656507	-4.015221605	5.939E-05	0.003646213	0.000240327
176.103	102.372	-0.498734978	0.124273597	-4.013201418	5.99008E-05	0.003646213	0.000240327
214.18	26.723	-0.411132984	0.101332342	-4.057273097	4.9649E-05	0.003646213	0.000240327

221.07	102.078	-0.435179548	0.108577392	-4.008012542	6.12319E-05	0.003646213	0.000240327
226.038	75.461	0.615732013	0.152863027	4.027998305	5.62537E-05	0.003646213	0.000240327
288.119	128.322	0.632602014	0.156403817	4.044671207	5.23966E-05	0.003646213	0.000240327
314.158	42.873	-0.463512338	0.114457471	-4.049646873	5.1295E-05	0.003646213	0.000240327
322.81	69.374	-0.512179312	0.126841514	-4.037947017	5.3921E-05	0.003646213	0.000240327
326.987	76.127	-0.472768376	0.116173108	-4.069516459	4.71108E-05	0.003646213	0.000240327
402.083	87.844	-0.448750787	0.111223567	-4.034673566	5.46783E-05	0.003646213	0.000240327
614.6	67.891	-0.516055096	0.128173831	-4.026212612	5.66824E-05	0.003646213	0.000240327
106.999	75.195	-0.471968381	0.118242899	-3.991515631	6.56524E-05	0.003683491	0.000242784
131.111	109.554	-0.461581987	0.115546089	-3.994786754	6.47525E-05	0.003683491	0.000242784
326.805	68.896	-0.508514959	0.127410679	-3.991148638	6.57541E-05	0.003683491	0.000242784
117.077	78.853	0.454784038	0.114710052	3.964639807	7.35069E-05	0.00402426	0.000265245
608.565	68.642	-0.516680285	0.130438038	-3.96111664	7.46001E-05	0.00402426	0.000265245
205.068	73.03	0.546934609	0.13891126	3.937295007	8.24053E-05	0.004364483	0.000287669
250.064	59.738	-0.499940782	0.127157267	-3.931672926	8.43568E-05	0.004388059	0.000289223
360.15	108.476	0.492120691	0.125566946	3.91918979	8.88471E-05	0.004540556	0.000299275
745.593	42.325	-0.426672387	0.109027734	-3.913429832	9.09944E-05	0.004570113	0.000301223
1038.657	51.615	-0.382165566	0.097778806	-3.908470359	9.28823E-05	0.004585869	0.000302261
331.092	96.557	-0.410724202	0.106058972	-3.872602132	0.00010768	0.005227841	0.000344574
722.486	66.165	-0.498879614	0.128979902	-3.867886441	0.000109783	0.005242577	0.000345546
572.662	68.132	-0.441624902	0.114471292	-3.857953346	0.00011434	0.005372158	0.000354087
672.558	68.111	-0.467264647	0.122059043	-3.828185411	0.000129091	0.005968945	0.000393422
315.161	42.821	-0.436886424	0.114473128	-3.816497633	0.000135359	0.006160966	0.000406078
542.153	102.319	-0.415414208	0.108984636	-3.811676784	0.000138027	0.006161856	0.000406137
546.83	77.148	-0.449865781	0.118110377	-3.808859067	0.000139609	0.006161856	0.000406137
362.062	90.079	0.433441253	0.114884416	3.772846385	0.000161396	0.007017098	0.000462507
508.711	67.545	-0.453517097	0.120439024	-3.765532812	0.000166194	0.00711947	0.000469254
520.12	55.829	-0.40321674	0.107270668	-3.758872281	0.000170681	0.007205704	0.000474938
169.059	96.539	-0.450702399	0.120104096	-3.752598066	0.000175011	0.007282972	0.000480031
261.043	87.544	-0.410809119	0.109592787	-3.748505089	0.000177892	0.007298572	0.000481059
662.53	68.617	-0.472958143	0.126621483	-3.73521248	0.000187557	0.007588234	0.000500151
101.071	96.528	-0.427360205	0.115462266	-3.701297572	0.0002145	0.008559421	0.000564164
382.766	68.554	-0.470982477	0.127535166	-3.692961652	0.000221657	0.008725516	0.000575111
146.6	96.634	-0.408180858	0.110681505	-3.687886763	0.000226124	0.008762598	0.000577555
438.727	68.044	-0.43661567	0.118481464	-3.685096858	0.000228616	0.008762598	0.000577555
329.03	87.116	-0.416656507	0.113248831	-3.67912412	0.000234036	0.008853869	0.000583571
476.306	44.819	0.435771288	0.118656271	3.672551692	0.000240141	0.008854803	0.000583633
504.871	71.618	0.449818738	0.122415442	3.674526104	0.000238291	0.008854803	0.000583633
312.782	71.919	-0.478396914	0.130520975	-3.665287632	0.000247061	0.0089961	0.000592946
349.064	96.841	-0.391997506	0.107759604	-3.63770366	0.00027508	0.009707666	0.000639846
604.571	68.42	-0.467977334	0.128547301	-3.640506874	0.000272102	0.009707666	0.000639846
606.568	68.596	-0.468140927	0.128741601	-3.636283259	0.0002766	0.009707666	0.000639846
254.824	72.613	-0.462001321	0.127195772	-3.632206591	0.000281008	0.009744956	0.000642304
216.063	122.209	0.472565262	0.130291688	3.626979355	0.000286756	0.009827304	0.000647732

149.077	96.666	-0.375150478	0.103858631	-3.61212617	0.000303697	0.010168604	0.000670227
159.092	58.364	-0.422738439	0.116949388	-3.614712707	0.000300681	0.010168604	0.000670227
147.555	96.563	-0.407323981	0.113074342	-3.602267104	0.000315454	0.010235925	0.000674664
276.119	116.59	-0.412859954	0.114556764	-3.603977091	0.000313385	0.010235925	0.000674664
369.894	67.014	-0.364005202	0.101067274	-3.601612957	0.000316249	0.010235925	0.000674664
148.08	94.797	-0.420332454	0.116970618	-3.593487505	0.000326281	0.010444587	0.000688418
238.107	39.303	0.382458071	0.107399039	3.561093976	0.000369313	0.011324299	0.000746401
316.776	71.543	-0.467105678	0.131163569	-3.561245563	0.0003691	0.011324299	0.000746401
388.254	41.378	0.396108571	0.111103936	3.565207373	0.000363569	0.011324299	0.000746401
90.055	84.601	0.417977342	0.117289621	3.563634528	0.000365755	0.011324299	0.000746401
343.123	109.031	0.438886087	0.123415821	3.556157415	0.000376319	0.011418919	0.000752637
556.859	81.955	-0.407543249	0.114735406	-3.552026896	0.000382276	0.011480096	0.00075667
1034.331	67.999	-0.421794371	0.119530486	-3.528759785	0.000417512	0.011882505	0.000783193
161.137	55	0.440859679	0.125191851	3.521472652	0.000429157	0.011882505	0.000783193
207.024	79.355	0.518496188	0.147321665	3.519483615	0.000432388	0.011882505	0.000783193
232.027	52.237	0.368922542	0.104482237	3.530959461	0.000414055	0.011882505	0.000783193
314.779	72.2	-0.459059945	0.13016364	-3.526790925	0.000420629	0.011882505	0.000783193
363.017	77.333	-0.350364448	0.099206708	-3.531660862	0.000412959	0.011882505	0.000783193
486.656	68.489	-0.413587935	0.117245594	-3.527534992	0.000419448	0.011882505	0.000783193
496.84	72.752	0.44007193	0.124333524	3.539447085	0.000400966	0.011882505	0.000783193
566.67	67.811	-0.416748913	0.118343784	-3.521510806	0.000429095	0.011882505	0.000783193
307.019	63.703	-0.404227846	0.115038101	-3.513860551	0.000441645	0.012023465	0.000792484
159.084	121.116	0.446248342	0.127636958	3.496231405	0.000471879	0.012727633	0.000838897
151.144	58.911	0.470982919	0.135858548	3.466715396	0.000526859	0.014024894	0.000924401
202.119	68.492	0.495985849	0.143128519	3.465318104	0.000529605	0.014024894	0.000924401
127.03	48.917	-0.343651504	0.099341486	-3.459294975	0.000541591	0.014086213	0.000928442
173.092	90.691	0.396287991	0.114512691	3.460646904	0.000538879	0.014086213	0.000928442
568.426	36.345	-0.421133913	0.121948327	-3.453379999	0.000553609	0.014271343	0.000940645
189.123	85.138	0.379061904	0.110097483	3.442966124	0.000575371	0.014702253	0.000969047
148.06	89.479	0.396376774	0.11528655	3.438187476	0.000585622	0.014834062	0.000977734
130.05	94.746	-0.411359557	0.119764901	-3.434725471	0.000593154	0.014895334	0.000981773
149.081	97.299	-0.397844027	0.115927261	-3.431841866	0.000599497	0.014925938	0.00098379
174.05	76.594	-0.372310137	0.108916665	-3.418302767	0.00063013	0.015094932	0.000994929
281.008	53.407	-0.383008736	0.112001769	-3.41966684	0.000626979	0.015094932	0.000994929
430.696	70.699	-0.440985897	0.128786456	-3.424163628	0.000616695	0.015094932	0.000994929
570.665	67.778	-0.419045895	0.122620813	-3.417412467	0.000632194	0.015094932	0.000994929
740.546	67.144	-0.382228743	0.111704395	-3.421787859	0.000622108	0.015094932	0.000994929
271.04	74.974	0.428800981	0.125569986	3.414836573	0.000638203	0.015114513	0.000996219
480.345	47	-0.427241232	0.125426641	-3.406303717	0.000658489	0.015469177	0.001019596
1041.685	49.43	-0.425316629	0.124970037	-3.403348835	0.000665652	0.015512363	0.001022442
177.1	100.826	-0.330761597	0.097597059	-3.389052901	0.000701345	0.016214423	0.001068716
386.76	68.644	-0.387309465	0.114518077	-3.382081462	0.000719388	0.01650061	0.001087579
130.077	96.602	-0.39787057	0.118520338	-3.35698139	0.000787984	0.017932796	0.001181976
428.698	68.62	-0.432406176	0.12930848	-3.343989329	0.000825829	0.018648374	0.001229141

551.425	36.279	-0.383521946	0.11497516	-3.335693961	0.000850868	0.019065983	0.001256666
164.029	86.645	-0.397945897	0.119624418	-3.326627657	0.000879037	0.01954173	0.001288023
245.049	81.304	0.387142346	0.116448474	3.324580673	0.000885516	0.01954173	0.001288023
483.364	49.807	-0.357132568	0.107843186	-3.311591421	0.000927669	0.020318049	0.001339192
1128.196	68.006	-0.339773289	0.102760951	-3.306443616	0.000944884	0.020388489	0.001343835
444.719	68.358	-0.374438315	0.11320785	-3.307529596	0.000941228	0.020388489	0.001343835
163.06	105.755	0.383725655	0.116451442	3.295155898	0.000983671	0.021069128	0.001388696
298.101	114.873	-0.352327645	0.106989663	-3.293099867	0.000990893	0.021069128	0.001388696
223.985	85.258	-0.366622857	0.111672254	-3.283025499	0.001026994	0.021678499	0.001428861
151.048	94.49	-0.391649138	0.119391498	-3.280377115	0.001036684	0.021725618	0.001431967
365.152	102.353	-0.31995319	0.09773169	-3.273791651	0.001061148	0.022079462	0.001455289
324.99	75.983	-0.378867178	0.116409185	-3.254615837	0.001135458	0.023458088	0.001546156
206.094	58.321	-0.391951398	0.120759978	-3.245706094	0.001171597	0.023700436	0.00156213
214.023	130.222	-0.336026519	0.103522082	-3.245940509	0.001170633	0.023700436	0.00156213
318.97	76.169	0.383796898	0.118107722	3.249549571	0.001155879	0.023700436	0.00156213
358.057	88.539	-0.336681073	0.103942715	-3.239102147	0.001199066	0.024088827	0.001587729
138.013	74.729	-0.341053894	0.105567346	-3.230676014	0.001234978	0.024640356	0.001624081
147.076	98.78	-0.377622332	0.117290405	-3.21955006	0.001283919	0.025442566	0.001676956
346.862	70.229	-0.316699702	0.098541492	-3.213871601	0.001309582	0.025775763	0.001698917
212.101	91.485	0.430502956	0.134086726	3.210630671	0.00132444	0.025829234	0.001702442
682.586	67.347	-0.366821346	0.11429523	-3.209419547	0.001330033	0.025829234	0.001702442
210.888	69.976	-0.397287416	0.123941382	-3.205446072	0.001348533	0.02601508	0.001714691
301.118	42.143	-0.354224136	0.111013868	-3.19080978	0.001418747	0.027189531	0.001792101
130.041	96.347	-0.33320186	0.104701845	-3.182387662	0.001460662	0.027629269	0.001821085
252.108	109.021	0.39523565	0.124188462	3.182547269	0.001459857	0.027629269	0.001821085
335.84	67.832	-0.372788514	0.117321469	-3.17749613	0.001485527	0.027918318	0.001840136
98.058	59.679	0.444652947	0.140067165	3.174569473	0.00150059	0.028020626	0.00184688
193.028	89.085	0.376883962	0.119417802	3.156011547	0.001599425	0.029488138	0.001943605
265.035	59.429	-0.366206755	0.116005694	-3.156799839	0.001595108	0.029488138	0.001943605
188.071	59.806	-0.38914189	0.123414488	-3.153129729	0.0016153	0.029593516	0.001950551
246.955	138.586	-0.347224814	0.110269714	-3.148868378	0.00163904	0.029840772	0.001966848
1047.732	48.709	-0.365825174	0.116576082	-3.13808087	0.00170058	0.03076887	0.00202802
548.827	78.227	-0.367280709	0.11726624	-3.132024254	0.001736055	0.031216847	0.002057547
247.14	110.194	0.355030415	0.113710073	3.122242434	0.001794791	0.032075003	0.002114109
1134.187	68.175	-0.353141742	0.113183974	-3.120068408	0.00180809	0.032115655	0.002116789
249.108	93.074	-0.377997582	0.122286522	-3.091081304	0.00199429	0.035208287	0.002320629
353.9	79.616	-0.358073092	0.116012759	-3.086497499	0.002025297	0.035540294	0.002342512
205.043	58.162	-0.306996642	0.099538649	-3.084195398	0.002041035	0.035602005	0.002346579
131.053	97.331	-0.364532755	0.118403001	-3.078745899	0.002078739	0.036043849	0.002375702
374.734	70.26	-0.386324805	0.125563834	-3.076720365	0.002092915	0.036074926	0.00237775
390.798	67.798	-0.377516324	0.123380735	-3.059767179	0.002215091	0.037956238	0.00250175
289.975	84.825	-0.344573221	0.112867439	-3.052901916	0.0022664	0.038608323	0.00254473
120.003	86.774	-0.387627691	0.127069137	-3.050525881	0.00228441	0.038688873	0.002550039
207.897	68.486	-0.382241344	0.125702293	-3.040846245	0.002359143	0.039337844	0.002592814

336.834	67.349	-0.364215065	0.119794839	-3.040323503	0.002363242	0.039337844	0.002592814
365.105	100.434	0.335412435	0.110288997	3.041213948	0.002356263	0.039337844	0.002592814
85.052	96.569	-0.308768759	0.10178328	-3.033590185	0.002416625	0.039997885	0.002636318
334.836	67.586	-0.363366784	0.120358098	-3.019047247	0.00253571	0.04156498	0.002739607
509.379	48.096	-0.380310974	0.125991126	-3.01855365	0.002539844	0.04156498	0.002739607
351.088	56.365	0.305005264	0.101104375	3.016736563	0.002555118	0.041581327	0.002740685
101.977	134.09	-0.348974207	0.116184126	-3.003630696	0.002667789	0.042837472	0.00282992
277.953	141.319	-0.323234654	0.107593357	-3.004225022	0.002662583	0.042837472	0.00282992
199.119	92.991	-0.331377335	0.110418426	-3.0011054	0.002690014	0.042837472	0.002837817
150.084	96.708	-0.302419879	0.101046535	-2.992877286	0.002763608	0.043752127	0.002899524
1150.25	67.735	-0.284961955	0.095308309	-2.989896253	0.002790722	0.04394256	0.002912058
585.195	102.316	-0.338904562	0.113417255	-2.988121701	0.002806978	0.043960891	0.002913188
1154.245	67.712	-0.280126249	0.093884715	-2.983725813	0.002847618	0.044080026	0.002936451
189.074	58.358	-0.364135031	0.122094634	-2.982399949	0.002859981	0.044080026	0.002936451
121.063	90.503	-0.331105226	0.111354831	-2.97342488	0.002944965	0.044914568	0.003007624
372.1	37.886	0.311225335	0.105318211	2.955095143	0.003125726	0.04742313	0.003175341
412.853	73.358	-0.31321659	0.106357343	-2.944945617	0.003230114	0.048252939	0.003264116
272.185	35.432	0.324976176	0.110698561	2.935685625	0.003328114	0.049083594	0.003345539
238.092	127.057	0.379832162	0.12992262	2.923526033	0.003460913	0.050373797	0.003460913

Supplementary Table 13. Sensitivity analysis of additionally adjusting for sample collection year among PD patients.

				C18			
mz	rt	beta	se	zvalue	pvalue	Full MWAS FDR p value	Replication FDR p value
209.067	36.808	-0.885275204	0.118046159	-7.499398671	6.41113E-14	1.42E-10	8.5268E-12
199.038	31.744	-0.835301409	0.115316709	-7.243541861	4.37116E-13	4.86E-10	2.90682E-11
200.041	32.191	-0.64529204	0.095298451	-6.77127524	1.27652E-11	7.09108E-09	4.24443E-10
223.082	38.976	-0.780508881	0.114886197	-6.793756798	1.0925E-11	7.09108E-09	4.24443E-10
269.088	35.076	0.739516914	0.120192135	6.152789568	7.61318E-10	3.3833E-07	2.02511E-08
113.024	36.458	0.655238047	0.110070151	5.952913135	2.63411E-09	9.06E-07	5.42275E-08
143.035	36.297	0.670733999	0.112922397	5.939778273	2.85408E-09	9.06E-07	5.42275E-08
225.062	37.895	0.656682217	0.111779728	5.874788097	4.23385E-09	1.17595E-06	7.03877E-08
215.033	30.913	0.597352114	0.110865061	5.388100694	7.12062E-08	1.3185E-05	7.89202E-07
215.091	31.459	0.600190939	0.110770474	5.418329624	6.01584E-08	1.3185E-05	7.89202E-07
217.03	30.51	0.594754393	0.110295135	5.392390082	6.95266E-08	1.3185E-05	7.89202E-07
218.033	30.739	0.591914011	0.109587733	5.401279798	6.61671E-08	1.3185E-05	7.89202E-07
195.051	31.406	0.597704173	0.112559422	5.310121189	1.09552E-07	1.87E-05	1.1208E-06
174.088	30.043	-0.626936971	0.118563157	-5.287789092	1.23804E-07	1.92021E-05	1.14936E-06
216.035	31.278	0.576366939	0.109173387	5.279372167	1.29627E-07	1.92021E-05	1.14936E-06
179.056	36.803	0.557365408	0.107415674	5.18886479	2.1158E-07	2.93832E-05	1.75876E-06
425.291	227.677	0.692820097	0.135312496	5.12014869	3.05295E-07	3.99038E-05	2.38848E-06
226.065	36.217	0.578027714	0.116765002	4.950350736	7.40799E-07	9.14475E-05	5.47368E-06
280.622	30.778	-0.485514288	0.100501555	-4.830913217	1.35908E-06	0.000158941	9.51358E-06
180.06	36.11	0.517935627	0.109215338	4.742334148	2.1127E-06	0.000234721	1.40494E-05
161.987	32.772	0.509872936	0.109480234	4.657214546	3.20516E-06	0.000339137	2.02994E-05
177.04	33.332	0.547400036	0.121386569	4.50956017	6.49622E-06	0.000656118	3.92726E-05
415.306	235.89	0.600526642	0.134871321	4.452589621	8.48408E-06	0.000819636	4.90601E-05
145.062	30.621	-0.530342211	0.119558707	-4.435830921	9.17178E-06	0.000849154	5.0827E-05
239.077	35.736	0.489290601	0.11054082	4.426334111	9.5848E-06	0.000851897	5.09912E-05
261.041	34.747	-0.441185735	0.10047517	-4.390992685	1.12834E-05	0.0009643	5.77191E-05
401.291	235.525	0.555342313	0.129443936	4.290214989	1.785E-05	0.001468991	8.79279E-05
160.062	29.915	0.470989793	0.110427696	4.265141892	1.99775E-05	1.59E-03	9.48932E-05
311.14	35.236	-0.421635804	0.099260447	-4.247772565	2.15906E-05	0.00165429	9.90191E-05
402.293	232.497	0.564196299	0.134078932	4.207941485	2.57708E-05	0.001908754	0.00011425
416.309	235.341	0.56817907	0.135855894	4.182218766	2.88678E-05	0.002069169	0.000123852
494.81	33.188	-0.403262428	0.096623022	-4.173564625	2.99871E-05	0.002082226	0.000124634
89.04	33.444	0.452253214	0.110843601	4.080102136	4.50159E-05	0.003031072	0.000181428
260.023	28.879	0.435825765	0.108145802	4.029983188	5.57808E-05	0.003645442	0.000218202
158.061	87.22	-0.506510086	0.126225629	-4.012735683	6.00191E-05	3.80E-03	0.0002273
89.024	31.295	0.445248592	0.111120889	4.006884711	6.15249E-05	0.003797452	0.0002273
253.093	34.817	-0.397774285	0.09962612	-3.992670645	6.53333E-05	0.003923529	0.000234847
149.046	31.935	0.453363609	0.115062334	3.940156534	8.14285E-05	4.41E-03	0.000263688
270.091	34.705	0.54782083	0.139019263	3.940610958	8.12743E-05	0.00440537	0.000263688
324.921	34.963	-0.430465955	0.10940001	-3.934788996	8.32698E-05	0.00440537	0.000263688
503.631	32.36	-0.437784671	0.111013337	-3.943532227	8.02902E-05	0.00440537	0.000263688

89.009	34.171	0.437781477	0.110848591	3.949364369	7.8359E-05	0.00440537	0.000263688
566.582	32.607	-0.48682001	0.123903719	-3.929018536	8.52933E-05	0.00440748	0.000263814
90.028	31.729	0.432970355	0.11118316	3.894208017	9.85201E-05	0.004975264	0.000297799
193.123	84.259	-0.44615039	0.115178785	-3.873546597	0.000107263	5.30E-03	0.000317021
213.049	35.964	-0.450430781	0.116988448	-3.850215876	0.000118014	0.005700578	0.000341214
496.807	33.723	-0.386509064	0.103176308	-3.746102888	0.000179603	0.008491016	0.000508238
279.614	29.271	-0.393057797	0.105250487	-3.734498618	0.00018809	0.008699835	0.000520737
439.306	227.232	0.560926618	0.150402126	3.729512566	0.000191851	0.008699835	0.000520737
203.021	32.471	-0.39583969	0.107080236	-3.696664322	0.000218451	0.009707957	0.000581079
268.96	35.981	-0.404335641	0.109715118	-3.685322922	0.000228413	0.009951634	0.000595665
424.278	219.234	0.479098912	0.13125454	3.65015115	0.000262086	0.011199138	0.000670335
216.971	30.784	0.448174461	0.123059963	3.641919371	0.000270613	0.011345316	0.000679085
224.952	56.909	-0.390701179	0.107632196	-3.629965695	0.000283459	0.011483385	0.000687349
256.934	34.638	-0.40729702	0.11222612	-3.629253329	0.000284242	0.011483385	0.000687349
103.04	32.211	0.395372254	0.109365733	3.615138323	0.000300187	1.18E-02	0.000709195
326.087	28.546	0.386230006	0.106932106	3.611918075	0.000303941	0.011848351	0.000709195
229.144	107.471	-0.379777401	0.10536733	-3.604318371	0.000312973	0.011914614	0.000713161
276	31.551	0.374892637	0.104092954	3.601517898	0.000316365	0.011914614	0.000713161
414.204	35.694	-0.463791815	0.129257132	-3.588133267	0.000333054	0.012334102	0.00073827
327.091	28.264	0.377354791	0.105598958	3.573470794	0.000352281	0.012832252	0.000768087
501.634	32.333	-0.374026051	0.104833714	-3.567803116	0.000359987	0.012901463	0.00077223
446.67	32.191	-0.430332691	0.12221928	-3.52098858	0.000429941	0.015163955	0.000907654
282.975	35.721	-0.34903217	0.099260178	-3.516336338	0.000437546	0.015191061	0.000909276
94.908	31.854	-0.448937968	0.127907071	-3.50987608	0.000448316	0.015325496	0.000917323
114.056	36.877	0.392868571	0.112390585	3.495564772	0.00047306	0.015580189	0.000932568
322.932	37.944	-0.398226452	0.113952933	-3.49465733	0.000474671	0.015580189	0.000932568
94.942	31.882	-0.431789592	0.123599351	-3.493461641	0.000476801	0.015580189	0.000932568
373.26	213.573	0.498622856	0.143219483	3.481529517	0.000498559	0.015825682	0.000947262
498.638	32.269	-0.433884489	0.124623593	-3.481559774	0.000498503	0.015825682	0.000947262
415.216	36.104	-0.434052537	0.127059297	-3.416141502	0.000635152	0.019877584	0.001189792
144.97	29.085	-0.417177641	0.122919211	-3.393917325	0.000689005	2.11E-02	0.001260554
505.628	32.397	-0.350013698	0.103164411	-3.392775601	0.000691883	0.021059779	0.001260554
508.623	32.501	-0.370123832	0.109233372	-3.388376887	0.000703076	0.021111277	0.001263636
127.051	32.393	-0.404391088	0.119660276	-3.379493179	0.000726196	2.13E-02	0.001272454
506.626	32.343	-0.409080048	0.121060225	-3.379144959	0.000727117	0.021258593	0.001272454
281.124	29.849	-0.382691354	0.113527339	-3.370918017	0.000749181	0.021619237	0.001294041
300.877	34.139	-0.362012114	0.107785353	-3.358639211	0.000783273	0.021901844	0.001310956
312.143	35.633	-0.353857681	0.105474007	-3.354927821	0.000793857	0.021901844	0.001310956
415.322	215.543	-0.387762967	0.115634568	-3.353348171	0.000798402	0.021901844	0.001310956
416.953	38.484	-0.335961685	0.099884091	-3.363515475	0.000769565	0.021901844	0.001310956
149.061	32.211	-0.390054903	0.117051264	-3.332342507	0.000861182	2.33E-02	0.001396795
151.007	30.177	0.415043571	0.124997041	3.320427173	0.000898798	2.35E-02	0.001406355
445.988	31.336	-0.384327272	0.115621376	-3.324015719	0.000887312	0.023495639	0.001406355
581.24	42.898	-0.347965599	0.104750842	-3.321840592	0.000894258	0.023495639	0.001406355

387.275	216.137	0.445330912	0.134401168	3.313445241	0.000921542	0.023810061	0.001425175
286.06	27.608	0.507930478	0.153779559	3.302977864	0.000956639	0.02443279	0.001462449
148.98	33.213	0.378844862	0.114940141	3.296018764	0.000980655	2.45E-02	0.001465473
442.676	32.194	-0.400382746	0.121456288	-3.296517222	0.000978916	0.02448331	0.001465473
397.259	210.47	0.417506416	0.127646382	3.270804931	0.001072419	0.026476823	0.001584796
302.874	34.257	-0.339638256	0.10435318	-3.254699632	0.001135124	0.0274157	0.001640994
440.679	32.243	-0.418213503	0.128452851	-3.255774384	0.001130836	0.0274157	0.001640994
431.244	82.667	0.400944514	0.123374501	3.249816693	0.001154794	0.027590889	0.00165148
194.039	27.536	0.38010484	0.117323732	3.23979499	0.001196157	2.78E-02	0.001662767
219.034	30.853	0.358670483	0.110587929	3.243305908	0.001181513	0.02777946	0.001662767
254.982	28.027	0.391689725	0.1209354	3.238834316	0.001200193	0.02777946	0.001662767
390.709	32.22	-0.367826792	0.11426275	-3.219131277	0.001285796	0.029454009	0.001762999
189.067	63.388	-0.358271731	0.111660432	-3.208582694	0.001333909	3.02E-02	0.001810306
356.098	28.295	0.348503909	0.108780748	3.203727827	0.001356607	0.030448281	0.001822512
359.121	49.326	0.3981412	0.124915346	3.18728814	0.001436136	0.031910948	0.001910061
424.247	181.74	0.420360158	0.132413478	3.1746025	0.001500419	0.033009215	0.001975799
216.909	24.258	0.409064041	0.129135172	3.167719798	0.001536395	0.033469315	0.002003339
295.984	32.846	-0.324259288	0.103032951	-3.147141636	0.001648751	0.035568196	0.002128969
195.976	28.201	-0.377889968	0.120565956	-3.134300758	0.001722642	3.58E-02	0.002141228
220.049	30.642	-0.384094398	0.12253855	-3.134478071	0.001721601	0.035772994	0.002141228
266.963	36.467	-0.357345314	0.113865946	-3.138298368	0.001699318	0.035772994	0.002141228
374.263	214.626	0.405231239	0.129243846	3.135400665	0.001716195	0.035772994	0.002141228
163.077	35.967	-0.32801314	0.105060025	-3.122149837	0.001795355	0.036700023	0.002196716
254.623	31.407	-0.364945894	0.116919718	-3.121337437	0.001800316	0.036700023	0.002196716
217.048	30.99	0.440428538	0.142096095	3.099511909	0.001938398	0.038571269	0.002308721
423.275	218.074	0.454712014	0.146746218	3.098628504	0.001944186	0.038571269	0.002308721
444.673	32.115	-0.374903758	0.120867403	-3.10177723	0.001923627	0.038571269	0.002308721
221.053	30.668	-0.377593451	0.122177364	-3.090535248	0.001997961	0.039287337	0.002351582
226.018	34.225	-0.361087784	0.1169579	-3.087331289	0.002019624	0.039364948	0.002356228
223.05	30.675	-0.375416632	0.122270601	-3.070375278	0.0021379	0.040338111	0.002414477
225.054	30.806	-0.351776751	0.114593488	-3.069779595	0.002142168	0.040338111	0.002414477
403.296	242.279	0.422144032	0.137415328	3.072030163	0.002126082	0.040338111	0.002414477
96.922	31.526	-0.391087168	0.127255917	-3.073233668	0.002117526	0.040338111	0.002414477
188.057	31.438	0.346679327	0.113277172	3.060451826	0.002210033	4.13E-02	0.002470037
162.056	40.608	-0.389377378	0.12783726	-3.04588332	0.002319978	0.042813528	0.002562646
418.95	37.272	-0.31030972	0.102010941	-3.041925873	0.002350698	0.042813528	0.002562646
452.705	33.294	-0.329791426	0.108397599	-3.042423702	0.002346813	0.042813528	0.002562646
146.065	30.227	-0.337758221	0.111309504	-3.034405956	0.002410099	4.35E-02	0.002606042
156.878	31.938	-0.370940081	0.122929779	-3.01749572	0.002548726	4.42E-02	0.002648128
255.63	33.175	-0.297710447	0.098505321	-3.022277812	0.002508802	0.044241653	0.002648128
369.154	47.826	-0.338882012	0.11248032	-3.0128116	0.002588396	0.044241653	0.002648128
406.925	35.575	-0.296046458	0.098081798	-3.018362865	0.002541444	0.044241653	0.002648128
412.278	210.722	0.367617893	0.121471756	3.026365195	0.002475132	0.044241653	0.002648128
556.597	32.309	-0.377693369	0.125333886	-3.013497645	0.00258255	0.044241653	0.002648128

564.083	212.912	-0.332122175	0.109886576	-3.022408988	0.002507715	0.044241653	0.002648128
118.051	31.331	-0.368066749	0.12238327	-3.007492346	0.002634128	0.04467963	0.002674343
229.012	27.859	0.386962198	0.129695747	2.983615161	0.002848648	0.047952244	0.002870229
562.588	32.428	-0.351722638	0.118748882	-2.961902732	0.003057444	0.050698803	0.003057444

Supplementary Table 14. Sensitivity analysis of additionally adjusting for sample collection year among non-PD participants.

HILIC

mz	rt	beta	se	zvalue	pvalue	Full MWAS FDR p value	Replication FDR p value
271.04	74.974	1.84452538	0.333058984	5.538134288	3.05711E-08	8.90536E-05	1.89541E-06
116.992	75.398	1.907646137	0.360634551	5.289693214	1.22522E-07	0.000103097	2.1943E-06
203.053	75.296	1.244966269	0.242478352	5.134339877	2.83136E-07	0.000103097	2.1943E-06
204.056	74.501	1.262006401	0.245444196	5.141724369	2.72228E-07	0.000103097	2.1943E-06
219.026	74.325	1.376776939	0.264648581	5.202283464	1.96855E-07	0.000103097	2.1943E-06
225.034	75.303	1.54856094	0.290520152	5.330304733	9.80481E-08	0.000103097	2.1943E-06
226.038	75.461	1.763632655	0.341190359	5.169057712	2.35277E-07	0.000103097	2.1943E-06
494.843	73.994	1.602341164	0.309451578	5.178002878	2.24274E-07	0.000103097	2.1943E-06
104.992	76.36	1.367193484	0.269574183	5.071678117	3.94323E-07	0.000127629	2.71645E-06
492.846	74.043	1.598393274	0.316489822	5.050378126	4.40936E-07	0.000128445	2.73381E-06
378.925	74.581	1.681597687	0.338578892	4.966634738	6.81247E-07	0.000180407	3.83976E-06
376.928	74.723	1.431735266	0.295555964	4.844210379	1.27116E-06	0.000308575	6.56767E-06
244.079	71.325	1.137437623	0.236970837	4.799905494	1.58741E-06	0.000341753	7.27384E-06
552.801	72.015	1.562360764	0.325962417	4.793070248	1.64248E-06	0.000341753	7.27384E-06
496.84	72.752	1.370302285	0.289069011	4.74039843	2.13298E-06	0.000414225	8.81633E-06
324.99	75.983	-1.099269619	0.233306525	-4.711696862	2.45663E-06	0.00044726	9.51943E-06
293.021	90.496	1.613346639	0.349024422	4.622446275	3.79241E-06	0.00060988	1.29806E-05
320.967	76.018	1.349588791	0.293257492	4.602060743	4.18331E-06	0.00060988	1.29806E-05
326.987	76.127	-0.935956769	0.202612862	-4.619434133	3.84788E-06	0.00060988	1.29806E-05
504.871	71.618	1.715052945	0.372686722	4.601862223	4.1873E-06	0.00060988	1.29806E-05
207.024	79.355	1.368945503	0.304323653	4.498321083	6.84922E-06	0.000832297	1.77145E-05
384.945	75.296	-0.980776407	0.216781323	-4.524266174	6.06054E-06	0.000832297	1.77145E-05
502.874	72.075	2.449318368	0.544503123	4.498263214	6.85108E-06	0.000832297	1.77145E-05
508.894	71.963	-0.896720481	0.199356616	-4.498072353	6.85724E-06	0.000832297	1.77145E-05
382.948	74.924	-0.953691716	0.213321951	-4.470668445	7.79755E-06	0.000908571	1.93379E-05
231.055	75.316	-0.946320454	0.21353824	-4.431620551	9.35275E-06	0.001047868	2.23027E-05
277.06	75	-0.934475126	0.212375512	-4.40010769	1.08197E-05	0.001167327	2.48453E-05
318.97	76.169	1.295679677	0.299139625	4.331354218	1.48195E-05	0.001541758	3.28146E-05
106.999	75.195	-0.89234752	0.212016101	-4.208866759	2.56655E-05	0.002578052	5.4871E-05
335.094	86.335	0.835791818	0.203746817	4.102109822	4.094E-05	0.003975273	8.46093E-05
165.013	74.843	0.938851251	0.229515034	4.090587162	4.30282E-05	0.004043267	8.60565E-05
160.133	55.358	0.877185481	0.217580485	4.031544833	5.54114E-05	0.005044171	0.00010736
138.013	74.729	-0.740871837	0.197021093	-3.76036812	0.000169664	0.014120851	0.000300547
230.051	75.122	-0.748017951	0.198404798	-3.770160596	0.000163143	0.014120851	0.000300547
570.665	67.778	-0.860769802	0.22884739	-3.761326723	0.000169014	0.014120851	0.000300547
400.918	71.845	-0.690162122	0.18440261	-3.742691722	0.00018206	0.01473165	0.000313547
176.103	102.372	-0.751159247	0.20332274	-3.694418287	0.000220391	0.015498298	0.000329864
177.106	100.743	-0.752254771	0.203741929	-3.692194209	0.000222328	0.015498298	0.000329864
247.028	75.129	-0.712688415	0.192420355	-3.703809895	0.000212385	0.015498298	0.000329864
322.011	233.412	-0.598703868	0.161459301	-3.708079164	0.000208837	0.015498298	0.000329864
344.956	69.034	-0.596419928	0.160255403	-3.721683754	0.000197899	0.015498298	0.000329864

405.096	96.144	1.231965375	0.333783971	3.690906342	0.000223456	0.015498298	0.000329864
198.085	100.113	-0.744949016	0.202209541	-3.684044834	0.000229562	0.01555148	0.000330996
151.039	271.329	0.90939015	0.248092415	3.66552984	0.000246827	0.016341071	0.000347802
113.071	101.512	-0.730810812	0.201804193	-3.621385661	0.000293029	0.018735712	0.000398769
159.076	102.224	-0.724199637	0.2001161	-3.618897408	0.000295861	0.018735712	0.000398769
160.08	100.482	-0.724919394	0.200748249	-3.611087002	0.000304916	0.018898326	0.00040223
171.07	100.464	-0.973612732	0.271039656	-3.592141259	0.000327972	0.019903799	0.00042363
169.027	75.746	-0.66827699	0.187103219	-3.571702262	0.000354668	0.02108468	0.000448764
325.982	69.952	-0.569844961	0.16036973	-3.553319945	0.000380402	0.022162192	0.000471698
199.088	100.117	-0.688281407	0.194056561	-3.546808231	0.000389928	0.022271788	0.00047403
155.075	65.529	-0.578251606	0.163517509	-3.536328365	0.00040573	0.022728685	0.000483755
1200.178	67.919	-0.526328049	0.150800124	-3.490236182	0.000482594	0.026033256	0.000554089
135.022	84.894	0.794848348	0.227415762	3.495133056	0.000473825	0.026033256	0.000554089
163.06	105.755	0.690525778	0.202036948	3.417819289	0.00063125	0.032303902	0.000711262
177.1	100.826	-0.634790318	0.186154	-3.410027807	0.000649563	0.032303902	0.000711262
245.042	232.059	-0.571803413	0.168006347	-3.40346315	0.000665374	0.032303902	0.000711262
293.034	73.377	-0.690640998	0.202721203	-3.406851323	0.000657169	0.032303902	0.000711262
455.296	45.646	0.844071215	0.250242249	3.373016424	0.000743495	0.034932281	0.0007813
419.315	278.321	-0.57591392	0.171913366	-3.350024108	0.000808045	0.0372831	0.00083498
360.063	88.585	-0.664073468	0.200081294	-3.319018253	0.000903345	0.040483765	0.000918154
311.204	43.067	0.801541528	0.247183929	3.242692721	0.001184058	0.051480031	0.001184058

Supplementary Table 14. Sensitivity analysis of additionally adjusting for sample collection year among non-PD participants.

C18

mz	rt	beta	se	zvalue	pvalue	Full MWAS FDR p value	Replication FDR p value
113.024	36.458	1.043210153	0.220003144	4.741796563	2.11831E-06	3.92E-04	5.64883E-06
143.035	36.297	1.205596157	0.234887192	5.132660256	2.85675E-07	2.46E-04	3.5364E-06
154.058	35.834	-0.674565682	0.18531217	-3.640158555	0.00027247	0.022423289	0.000335348
174.088	30.043	-0.745965697	0.195058288	-3.824321973	0.000131132	0.012668536	0.000182445
179.056	36.803	1.094942716	0.224997856	4.866458442	1.13616E-06	0.000336312	4.84338E-06
180.06	36.11	1.140430549	0.235755108	4.837352457	1.3158E-06	3.36E-04	4.84338E-06
199.038	31.744	-0.788823137	0.189255101	-4.168041619	3.07228E-05	3.59E-03	5.17436E-05
200.041	32.191	-0.584034479	0.154742327	-3.774238691	0.000160497	0.014859351	0.000213996
209.067	36.808	-0.829306521	0.193029725	-4.296263288	1.73701E-05	0.002412276	3.47403E-05
215.033	30.913	1.321480031	0.265599597	4.975459468	6.50931E-07	0.000245558	3.5364E-06
215.091	31.459	1.151600062	0.248657025	4.631279014	3.63414E-06	0.00057679	8.3066E-06
216.035	31.278	1.336982419	0.286055916	4.673849919	2.95605E-06	0.000505258	7.27644E-06
216.971	30.784	1.230403387	0.308028832	3.994442267	6.48467E-05	6.86E-03	9.8814E-05
217.03	30.51	1.298723711	0.261213912	4.97187804	6.63074E-07	0.000245558	3.5364E-06
218.033	30.739	1.49185243	0.308842921	4.830456942	1.3622E-06	0.000336312	4.84338E-06
219.034	30.853	1.712688317	0.359114156	4.769203023	1.84956E-06	0.000373611	5.38054E-06
220.049	30.642	-0.735835769	0.201407553	-3.653466611	0.000258723	0.022110904	0.000331166
220.992	30.561	-0.76201487	0.197726066	-3.853891835	0.000116255	0.011741752	0.000169098
221.053	30.668	-0.724476303	0.200499749	-3.61335266	0.000302263	0.023159611	0.000345444
223.05	30.675	-0.726533794	0.200733346	-3.61939761	0.00029529	0.023159611	0.000345444
223.082	38.976	-0.704705739	0.198601279	-3.548344407	0.000387661	0.027786537	0.000427764
225.062	37.895	1.175814565	0.23622127	4.977598186	6.43781E-07	0.000245558	3.5364E-06
226.065	36.217	1.275468317	0.252080025	5.059775424	4.19751E-07	0.000245558	3.5364E-06
239.077	35.736	1.121519283	0.234025428	4.792296691	1.64883E-06	0.00036637	5.27625E-06
265.002	30.312	-1.054647261	0.234947648	-4.488860695	7.16051E-06	0.00106071	1.52758E-05
267	30.325	-0.956696138	0.228049131	-4.195131689	2.72713E-05	0.003366492	4.84823E-05
269.088	35.076	1.053825559	0.246868043	4.268780792	1.96544E-05	0.00256895	3.69966E-05
270.091	34.705	1.153506637	0.279336924	4.12944562	3.63639E-05	4.04E-03	5.81822E-05
283.103	34.333	2.017520001	0.574582505	3.5112799	0.000445955	0.030965971	0.000475685
344.135	35.626	1.762024685	0.504302785	3.493981669	0.000475874	0.031357328	0.000491224
359.121	49.326	1.782767008	0.347412632	5.131554941	2.87358E-07	0.000245558	3.5364E-06
484.04	37.132	1.395129538	0.406482498	3.43220076	0.000598704	0.038009163	0.000598704

Supplementary Table 15. Sensitivity analysis of additionally adjusting for T2DM medication use.

PD Group

HILIC

Mass to charge ratio	Retention Time	beta	se	zvalue	p value	FDR p value	Overlapping with main analysis
130.136	49.642	1.100011434	0.140452054	7.831935521	4.80416E-15	1.39945E-11	Significant in both
335.094	86.335	1.027080627	0.13682686	7.506425476	6.07637E-14	8.85023E-11	Significant in both
204.056	74.501	0.71246987	0.116249977	6.128774301	8.85586E-10	8.59904E-07	Significant in both
203.053	75.296	0.693042063	0.115670031	5.99154386	2.07858E-09	1.51373E-06	Significant in both
160.133	55.358	0.716853748	0.128514415	5.578002666	2.43296E-08	1.41744E-05	Significant in both
744.59	42.524	-0.731291439	0.136610031	-5.353131356	8.6445E-08	4.19691E-05	Significant in both
1037.653	51.282	-0.670954411	0.126523451	-5.303004363	1.13912E-07	4.74037E-05	Significant in both
221.988	85.683	-0.664226207	0.127988147	-5.189747813	2.10579E-07	7.66771E-05	Significant in both
668.521	68.55	-0.692474118	0.136617021	-5.06872507	4.00489E-07	0.000129625	Significant in both
231.055	75.316	-0.62959547	0.12626209	-4.986417289	6.15092E-07	0.000177188	Significant in both
313.154	42.894	-0.603525859	0.121430689	-4.970126289	6.69093E-07	0.000177188	Significant in both
165.013	74.843	0.624757448	0.127885985	4.885269063	1.03288E-06	0.0002471	Significant in both
219.026	74.325	0.623453987	0.128343456	4.857699851	1.18757E-06	0.0002471	Significant in both
496.686	67.924	-0.692047615	0.142074076	-4.871033722	1.11016E-06	0.0002471	Significant in both
190.119	93.248	0.577575747	0.122102848	4.73023976	2.24255E-06	0.000384267	Significant in both
225.034	75.303	0.584853194	0.123141109	4.74945531	2.03965E-06	0.000384267	Significant in both
244.079	71.325	0.55734486	0.117665899	4.736672762	2.17256E-06	0.000384267	Significant in both
258.15	74.904	-0.543314239	0.115708435	-4.695545655	2.65896E-06	0.000407661	Significant in both
360.15	108.476	0.633270127	0.134806866	4.697610347	2.63223E-06	0.000407661	Significant in both
198.085	100.113	-0.573986779	0.12490714	-4.595307988	4.32111E-06	0.000626633	Significant in both
666.523	68.093	-0.614332102	0.133957141	-4.586034738	4.51743E-06	0.000626633	Significant in both
402.083	87.844	-0.529790898	0.117102012	-4.524182725	6.06293E-06	0.000802787	Significant in both
326.987	76.127	-0.545640104	0.121889687	-4.476507538	7.5874E-06	0.000960961	Significant in both
104.992	76.36	0.515548412	0.116549398	4.423432654	9.71449E-06	0.000977179	Significant in both
130.123	27.204	0.516014678	0.115917453	4.451570194	8.52447E-06	0.000977179	Significant in both
159.076	102.224	-0.573106486	0.129976092	-4.409322328	1.03695E-05	0.000977179	Significant in both
161.137	55	0.593262975	0.134566288	4.408704316	1.03991E-05	0.000977179	Significant in both
220.067	99.688	-0.527474955	0.119129612	-4.427740075	9.52256E-06	0.000977179	Significant in both
257.147	74.625	-0.575035569	0.129990245	-4.423682488	9.70326E-06	0.000977179	Significant in both
288.119	128.322	0.74991048	0.168459709	4.451571737	8.5244E-06	0.000977179	Significant in both
670.518	68.336	-0.519192607	0.117620045	-4.414150727	1.01407E-05	0.000977179	Significant in both
199.088	100.117	-0.539203411	0.122677424	-4.39529452	1.10623E-05	0.001007012	Significant in both
106.999	75.195	-0.549042369	0.126087412	-4.354458236	1.33396E-05	0.001118857	Significant in both
113.071	101.512	-0.551105895	0.126392704	-4.360266686	1.29904E-05	0.001118857	Significant in both
722.486	66.165	-0.601145841	0.138106696	-4.352763908	1.34432E-05	0.001118857	Significant in both
160.08	100.482	-0.556050916	0.128062685	-4.34202137	1.41178E-05	0.001121084	Significant in both
608.565	68.642	-0.604207732	0.139402447	-4.334269206	1.46245E-05	0.001121084	Significant in both
614.6	67.891	-0.596891104	0.137563723	-4.339015323	1.43123E-05	0.001121084	Significant in both
177.106	100.743	-0.550773492	0.127659135	-4.314407197	1.60032E-05	0.001146371	Significant in both
226.038	75.461	0.712472211	0.165187189	4.313120264	1.60966E-05	0.001146371	Significant in both

664.526	68.434	-0.569695803	0.132100481	-4.312594471	1.6135E-05	0.001146371	Significant in both
146.118	59.646	-0.587782974	0.136905112	-4.293360323	1.75989E-05	0.00122061	Significant in both
221.07	102.078	-0.482940233	0.112865173	-4.278912798	1.87808E-05	0.001272293	Significant in both
250.064	59.738	-0.569745113	0.134140093	-4.247388676	2.16277E-05	0.001431849	Significant in both
131.111	109.554	-0.508881192	0.120256167	-4.231643196	2.3199E-05	0.001488247	Significant in both
165.033	87.089	-0.530305804	0.125541354	-4.22415234	2.39842E-05	0.001488247	Significant in both
314.158	42.873	-0.507002501	0.120042411	-4.223528128	2.40507E-05	0.001488247	Significant in both
322.81	69.374	-0.567547698	0.134611875	-4.216178529	2.48477E-05	0.001488247	Significant in both
373.057	87.578	-0.474283929	0.11253642	-4.214492764	2.5034E-05	0.001488247	Significant in both
176.06	99.313	-0.478825762	0.114662878	-4.175944036	2.96753E-05	0.001678369	Significant in both
223.985	85.258	-0.492623571	0.118096282	-4.171372377	3.02771E-05	0.001678369	Significant in both
542.153	102.319	-0.469093244	0.112623003	-4.165163722	3.11129E-05	0.001678369	Significant in both
662.53	68.617	-0.560165571	0.13441463	-4.167444938	3.08033E-05	0.001678369	Significant in both
672.558	68.111	-0.536464345	0.128414882	-4.17758704	2.94618E-05	0.001678369	Significant in both
326.805	68.896	-0.56049919	0.134830412	-4.157067981	3.22358E-05	0.001707326	Significant in both
1041.685	49.43	-0.53779815	0.131912694	-4.076924939	4.56352E-05	0.002373846	Significant in both
1152.248	67.801	-0.409495769	0.100684797	-4.067106251	4.76005E-05	0.002432638	Significant in both
170.055	95.669	-0.611989078	0.150746966	-4.059710735	4.91335E-05	0.00246769	Significant in both
271.04	74.974	0.547721103	0.135230942	4.050264636	5.11597E-05	0.002525904	Significant in both
176.103	102.372	-0.526494842	0.130212473	-4.043351837	5.26925E-05	0.002558219	Significant in both
315.161	42.821	-0.482371135	0.119999032	-4.019791889	5.82496E-05	0.002651266	Significant in both
382.766	68.554	-0.54695111	0.135716324	-4.030105552	5.57518E-05	0.002651266	Significant in both
604.571	68.42	-0.550270865	0.13677545	-4.023169841	5.74201E-05	0.002651266	Significant in both
606.568	68.596	-0.55078022	0.136935757	-4.022179674	5.7662E-05	0.002651266	Significant in both
546.83	77.148	-0.49462183	0.12340599	-4.008086063	6.12128E-05	0.002743276	Significant in both
312.782	71.919	-0.554478565	0.13890591	-3.991756463	6.55857E-05	0.00285151	Significant in both
476.306	44.819	0.498291408	0.124729206	3.994985813	6.46981E-05	0.00285151	Significant in both
1038.657	51.615	-0.395695223	0.099229927	-3.987660124	6.67282E-05	0.002858517	Significant in both
254.824	72.613	-0.530312246	0.133688757	-3.966767728	7.28539E-05	0.003075703	Significant in both
238.107	39.303	0.437529754	0.110399316	3.963156387	7.39653E-05	0.003078013	Significant in both
307.019	63.703	-0.468922746	0.11999469	-3.907862457	9.31163E-05	0.003770375	Significant in both
98.058	59.679	0.578997936	0.148169716	3.90766718	9.31916E-05	0.003770375	Significant in both
151.144	58.911	0.565519213	0.144912139	3.902497179	9.52053E-05	0.003799085	Significant in both
745.593	42.325	-0.468449307	0.120236324	-3.896071419	9.77656E-05	0.003848528	Significant in both
314.779	72.2	-0.538796054	0.138645599	-3.886138897	0.000101851	0.003955902	Significant in both
329.03	87.116	-0.463610053	0.119626204	-3.875489122	0.000106411	0.004078611	Significant in both
159.092	58.364	-0.470059805	0.121491906	-3.86906274	0.000109255	0.004133227	Significant in both
316.776	71.543	-0.538214561	0.139241978	-3.865318259	0.000110944	0.004143348	Significant in both
174.05	76.594	-0.440082892	0.114136866	-3.855747101	0.000115377	0.004254334	Significant in both
1162.277	67.73	-0.496411884	0.129103953	-3.845055651	0.000120525	0.00438862	Significant in both
508.711	67.545	-0.48748786	0.127142249	-3.83419252	0.000125977	0.004530522	Significant in both
261.043	87.544	-0.42965982	0.112514165	-3.818717589	0.000134147	0.004765499	Significant in both
331.092	96.557	-0.419256528	0.109966222	-3.812593731	0.000137516	0.004826316	Significant in both
173.092	90.691	0.450351115	0.118486643	3.800859787	0.000144195	0.005000473	Significant in both

1134.187	68.175	-0.449001207	0.11855283	-3.787351244	0.000152262	0.005218101	Significant in both
324.99	75.983	-0.463691293	0.122566743	-3.783173809	0.000154841	0.005244796	Significant in both
428.698	68.62	-0.525820256	0.139350638	-3.773360947	0.000161063	0.005392834	Significant in both
388.254	41.378	0.434538777	0.11538867	3.765870409	0.00016597	0.00549398	Significant in both
349.064	96.841	-0.418413631	0.111282135	-3.759935318	0.000169957	0.005500951	Significant in both
482.36	49.589	-0.501704103	0.133349308	-3.762330017	0.000168338	0.005500951	Significant in both
205.068	73.03	0.539701886	0.144116696	3.744894937	0.000180469	0.005714207	Significant in both
386.76	68.644	-0.458332942	0.122354916	-3.745929914	0.000179727	0.005714207	Significant in both
90.055	84.601	0.461505009	0.123389295	3.740235398	0.000183848	0.005758594	Significant in both
438.727	68.044	-0.463212886	0.124304938	-3.726423838	0.000194216	0.006018621	Significant in both
1047.732	48.709	-0.450014581	0.120970749	-3.720028049	0.000199201	0.006108121	Significant in both
486.656	68.489	-0.450496767	0.121380232	-3.711450846	0.000206075	0.006253079	Significant in both
127.03	48.917	-0.376475121	0.101615223	-3.704908676	0.000211467	0.006350547	Significant in both
430.696	70.699	-0.507146099	0.137040539	-3.700701285	0.000215004	0.006390898	Significant in both
101.071	96.528	-0.442891663	0.121060241	-3.65844028	0.000253755	0.007466544	Significant in both
149.077	96.666	-0.389594914	0.106728306	-3.650342888	0.00026189	0.007553336	Significant in both
169.059	96.539	-0.461024722	0.126210238	-3.652831412	0.000259365	0.007553336	Significant in both
117.077	78.853	0.426897639	0.117291249	3.63963759	0.000273022	0.007721487	Significant in both
520.12	55.829	-0.406969564	0.111772441	-3.641054642	0.000271523	0.007721487	Significant in both
424.143	112.801	-0.399744483	0.109954036	-3.635559874	0.000277378	0.007769239	Significant only in sensitivity analysis
572.662	68.132	-0.434633761	0.119759527	-3.629220743	0.000284278	0.007886686	Significant in both
301.118	42.143	-0.420226036	0.115960183	-3.623882139	0.000290214	0.00790087	Significant in both
566.67	67.811	-0.453984726	0.125236976	-3.625005483	0.000288955	0.00790087	Significant in both
147.555	96.563	-0.42521578	0.117961064	-3.604712993	0.000312498	0.008416591	Significant in both
246.955	138.586	-0.405767345	0.11263544	-3.602483775	0.000315191	0.008416591	Significant in both
444.719	68.358	-0.423731308	0.117692666	-3.600320422	0.000317825	0.008416591	Significant in both
202.119	68.492	0.544939362	0.151958631	3.586103397	0.000335656	0.0088087	Significant in both
148.08	94.797	-0.439286256	0.122847995	-3.575852047	0.000349089	0.009079432	Significant in both
177.1	100.826	-0.353887688	0.09924539	-3.565784629	0.000362769	0.009337302	Significant in both
568.426	36.345	-0.455111695	0.127701221	-3.563878955	0.000365415	0.009337302	Significant in both
365.152	102.353	-0.356213574	0.100116574	-3.557988039	0.000373706	0.009466144	Significant in both
1034.331	67.999	-0.442445359	0.124655488	-3.549345214	0.00038619	0.009698041	Significant in both
206.094	58.321	-0.448757436	0.126743297	-3.540679842	0.000399098	0.009852298	Significant in both
346.862	70.229	-0.355550591	0.100395439	-3.541501433	0.000397857	0.009852298	Significant in both
189.123	85.138	0.404395717	0.114578095	3.529433055	0.000416451	0.010194302	Significant in both
362.062	90.079	0.420204412	0.119330818	3.521340261	0.000429371	0.010400994	Significant in both
504.871	71.618	0.444902027	0.126403408	3.519699609	0.000432036	0.010400994	Significant in both
232.027	52.237	0.383880541	0.109201586	3.515338502	0.000439194	0.010401394	Significant in both
374.734	70.26	-0.470133206	0.133656674	-3.517468996	0.000435683	0.010401394	Significant in both
138.013	74.729	-0.382624023	0.109009645	-3.510001538	0.000448104	0.010526833	Significant in both
207.024	79.355	0.541733858	0.15461984	3.503650354	0.000458927	0.010694846	Significant in both
363.017	77.333	-0.3629698	0.103722954	-3.499416324	0.000466278	0.010779902	Significant in both
163.06	105.755	0.420533789	0.120345804	3.494378504	0.000475167	0.010898905	Significant in both
318.97	76.169	0.431987369	0.123721327	3.491616027	0.000480108	0.010926205	Significant in both

146.6	96.634	-0.400080504	0.114750477	-3.486525836	0.000489338	0.011049937	Significant in both
551.425	36.279	-0.417106702	0.120449007	-3.462931839	0.000534324	0.011972962	Significant in both
216.063	122.209	0.461055474	0.134295124	3.433151246	0.000596609	0.01316608	Significant in both
740.546	67.144	-0.397008862	0.115570807	-3.435200213	0.000592116	0.01316608	Significant in both
188.071	59.806	-0.443000238	0.129261962	-3.42715082	0.00060995	0.013359285	Significant in both
570.665	67.778	-0.437806455	0.128384651	-3.410115248	0.000649354	0.014116188	Significant in both
353.9	79.616	-0.411323887	0.121062337	-3.397620573	0.000679746	0.014667414	Significant in both
480.345	47	-0.440848648	0.130368241	-3.381564757	0.000720742	0.015437667	Significant in both
164.029	86.645	-0.424666609	0.125686642	-3.378772806	0.000728102	0.015481458	Significant in both
276.119	116.59	-0.399218541	0.118302892	-3.374545913	0.000739376	0.015541366	Significant in both
682.586	67.347	-0.406194563	0.120399498	-3.373723055	0.000741589	0.015541366	Significant in both
130.05	94.746	-0.421833876	0.12540801	-3.363691635	0.000769074	0.016002237	Significant in both
496.84	72.752	0.429386566	0.128076536	3.352577907	0.000800627	0.016540614	Significant in both
343.123	109.031	0.430119101	0.128513543	3.346877609	0.000817273	0.016765604	Significant in both
277.953	141.319	-0.372769639	0.111899881	-3.331278247	0.000864481	0.017610031	Significant in both
130.077	96.602	-0.413395959	0.12483575	-3.311519004	0.000927909	0.01802	Significant in both
159.084	121.116	0.440948966	0.133102224	3.312859493	0.000923474	0.01802	Significant in both
256.821	72.9	-0.443459448	0.133904783	-3.311752103	0.000927137	0.01802	Significant only in sensitivity analysis
289.975	84.825	-0.390845879	0.117854744	-3.316335578	0.000912062	0.01802	Significant in both
360.063	88.585	-0.399718672	0.120427111	-3.319175123	0.000902838	0.01802	Significant only in sensitivity analysis
365.105	100.434	0.381637686	0.115205079	3.312681068	0.000924063	0.01802	Significant in both
483.364	49.807	-0.364116704	0.109807893	-3.315942915	0.000913345	0.01802	Significant in both
1128.196	68.006	-0.349406529	0.105667676	-3.306654806	0.000944172	0.018214384	Significant in both
85.052	96.569	-0.344781422	0.104534155	-3.298265748	0.00097284	0.018643969	Significant in both
149.081	97.299	-0.401311073	0.121892493	-3.292336263	0.000993587	0.018794285	Significant in both
556.859	81.955	-0.405823463	0.123205959	-3.293862293	0.000988209	0.018794285	Significant in both
212.101	91.485	0.46610142	0.141672478	3.289992704	0.0010019	0.018829252	Significant in both
148.06	89.479	0.392988833	0.119533947	3.287675531	0.001010182	0.018863205	Significant in both
120.003	86.774	-0.446764253	0.136337672	-3.276895127	0.001049553	0.019473562	Significant in both
548.61	68.658	-0.42627708	0.130242982	-3.27293703	0.001064362	0.019499913	Significant only in sensitivity analysis
554.645	68.137	-0.415251524	0.126867411	-3.273114188	0.001063695	0.019499913	Significant only in sensitivity analysis
210.888	69.976	-0.426051261	0.130629969	-3.261512381	0.001108196	0.020068153	Significant in both
357.963	84.122	-0.354138746	0.108589322	-3.26126676	0.001109156	0.020068153	Significant only in sensitivity analysis
189.074	58.358	-0.41671883	0.128043694	-3.254504911	0.001135902	0.020369369	Significant in both
265.035	59.429	-0.393688842	0.121003439	-3.253534312	0.00113979	0.020369369	Significant in both
205.097	59.833	-0.419825955	0.129332713	-3.246092535	0.001170008	0.02065596	Significant only in sensitivity analysis
351.088	56.365	0.341108195	0.105074027	3.24636074	0.001168906	0.02065596	Significant in both
1150.25	67.735	-0.312983547	0.096494056	-3.243552591	0.00118049	0.020715469	Significant in both
249.061	60.032	-0.399186292	0.123543173	-3.231148121	0.00123294	0.021506318	Significant only in sensitivity analysis
245.049	81.304	0.386058104	0.120024584	3.21649192	0.001297682	0.022500877	Significant in both
298.101	114.873	-0.351530667	0.109499109	-3.210351851	0.001325726	0.022851121	Significant in both
101.977	134.09	-0.387373691	0.121170919	-3.196919654	0.001389036	0.023787516	Significant in both
151.048	94.49	-0.403228615	0.126190438	-3.195397545	0.001396384	0.023787516	Significant in both
193.028	89.085	0.397915446	0.124633592	3.192682165	0.00140958	0.023872713	Significant in both

1132.19	68.158	-0.366979919	0.115159934	-3.186697879	0.00143907	0.024231279	Significant only in sensitivity analysis
432.28	43.808	0.372501981	0.117238871	3.177290745	0.001486579	0.024887387	Significant only in sensitivity analysis
116.992	75.398	0.360744529	0.114029353	3.163611114	0.001558249	0.02593816	Significant only in sensitivity analysis
390.798	67.798	-0.412965012	0.13079565	-3.157329869	0.001592211	0.026352904	Significant in both
543.325	53.816	-0.431083725	0.136917042	-3.148503064	0.00164109	0.027008448	Significant only in sensitivity analysis
1154.245	67.712	-0.2994077	0.09529738	-3.141825108	0.001678983	0.027323337	Significant in both
198.862	74.033	-0.429488452	0.136678604	-3.142323964	0.001676125	0.027323337	Significant only in sensitivity analysis
509.379	48.096	-0.40948094	0.130771689	-3.131265966	0.001740545	0.028167812	Significant in both
144.081	62.767	-0.421157241	0.134641142	-3.127998128	0.001760013	0.028225527	Significant only in sensitivity analysis
334.836	67.586	-0.398172711	0.127316753	-3.127418038	0.00176349	0.028225527	Significant in both
300.004	86.638	-0.3916408	0.125638292	-3.117208894	0.001825722	0.029061896	Significant only in sensitivity analysis
233.872	253.929	-0.338542478	0.108832663	-3.110669791	0.001866635	0.02955168	Significant only in sensitivity analysis
1160.279	67.582	-0.390724021	0.125704249	-3.108280138	0.001881796	0.029570445	Significant only in sensitivity analysis
206.894	71.399	-0.407122784	0.131021899	-3.107288065	0.001888123	0.029570445	Significant only in sensitivity analysis
147.076	98.78	-0.38159288	0.123174227	-3.097992911	0.001948361	0.03035067	Significant in both
191.066	104.919	0.409707541	0.13254631	3.091052043	0.001994487	0.030813468	Significant only in sensitivity analysis
205.043	58.162	-0.313878904	0.101567519	-3.090347252	0.001999226	0.030813468	Significant in both
484.366	40.72	-0.32458037	0.105232522	-3.084411209	0.002039555	0.031269595	Significant only in sensitivity analysis
238.092	127.057	0.420813115	0.136537154	3.082041068	0.002055865	0.031354628	Significant in both
337.109	95.451	-0.362848066	0.117871225	-3.078343058	0.002081551	0.031581037	Significant only in sensitivity analysis
1164.275	67.557	-0.42098272	0.137156991	-3.069349339	0.002145256	0.032037608	Significant only in sensitivity analysis
199.119	92.991	-0.348663055	0.113639781	-3.068142617	0.002153938	0.032037608	Significant in both
214.023	130.222	-0.341175266	0.111317958	-3.064871761	0.002177633	0.032037608	Significant in both
270.23	36.512	0.394568746	0.128665858	3.066615739	0.00216497	0.032037608	Significant only in sensitivity analysis
281.008	53.407	-0.36894577	0.120267568	-3.067707926	0.002157073	0.032037608	Significant in both
378.925	74.581	0.341804264	0.111472452	3.066266676	0.002167499	0.032037608	Significant only in sensitivity analysis
336.834	67.349	-0.385200743	0.125829569	-3.061289532	0.002203859	0.032260505	Significant in both
150.084	96.708	-0.318773471	0.104236973	-3.058161235	0.002226997	0.032436214	Significant in both
196.865	74.031	-0.413464638	0.135460544	-3.052288339	0.002271038	0.032913108	Significant only in sensitivity analysis
372.1	37.886	0.330941016	0.108615086	3.04691574	0.002312025	0.033341233	Significant in both
249.108	93.074	-0.392806315	0.129010223	-3.044768891	0.002328592	0.033414717	Significant in both
270.1	117.939	0.457822097	0.150844624	3.035057431	0.002404899	0.034173026	Significant only in sensitivity analysis
358.057	88.539	-0.325643069	0.107293837	-3.035058477	0.002404891	0.034173026	Significant in both
1144.216	67.937	-0.325974978	0.107478232	-3.032939526	0.002421841	0.034246717	Significant only in sensitivity analysis
260.815	68.885	-0.393984207	0.130645357	-3.015677063	0.002564062	0.036082667	Significant only in sensitivity analysis
247.14	110.194	0.362539103	0.120348958	3.012399171	0.002591915	0.036299275	Significant in both
506.714	67.783	-0.356513258	0.118524486	-3.007929154	0.002630344	0.036661213	Significant only in sensitivity analysis
1220.235	67.565	-0.405304029	0.135075725	-3.000568972	0.002694757	0.037307939	Significant only in sensitivity analysis
162.015	132.11	-0.367312096	0.122449167	-2.999710848	0.00270236	0.037307939	Significant only in sensitivity analysis
1063.67	49.471	-0.35577531	0.118753478	-2.995914872	0.002736228	0.037597323	Significant only in sensitivity analysis
272.185	35.432	0.342583328	0.114840655	2.983118906	0.002853271	0.039021498	Significant in both
1146.214	67.781	-0.30286414	0.101983703	-2.969730773	0.002980608	0.040572488	Significant only in sensitivity analysis
131.053	97.331	-0.369370889	0.124463457	-2.967705518	0.003000316	0.040650797	Significant in both
258.818	71.593	-0.389357945	0.131491163	-2.961095916	0.003065465	0.041341196	Significant only in sensitivity analysis

508.894	71.963	-0.335407338	0.11334671	-2.959127258	0.003085117	0.041414493	Significant only in sensitivity analysis
199.144	33.14	0.311284197	0.105656395	2.946193629	0.003217109	0.042213693	Significant only in sensitivity analysis
207.897	68.486	-0.387382962	0.131479828	-2.946330001	0.003215691	0.042213693	Significant in both
212.02	130.286	-0.324799367	0.110212426	-2.947030365	0.003208417	0.042213693	Significant only in sensitivity analysis
252.108	109.021	0.38125672	0.129405492	2.94621745	0.003216862	0.042213693	Significant in both
538.798	71.325	-0.350438755	0.118938717	-2.946380834	0.003215163	0.042213693	Significant only in sensitivity analysis
246.17	47.929	0.364301717	0.124288237	2.931103746	0.0033776	0.043728656	Significant only in sensitivity analysis
502.677	68.359	-0.335795178	0.114485915	-2.933069793	0.003356284	0.043728656	Significant only in sensitivity analysis
936.475	67.205	-0.305443897	0.104173724	-2.932062774	0.003367187	0.043728656	Significant only in sensitivity analysis
200.859	73.078	-0.384309937	0.131188035	-2.929458757	0.003395529	0.043751885	Significant only in sensitivity analysis
454.388	37.893	-0.315535387	0.107757881	-2.928188494	0.003409433	0.043751885	Significant only in sensitivity analysis
335.84	67.832	-0.355354339	0.121973021	-2.913384752	0.003575338	0.045679645	Significant in both
797.623	41.35	-0.296118155	0.101891358	-2.906214644	0.003658304	0.046535545	Significant only in sensitivity analysis
369.894	67.014	-0.311562193	0.107339558	-2.90258502	0.003700967	0.04687355	Significant in both
218.92	71.96	-0.351958852	0.121468605	-2.897529373	0.003761145	0.047301137	Significant only in sensitivity analysis
244.154	37.556	0.317189112	0.109487895	2.89702449	0.003767203	0.047301137	Significant only in sensitivity analysis
450.752	67.129	-0.355125446	0.122988331	-2.887472688	0.003883503	0.048552124	Significant only in sensitivity analysis
198.128	36.477	0.387377255	0.13426667	2.885133409	0.003912478	0.048705338	Significant only in sensitivity analysis
121.063	90.503	-0.338114874	0.117256297	-2.883554085	0.003932151	0.048741942	Significant in both

C18

Mass to charge ratio	Retention Time	beta	se	zvalue	p value	FDR p value	Overlapping with main analysis
209.067	36.808	-0.953871368	0.12370796	-7.710670927	1.25158E-14	2.78101E-11	Significant in both
199.038	31.744	-0.90742954	0.120881937	-7.506742229	6.06169E-14	6.73454E-11	Significant in both
200.041	32.191	-0.736287317	0.100820352	-7.302963192	2.81498E-13	2.08496E-10	Significant in both
223.082	38.976	-0.78773144	0.120138501	-6.556860919	5.49522E-11	3.0526E-08	Significant in both
269.088	35.076	0.821989345	0.129298013	6.357323884	2.05299E-10	9.12348E-08	Significant in both
113.024	36.458	0.729558869	0.115704279	6.305375013	2.87497E-10	1.0647E-07	Significant in both
143.035	36.297	0.743038403	0.118799571	6.254554595	3.98652E-10	1.26544E-07	Significant in both
225.062	37.895	0.710928733	0.117015791	6.075493976	1.23607E-09	3.43317E-07	Significant in both
215.033	30.913	0.656949035	0.116522552	5.637956112	1.7208E-08	3.33567E-06	Significant in both
215.091	31.459	0.655461733	0.116032553	5.648946909	1.61434E-08	3.33567E-06	Significant in both
217.03	30.51	0.651691796	0.115752135	5.630062846	1.80144E-08	3.33567E-06	Significant in both
218.033	30.739	0.648556544	0.114759056	5.651462855	1.59088E-08	3.33567E-06	Significant in both
179.056	36.803	0.619159791	0.112000413	5.528192022	3.23548E-08	5.15267E-06	Significant in both
216.035	31.278	0.631484419	0.114242164	5.527595024	3.2465E-08	5.15267E-06	Significant in both
226.065	36.217	0.637985192	0.122506624	5.207760806	1.91133E-07	2.83132E-05	Significant in both
195.051	31.406	0.597718858	0.116156205	5.145819456	2.66356E-07	3.69901E-05	Significant in both
174.088	30.043	-0.636057065	0.123918603	-5.132861814	2.85369E-07	3.72995E-05	Significant in both
180.06	36.11	0.577742727	0.114225854	5.057898062	4.23903E-07	5.23285E-05	Significant in both
280.622	30.778	-0.500974763	0.102793487	-4.873604118	1.0958E-06	0.000128151	Significant in both
261.041	34.747	-0.494744256	0.103292283	-4.789750443	1.66989E-06	0.000185525	Significant in both
239.077	35.736	0.546933373	0.115581905	4.731998253	2.2232E-06	0.000235236	Significant in both
177.04	33.332	0.588247079	0.126705295	4.642640074	3.43985E-06	0.000347425	Significant in both
253.093	34.817	-0.462398565	0.10226527	-4.521560114	6.13855E-06	0.000593038	Significant in both

311.14	35.236	-0.454343218	0.101013078	-4.497865311	6.86392E-06	0.000610065	Significant in both
425.291	227.677	0.632994435	0.140521205	4.504618598	6.64923E-06	0.000610065	Significant in both
494.81	33.188	-0.434621021	0.098896096	-4.394723739	1.10914E-05	0.000947886	Significant in both
149.046	31.935	0.525626947	0.120415249	4.365119465	1.27053E-05	0.001045602	Significant in both
160.062	29.915	0.490072277	0.114491941	4.280408479	1.86551E-05	0.001480412	Significant in both
161.987	32.772	0.486599894	0.114854692	4.23665665	2.26873E-05	0.001575347	Significant in both
566.582	32.607	-0.550669534	0.129473428	-4.253147089	2.10787E-05	0.001575347	Significant in both
89.024	31.295	0.494591236	0.116709384	4.237801773	2.25719E-05	0.001575347	Significant in both
89.04	33.444	0.492614961	0.116255352	4.237352956	2.2617E-05	0.001575347	Significant in both
145.062	30.621	-0.526001239	0.124832185	-4.213666856	2.51258E-05	0.001691803	Significant in both
503.631	32.36	-0.477810951	0.113846372	-4.196980052	2.70498E-05	0.001767782	Significant in both
496.807	33.723	-0.442498785	0.105851877	-4.18035843	2.9105E-05	0.001847751	Significant in both
260.023	28.879	0.469917595	0.113217252	4.150582952	3.3163E-05	0.002046891	Significant in both
193.123	84.259	-0.493711423	0.119818278	-4.120501744	3.78048E-05	0.002270333	Significant in both
89.009	34.171	0.476251811	0.116212195	4.098122498	4.16515E-05	0.002435516	Significant in both
90.028	31.729	0.476267185	0.116487264	4.088577303	4.34027E-05	0.002472841	Significant in both
158.061	87.22	-0.521577519	0.130730652	-3.989710998	6.61538E-05	0.003674846	Significant in both
270.091	34.705	0.593828963	0.149338878	3.976385606	6.99706E-05	0.003792067	Significant in both
224.952	56.909	-0.437294249	0.11249935	-3.887082445	0.000101456	0.005367522	Significant in both
415.306	235.89	0.537938681	0.139214861	3.864089482	0.000111504	0.005761922	Significant in both
501.634	32.333	-0.416601978	0.108138006	-3.852502867	0.000116917	0.005904289	Significant in both
324.921	34.963	-0.441625689	0.11538909	-3.827274226	0.00012957	0.006397885	Significant in both
103.04	32.211	0.432265087	0.113553583	3.806705829	0.00014083	0.006802706	Significant in both
498.638	32.269	-0.492792965	0.129988125	-3.791061407	0.000150005	0.007091719	Significant in both
282.975	35.721	-0.381061746	0.101163249	-3.766800185	0.000165353	0.007654477	Significant in both
256.934	34.638	-0.446114447	0.118789646	-3.755499417	0.000172996	0.00768795	Significant in both
268.96	35.981	-0.435584448	0.115983124	-3.755584717	0.000172937	0.00768795	Significant in both
446.67	32.191	-0.475167583	0.127464201	-3.727851261	0.000193119	0.008413942	Significant in both
279.614	29.271	-0.399360907	0.108071269	-3.695347626	0.000219586	0.009206045	Significant in both
327.091	28.264	0.407749064	0.110329696	3.695732694	0.000219254	0.009206045	Significant in both
213.049	35.964	-0.448559314	0.121830023	-3.681845424	0.000231552	0.009368181	Significant in both
401.291	235.525	0.493534112	0.134058683	3.681478156	0.000231886	0.009368181	Significant in both
402.293	232.497	0.50804683	0.138660928	3.663950892	0.000248354	0.009854349	Significant in both
286.06	27.608	0.614564881	0.168625969	3.644544692	0.000267866	0.010442058	Significant in both
414.204	35.694	-0.489192628	0.135371875	-3.613694698	0.000301864	0.011564533	Significant in both
505.628	32.397	-0.381386046	0.105784065	-3.60532607	0.000311761	0.011741245	Significant in both
506.626	32.343	-0.450717651	0.125615092	-3.588085178	0.000333115	0.012336376	Significant in both
442.676	32.194	-0.452175171	0.126590596	-3.57194913	0.000354334	0.012698882	Significant in both
94.908	31.854	-0.481443825	0.134667578	-3.57505372	0.000350156	0.012698882	Significant in both
416.953	38.484	-0.368439922	0.103312963	-3.566250654	0.000362125	0.012772092	Significant in both
508.623	32.501	-0.398870401	0.112152459	-3.556501608	0.000375826	0.013048215	Significant in both
300.877	34.139	-0.402708361	0.113597366	-3.545050149	0.000392538	0.013273505	Significant in both
416.309	235.341	0.495751171	0.139888794	3.543894798	0.000394263	0.013273505	Significant in both
216.971	30.784	0.449118291	0.127003701	3.536261439	0.000405833	0.013459113	Significant in both

302.874	34.257	-0.384204256	0.109051358	-3.523149683	0.00042645	0.013934895	Significant in both
281.124	29.849	-0.416916157	0.118515146	-3.517830186	0.000435091	0.014011181	Significant in both
94.942	31.882	-0.45189643	0.128649336	-3.512621541	0.000443709	0.014084592	Significant in both
312.143	35.633	-0.377495492	0.108812497	-3.469229203	0.000521954	0.016334953	Significant in both
581.24	42.898	-0.370848152	0.107424267	-3.452182277	0.000556072	0.017160998	Significant in both
254.982	28.027	0.431117919	0.125373708	3.43866289	0.000584595	0.017794102	Significant in both
564.083	212.912	-0.393978762	0.114722647	-3.434184715	0.000594339	0.017846234	Significant in both
444.673	32.115	-0.43021599	0.125729329	-3.421763187	0.000622165	0.018190133	Significant in both
452.705	33.294	-0.379919189	0.110942673	-3.424464005	0.000616013	0.018190133	Significant in both
326.087	28.546	0.381707966	0.111905174	3.410994798	0.000647263	0.018678168	Significant in both
440.679	32.243	-0.457238792	0.134204254	-3.407036506	0.000656723	0.018708198	Significant in both
220.049	30.642	-0.443993902	0.131133864	-3.385806593	0.000709694	0.019809803	Significant in both
435.146	46.59	-0.464575928	0.137267998	-3.384444558	0.000713224	0.019809803	Significant only in sensitivity analysis
219.034	30.853	0.387361832	0.115375136	3.357411706	0.000786759	0.021325852	Significant in both
373.26	213.573	0.505088717	0.150443751	3.357325993	0.000787003	0.021325852	Significant in both
114.056	36.877	0.389741531	0.116532291	3.344493844	0.000824329	0.02206817	Significant in both
221.053	30.668	-0.435346064	0.130592249	-3.33362865	0.00085721	0.022300125	Significant in both
223.05	30.675	-0.435552902	0.130729025	-3.331723007	0.000863101	0.022300125	Significant in both
585.262	138.73	-0.385774932	0.115591166	-3.337408441	0.000845636	0.022300125	Significant only in sensitivity analysis
415.322	215.543	-0.404634543	0.121577134	-3.328212542	0.000874052	0.022323478	Significant in both
265.002	30.312	-0.414710412	0.12484644	-3.321764012	0.000894503	0.022586204	Significant only in sensitivity analysis
408.921	37.112	-0.381131373	0.114940053	-3.315914377	0.000913438	0.022715278	Significant only in sensitivity analysis
605.238	135.326	-0.374172943	0.112910322	-3.313894926	0.000920061	0.022715278	Significant only in sensitivity analysis
276	31.551	0.360655762	0.108940661	3.310570709	0.000931059	0.022734216	Significant in both
127.051	32.393	-0.412066504	0.12550797	-3.283189941	0.001026395	0.0230532	Significant in both
144.97	29.085	-0.423078722	0.128316612	-3.297146909	0.000976724	0.0230532	Significant in both
148.98	33.213	0.395850006	0.120183941	3.293701339	0.000988775	0.0230532	Significant in both
179.012	36.848	0.425031893	0.128795584	3.300050209	0.000966675	0.0230532	Significant only in sensitivity analysis
189.067	63.388	-0.383660588	0.116725626	-3.286858261	0.001013118	0.0230532	Significant in both
213.006	33.813	0.434423514	0.132209545	3.285871019	0.001016676	0.0230532	Significant only in sensitivity analysis
439.306	227.232	0.50788474	0.154701881	3.282990084	0.001027123	0.0230532	Significant in both
562.588	32.428	-0.403321151	0.122671453	-3.287815874	0.001009679	0.0230532	Significant in both
203.021	32.471	-0.362748776	0.111285477	-3.259623691	0.001115601	0.024788659	Significant in both
195.976	28.201	-0.409708216	0.12625551	-3.245071951	0.00117421	0.025832609	Significant in both
229.144	107.471	-0.354954946	0.109654417	-3.237032808	0.001207795	0.026128503	Significant in both
359.121	49.326	0.419465817	0.129615385	3.236234769	0.001211177	0.026128503	Significant in both
454.702	33.379	-0.341376515	0.105726789	-3.228855399	0.001242867	0.026554336	Significant only in sensitivity analysis
244.915	35.658	-0.377962204	0.117193913	-3.22510098	0.001259282	0.026648815	Significant only in sensitivity analysis
390.709	32.22	-0.393108858	0.122345824	-3.213095828	0.001313125	0.027526067	Significant in both
151.007	30.177	0.414994637	0.129545241	3.203472644	0.001357809	0.028155775	Significant in both
221.033	29.078	-0.361896058	0.113049697	-3.201212115	0.001368507	0.028155775	Significant only in sensitivity analysis
225.054	30.806	-0.381043045	0.119187656	-3.197000903	0.001388645	0.028307973	Significant in both
261.111	87.558	-0.408680023	0.1283396	-3.184364172	0.001450724	0.029040614	Significant only in sensitivity analysis
322.932	37.944	-0.384805452	0.120782139	-3.185946666	0.001442812	0.029040614	Significant in both

558.594	32.365	-0.399497363	0.125562135	-3.181670668	0.001464282	0.029050313	Significant only in sensitivity analysis
188.057	31.438	0.379558905	0.119530272	3.175420736	0.001496194	0.029420733	Significant in both
87.973	28.539	-0.358739843	0.113304417	-3.16615937	0.001544661	0.030107347	Significant only in sensitivity analysis
254.623	31.407	-0.386511826	0.122372769	-3.158479031	0.001585947	0.030379092	Significant in both
556.597	32.309	-0.41077474	0.129985888	-3.160148738	0.001576886	0.030379092	Significant in both
226.018	34.225	-0.383593919	0.121887023	-3.147126826	0.001648834	0.030640189	Significant in both
272.073	37.138	-0.325851319	0.103557368	-3.146577828	0.001651933	0.030640189	Significant only in sensitivity analysis
295.984	32.846	-0.335325485	0.106338733	-3.153371066	0.001613965	0.030640189	Significant in both
334.049	36.375	0.401119214	0.127498014	3.146082066	0.001654736	0.030640189	Significant only in sensitivity analysis
382.88	33.695	-0.313153627	0.099701808	-3.14090219	0.001684283	0.030929555	Significant only in sensitivity analysis
246.905	38.3	-0.361711769	0.115317023	-3.136672804	0.001708767	0.031121971	Significant only in sensitivity analysis
212.002	34.278	0.393715038	0.125821501	3.129155473	0.001753095	0.031669737	Significant only in sensitivity analysis
378.956	34.258	-0.342611826	0.110003825	-3.114544656	0.001842291	0.03301266	Significant only in sensitivity analysis
578.089	213.548	-0.389100877	0.125187555	-3.108143432	0.001882667	0.033466284	Significant only in sensitivity analysis
194.039	27.536	0.377761222	0.122584436	3.081640983	0.00205863	0.035736524	Significant in both
266.963	36.467	-0.364380729	0.118223032	-3.082146695	0.002055135	0.035736524	Significant in both
387.275	216.137	0.430024479	0.139439743	3.083944872	0.002042754	0.035736524	Significant in both
415.216	36.104	-0.407429552	0.132517995	-3.074522457	0.002108398	0.036316755	Significant in both
409.307	162.174	-0.387964624	0.126381253	-3.069795685	0.002142053	0.036419147	Significant only in sensitivity analysis
445.988	31.336	-0.374884211	0.122238865	-3.066816856	0.002163514	0.036419147	Significant in both
448.668	32.25	-0.357735407	0.116631461	-3.067229058	0.002160532	0.036419147	Significant only in sensitivity analysis
96.922	31.526	-0.409684056	0.133798069	-3.06195791	0.002198944	0.03673724	Significant in both
163.077	35.967	-0.330908135	0.108181104	-3.058834886	0.002221996	0.036845333	Significant in both
156.878	31.938	-0.392524531	0.128926941	-3.044550095	0.002330286	0.038229427	Significant in both
339.057	36.653	-0.295461761	0.09708551	-3.043314702	0.002339875	0.038229427	Significant only in sensitivity analysis
406.925	35.575	-0.305772726	0.100911362	-3.03011196	0.002444631	0.039649416	Significant in both
436.851	33.515	-0.32456867	0.107295666	-3.024993292	0.002486386	0.040034425	Significant only in sensitivity analysis
223.057	34.827	0.354394168	0.117317011	3.02082506	0.00252087	0.040297642	Significant only in sensitivity analysis
560.591	32.367	-0.384097987	0.127793652	-3.005610843	0.00265048	0.0420669	Significant only in sensitivity analysis
559.337	191.995	-0.387782379	0.129548855	-2.993329272	0.002759519	0.043486881	Significant only in sensitivity analysis
118.051	31.331	-0.380715749	0.127748323	-2.980201546	0.002880588	0.044397979	Significant in both
133.014	116.205	0.415733	0.13965375	2.976883896	0.002911943	0.044397979	Significant only in sensitivity analysis
176.072	75.87	-0.392071312	0.13139666	-2.98387579	0.002846223	0.044397979	Significant only in sensitivity analysis
255.63	33.175	-0.301497279	0.101310631	-2.975968804	0.002920646	0.044397979	Significant in both
474.651	32.522	-0.340181136	0.114138596	-2.980421581	0.002878519	0.044397979	Significant only in sensitivity analysis
98.956	25.02	0.339550694	0.114164123	2.974232942	0.00293722	0.044397979	Significant only in sensitivity analysis
96.942	24.614	0.339946266	0.114437597	2.970582005	0.00297236	0.044625573	Significant only in sensitivity analysis
242.918	34.433	-0.342231648	0.11545107	-2.964300367	0.00303372	0.045241115	Significant only in sensitivity analysis
216.909	24.258	0.399691332	0.135055316	2.959463904	0.003081748	0.045650961	Significant in both
229.012	27.859	0.401101947	0.135633071	2.957257733	0.003103886	0.045674395	Significant in both
117.976	35.401	-0.330781138	0.112177741	-2.948723461	0.003190894	0.046456686	Significant only in sensitivity analysis
186.039	36.013	-0.280423117	0.095124709	-2.947952441	0.003198863	0.046456686	Significant only in sensitivity analysis
163.061	33.325	-0.330209241	0.112141597	-2.944574085	0.003233995	0.046477307	Significant only in sensitivity analysis
589.571	32.396	-0.324429516	0.110207809	-2.943797902	0.003242116	0.046477307	Significant only in sensitivity analysis

360.833	33.91	-0.29774019	0.101393109	-2.936493333	0.00331946	0.04728102	Significant only in sensitivity analysis
356.098	28.295	0.334551158	0.114385964	2.92475709	0.003447252	0.0485835	Significant in both
431.244	82.667	0.374856806	0.128195992	2.924091457	0.003454632	0.0485835	Significant in both
472.654	32.49	-0.316808965	0.108434578	-2.921659969	0.003481714	0.048656409	Significant only in sensitivity analysis
566.34	215.741	-0.340214694	0.116558671	-2.918827845	0.003513502	0.048793759	Significant only in sensitivity analysis
424.247	181.74	0.401031665	0.137568794	2.915135429	0.003555342	0.04906814	Significant in both

Supplementary Table 15. Sensitivity analysis of additionally adjusting for T2DM medication use.

				Non-PD Group			
				HILIC			
Mass to charge ratio	Retention Time	beta	se	zvalue	p value	FDR p value	Overlapping with main analysis
104.992	76.36	1.498610926	0.293007434	5.11458329	3.14434E-07	9.88338E-05	Significant in both
116.992	75.398	1.879145955	0.365855823	5.136301892	2.80197E-07	9.88338E-05	Significant in both
203.053	75.296	1.303254287	0.255686843	5.097072159	3.44947E-07	9.88338E-05	Significant in both
204.056	74.501	1.330883107	0.261874745	5.082136153	3.73214E-07	9.88338E-05	Significant in both
219.026	74.325	1.406511676	0.276070499	5.094755428	3.49192E-07	9.88338E-05	Significant in both
225.034	75.303	1.715081368	0.32027748	5.354985841	8.55631E-08	9.88338E-05	Significant in both
226.038	75.461	1.810898027	0.355710921	5.090926145	3.56319E-07	9.88338E-05	Significant in both
271.04	74.974	1.959018984	0.358512287	5.464300826	4.64735E-08	9.88338E-05	Significant in both
378.925	74.581	1.885001321	0.370635829	5.085858335	3.65967E-07	9.88338E-05	Significant in both
492.846	74.043	1.756552401	0.342047698	5.135401902	2.81542E-07	9.88338E-05	Significant in both
494.843	73.994	1.733673447	0.331947077	5.222740517	1.76294E-07	9.88338E-05	Significant in both
376.928	74.723	1.565766574	0.31694823	4.940133523	7.80691E-07	0.000189513	Significant in both
496.84	72.752	1.518888441	0.311663129	4.873494166	1.09642E-06	0.000245681	Significant in both
552.801	72.015	1.625676238	0.340963369	4.76789117	1.86164E-06	0.000387355	Significant in both
244.079	71.325	1.166365812	0.246297611	4.73559532	2.18413E-06	0.000424158	Significant in both
320.967	76.018	1.470943946	0.312817061	4.702249742	2.57311E-06	0.000468466	Significant in both
502.874	72.075	2.851413642	0.617834271	4.61517558	3.92763E-06	0.000673011	Significant in both
293.021	90.496	1.611214911	0.353868782	4.553142275	5.28505E-06	0.000855297	Significant in both
324.99	75.983	-1.07265539	0.236779046	-4.530195599	5.89291E-06	0.000903476	Significant in both
318.97	76.169	1.39874138	0.3146534	4.445340114	8.77529E-06	0.001217258	Significant in both
504.871	71.618	1.698108758	0.381653588	4.44934572	8.61323E-06	0.001217258	Significant in both
326.987	76.127	-0.897051574	0.204472212	-4.387156408	1.14842E-05	0.001520615	Significant in both
231.055	75.316	-0.979331332	0.224088571	-4.370286827	1.24083E-05	0.001542764	Significant in both
508.894	71.963	-0.898808474	0.205911332	-4.365026756	1.27107E-05	0.001542764	Significant in both
207.024	79.355	1.414752181	0.324828199	4.35538597	1.32833E-05	0.001547766	Significant in both
277.06	75	-0.956573708	0.220919522	-4.3299646	1.49133E-05	0.001670867	Significant in both
384.945	75.296	-0.937997518	0.217263323	-4.317330258	1.57928E-05	0.001703865	Significant in both
382.948	74.924	-0.918245584	0.215770834	-4.255652005	2.08441E-05	0.002168528	Significant in both
335.094	86.335	0.920558514	0.218211551	4.218651632	2.45768E-05	0.002468694	Significant in both
160.133	55.358	0.98215215	0.234966731	4.179962607	2.91557E-05	0.002831019	Significant in both
106.999	75.195	-0.923597372	0.222413106	-4.152621173	3.28689E-05	0.003088613	Significant in both
165.013	74.843	0.996872946	0.242294109	4.114309464	3.8834E-05	0.003535109	Significant in both
405.096	96.144	1.497577067	0.378094408	3.960854848	7.46819E-05	0.006592377	Significant in both
322.011	233.412	-0.659520767	0.167730856	-3.932018141	8.42357E-05	0.007217018	Significant in both
113.071	101.512	-0.825485772	0.212116563	-3.891661077	9.95603E-05	0.00763208	Significant in both
176.103	102.372	-0.823650646	0.211106264	-3.901592647	9.55619E-05	0.00763208	Significant in both
177.106	100.743	-0.827947149	0.211975543	-3.905861676	9.38902E-05	0.00763208	Significant in both
344.956	69.034	-0.642866072	0.165173957	-3.892054665	9.93988E-05	0.00763208	Significant in both
138.013	74.729	-0.807248276	0.208337374	-3.874716572	0.000106749	0.007818279	Significant in both
159.076	102.224	-0.808125996	0.208963155	-3.867313329	0.000110041	0.007818279	Significant in both

160.08	100.482	-0.813658851	0.21025222	-3.869917995	0.000108872	0.007818279	Significant in both
230.051	75.122	-0.793087123	0.208285309	-3.807695922	0.000140268	0.00972856	Significant in both
198.085	100.113	-0.786710891	0.207402609	-3.793158119	0.000148743	0.010076499	Significant in both
135.022	84.894	0.890316221	0.237469028	3.749188803	0.000177407	0.011745182	Significant in both
400.918	71.845	-0.713078452	0.19081147	-3.737083792	0.000186167	0.012051204	Significant in both
199.088	100.117	-0.73902782	0.199629028	-3.702005805	0.000213902	0.013545559	Significant in both
1054.391	67.307	-0.689089943	0.187296687	-3.679135774	0.000234026	0.014504612	Significant only in sensitivity analysis
245.042	232.059	-0.654465658	0.178504712	-3.666377493	0.000246011	0.014929774	Significant in both
1200.178	67.919	-0.572332755	0.156879466	-3.648232437	0.000264051	0.015697545	Significant in both
155.075	65.529	-0.602825265	0.165878171	-3.634144631	0.000278904	0.016248969	Significant in both
169.027	75.746	-0.693661374	0.194232235	-3.571298944	0.000355215	0.019175544	Significant in both
247.028	75.129	-0.718244304	0.201397376	-3.566304179	0.000362051	0.019175544	Significant in both
325.982	69.952	-0.566905579	0.158842215	-3.568985594	0.000358366	0.019175544	Significant in both
570.665	67.778	-0.867552548	0.24258385	-3.576299685	0.000348492	0.019175544	Significant in both
744.59	42.524	-0.634690054	0.177922914	-3.567219314	0.00036079	0.019175544	Significant only in sensitivity analysis
177.1	100.826	-0.789344775	0.223419056	-3.533023504	0.000410836	0.021370805	Significant in both
171.07	100.464	-0.934384849	0.272284568	-3.431648202	0.000599925	0.030659337	Significant in both
412.307	39.743	1.094325247	0.321665134	3.402063613	0.000668791	0.033386775	Significant only in sensitivity analysis
520.738	66.586	-0.725303742	0.213673727	-3.394445135	0.000687678	0.033386775	Significant only in sensitivity analysis
748.53	42.39	0.806491254	0.237540016	3.395180604	0.000685833	0.033386775	Significant only in sensitivity analysis
293.034	73.377	-0.708793107	0.209350605	-3.385674988	0.000710034	0.033907045	Significant in both
121.072	47.837	0.82501193	0.246287419	3.349793237	0.000808719	0.036913365	Significant only in sensitivity analysis
360.063	88.585	-0.695603715	0.207358005	-3.354602657	0.000794791	0.036913365	Significant in both
455.296	45.646	0.899753858	0.268662515	3.349011518	0.000811004	0.036913365	Significant in both
151.039	271.329	0.813379708	0.245296415	3.315905404	0.000913467	0.040393506	Significant in both
163.06	105.755	0.697549153	0.210398175	3.315376446	0.000915198	0.040393506	Significant in both
265.02	232.971	-0.591692196	0.18039269	-3.280023129	0.001037986	0.045129142	Significant only in sensitivity analysis

C18

Mass to charge ratio	Retention Time	beta	se	zvalue	p value	FDR p value	Overlapping with main analysis
143.035	36.297	1.154986742	0.240957708	4.793317259	1.64046E-06	0.001019704	Significant in both
215.033	30.913	1.244698264	0.26244123	4.742769508	2.10816E-06	0.001019704	Significant in both
217.03	30.51	1.223500695	0.258910007	4.725582867	2.29456E-06	0.001019704	Significant in both
226.065	36.217	1.224096677	0.25702075	4.76263756	1.91079E-06	0.001019704	Significant in both
359.121	49.326	1.694782582	0.351922575	4.815782518	1.46624E-06	0.001019704	Significant in both
218.033	30.739	1.409270514	0.305272132	4.616440105	3.90379E-06	0.001196395	Significant in both
225.062	37.895	1.09619361	0.235945929	4.645952628	3.38511E-06	0.001196395	Significant in both
239.077	35.736	1.09697535	0.238682125	4.595967757	4.30745E-06	0.001196395	Significant in both
113.024	36.458	0.99506934	0.221242737	4.497636181	6.87131E-06	0.001447321	Significant in both
179.056	36.803	1.020770016	0.227820166	4.480595522	7.44351E-06	0.001447321	Significant in both
180.06	36.11	1.07335046	0.23824226	4.505289947	6.62824E-06	0.001447321	Significant in both
219.034	30.853	1.580526248	0.353573074	4.470154445	7.81631E-06	0.001447321	Significant in both
216.035	31.278	1.26183175	0.286497809	4.404332982	1.0611E-05	0.001813662	Significant in both
265.002	30.312	-1.049655405	0.239676451	-4.379468236	1.18969E-05	0.001888212	Significant in both
215.091	31.459	1.065493287	0.246671255	4.319487032	1.56392E-05	0.002316691	Significant in both

174.088	30.043	-0.865438179	0.205698255	-4.207319005	2.58418E-05	0.003434492	Significant in both
209.067	36.808	-0.834617615	0.198550812	-4.203546711	2.62765E-05	0.003434492	Significant in both
267	30.325	-0.948263063	0.23171182	-4.092424222	4.26887E-05	0.004992328	Significant in both
269.088	35.076	1.017353659	0.24793981	4.103228357	4.07425E-05	0.004992328	Significant in both
199.038	31.744	-0.788278443	0.19380231	-4.067435735	4.75333E-05	0.005029477	Significant in both
270.091	34.705	1.165788929	0.286357086	4.071102087	4.67912E-05	0.005029477	Significant in both
220.992	30.561	-0.835843564	0.209697905	-3.985941411	6.72131E-05	0.006788523	Significant in both
225.054	30.806	-0.846731211	0.21683489	-3.904958338	9.42416E-05	0.00910456	Significant only in sensitivity analysis
154.058	35.834	-0.750501055	0.196953961	-3.810540553	0.000138663	0.012464412	Significant in both
220.049	30.642	-0.81157517	0.213137894	-3.807746965	0.000140239	0.012464412	Significant in both
200.041	32.191	-0.596199607	0.15736267	-3.788697834	0.000151439	0.01264604	Significant in both
221.053	30.668	-0.801600495	0.212287304	-3.776017119	0.000159356	0.01264604	Significant in both
223.05	30.675	-0.802866314	0.212574946	-3.776862365	0.000158816	0.01264604	Significant in both
216.971	30.784	1.167785213	0.31318659	3.728720358	0.000192455	0.014746002	Significant in both
223.112	30.845	-0.582247253	0.168648202	-3.452436769	0.000555548	0.041147564	Significant only in sensitivity analysis
283.103	34.333	2.015105583	0.594219753	3.391179058	0.000695926	0.049882193	Significant in both
