# Supplementary material for: Untargeted serum metabolic profiling of diabetes mellitus among Parkinson’s disease patients
Source: NPJ Parkinsons Dis. 2024 May 10;10:100. doi: 10.1038/s41531-024-00711-4 (PMC11087477; doi:10.1038/s41531-024-00711-4)
Supplement: Supplementary file 2 — Reporting Summary [file 41531_2024_711_MOESM2_ESM.pdf]

nature portfolio

Corresponding author(s): Kimberly C. Paul

Last updated by author(s): Jul 13, 2023

Reporting Summary

Nature Portfolio wishes to improve the reproducibility of the work that we publish. This form provides structure for consistency and transparency in reporting. For further information on Nature Portfolio policies, see our Editorial Policies and the Editorial Policy Checklist.

Statistics

For all statistical analyses, confirm that the following items are present in the figure legend, table legend, main text, or Methods section.

n/a	Confirmed	
☐	☒	The exact sample size (n) for each experimental group/condition, given as a discrete number and unit of measurement
☐	☒	A statement on whether measurements were taken from distinct samples or whether the same sample was measured repeatedly
☐	☒	The statistical test(s) used AND whether they are one- or two-sided<br>*Only common tests should be described solely by name; describe more complex techniques in the Methods section.*
☐	☒	A description of all covariates tested
☐	☒	A description of any assumptions or corrections, such as tests of normality and adjustment for multiple comparisons
☐	☒	A full description of the statistical parameters including central tendency (e.g. means) or other basic estimates (e.g. regression coefficient) AND variation (e.g. standard deviation) or associated estimates of uncertainty (e.g. confidence intervals)
☐	☒	For null hypothesis testing, the test statistic (e.g. F, t, r) with confidence intervals, effect sizes, degrees of freedom and P value noted<br>*Give P values as exact values whenever suitable.*
☒	☐	For Bayesian analysis, information on the choice of priors and Markov chain Monte Carlo settings
☒	☐	For hierarchical and complex designs, identification of the appropriate level for tests and full reporting of outcomes
☐	☒	Estimates of effect sizes (e.g. Cohen's d, Pearson's r), indicating how they were calculated

Our web collection on statistics for biologists contains articles on many of the points above.

Software and code

Policy information about availability of computer code

Data collection	No software used.
Data analysis	R version 4.1.3

For manuscripts utilizing custom algorithms or software that are central to the research but not yet described in published literature, software must be made available to editors and reviewers. We strongly encourage code deposition in a community repository (e.g. GitHub). See the Nature Portfolio guidelines for submitting code & software for further information.

Data

Policy information about availability of data

All manuscripts must include a data availability statement. This statement should provide the following information, where applicable:

- Accession codes, unique identifiers, or web links for publicly available datasets
- A description of any restrictions on data availability
- For clinical datasets or third party data, please ensure that the statement adheres to our policy

The data used in this study will be made available upon reasonable request to the authors. The code is available on GitHub at https://github.com/sherlockli1/metabolomics-PD-and-DM.git. The complete summary statistics for the metabolic features associated with T2DM are available through figshare (10.6084/m9.figshare.22589464).

Research involving human participants, their data, or biological material

Policy information about studies with human participants or human data. See also policy information about sex, gender (identity/presentation), and sexual orientation and race, ethnicity and racism.

Reporting on sex and gender	We used gender as it was self identified by study participants. Informed consent was obtained from all study participants. There were 406 male PD patients, 230 female PD patients, 119 non-PD male participants and 134 non-PD female participants. We did not exclude participants based on sex or gender in the study design. We performed gender stratified analysis.
Reporting on race, ethnicity, or other socially relevant groupings	The race and ethnicity was self reported by study participants. We controlled race/ethnicity as potential confounder. We controlled whether they were of European ancestry or not in the model. We also performed stratified analysis by European ancestry.
Population characteristics	15% of the 636 PD patients had T2DM and the mean age of PD diagnosis was similar in PD patients with T2DM (68 y, SD=10 y) and without T2DM (67 y, SD=10 y). PD patients with and without T2DM were also similar in terms of the percentages of men, people with ≥12 years of education, and have never smoked. However, double the PD patients with T2DM (42%) than those without reported non-European ancestry which includes Latino, Black, Asian, and Native American. In total, 253 non-PD participants were included in the analysis with 36 (14%) having T2DM and the patterns of age, lifestyle, and ethnicity were also similar to T2DM.
Recruitment	PD patients were recruited as part of a community-based case-control study, the Parkinson's Gene and Environment (PEG) study. Eligibility criteria for cases included: living in California for five years at minimum, having been diagnosed with PD for less than or equal to three years for PEG1 and less than or equal to five years for PEG2, and agreeing to participate in the study. PD patients were recruited from local clinics, neurologists, medical groups, radio advertisements, and the California PD registry. Although these PD patients were newly onset patients, the majority of the PD patients (>70%) were taking medication. Population controls for PD were randomly sampled from the study area using either Medicare enrollee lists (prior to Health Insurance Portability and Accountability Act) or residential parcels listed in property tax assessor records. PEG1 and PEG2 also enrolled a set of household controls for PD patients. These household controls for PD were limited to one per household. Eligibility criteria for controls included: being more than 35 years old, living in California for five years at minimum, and not being diagnosed with PD. Household control had an additional requirement of living in the same household with the PD participants for at least 1 year.
Ethics oversight	University of California, Los Angeles

Note that full information on the approval of the study protocol must also be provided in the manuscript.

Field-specific reporting

Please select the one below that is the best fit for your research. If you are not sure, read the appropriate sections before making your selection.

☐ Life sciences ☒ Behavioural & social sciences ☐ Ecological, evolutionary & environmental sciences

For a reference copy of the document with all sections, see nature.com/documents/nr-reporting-summary-flat.pdf

Behavioural & social sciences study design

All studies must disclose on these points even when the disclosure is negative.

Study description	Untargeted metabolomics analysis for T2DM among PD patients
Research sample	PD patients were recruited as part of a community-based case-control study, the Parkinson's Gene and Environment (PEG) study from central California (Kern, Fresno, and Tulare). PD patients with and without T2DM were also similar in terms of the percentages of men, people with ≥12 years of education, and have never smoked. However, double the PD patients with T2DM (42%) than those without reported non-European ancestry.
Sampling strategy	We recruited PD patients from three counties in central California. Our non-PD participants were population-based controls and household controls and thus should have been representative of the confounder distribution in the three counties (source population).
Data collection	We collected blood sample from participants and covariate information using paper and pen through in-person, mailing, or phone interview.
Timing	Idiopathic PD patients were recruited between 2000 and 2017 in two separate study waves, referred to as PEG1 and PEG2. PEG1 participants were recruited between 2001 and 2007. PEG 2 participants were recruited between 2011 and 2017.
Data exclusions	We excluded participants without metabolomics data (194 PD patients and 607 non-PD participants excluded from analysis).
Non-participation	We screened 1167 participants in PEG1 and 2713 participants in PEG2. 357 PD patients and 470 PD patients completed baseline

Non-participation	data. Participants that were excluded were either not eligible to participate, refused to participant, too ill or deceased, did not have PD, or did not have complete data.
Randomization	There were no randomization. We controlled for age of PD diagnosis, gender, education, smoking, non-European ancestry, and study wave to control for potential confounding.

Reporting for specific materials, systems and methods

We require information from authors about some types of materials, experimental systems and methods used in many studies. Here, indicate whether each material, system or method listed is relevant to your study. If you are not sure if a list item applies to your research, read the appropriate section before selecting a response.

Materials & experimental systems

n/a	Involved in the study
☒	☐ Antibodies
☒	☐ Eukaryotic cell lines
☒	☐ Palaeontology and archaeology
☒	☐ Animals and other organisms
☒	☐ Clinical data
☒	☐ Dual use research of concern
☒	☐ Plants

Methods

n/a	Involved in the study
☒	☐ ChIP-seq
☒	☐ Flow cytometry
☒	☐ MRI-based neuroimaging
